# Time spent above optimal cerebral perfusion pressure is not associated with failure to improve in outcome in traumatic brain injury

**DOI:** 10.1186/s40635-023-00579-3

**Published:** 2023-12-14

**Authors:** Kevin Y. Stein, Logan Froese, Alwyn Gomez, Amanjyot Singh Sainbhi, Nuray Vakitbilir, Younis Ibrahim, Abrar Islam, Izabella Marquez, Fiorella Amenta, Tobias Bergmann, Frederick A. Zeiler

**Affiliations:** 1 Biomedical Engineering, Price Faculty of Engineering, University of Manitoba, Winnipeg, MB Canada; 2 Section of Neurosurgery, Department of Surgery, Rady Faculty of Health Sciences, University of Manitoba, Winnipeg, MB Canada; 3 Department of Human Anatomy and Cell Science, Rady Faculty of Health Sciences, University of Manitoba, Winnipeg, Canada; 4 Undergraduate Engineering, Price Faculty of Engineering, University of Manitoba, Winnipeg, MB Canada; 5 Department of Clinical Neuroscience, Karolinska Institutet, Stockholm, Sweden; 6 Division of Anaesthesia, Department of Medicine, Addenbrooke’s Hospital, University of Cambridge, Cambridge, United Kingdom; 7 Pan Am Clinic Foundation, Winnipeg, MB Canada

**Keywords:** Optimal cerebral perfusion pressure, CPPopt, Outcome transition, Traumatic brain injury, TBI

## Abstract

**Background:**

Optimal cerebral perfusion pressure (CPPopt) has emerged as a promising personalized medicine approach to the management of moderate-to-severe traumatic brain injury (TBI). Though literature demonstrating its association with poor outcomes exists, there is yet to be work done on its association with outcome transition due to a lack of serial outcome data analysis. In this study we investigate the association between various metrics of CPPopt and failure to improve in outcome over time.

**Methods:**

CPPopt was derived using three different cerebrovascular reactivity indices; the pressure reactivity index (PRx), the pulse amplitude index (PAx), and the RAC index. For each index, % times spent with cerebral perfusion pressure (CPP) above and below its CPPopt and upper and lower limits of reactivity were calculated. Patients were dichotomized based on improvement in Glasgow Outcome Scale-Extended (GOSE) scores into *Improved* vs. *Not Improved* between 1 and 3 months, 3 and 6 months, and 1- and 6-month post-TBI. Logistic regression analyses were then conducted, adjusting for the International Mission for Prognosis and Analysis of Clinical Trials (IMPACT) variables.

**Results:**

This study included a total of 103 patients from the Winnipeg Acute TBI Database. Through Mann–Whitney *U* testing and logistic regression analysis, it was found that % time spent with CPP below CPPopt was associated with failure to improve in outcome, while % time spent with CPP above CPPopt was generally associated with improvement in outcome.

**Conclusions:**

Our study supports the existing narrative that time spent with CPP below CPPopt results in poorer outcomes. However, it also suggests that time spent above CPPopt may not be associated with worse outcomes and is possibly even associated with improvement in outcome.

**Supplementary Information:**

The online version contains supplementary material available at 10.1186/s40635-023-00579-3.

## Introduction

The concept of optimal CPP (CPPopt) was first developed in 2002 when Steiner et al. were able to demonstrate the feasibility of using the relationship between CPP and cerebrovascular reactivity, a surrogate for cerebral autoregulatory capacity, to calculate an individualized CPP target that optimizes cerebrovascular reactivity [1]. They accomplished this by plotting CPP against the pressure reactivity index (PRx—correlation between ICP and mean arterial pressure [MAP]), producing a U-shaped curve, and pinpointing the CPP value that minimized PRx. As a note, more negative PRx generally indicates more intact cerebral autoregulation. The terms “upper limit of reactivity” (ULR) and “lower limit of reactivity” (LLR) describe the upper and lower CPP thresholds past which cerebrovascular reactivity becomes deranged [2]. These can be visualized graphically as the points, where the U-shaped curve intersects a preset PRx value that has been chosen to represent the threshold past which cerebrovascular reactivity becomes impaired.

Subsequent research has demonstrated the feasibility of continuously determining such personalized CPP targets, rendering their application in clinical settings viable [3–5]. It should be noted that other cerebrovascular reactivity indices, such as the pulse amplitude index (PAx—correlation between pulse amplitude of ICP (AMP) and MAP) and RAC (the correlation (R) between AMP (A) and CPP (C)), can also be used to for CPPopt derivation. At this time, there are no definite conclusions on which cerebrovascular reactivity index produces the most superior CPPopt calculation. However, a multi-center study by Zeiler et al. found that RAC-based CPPopt produced comparable outcome associations as PRx-based CPPopt, while PAx-based CPPopt failed to produce statistically significant associations with outcome [6].

Poorer outcomes in traumatic brain injury (TBI) have been shown to be strongly associated with larger deviation between actual CPP and CPPopt [1, 3, 7, 8], as well as greater duration of deviation [2, 6, 8]. In addition, CPPopt targets have been shown to have more robust associations with patient outcomes compared to guideline-based CPP targets of 60–70 mmHg [3, 6–8]. Therefore, CPPopt offers a potential way forward in TBI management, where mortality rates have remained relatively unchanged over the past few decades despite improvements in our capabilities to achieve guideline-based targets [9]. There has been mixed evidence in regard to having actual CPP above vs. below CPPopt. The original study by Steiner et al*.* found statistically significant associations with worse outcome for both CPP above and below CPPopt [1]. Other studies found that CPP below CPPopt was associated with mortality, while CPP above CPPopt was associated with severe disability [3, 10]. Some other studies found that only time spent below CPPopt was statistically associated with worse outcomes [2, 6]. Time spent, as well as dose-time, with CPP below the LLR has also been shown to be associated with mortality and unfavorable outcome [2, 11]. On the other hand, time spent above the ULR has been shown to be associated with unfavorable outcome but not with mortality [2].

Though a strong relationship between deviations from CPPopt and poor long-term outcomes has already been demonstrated, the current literature has focused completely on single point measures of outcome. Therefore, it remains largely unknown whether time spent with actual CPP deviated from CPPopt is associated with improvement in outcome across time; information which would allow for improvements in prognostic outcome trajectory modelling. Using a similar methodology to a previous study conducted by our lab on the association between cerebrovascular reactivity derangement and transition in outcome [12], we investigated the association between CPPopt; derived using PRx, PAx, and RAC; and failure to improve in outcome post-TBI.

## Methods

### Patient population and data collection

As part of the ongoing Winnipeg Acute TBI Database, all adult (≥ 18 years) moderate-to-severe TBI patients admitted to the Surgical Intensive Care Unit (ICU) at the Health Sciences Centre for invasive cerebral physiologic monitoring have their data prospectively collected [13]. All patients undergo invasive ICP and arterial blood pressure (ABP) monitoring during their ICU stay, as per Brain Trauma Foundation (BTF) guidelines. Intra-parenchymal strain gauge probes (Codman ICP MicroSensor; Codman & Shurtlef Inc., Raynham, MA, USA) placed in the frontal lobe or external ventricular drains are used to monitor ICP, while radial or femoral arterial lines connected to pressure transducers (Baxter Healthcare Corp. CardioVascular Group, Irvine, CA, USA) zeroed at the level of the tragus [14, 15] are used to monitor ABP. Patients receive standard-of-care consistent with the BTF guidelines, which involves maintaining ICP below 20 mmHg or 22 mmHg, to thwart intracranial hypertension, and CPP above 60 mmHg, to prevent insufficient cerebral perfusion [16]. It should be noted that local practice does not warrant aggressive intervention to combat elevated CPP unless a direct association between elevated CPP and subsequent ICP elevations is evident.

Data collected as part of the Winnipeg Acute TBI Database includes patient demographics, admission characteristics, imaging profiles, treatment descriptions, and outcome grading, all of which are primarily collected through patient files. In addition, all physiologic signals available from patients’ ICU monitors are recorded in time-series at a sampling frequency of at least 100 Hz using Intensive Care Monitoring “Plus” (ICM +) (Cambridge Enterprise Ltd, Cambridge, UK, http://icmplus.neurosurg.cam.ac.uk), through either direct digital data transfer or analog-to-digital signal conversion (DT9804/DT9826, Data Translations, Marlboro, MA, USA). To ensure data quality, a two-tier approach involving both manual and automated techniques is employed to eliminate ICP and ABP signal artifacts. In general, this includes removal of data segments that lack proper waveform morphology or have implausibly low or high values. For cases where ICP is monitored using an external ventricular drain (*n* = 4), any artifacts related to drain opening are addressed through manual curation.

Following discharge from the ICU, patients undergo routine follow-up appointments at 1, 3, and 6 months. During these visits, patients have their overall outcome status evaluated using the Glasgow Outcome Scale-Extended (GOSE) [17]. These assessments are conducted by experienced specialist surgeons through structured interviews with the patients themselves and, when applicable, their designated proxies. For the purposes of this study, all complete data sets collected, since the inception of the Winnipeg Acute TBI Database in January of 2019 was accessed. Patients who did not have their 6-month outcome assessed by May of 2023 were excluded.

### Ethical considerations

Ethics approval for all facets of data collection for the ongoing Winnipeg Acute TBI Database have been obtained from the University of Manitoba Health Research Ethics Board (H2017:181, H2017:188), the Shared Health Services Manitoba Research Impact Committee, and the Patient Privacy Offices of Manitoba (RI2017:078 and RI:2017:076). Furthermore, since all collected data are thoroughly de-identified to the point that it cannot be traced back to any individual patient, the data collection process has been approved to operate under a waived consent model by both the research ethics board and the provincial patient privacy offices of Manitoba. Retrospective access of this database for this outcome analysis has also been approved by the local ethics board (H2020:118).

### Signal processing

Post-acquisition signal processing was carried out using ICM + . To derive AMP, Fourier analysis of the fundamental amplitude of the ICP pulse waveform was calculated over a 10-s window, updating every 10 s [18–20]. Next, to concentrate on the frequency range pertinent to cerebrovascular reactivity [21, 22] and mitigate the influence of the respiratory cycle [9], a 10-s non-overlapping moving average filter was applied to down-sample ICP and ABP (yielding MAP). The difference between MAP and ICP was then used to calculate CPP, as described by the following formula: CPP = MAP–ICP. To evaluate cerebrovascular reactivity, three ICP-based surrogate indices were derived: PRx, PAx, and RAC. PRx was computed by assessing the Pearson correlation between 30 successive 10-s windows of ICP and MAP, continually updated on a minute-by-minute basis [23–25]. In a similar manner, PAx and RAC were computed using AMP and MAP, and AMP and CPP, respectively [19, 26].

CPPopt was derived in ICM + using the methodology outlined by Aries et al.[3]. Utilizing a minute-by-minute updating 4-h sliding window, a 5-min median CPP time trend was computed alongside each of the cerebrovascular reactivity indices. PRx, PAx, and RAC values were then divided and averaged into 5 mmHg bins of CPP. Employing automatic parabolic curve fitting, the CPP values corresponding to the lowest PRx, PAx, and RAC values were identified, thereby establishing CPPopt–PRx, CPPopt–PAx, and CPPopt–RAC. ΔCPPopt values were then determined every minute by subtracting the respective CPPopt value from the actual CPP value using the following formula: CPP—CPPopt. To calculate the ULR and LLR values for each cerebrovascular reactivity metric, the two points, where the parabolic curve intersected literature defined thresholds of + 0.25 (for PRx), + 0.25 (for PAx), and -0.05 (for RAC) were identified [24, 27, 28]. The LLR was identified as the lower CPP value, where the curve first crosses the threshold line and descends towards the most intact cerebrovascular reactivity, while the ULR was identified as the higher CPP value, where the curve crosses the threshold and ascends towards impaired reactivity. Finally, all data were down-sampled to minute-by-minute resolution and exported as comma-separated values (CSV) files for further processing in R Statistical Computing Software (Version 4.1.0, R Core Team (2020). R: a language and environment for statistical computing. R Foundation for Statistical Computing, Vienna, Austria. URL https://www.R-project.org/).

For each patient, average CPPopt values were calculated for all three cerebrovascular reactivity indices, as well as the following metrics:

1. % time with ΔCPPopt > 5 mmHg
2. % time with ΔCPPopt > 10 mmHg
3. % time with ΔCPPopt < -5 mmHg
4. % time with ΔCPPopt < -10 mmHg
5. % time with CPP > ULR
6. % time with CPP < LLR

### Statistical analysis

The statistical analysis methodology used in this study is similar to that of a previous study from our lab [12]. All statistical testing was performed using R Statistical Computing Software with the following packages: *MASS, purrr, fmsb, pROC, broom, ggplot2,* and *verification*. For all continuous variables, we assessed normality using the Shapiro–Wilk test. All physiologic variables were revealed to be non-parametric in nature and were subsequently summarized using medians and interquartile ranges (IQR). Demographic data were summarized using raw counts or medians and IQR where appropriate.

Patients were dichotomized based on GOSE scores into *Alive* (GOSE 2–8) vs. *Dead* (GOSE 1) and *Favorable* (GOSE 5–8) vs. *Unfavorable* (GOSE 1–4) at 1-, 3-, and 6-month post-TBI. Next, patients were dichotomized based on transition in outcome into *Improved* vs. *Not Improved* between 1 and 3 months, 3 and 6 months, and 1 and 6 months. A patient was classified as having improved in outcome if their GOSE score had increased between the two timepoints, and as having failed to improve in outcome if their GOSE score had either decreased or remained the same. We performed a similar dichotomization, where those who had died (GOSE 1) were excluded. In this dichotomization, only those who were both alive and failed to improve in outcome were classified as having *Not Improved*. As part of an additional secondary analysis, patients were also trichotomized based on age (< 30, 30–60, > 60).

To assess any differences in continuous and non-continuous variables between the various dichotomized groups, we utilized Mann–Whitney *U* testing and Chi-square testing, respectively. We then employed univariate logistic regression analysis to investigate the association that the various metrics of CPPopt, described at the end of the signal processing section, have with transition in patient outcome. To confirm whether any observed associations would remain significant after accounting for established outcome-associated factors, such as intracranial hypertension [29], we conducted a multivariable logistic regression analysis. The standardized multivariable models used in this analysis comprised of the International Mission for Prognosis and Analysis of Clinical Trials (IMPACT) Core model, Core + computed tomography (CT) model, Core + CT + % time with ICP > 20 mmHg model, and Core + CT + % time with ICP > 22 mmHg model [12, 30]. The components of the IMPACT Core are age, admission Glasgow Coma Scale (GCS) motor score, and pupillary response (normal bilaterally, unilaterally unreactive, or bilaterally unreactive), while the CT variables consisted of admission Marshall CT grade, presence of traumatic subarachnoid hemorrhage (tSAH), and presence of extradural hematoma [31, 32]. The various CPPopt metrics described earlier were then added to these models.

Using bootstrapping methods, the area under the receiver operating curve (AUC) values and their associated confidence intervals were calculated for each model and reported alongside with Akaike information criteria (AIC), *p* values, and Nagelkerke’s pseudo-R^2^. Finally, the additional variance in outcome transition attributable to the CPPopt metrics, over the standardized multivariable models, was assessed by calculating the difference in Nagelkerke’s pseudo-R^2^. All *p* values were adjusted for multiple comparisons using the false discovery rate (FDR) method, developed by Benjamini and Hochberg, with alpha set to 0.05 [33]. Given the exploratory nature of this study, the FDR approach was selected over the conventional Bonferroni method to preserve statistical power while simultaneously addressing the need for some degree of correction for multiple comparisons.

## Results

### Patient population

At the time of analysis, a total of 110 patients from the Winnipeg Acute TBI Database had complete data sets with 6-month GOSE scores. However, CPPopt values were not possible to calculate in seven of the patients, leaving 103 patients to be included in this study. A total of four patients had their ICP monitored using an EVD, while intra-parenchymal strain gauge probes were used in the rest of the cohort. The median age of the entire cohort was 42 years (IQR = 27–56.5 years) and the median admission GCS score was 7 (IQR = 4–8). The proportion of the cohort that was male was 81%. The median recording duration was 69.6 h (IQR = 36.37–122.2 h). Patient demographics and cerebral physiology can be found summarized in Tables 1 and 2, respectively.

**Table 1:** Patient demographics

Demographic variable	Median (IQR) or raw numbers (%)
Number of patients	103
Age (years)	42 (27–56.5)
Sex	
Male	83 (81%)
Female	20 (19%)
Admission GCS total	7 (4–8)
Admission GCS motor	4 (2–5)
Admission GCS eyes	1 (1–2)
Admission GCS verbal	1 (1–2)
Admission pupil response	
Bilaterally reactive	62 (60%)
Unilaterally unreactive	23 (22%)
Bilaterally unreactive	18 (17%)
Marshall CT grade	4 (3–5)
Rotterdam CT grade	4 (4–5.5)
Helsinki ct score	6 (4–9)
Stockholm CT score	3.2 (2.5–3.75)
GOSE	
1 month	4 (1–6)
3 months	5 (1–6)
6 months	6 (1–7)
Number alive (GOSE > 1)	
1 month	69 (67%)
3 months	67 (65%)
6 months	67 (65%)
Number dead (GOSE of 1)	
1 month	34 (33%)
3 months	36 (35%)
6 months	36 (35%)
Number favorable (GOSE 5–8)	
1 month	50 (49%)
3 months	61 (59%)
6 months	63 (61%)
Number unfavorable (GOSE 1–4)	
1 month	53 (51%)
3 months	42 (41%)
6 months	40 (39%)
Number with hypoxia episode	36 (35%)
Number with hypotension episode	11 (11%)
Number with traumatic SAH	99 (96%)
Number with epidural hematoma	11 (11%)
Admission hemoglobin	132 (114–145)
Admission serum glucose	8.1 (7–10.5)
Length of hospital stay	22 (8.75–42.75)
Length of ICU stay	8 (4–15)

*CT* computed tomography, *GCS* Glasgow Coma Scale, *GOSE* Glasgow Outcome Scale-Extended, *ICU* intensive care unit, *IQR* interquartile range, *SAH* subarachnoid hemorrhage

**Table 2:** Patient cerebral physiology summary

Physiologic metric	Median (IQR)
Duration of physiologic monitoring (hours)	69.60 (36.37–122.2)
Mean MAP (mmHg)	83.70 (79.22–88.15)
Mean ICP (mmHg)	9.010 (5.658–12.43)
% Time ICP > 20 mmHg	1.172 (0.02757–5.752)
% Time ICP > 22 mmHg	0.7295 (0–3.210)
Mean CPP (mmHg)	73.74 (69.8–79.85)
% Time CPP < 60 mmHg	4.239 (1.246–9.532)
% Time CPP > 70 mmHg	63.38 (45.10–77.95)
Mean PRx	0.1481 (0.03969–0.2767)
% Time PRx > 0	65.68 (53.57–82.22)
% Time PRx > 0.25	40.16 (28.22–57.05)
% Time PRx > 0.35	30.12 (19.85–45.14)
Mean PAx	0.002225 (−0.1046–0.1171)
% Time PAx > 0	50.6 (35.35–65.87)
% Time PAx > 0.25	24.29 (13.09–38.87)
Mean RAC	−0.2222 (−0.3793 to −0.04453)
% Time RAC > −0.10	34.43 (20.53–55.35)
% Time RAC > −0.05	29.89 (17.95–49.42)
CPPopt–PRx	74.30 (69.29–80.02)
% Time ΔCPPopt–PRx > 5 mmHg	24.46 (16.41–33.77)
% Time ΔCPPopt–PRx > 10 mmHg	10.55 (6.787–19.32)
% Time ΔCPPopt–PRx < −5 mmHg	24.20 (15.96–35.79)
% Time ΔCPPopt–PRx < −10 mmHg	10.02 (4.775–19.14)
% Time CPP > ULR–PRx	14.45 (7.156–22.45)
% Time CPP < LLR–PRx	11.49 (5.594–26.58)
CPPopt–PAx	74.97 (71.64–82.50)
% Time ΔCPPopt–PAx > 5 mmHg	21.76 (13.58–31.45)
% Time ΔCPPopt–PAx > 10 mmHg	9.472 (4.634–17.82)
% Time ΔCPPopt–PAx < −5 mmHg	30.15 (19.96–40.68)
% Time ΔCPPopt–PAx < −10 mmHg	15.43 (8.104–25.25)
% Time CPP > ULR–PAx	5.682 (2.755–12.74)
% Time CPP < LLR–PAx	6.621 (2.447–16.77)
CPPopt–RAC	73.24 (69.48–79.30)
% Time ΔCPPopt–RAC > 5 mmHg	25.63 (14.93–37.19)
% Time ΔCPPopt–RAC > 10 mmHg	11.29 (6.417–20.08)
% Time ΔCPPopt–RAC < −5 mmHg	22.57 (13.45–35.27)
% Time ΔCPPopt–RAC < −10 mmHg	10.35 (3.166–18.27)
% Time CPP > ULR–RAC	3.676 (1.691–8.185)
% Time CPP < LLR–RAC	4.160 (1.058–9.772)

*AMP* pulse amplitude of ICP, *CPP* cerebral perfusion pressure, *CPPopt* cerebral perfusion pressure optimum, *ΔCPPopt* CPP—CPPopt, *ICP* intracranial pressure, *IQR* interquartile range, *LLR* lower limit of reactivity, *MAP* mean arterial pressure, *mmHg* millimeters of mercury, *PAx* pulse amplitude index, *PRx* pressure reactivity index, *RAC* correlation (R) between slow-waves of AMP (A) and CPP (C), *ULR* upper limit of reactivity

### Comparisons between dichotomized groups

The results of the Mann–Whitney *U* and Chi-square testing for *Alive* vs. *Dead* and *Favorable* vs. *Unfavorable* are available in Additional file 1: Appendices A–C, organized by the respective month used for dichotomization, months 1, 3, and 6.

The results of the Mann–Whitney *U* and Chi-square testing for transition in outcome (*Improved* vs. *Not Improved*) between 1 and 3 months, and 1 and 6 months are presented in Table 3. Only two demographic variables showed statistically significant differences between the two cohorts. Age was greater in the *Not Improved* group, and length of hospital stay was greater in the *Improved* group. For the cerebrovascular reactivity indices, only PAx and RAC produced statistically significant differences. Mean PAx and RAC, as well as % time spent above their thresholds, were consistently higher in the *Not Improved* group for both transition periods. PRx failed to produce any statistically significant results. All three CPPopt means; CPPopt–PRx, CPPopt–PAx, and CPPopt–RAC; failed to produce any significant results. However, the following CPPopt metrics were significantly greater in the *Improved* group for the 1–6-month transition period: % time with ΔCPPopt–PRx > 10 mmHg, % time with ΔCPPopt–PAx > 5 mmHg, % time with ΔCPPopt–PAx > 10 mmHg, % time with ΔCPPopt–RAC > 5 mmHg, and % time with ΔCPPopt–RAC > 10 mmHg. In addition, % time with CPP below the LLR–PAx was significantly greater in the *Not Improved* group. No CPPopt metric produced statistically significant results for the 1–3-month transition period. The Mann–Whitney *U* and Chi-square testing results for the 3–6-month transition period are presented in Additional file 1: Appendix D, as none of the variables, neither demographic nor physiologic, produced statistically significant differences.

**Table 3:** Mann–Whitney U/Chi-square testing of physiologic and demographic data for improved/not improved (1–3 months and 1–6 months)

Variable	1 month → 3 months			1 month → 6 months		
	Improved median (IQR)	Not improved median (IQR)	*p* value	Improved median (IQR)	Not improved median (IQR)	*p* value
Age (years)	37.5 (25–49)	49 (33–62)	**0.0263**	36 (24–50)	52 (36–65.2)	**0.0056**
Sex (% Male)	83.30%	78.70%	0.9373	84.90%	76%	0.5580
Admission GCS total	7 (5–8)	6 (4–8)	0.9263	7 (5–8)	6 (4–8)	0.5700
Admission GCS motor	4.5 (3–5)	4 (2–5)	0.4953	4 (3–5)	4 (2–5)	0.3518
Admission GCS eyes	1 (1–2)	1 (1–2)	0.9322	1 (1–2)	1 (1–2)	1.0000
Admission GCS verbal	1 (1–1)	1 (1–2)	0.5914	1 (1–1)	1 (1–2)	0.8315
Admission pupil response (% bilaterally reactive)	64.30%	57.40%	0.7770	62.30%	58%	0.8016
Marshall CT grade	4 (3–5)	5 (3–5)	0.5976	4 (3–5)	5 (4–5)	0.3099
Rotterdam CT grade	4 (3.25–5)	4 (4–6)	0.9131	4 (4–5)	4 (4–6)	0.8851
Helsinki CT score	6 (4–9)	6 (4–9)	0.9398	6 (4–9)	7 (5–9)	0.5700
Stockholm CT score	3.1 (2.6–3.77)	3.2 (2.4–3.7)	0.9338	3.1 (2.9–3.8)	3.2 (2.22–3.68)	0.8276
Number with hypoxia episode	38.10%	32.80%	0.9457	37.70%	32%	0.7992
Number with hypotension episode	14.30%	8.20%	0.7857	17%	4%	0.1532
Number with traumatic SAH	97.60%	95.10%	0.9593	98.10%	94%	0.7722
Number with epidural hematoma	11.90%	9.80%	0.9920	13.20%	8%	0.7669
Admission hemoglobin	136 (120–148)	129 (113–143)	0.3534	138 (116–149)	128 (113–140)	0.1012
Admission serum glucose	8.05 (7.15–10.1)	8.2 (7–11)	0.9622	8.1 (7–10.2)	8.05 (7–10.9)	0.8462
Length of hospital stay	42 (30–79)	11 (6.5–25.5)	** < 0.0001**	36.5 (22–62.5)	9.5 (6–21)	** < 0.0001**
Length of ICU stay	11.5 (4.25–18)	7 (4–11)	0.1471	11 (4–17)	6.5 (4–11)	0.1110
Mean MAP (mmHg)	84.1 (80.7–92.1)	82.8 (78.6–87.8)	0.4825	84.6 (81.1–89.4)	81.2 (78.1–87.9)	0.1200
Mean ICP (mmHg)	8.95 (5.51–12)	9.01 (5.98–13.3)	0.8964	9.13 (5.95–12.5)	8.73 (5.5–12.3)	0.9920
% Time ICP > 20 mmHg	1.06 (0–5.19)	1.19 (0.136–5.76)	0.5918	1.66 (0–5.76)	1.14 (0.142–5.26)	0.7571
% Time ICP > 22 mmHg	0.489 (0–2.58)	0.767 (0.0852–4)	0.4845	0.923 (0–3.02)	0.667 (0.0865–3.33)	0.6923
Mean CPP (mmHg)	74.4 (71.2–80.3)	73.4 (69–78.6)	0.3447	75.1 (71.3–80.6)	73 (68.1–76.5)	0.0659
% Time CPP < 60 mmHg	4.14 (1.47–8.85)	4.5 (1.04–9.87)	0.9610	3.81 (1.45–7.7)	5.3 (1.22–14.3)	0.3558
% Time CPP > 70 mmHg	65.7 (54.5–80.7)	61.4 (39.6–76.1)	0.3449	67.2 (54.9–82.8)	56 (36.2–73.5)	0.0657
Mean PRx	0.102 (0.0111–0.212)	0.178 (0.074–0.363)	0.0895	0.136 (0.0347–0.237)	0.177 (0.079–0.364)	0.1375
% Time PRx > 0	60.2 (51.6–74.1)	68.5 (58.9–86.6)	0.0945	62.5 (51.9–78.4)	67.7 (59–87.2)	0.1301
% Time PRx > 0.25	35.9 (26.4–46.9)	45 (31.8–68.2)	0.0925	38.6 (27.3–54.5)	42.7 (32.3–68.6)	0.1555
% Time PRx > 0.35	26.7 (19–35.3)	33.4 (23.9–57.3)	0.0860	27.4 (19.1–39.6)	32.6 (23.4–56.7)	0.1590
Mean PAx	−0.0507 (−0.142 to 0.0302)	0.0525 (−0.0984 to 0.232)	**0.0115**	−0.0437 (−0.142 to 0.0607)	0.0463 (−0.0968 to 0.231)	**0.0464**
% Time PAx > 0	43.2 (34–53.8)	58.9 (38.7–76.1)	**0.0133**	44.8 (33.9–58.9)	55.6 (38.8–77)	**0.0443**
% Time PAx > 0.25	17.7 (12.6–27.5)	30.7 (17.3–51.3)	**0.0125**	18.5 (12.5–29.4)	30 (17.3–50.6)	**0.0440**
Mean RAC	−0.328 (−0.432 to −0.175)	−0.127 (−0.313 to 0.00493)	**0.0085**	−0.285 (−0.425 to −0.153)	−0.123 (−0.314 to 0.0205)	**0.0161**
% Time RAC > -0.10	25.3 (13–42.2)	43.4 (27.4–61.9)	**0.0107**	29.4 (14.3–46)	44.8 (27.3–63.3)	**0.0282**
% Time RAC > -0.05	22.3 (10.8–37)	38.6 (23.3–56.9)	**0.0116**	25.6 (12–39.6)	39.4 (23.2–58.6)	**0.0240**
Mean CPPopt–PRx	74.4 (68.7–81.2)	74.1 (69.5–79.9)	0.9416	74.4 (70–83.6)	73.9 (69.2–79.1)	0.7562
% Time ΔCPPopt–PRx > 5 mmHg	26.5 (17.9–33.8)	23.7 (15–33.4)	0.5590	27.9 (18.6–34.9)	21.6 (14.5–29.1)	0.0665
% Time ΔCPPopt–PRx > 10 mmHg	12.5 (8.88–19.8)	10.1 (5.47–17.3)	0.1837	12.5 (8.99–20.8)	8.62 (5.49–15.7)	**0.0465**
% Time ΔCPPopt–PRx < −5 mmHg	25.4 (15.7–32.8)	24.1 (17.4–37.4)	0.9213	23.6 (14.7–32.9)	25.7 (19.1–37.9)	0.2451
% Time ΔCPPopt–PRx < −10 mmHg	11.9 (5.17–19)	9.82 (4.5–18.9)	0.9771	9.09 (4.5–17.9)	11.7 (5.61–20.6)	0.3974
% Time CPP > ULR–PRx	12.9 (6.27–20.3)	15 (7.51–22.6)	0.5879	14.5 (6.94–24)	14.5 (7.74–22)	0.9307
% Time CPP < LLR–PRx	9.15 (5.36–23.1)	14.2 (5.91–26.7)	0.4801	9.86 (4.58–23.4)	14.4 (6.06–27)	0.2648
Mean CPPopt–PAx	74.6 (71.8–82.3)	75.7 (71.2–82.6)	0.9458	75.7 (71.8–82.8)	74.9 (71.2–79.6)	0.7410
% Time ΔCPPopt–PAx > 5 mmHg	26.3 (15.7–35.1)	19.8 (13–28.7)	0.1479	27.4 (18.7–35.1)	18.3 (12.3–27.4)	**0.0281**
% Time ΔCPPopt–PAx > 10 mmHg	12.3 (6.38–22.5)	8 (4.43–14.2)	0.0924	12.9 (6.57–22.7)	7.03 (4.2–12.1)	**0.0323**
% Time ΔCPPopt–PAx < −5 mmHg	29.9 (18.3–37.8)	30.1 (21.1–41)	0.9182	28.2 (17.1–37.5)	31.7 (22.9–43.8)	0.2280
% Time ΔCPPopt–PAx < −10 mmHg	12.7 (6.42–25.5)	16.5 (9.77–24.5)	0.6001	12 (5.44–25.2)	17 (10.4–25.1)	0.1347
% Time CPP > ULR–PAx	4.2 (2.46–8.94)	7.22 (3.07–16.4)	0.1471	5.68 (2.59–10.8)	5.53 (2.85–14.7)	0.5554
% Time CPP < LLR–PAx	4.98 (2.1–10)	10.3 (3.04–29.7)	0.0646	4.51 (2.1–10.1)	11.8 (3.56–29.4)	**0.0307**
Mean CPPopt–RAC	72.9 (70.1–78.4)	73.4 (69.1–79.7)	0.9348	73.2 (70–79.5)	73.2 (68.5–78.5)	0.7609
% Time ΔCPPopt–RAC > 5 mmHg	31.6 (15.4–41)	24.6 (15–33.3)	0.3693	31.4 (17.5–41.3)	23.8 (14.3–29.8)	**0.0480**
% Time ΔCPPopt–RAC > 10 mmHg	15.1 (7.46–25.4)	10.5 (6.37–16.7)	0.2307	14.5 (8.62–25.8)	9.56 (6.15–15.9)	**0.0456**
% Time ΔCPPopt–RAC < -5 mmHg	21.9 (11.2–33.7)	23.1 (14.1–35.4)	0.7775	19.2 (9.01–32.1)	27 (16.1–38.8)	0.0714
% Time ΔCPPopt–RAC < -10 mmHg	9.01 (2.52–17.9)	10.7 (5.97–18.3)	0.4729	7.94 (2.51–15.4)	13.7 (7.2–18.5)	0.0608
% Time CPP > ULR–RAC	3.3 (1.48–6.44)	4.34 (1.7–11.9)	0.3634	3.48 (1.83–7.47)	4.08 (1.64–10.5)	0.9204
% Time CPP < LLR–RAC	3.7 (1.03–6.78)	4.37 (1.99–14.8)	0.3757	3.73 (1.02–7.4)	4.43 (2.01–14.5)	0.3504

All *p* values have been adjusted using the False Discovery Rate (FDR) method

*AMP* pulse amplitude of ICP, *CPP* cerebral perfusion pressure, *CPPopt* cerebral perfusion pressure optimum, *ΔCPPopt* CPP—CPPopt, *CT* computed tomography, *GCS* Glasgow Coma Scale, *ICP* intracranial pressure, *ICU* intensive care unit, *IQR* interquartile range, *LLR* lower limit of reactivity, *MAP* mean arterial pressure, *mmHg* millimeters of mercury, *PAx* pulse amplitude index, *PRx* pressure reactivity index, *RAC* correlation (R) between slow-waves of AMP (A) and CPP (C), *SAH* subarachnoid hemorrhage, *ULR* upper limit of reactivity

Bolded *p* values are those reaching statistical significance, *p* < 0.05

The results the Mann–Whitney *U* and Chi-square testing for transition in outcome, with those who died (GOSE 1) removed (*n* = 69), are presented in Additional file 1: Appendices E–G, partitioned by transition period. All *p* values fell out of statistical significance. This is likely explained by a reduced number of patients remaining in the *Not Improved* group after removal of those who died. The Mann–Whitney *U* and Chi-square testing results for the age trichotomized data can be found in Additional file 1: Appendices H–J, partitioned by transition period.

**Fig. 1:**
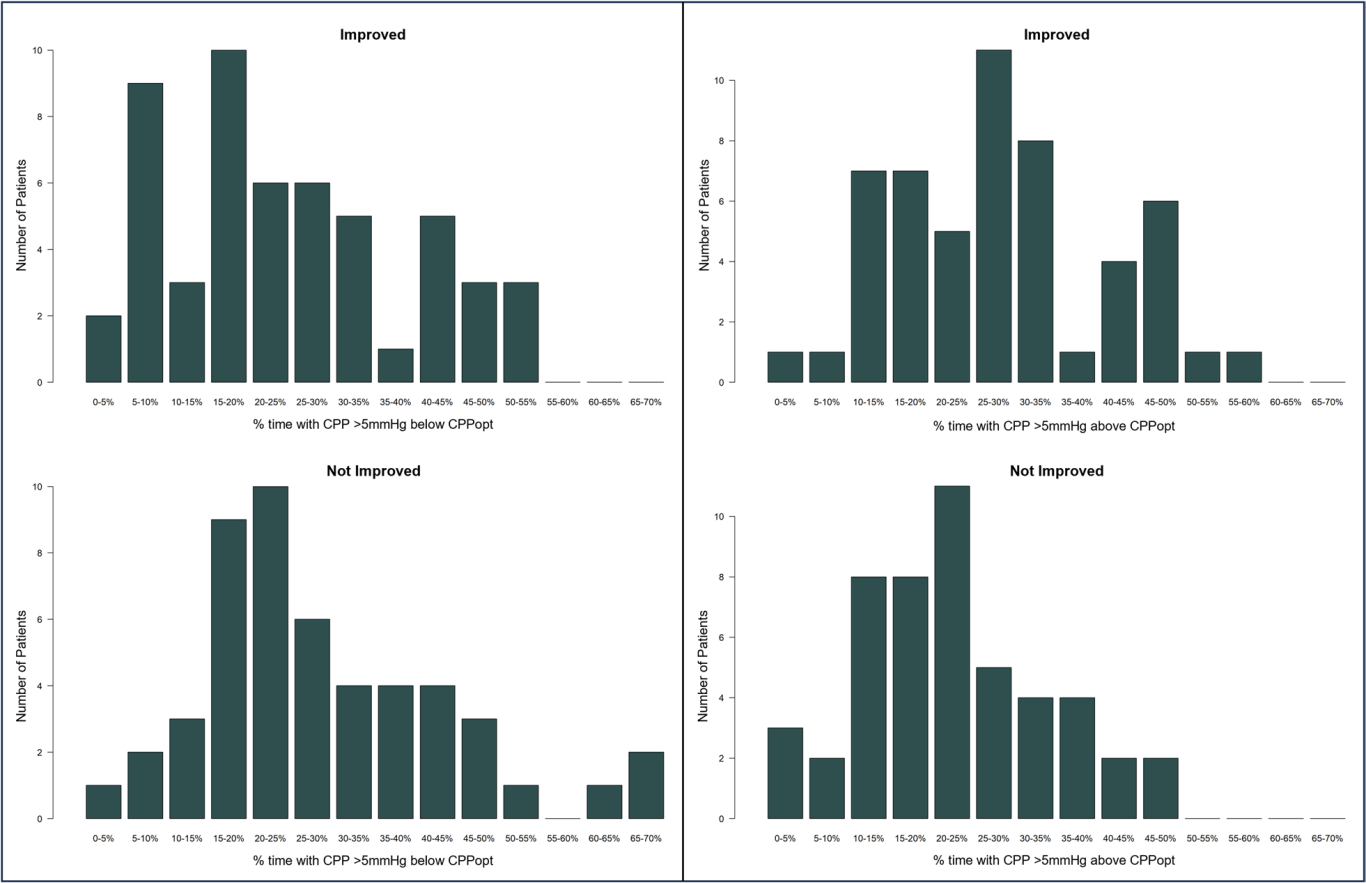
Histograms comparing Improved and Not Improved Patients for % time with ΔCPPopt above/below 5 mmHg. Left panel illustrates the distributions of improved (top) vs. Not Improved (bottom) patients for % time with ΔCPPopt below 5 mmHg. Right panel illustrates the distributions of improved (top) vs. Not Improved (bottom) patients for % time with ΔCPPopt above 5 mmHg. *CPPopt* cerebral perfusion pressure optimum

Histograms plots comparing the *Improved* and *Not Improved* cohorts for % times that patients spent with their CPP 5 mmHg below/above CPPopt are presented in Fig. 1. For CPP below CPPopt (ΔCPP < −5 mmHg), the distribution for the *Improved* group was shifted more towards lower % times compared to the *Not Improved* group, while for CPP above CPPopt (ΔCPP > 5 mmHg), the distribution for the *Improved* group was shifted more towards greater % times compared to the *Not Improved* group. Histogram plots comparing the *Improved* and *Not Improved* cohorts for % times that patients spent with their CPP 10 mmHg below/above CPPopt presented similar findings and can be found in Additional file 1: Appendix K.

### Logistic regression analysis

Univariate logistic regression results for the various CPPopt metrics are presented in Table 4. None of the metrics produced statistically significant results for the 1–3-month transition period. For the 3–6-month period, only % time with ΔCPPopt–PAx > 5 mmHg and % time with ΔCPPopt–PAx < −5 mmHg produced significant *p* values. The 1–6-month period produced significant results for the following metrics: % time with ΔCPPopt–PRx > 5 mmHg, % time with ΔCPPopt–PRx > 10 mmHg, ΔCPPopt–PAx > 5 mmHg, % time with ΔCPPopt–PAx > 10 mmHg, % time with CPP below the LLR–PAx, % time with ΔCPPopt–RAC > 5 mmHg, % time with ΔCPPopt–RAC > 10 mmHg, % time with ΔCPPopt–RAC < −5 mmHg, and % time with ΔCPPopt–RAC < −10 mmHg. Similar with the Mann–Whitney *U* and Chi-square testing results, most *p* values fell out of significance once those who died (GOSE 1) were removed, see Additional file 1: Appendix L. Results for the age trichotomized data can be found in Additional file 1: Appendix M.

**Table 4:** Univariate models of cerebrovascular reactivity measures for transition in outcome

Model	AUC (95% CI)	AIC	*p* value	Nagelkerke's R^2^
1 month → 3 months				
CPPopt–PRx	0.489 (0.369–0.602)	143.1	0.5732	0.002
% Time ΔCPPopt–PRx > 5 mmHg	0.560 (0.447–0.669)	141.6	0.2651	0.021
% Time ΔCPPopt–PRx > 10 mmHg	0.610 (0.501–0.719)	140.3	0.1218	0.039
% Time ΔCPPopt–PRx < −5 mmHg	0.516 (0.370–0.595)	142.9	0.4886	0.004
% Time ΔCPPopt–PRx < −10 mmHg	0.497 (0.393–0.614)	143.1	0.5777	0.003
% Time CPP > ULR–PRx	0.553 (0.441–0.663)	142.1	0.2904	0.016
% Time CPP < LLR–PRx	0.572 (0.457–0.684)	140.9	0.2291	0.030
CPPopt–PAx	0.492 (0.399–0.619)	143.1	0.5824	0.002
% Time ΔCPPopt–PAx > 5 mmHg	0.617 (0.507–0.723)	139.0	0.1166	0.055
% Time ΔCPPopt–PAx > 10 mmHg	0.633 (0.520–0.744)	137.5	0.1197	0.073
% Time ΔCPPopt–PAx < −5 mmHg	0.520 (0.405–0.638)	143.1	0.4855	0.002
% Time ΔCPPopt–PAx < −10 mmHg	0.552 (0.427–0.666)	143.0	0.2832	0.003
% Time CPP > ULR–PAx	0.620 (0.509–0.726)	134.7	0.1365	0.108
% Time CPP < LLR–PAx	0.649 (0.543–0.751)	131.9	0.1113	0.140
CPPopt–RAC	0.507 (0.381–0.608)	142.8	0.5257	0.005
% Time ΔCPPopt–RAC > 5 mmHg	0.584 (0.473–0.708)	141.2	0.1945	0.027
% Time ΔCPPopt–RAC > 10 mmHg	0.603 (0.490–0.716)	137.7	0.1348	0.071
% Time ΔCPPopt–RAC < −5 mmHg	0.537 (0.421–0.650)	142.9	0.3713	0.005
% Time ΔCPPopt–RAC < −10 mmHg	0.571 (0.460–0.680)	142.4	0.2131	0.011
% Time CPP > ULR–RAC	0.584 (0.467–0.688)	138.4	0.1773	0.062
% Time CPP < LLR–RAC	0.585 (0.470–0.695)	138.6	0.2181	0.060
3 months → 6 months				
CPPopt–PRx	0.597 (0.475–0.716)	125.1	0.1076	0.044
% Time ΔCPPopt–PRx > 5 mmHg	0.605 (0.482–0.720)	125.9	0.1118	0.033
% Time ΔCPPopt–PRx > 10 mmHg	0.593 (0.464–0.720)	125.6	0.1119	0.037
% Time ΔCPPopt–PRx < −5 mmHg	0.547 (0.425–0.665)	127.4	0.3198	0.013
% Time ΔCPPopt–PRx < −10 mmHg	0.521 (0.401–0.639)	128.0	0.4112	0.004
% Time CPP > ULR–PRx	0.601 (0.472–0.730)	120.7	0.1044	0.101
% Time CPP < LLR–PRx	0.511 (0.386–0.642)	128.3	0.4569	0
CPPopt–PAx	0.544 (0.411–0.668)	127.0	0.3204	0.018
% Time ΔCPPopt–PAx > 5 mmHg	0.679 (0.561–0.790)	121.0	**0.0483**	0.098
% Time ΔCPPopt–PAx > 10 mmHg	0.654 (0.523–0.778)	119.8	0.0746	0.112
% Time ΔCPPopt–PAx < −5 mmHg	0.653 (0.538–0.762)	122.6	**0.0399**	0.076
% Time ΔCPPopt–PAx < −10 mmHg	0.626 (0.502–0.742)	124.5	0.0958	0.051
% Time CPP > ULR–PAx	0.622 (0.502–0.741)	125.2	0.0795	0.042
% Time CPP < LLR–PAx	0.543 (0.425–0.663)	127.4	0.3072	0.013
CPPopt–RAC	0.534 (0.404–0.662)	127.1	0.3434	0.017
% Time ΔCPPopt–RAC > 5 mmHg	0.654 (0.537–0.768)	121.3	0.0511	0.093
% Time ΔCPPopt–RAC > 10 mmHg	0.623 (0.491–0.745)	123.2	0.0882	0.068
% Time ΔCPPopt–RAC < −5 mmHg	0.603 (0.478–0.721)	125.2	0.1067	0.042
% Time ΔCPPopt–RAC < −10 mmHg	0.558 (0.427–0.671)	127.2	0.2661	0.015
% Time CPP > ULR–RAC	0.611 (0.487–0.736)	126.2	0.1013	0.029
% Time CPP < LLR–RAC	0.471 (0.347–0.585)	128.3	0.6802	0
1 month → 6 months				
CPPopt–PRx	0.531 (0.422–0.648)	145.6	0.3472	0.014
% Time ΔCPPopt–PRx > 5 mmHg	0.632 (0.523–0.740)	140.6	**0.0281**	0.076
% Time ΔCPPopt–PRx > 10 mmHg	0.650 (0.542–0.746)	139.6	**0.0189**	0.089
% Time ΔCPPopt–PRx < −5 mmHg	0.587 (0.471–0.694)	143.9	0.1122	0.035
% Time ΔCPPopt–PRx < −10 mmHg	0.566 (0.451–0.678)	144.9	0.1757	0.024
% Time CPP > ULR–PRx	0.508 (0.381–0.600)	145.4	0.4488	0.016
% Time CPP < LLR–PRx	0.584 (0.468–0.693)	143.3	0.1161	0.043
CPPopt–PAx	0.529 (0.422–0.640)	145.6	0.3366	0.015
% Time ΔCPPopt–PAx > 5 mmHg	0.664 (0.559–0.765)	138.3	**0.0147**	0.105
% Time ΔCPPopt–PAx > 10 mmHg	0.675 (0.564–0.772)	134.2	**0.0231**	0.152
% Time ΔCPPopt–PAx < −5 mmHg	0.590 (0.481–0.705)	144.5	0.1103	0.029
% Time ΔCPPopt–PAx < −10 mmHg	0.609 (0.502–0.717)	145.0	0.0599	0.022
% Time CPP > ULR–PAx	0.551 (0.344–0.569)	145.9	0.2483	0.011
% Time CPP < LLR–PAx	0.665 (0.556–0.766)	137.0	**0.0210**	0.120
CPPopt–RAC	0.532 (0.415–0.645)	145.6	0.3537	0.015
% Time ΔCPPopt–RAC > 5 mmHg	0.643 (0.535–0.746)	138.9	**0.0214**	0.097
% Time ΔCPPopt–RAC > 10 mmHg	0.651 (0.542–0.753)	135.3	**0.0221**	0.140
% Time ΔCPPopt–RAC < −5 mmHg	0.629 (0.516–0.740)	142.1	**0.0282**	0.059
% Time ΔCPPopt–RAC < −10 mmHg	0.637 (0.528–0.743)	143.2	**0.0246**	0.044
% Time CPP > ULR–RAC	0.509 (0.397–0.620)	146.6	0.4576	0.001
% Time CPP < LLR–RAC	0.572 (0.465–0.682)	143.6	0.1566	0.039

All *p* values have been adjusted using the False Discovery Rate (FDR) method

*AIC* Akaike information criterion, *AMP* pulse amplitude of ICP, *AUC* area under the curve, *CI* confidence interval, *CPP* cerebral perfusion pressure, *CPPopt* cerebral perfusion pressure optimum, *ΔCPPopt* CPP—CPPopt, *ICP* intracranial pressure, *LLR* lower limit of reactivity, *mmHg* millimeters of mercury, *PAx* pulse amplitude index, *PRx* pressure reactivity index, *RAC* correlation (R) between slow-waves of AMP (A) and CPP (C), *ULR* upper limit of reactivity

Bolded *p* values are those reaching statistical significance, *p* < 0.05

The multivariable logistic regression results are presented in Additional file 1: Appendices N–P, separated by transition period. All four of the standardized multivariable models; IMPACT Core, Core + CT, Core + CT + % time with ICP > 20 mmHg, and Core + CT + % time with ICP > 22 mmHg, as well as the models with CPPopt metrics added reached statistical significance for all three time periods. When those who died (GOSE 1) were removed, all models remained statistically significant for the 1–3-month and 1–6-month transition periods, while some fell out of significance for the 3–6-month period. These results can be found in Additional file 1: Appendices Q–S. Results for the age trichotomized data can be found in Additional file 1: Appendices T–V.

### Additional variance in outcome transition

The differences in Nagelkerke’s pseudo-R^2^ between the models with CPPopt metrics added and the standardized multivariable models alone are presented in Table 5. These values represent the added variance in outcome transition that the CPPopt variables offer over the standardized multivariable models. Overall, most of the CPPopt metrics were able to provide additional variance in outcome for all three time periods. These results held for the most part when those who were dead (GOSE 1) were removed, see Additional file 1: Appendix W. The results from the age trichotomized data can be found in Additional file 1: Appendix X.

**Table 5:** Added variance in transition in outcome from cerebrovascular reactivity measures over IMPACT Core ± CT ± ICP > 20 or 22 mmHg

Variable	1 month → 3 months	3 month → 6 months	1 month → 6 months
Δ Nagelkerke’s R^2^			
Core			
CPPopt–PRx	0	0.035	0.009
% Time ΔCPPopt–PRx > 5 mmHg	0.014	0.027	0.063
% Time ΔCPPopt–PRx > 10 mmHg	0.037	0.034	0.089
% Time ΔCPPopt–PRx < −5 mmHg	0.003	0.010	0.029
% Time ΔCPPopt–PRx < −10 mmHg	0.001	0.003	0.019
% Time CPP > ULR–PRx	0.003	0.142	0.047
% Time CPP < LLR–PRx	0.013	0.002	0.019
CPPopt–PAx	0.009	0.025	0.032
% Time ΔCPPopt–PAx > 5 mmHg	0.018	0.065	0.047
% Time ΔCPPopt–PAx > 10 mmHg	0.035	0.082	0.091
% Time ΔCPPopt–PAx < −5 mmHg	0.007	0.043	0.001
% Time ΔCPPopt–PAx < −10 mmHg	0.009	0.021	0
% Time CPP > ULR–PAx	0.075	0.069	0
% Time CPP < LLR–PAx	0.088	0.005	0.064
CPPopt–RAC	0.016	0.023	0.034
% Time ΔCPPopt–RAC > 5 mmHg	0.004	0.064	0.041
% Time ΔCPPopt–RAC > 10 mmHg	0.033	0.045	0.078
% Time ΔCPPopt–RAC < −5 mmHg	0.005	0.016	0.008
% Time ΔCPPopt–RAC < −10 mmHg	0.003	0.001	0.001
% Time CPP > ULR–RAC	0.037	0.052	0.005
% Time CPP < LLR–RAC	0.039	0.001	0.019
Core + CT			
CPPopt–PRx	0	0.025	0.008
% Time ΔCPPopt–PRx > 5 mmHg	0.014	0.021	0.064
% Time ΔCPPopt–PRx > 10 mmHg	0.040	0.023	0.092
% Time ΔCPPopt–PRx < −5 mmHg	0.002	0.014	0.027
% Time ΔCPPopt–PRx < −10 mmHg	0.001	0.007	0.020
% Time CPP > ULR–PRx	0.003	0.130	0.049
% Time CPP < LLR–PRx	0.014	0	0.023
CPPopt–PAx	0.008	0.018	0.026
% Time ΔCPPopt–PAx > 5 mmHg	0.023	0.063	0.056
% Time ΔCPPopt–PAx > 10 mmHg	0.046	0.073	0.112
% Time ΔCPPopt–PAx < −5 mmHg	0.006	0.044	0.002
% Time ΔCPPopt–PAx < −10 mmHg	0.007	0.025	0
% Time CPP > ULR–PAx	0.077	0.066	0
% Time CPP < LLR–PAx	0.098	0.008	0.079
CPPopt–RAC	0.013	0.018	0.028
% Time ΔCPPopt–RAC > 5 mmHg	0.006	0.062	0.049
% Time ΔCPPopt–RAC > 10 mmHg	0.042	0.038	0.096
% Time ΔCPPopt–RAC < −5 mmHg	0.003	0.017	0.012
% Time ΔCPPopt–RAC < −10 mmHg	0.002	0.002	0.003
% Time CPP > ULR–RAC	0.036	0.053	0.005
% Time CPP < LLR–RAC	0.041	0.002	0.020
Core + CT + ICP > 20 mmHg			
CPPopt–PRx	0.002	0.026	0.010
% Time ΔCPPopt–PRx > 5 mmHg	0.002	0.015	0.030
% Time ΔCPPopt–PRx > 10 mmHg	0.020	0.018	0.056
% Time ΔCPPopt–PRx < −5 mmHg	0.010	0.008	0.001
% Time ΔCPPopt–PRx < −10 mmHg	0.022	0.002	0.001
% Time CPP > ULR–PRx	0.002	0.130	0.045
% Time CPP < LLR–PRx	0	0.006	0
CPPopt–PAx	0.004	0.016	0.016
% Time ΔCPPopt–PAx > 5 mmHg	0.014	0.057	0.037
% Time ΔCPPopt–PAx > 10 mmHg	0.035	0.068	0.086
% Time ΔCPPopt–PAx < −5 mmHg	0.020	0.037	0
% Time ΔCPPopt–PAx < −10 mmHg	0.024	0.020	0.004
% Time CPP > ULR–PAx	0.031	0.085	0.006
% Time CPP < LLR–PAx	0.028	0.001	0.014
CPPopt–RAC	0.007	0.015	0.015
% Time ΔCPPopt–RAC > 5 mmHg	0.002	0.056	0.033
% Time ΔCPPopt–RAC > 10 mmHg	0.036	0.036	0.081
% Time ΔCPPopt–RAC < −5 mmHg	0.018	0.013	0.002
% Time ΔCPPopt–RAC < −10 mmHg	0.016	0	0.001
% Time CPP > ULR–RAC	0.006	0.077	0.030
% Time CPP < LLR–RAC	0	0.019	0.004
Core + CT + ICP > 22 mmHg			
CPPopt–PRx	0.003	0.026	0.010
% Time ΔCPPopt–PRx > 5 mmHg	0.002	0.014	0.028
% Time ΔCPPopt–PRx > 10 mmHg	0.021	0.018	0.056
% Time ΔCPPopt–PRx < −5 mmHg	0.011	0.007	0.001
% Time ΔCPPopt–PRx < −10 mmHg	0.024	0.001	0.001
% Time CPP > ULR–PRx	0.001	0.131	0.047
% Time CPP < LLR–PRx	0.001	0.007	0
CPPopt–PAx	0.005	0.016	0.016
% Time ΔCPPopt–PAx > 5 mmHg	0.012	0.056	0.034
% Time ΔCPPopt–PAx > 10 mmHg	0.037	0.067	0.085
% Time ΔCPPopt–PAx < −5 mmHg	0.022	0.037	0
% Time ΔCPPopt–PAx < −10 mmHg	0.024	0.020	0.004
% Time CPP > ULR–PAx	0.028	0.086	0.006
% Time CPP < LLR–PAx	0.024	0.001	0.013
CPPopt–RAC	0.008	0.015	0.015
% Time ΔCPPopt–RAC > 5 mmHg	0.001	0.055	0.030
% Time ΔCPPopt–RAC > 10 mmHg	0.035	0.035	0.078
% Time ΔCPPopt–RAC < −5 mmHg	0.019	0.013	0.001
% Time ΔCPPopt–RAC < −10 mmHg	0.016	0	0.001
% Time CPP > ULR–RAC	0.005	0.078	0.031
% Time CPP < LLR–RAC	0	0.019	0.005

The Core model consisted of age, admission Glasgow Coma Scale—motor score, and admission pupillary response. CT variables consisted of admission Marshall CT grade, presence of traumatic subarachnoid hemorrhage, and presence of epidural hematoma

*AMP* pulse amplitude of ICP, *CPP* cerebral perfusion pressure, *CPPopt* cerebral perfusion pressure optimum, *ΔCPPopt* CPP—CPPopt, *CT* computed tomography, *ICP* intracranial pressure, *LLR* lower limit of reactivity, *PAx* pulse amplitude index, *PRx* pressure reactivity index, *RAC* correlation (R) between slow-waves of AMP (A) and CPP (C), *ULR* upper limit of reactivity

**Fig. 2:**
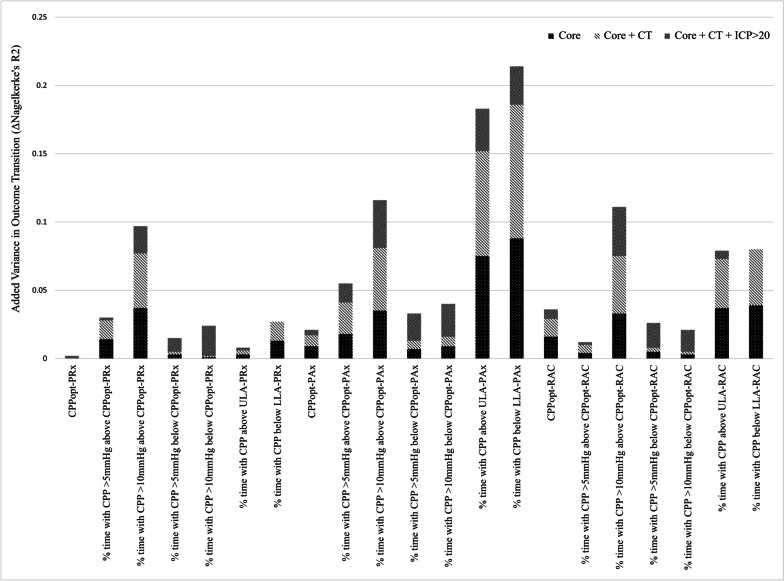
Stacked bar chart of the added variance in transition in outcome of the CPPopt variables over the Core, Core + CT, and Core + CT + ICP > 20 mmHg base models. This stacked bar chart illustrates the amount of added variance in transition in outcome between 1- and 3-month post-TBI that the various CPPopt variables provided over the Core, Core + CT, and Core + CT + ICP > 20 mmHg Base Models. The Core model consisted of age, admission Glasgow Coma Scale—motor score, and admission pupillary response. CT variables consisted of admission Marshall CT grade, presence of traumatic subarachnoid hemorrhage, and presence of epidural hematoma. *AMP* pulse amplitude of ICP, *CPP* cerebral perfusion pressure, *CPPopt* cerebral perfusion pressure optimum, *CT* computed tomography, *ICP* intracranial pressure, *LLR* lower limit of reactivity, *PAx* pulse amplitude index, *PRx* pressure reactivity index, *RAC* correlation (R) between slow-waves of AMP (A) and CPP (C), *ULR* upper limit of reactivity

To help visualize the amount of added variance in outcome transition that the various CPPopt variables provide over the base models, a stacked bar chart is presented in Fig. 2. Among the respective CPPopt metrics of each cerebrovascular reactivity index, time spent above CPPopt generally provided more additional variance in outcome transition than time spent below CPPopt. In addition, time spent below/above the LLR/ULR generally performed the best for each cerebrovascular reactivity index, except for PRx, where these parameters added little variance in outcome transition. Overall, Pax-based CPPopt metrics provided the most additional variance in outcome transition, while PRx-based metrics offered the least. Finally, as one would expect, the > 10 mmHg above/below CPPopt parameters performed better than their respective > 5 mmHg parameters.

## Discussion

Through our investigation on the association between CPPopt metrics and improvement in outcome, we uncovered several interesting findings. Findings of the Mann–Whitney *U* and Chi-square testing are discussed in Additional file 1: Appendix Y. The main findings of this study are those regarding CPPopt. Through logistic regression analysis and Mann–Whitney *U* testing, we were able to demonstrate an association between CPPopt metrics; derived using PRx, PAx, and RAC; and improvement in outcome over time. We found that increased time spent with actual CPP below CPPopt, ΔCPPopt < −5 mmHg and ΔCPPopt < −10 mmHg, or below the LLR are associated with failure to improve in outcome. This in keeping with existing literature [2, 6, 8, 11]. Furthermore, through the use of differences in Nagelkerke’s pseudo-R^2^, we were able to demonstrate that time spent below CPPopt, as well as below the LLR, offers additional prognostic utility for predicting outcome transition when controlling for intracranial hypertension and other variables with known associations with outcome. This highlights the importance of maintaining sufficient CPP, in tandem with ICP control, when managing patients who have suffered TBI.

Looking at the results of the logistic regression analyses, we can see that time spent with ΔCPPopt > 5 mmHg, ΔCPPopt > 10 mmHg, and CPP above the ULR generally showed weak associations with transition in outcome. However, looking at the Mann–Whitney *U* testing results, we see that increased time above these thresholds were surprisingly associated with improvement in outcome rather than failure to improve in outcome. The fact that these results are not showing an association with failure to improve in outcome is actually consistent with some recent literature which has found that only time spent below CPPopt was associated with outcome, while time spent above was not [2, 6]. Furthermore, it is also in line with current thoughts that avoiding time spent with CPP below CPPopt, especially the LLR, is crucial while preventing hyperemic CPP, as long as not extremely elevated, may not actually affect outcome too significantly [2, 34, 35]. In a study comparing brain tissue oxygenation with various physiologic metrics, Wettervik et al*.* showed that brain tissue oxygenation decreases significantly at lower CPP and ΔCPPopt values; however, only suffers from mild reduction at high CPP values [34]. Thiara et al*.* conducted a study that refuted the existence of an association between the elevated CPP and development of acute respiratory distress syndrome [35]. Donnelly et al*.* found that spending time with CPP below 60 mmHg or with ΔCPPopt < −10 mmHg was related to death, while spending time with CPP above 70 mmHg or with ΔCPPopt > 10 mmHg was not [2]. These results all point towards the notion that preventing cerebral hyperperfusion may not play a major role in reducing poor outcomes. However, our data suggest that spending time with CPP above CPPopt is not only not associated with failure to improve in outcome but may also be correlated with patients improving in outcome. The study by Donnelly et al*.* provides support for this possibility as they found that both time spent with CPP above 70 mmHg and time spent with ΔCPPopt > 10 mmHg exhibited inverse relationships with mortality [2]. Considering the findings discussed above, it may be conceivable that maintaining CPP above CPPopt actually helps promote recovery. One potential explanation for this is that the injured brain, which is actively attempting to repair itself, may require greater blood flow than the healthy brain due to increased energy requirements and waste production. This would be quite interesting as it would indicate that CPPopt is not an “optimal” target as currently believed, but rather a lower limit threshold. However, it is important to acknowledge that our results suffer from multiple limitations, discussed in the limitations section, which prevent us from making any strong statements.

Comparing the cerebrovascular reactivity metrics that CPPopt derivations were based on, we found that there were no significant differences in associations with outcome transition; however, AMP-based indices (CPPopt–PAx and CPPopt–RAC) generally performed better, particularly PAx according to the results of Fig. 2. This may be explained by the fact that AMP-based indices have been shown to possibly be superior to PRx for predicting long-term outcome [26, 36–38]. This somewhat contrasts a study by Zeiler et al*.* which found that CPPopt metrics based on PRx and RAC performed similarly for long-term outcome prediction, and that CPPopt metrics based on PAx were poorly correlated with outcome [6]. However, this may be explained by the limited sample size of our study. To help demonstrate that differences between the three cerebrovascular reactivity indices exist, we present linear regressions and LOESS curves illustrating their relationships with ICP in Additional file 1: Appendix Z. For a further discussion on the influences of ICP on cerebrovascular reactivity, please refer to Additional file 1: Appendix Y.

Most of our findings held true in general, although weaker, when those who died (GOSE 1) were removed (*n* = 69). This suggests that CPPopt metrics can provide additional utility in outcome transition prognostication for patients who have survived their TBI. Our findings also generally held true when patients were trichotomized based on age. However, it should be noted that, due to the small sample sizes of the individual age cohort, many models were unable to converge. Therefore, the results from this secondary analysis should be considered with caution.

Lastly, our results were generally strongest for the 1–6-month transition period, suggesting that CPPopt metrics provide more utility in predicting outcome transition over longer intervals of time. This is supported by our previous study, which found that the 1–6-month interval produced the most robust associations between cerebrovascular reactivity metrics and outcome transition [12]. This finding may be explained by the fact that longer time intervals allow for more time for the neuronal recovery mentioned above to take place, indicating that the effects that optimization of cerebrovascular reactivity during the acute phase post-TBI has on neuronal recovery continues on outside of the ICU. Despite the interesting findings uncovered here, it must be highlighted that this study is preliminary in nature and requires further validation. Particularly, additional work is needed to clarify the importance of spending time above CPPopt or the ULR on long-term outcomes.

### Limitations

In light of the noteworthy findings uncovered, it is important to acknowledge some significant limitations of this study. Firstly, a notable shortcoming is the relatively small sample size used. While some of the results achieved statistical significance, it is recommended to exercise caution when interpreting associations between cerebral physiologic metrics and outcome when sample sizes fall below 100 [39]. Though our study did have more than 100 patients, a far greater sample size would be ideal to increase statistical power and reduce the risk of misleading results. Consequently, to validate these findings, further investigation involving larger multi-center data sets is warranted. Moreover, it is essential to note that the data utilized in this study originates from a single institution, thereby limiting the generalizability of the results to the broader population. This further underscores the necessity for validation through the utilization of a multi-center database.

Another limitation of this study is that many of the associations discussed did not consistently produce statistically significant *p* values. This is likely in part due to the small sample size. This inconsistency unfortunately prevents us from making any strong statements about the associations we found. This further necessitates validation studies on the associations found. In regard to correction for multiple comparisons, some may argue that the fact that we did not use the conventional Bonferroni method may potentially be not conservative enough and put our results at risk of Type I errors. However, due to the exploratory nature of our study, we decided that maximizing statistical power, while still correcting for multiple comparisons, was more suitable. Another limitation of this study is that we only evaluated patients’ outcomes up to 6-month post-TBI, masking any further improvements that may occur over longer periods of recovery. The inclusion of longer term outcome data, such as from 12-month follow-ups, would have allowed us to better assess associations with long-term improvements.

Moving forward, there needs to be a large multi-centered validation study on the relationship between time spent with CPP above CPPopt, as well as above the ULR, and long-term outcome. In addition, further work is needed on the optimization of the CPPopt algorithm and comparing the various algorithms to determine which produces the most clinically beneficial CPPopt derivation. This will also require large multi-centered data sets. Such work is currently in progress by our lab group. In addition, the ongoing multi-center CAnadian High-Resolution TBI (CAHR-TBI) Research Collaborative, which our lab is the lead center of, is optimally positioned to tackle such important questions as the largest high-frequency cerebral physiology database in the world.

## Conclusion

In this study, we showed that time spent with actual CPP below CPPopt is associated with failure to improve in outcome, supporting the existing narrative. More interestingly, we also presented data suggesting that spending time with CPP above CPPopt is not associated with failure to improve in outcome and is possibly even related to improved outcome. However, due to limitations of this study, namely small sample size, we are unable to make any conclusive statements. Further work is needed to validate the findings uncovered here.

### Supplementary Information

**Additional file 1: Appendix A.** Mann–Whitney *U*/Chi-Square Testing of Physiologic and Demographic Data for Alive/Dead and Favorable/Unfavorable at 1 month. **Appendix B.** Mann–Whitney *U*/Chi-Square Testing of Physiologic and Demographic Data for Alive/Dead and Favorable/Unfavorable at 3 months. **Appendix C.** Mann–Whitney *U*/Chi-Square Testing of Physiologic and Demographic Data for Alive/Dead and Favorable/Unfavorable at 6 months. **Appendix D.** Mann–Whitney *U*/Chi-Square Testing of Physiologic and Demographic Data for Improved/Not Improved (3–6 months). **Appendix E.** Mann–Whitney *U*/Chi-Square Testing of Physiologic and Demographic Data for Improved/Not Improved (1–3 months) with Those Who Died (GOSE = 1) Removed. **Appendix F.** Mann–Whitney *U*/Chi-Square Testing of Physiologic and Demographic Data for Improved/Not Improved (3–6 months) with Those Who Died (GOSE = 1) Removed. **Appendix G.** Mann–Whitney *U*/Chi-Square Testing of Physiologic and Demographic Data for Improved/Not Improved (1–6 months) with Those Who Died (GOSE = 1) Removed. **Appendix H.** Mann–Whitney *U*/Chi-Square Testing of Physiologic and Demographic Data for Improved/Not Improved (1–3 months) with Patients Trichotomized by Age. **Appendix I.** Mann–Whitney *U*/Chi-Square Testing of Physiologic and Demographic Data for Improved/Not Improved (3–6 months) with Patients Trichotomized by Age. **Appendix J.** Mann–Whitney *U*/Chi-Square Testing of Physiologic and Demographic Data for Improved/Not Improved (1–6 months) with Patients Trichotomized by Age. **Appendix K.** Histograms Comparing Improved and Not Improved Patients for % time with ΔCPPopt above/below 10 mmHg. **Appendix L.** Univariate Models of Cerebrovascular Reactivity Measures for Transition in Outcome with Those Who Died (GOSE = 1) Removed. **Appendix M.** Univariate Models of Cerebrovascular Reactivity Measures for Transition in Outcome with Patients Trichotomized by Age. **Appendix N.** Multivariable Models of Cerebrovascular Reactivity Measures + IMPACT Core ± CT ± ICP > 20 or 22 mmHg for Transition in Outcome from 1 to 3 months. **Appendix O.** Multivariable Models of Cerebrovascular Reactivity Measures + IMPACT Core ± CT ± ICP > 20 or 22 mmHg for Transition in Outcome from 3 to 6 months. **Appendix P.** Multivariable Models of Cerebrovascular Reactivity Measures + IMPACT Core ± CT ± ICP > 20 or 22 mmHg for Transition in Outcome from 1 to 6 months. **Appendix Q.** Multivariable Models of Cerebrovascular Reactivity Measures + IMPACT Core ± CT ± ICP > 20 or 22 mmHg for Transition in Outcome from 1 to 3 months with Those Who Died (GOSE = 1) Removed. **Appendix R.** Multivariable Models of Cerebrovascular Reactivity Measures + IMPACT Core ± CT ± ICP > 20 or 22 mmHg for Transition in Outcome from 3 to 6 months with Those Who Died (GOSE = 1) Removed. **Appendix S.** Multivariable Models of Cerebrovascular Reactivity Measures + IMPACT Core ± CT ± ICP > 20 or 22 mmHg for Transition in Outcome from 1 to 6 months with Those Who Died (GOSE = 1) Removed. **Appendix T.** Multivariable Models of Cerebrovascular Reactivity Measures for Transition in Outcome from 1 to 3 months with Patients Trichotomized by Age. **Appendix U.** Multivariable Models of Cerebrovascular Reactivity Measures for Transition in Outcome from 3 to 6 months with Patients Trichotomized by Age. **Appendix V.** Multivariable Models of Cerebrovascular Reactivity Measures for Transition in Outcome from 1 to 6 months with Patients Trichotomized by Age. **Appendix W.** Added Variance in Transition in Outcome of Cerebrovascular Reactivity Measures Over IMPACT Core ± CT ± ICP > 20 or 22 mmHg with Those Who Died (GOSE = 1) Removed. **Appendix X.** Added Variance in Transition in Outcome of Cerebrovascular Reactivity Measures Over IMPACT Core ± CT ± ICP > 20 or 22 mmHg with Patients Trichotomized by Age. **Appendix Y.** Extended Discussion. **Appendix Z.** Graphical Illustration of the Relationships Between ICP and Cerebrovascular Reactivity

## Data Availability

The data sets analyzed in this study are currently not publicly available as the Canadian and EU jurisdictions, including the research ethics boards and regional privacy bodies under which data were collected, do not allow for data sharing.

## Abbreviations

ABP: Arterial blood pressure
AIC: Akaike information criteria
AMP: Pulse amplitude of ICP
AUC: Area under the curve
BTF: Brain Trauma Foundation
CAHR-TBI: Canadian High-Resolution TBI Research Collaborative
CPP: Cerebral perfusion pressure
CPPopt: Optimal cerebral perfusion pressure
CSV: Comma separated values
CT: Computed tomography
FDR: False discovery rate
GCS: Glasgow coma score
GOSE: Glasgow outcome scale-extended
ICM +: Intensive care monitoring “Plus”
ICP: Intracranial pressure
ICU: Intensive care unit
IMPACT: International Mission for Prognosis and Analysis of Clinical Trials
IQR: Interquartile range
LLR: Lower limit of reactivity
MAP: Mean arterial pressure
PAx: Pulse amplitude index
PRx: Pressure reactivity index
RAC: Correlation (R) between AMP (A) and CPP (C)
TBI: Traumatic brain injury
tSAH: Traumatic subarachnoid hemorrhage
ULR: Upper limit of reactivity
