# Supplementary material for: Time spent above optimal cerebral perfusion pressure is not associated with failure to improve in outcome in traumatic brain injury
Source: Intensive Care Med Exp. 2023 Dec 14;11:92. doi: 10.1186/s40635-023-00579-3 (PMC10721751; doi:10.1186/s40635-023-00579-3)
Supplement: Supplementary file 1 — Additional file 1: Appendix A. Mann–Whitney U/Chi-Square Testing of Physiologic and Demographic Data for Alive/Dead and Favorable/Unfavorable at 1 month. Appendix B. Mann–Whitney U/Chi-Square Testing of Physiologic and Demographic Data for Alive/Dead and Favorable/Unfavorable at 3 months. Appendix C. Mann–Whitney U/Chi-Square Testing of Physiologic and Demographic Data for Alive/Dead and Favorable/Unfavorable at 6 months. Appendix D. Mann–Whitney U/Chi-Square Testing of Physiologic and Demographic Data for Improved/Not Improved (3–6 months). Appendix E. Mann–Whitney U/Chi-Square Testing of Physiologic and Demographic Data for Improved/Not Improved (1–3 months) with Those Who Died (GOSE = 1) Removed. Appendix F. Mann–Whitney U/Chi-Square Testing of Physiologic and Demographic Data for Improved/Not Improved (3–6 months) with Those Who Died (GOSE = 1) Removed. Appendix G. Mann–Whitney U/Chi-Square Testing of Physiologic and Demographic Data for Improved/Not Improved (1–6 months) with Those Who Died (GOSE = 1) Removed. Appendix H. Mann–Whitney U/Chi-Square Testing of Physiologic and Demographic Data for Improved/Not Improved (1–3 months) with Patients Trichotomized by Age. Appendix I. Mann–Whitney U/Chi-Square Testing of Physiologic and Demographic Data for Improved/Not Improved (3–6 months) with Patients Trichotomized by Age. Appendix J. Mann–Whitney U/Chi-Square Testing of Physiologic and Demographic Data for Improved/Not Improved (1–6 months) with Patients Trichotomized by Age. Appendix K. Histograms Comparing Improved and Not Improved Patients for % time with ΔCPPopt above/below 10 mmHg. Appendix L. Univariate Models of Cerebrovascular Reactivity Measures for Transition in Outcome with Those Who Died (GOSE = 1) Removed. Appendix M. Univariate Models of Cerebrovascular Reactivity Measures for Transition in Outcome with Patients Trichotomized by Age. Appendix N. Multivariable Models of Cerebrovascular Reactivity Measures + IMPACT Core ± CT ± ICP > 20 or 22 mmHg for Tr [file 40635_2023_579_MOESM1_ESM.docx]

Appendix A. Mann-Whitney U/Chi-Square Testing of Physiologic and Demographic Data for Alive/Dead and Favorable/Unfavorable at 1-Month

| **Variable** | **Alive/Dead Outcome Groups** | | | **Favorable/Unfavorable Outcome Groups** | | |
| --- | --- | --- | --- | --- | --- | --- |
|  | **Alive**  **Median (IQR)** | **Dead**  **Median (IQR)** | **p-value** | **Dead**  **Median (IQR)** | **Alive**  **Median (IQR)** | **p-value** |
| Age (years) | 40 (26 - 50) | 60.5 (34.5 - 67) | **0.0061** | 42 (27.5 - 50) | 42 (27 - 63) | 0.2454 |
| Sex (% Male) | 82.60% | 76.50% | 0.7375 | 86% | 75.50% | 0.5941 |
| Admission GCS Total | 7 (5 - 8) | 5 (3.25 - 8) | 0.0988 | 7 (6 - 8.75) | 6 (4 - 8) | 0.0599 |
| Admission GCS Motor | 5 (3 - 5) | 2.5 (1 - 4) | **0.0204** | 5 (4 - 5) | 4 (1 - 5) | **0.0379** |
| Admission GCS Eyes | 1 (1 - 2) | 1 (1 - 2) | 0.8605 | 1 (1 - 2) | 1 (1 - 2) | 0.6392 |
| Admission GCS Verbal | 1 (1 - 2) | 1 (1 - 2) | 0.9617 | 1 (1 - 2) | 1 (1 - 1) | 0.1458 |
| Admission Pupil Response  (% Bilaterally Reactive) | 62.30% | 55.90% | 0.8496 | 68% | 52.80% | 0.5605 |
| Marshall CT Grade | 4 (3 - 5) | 5 (4 - 5) | 0.0708 | 4 (3 - 5) | 5 (4 - 5) | 0.4932 |
| Rotterdam CT Grade | 4 (3 - 5) | 5 (4 - 6) | 0.0942 | 4 (3 - 5) | 5 (4 - 6) | 0.1804 |
| Helsinki CT Score | 5 (4 - 9) | 8 (6 - 9.75) | **0.0325** | 5 (3.25 - 8) | 8 (5 - 9) | **0.0390** |
| Stockholm CT Score | 3.1 (2.4 - 3.5) | 3.5 (2.8 - 4) | 0.1655 | 3 (2.2 - 3.45) | 3.5 (3 - 4) | **0.0087** |
| Number with Hypoxia Episode | 34.80% | 35.30% | 1.0000 | 32% | 37.70% | 0.7832 |
| Number with Hypotension Episode | 13% | 5.90% | 0.5489 | 8% | 13.20% | 0.7499 |
| Number with Traumatic SAH | 95.70% | 97.10% | 1.0000 | 96% | 96.20% | 1.0000 |
| Number with Epidural Hematoma | 14.50% | 2.90% | 0.2343 | 18% | 3.80% | 0.1557 |
| Admission Hemoglobin | 135 (115 - 148) | 128 (113 - 140) | 0.2526 | 137 (114 - 146) | 129 (113 - 143) | 0.5495 |
| Admission Serum Glucose | 8 (6.9 - 10) | 8.65 (7.12 - 11.3) | 0.2793 | 7.85 (6.93 - 10) | 8.2 (7.1 - 11.1) | 0.6056 |
| Length of Hospital Stay | 35 (20.8 - 60.5) | 8 (4 - 12.2) | **<0.0001** | 30 (15.5 - 41.8) | 13.5 (6.25 - 55.5) | 0.2464 |
| Length of ICU Stay | 9 (4 - 16) | 6.5 (4 - 11) | 0.2558 | 7 (3 - 11) | 11 (5 - 18.5) | 0.0667 |
| Mean MAP (mmHg) | 84 (80.2 - 87.2) | 82.7 (78.6 - 88.2) | 0.5700 | 83.4 (79.5 - 88.1) | 83.7 (79 - 88) | 1.0000 |
| Mean ICP (mmHg) | 8.55 (4.75 - 12.1) | 9.76 (6.82 - 14.5) | 0.2475 | 7.71 (3.43 - 10.7) | 10.5 (7.92 - 14) | **0.0479** |
| % Time ICP > 20 mmHg | 1.06 (0 - 5.03) | 1.65 (0.142 - 14.6) | 0.3059 | 0.335 (0 - 3.08) | 1.75 (0.208 - 9.08) | 0.0636 |
| % Time ICP > 22 mmHg | 0.571 (0 - 2.57) | 0.874 (0.0908 - 11.1) | 0.2247 | 0.142 (0 - 1.66) | 0.985 (0.106 - 4.22) | 0.0717 |
| Mean CPP (mmHg) | 74.4 (71.1 - 80.6) | 72.6 (67.7 - 75.2) | 0.0634 | 75.5 (71.8 - 81.2) | 72.7 (69.2 - 76) | 0.1013 |
| % Time CPP < 60 mmHg | 4.04 (1.08 - 7.7) | 7.05 (1.39 - 19) | 0.1569 | 3.59 (0.526 - 7.64) | 4.5 (1.76 - 13) | 0.2118 |
| % Time CPP > 70 mmHg | 64.4 (52.3 - 83.4) | 52.3 (30.5 - 71.5) | 0.0550 | 66.7 (52.2 - 84) | 58.8 (39.6 - 71) | 0.1431 |
| Mean PRx | 0.136 (0.0371 - 0.253) | 0.189 (0.0984 - 0.409) | 0.0990 | 0.166 (0.0533 - 0.276) | 0.13 (0.0371 - 0.276) | 0.6363 |
| % Time PRx > 0 | 63.3 (52.4 - 80.8) | 68.7 (61.5 - 87.2) | 0.1216 | 70.2 (54.9 - 82.9) | 64 (53.1 - 80.2) | 0.6488 |
| % Time PRx > 0.25 | 38.6 (27.3 - 54.9) | 45.4 (35.3 - 70.3) | 0.1246 | 41.4 (29.2 - 57.2) | 36.9 (26.3 - 56.2) | 0.6255 |
| % Time PRx > 0.35 | 27.5 (19.1 - 40.7) | 34.6 (25.4 - 58.9) | 0.1191 | 30.8 (22.8 - 44.5) | 28.2 (18.9 - 45.1) | 0.6374 |
| Mean PAx | -0.041 (-0.131 - 0.0665) | 0.0601 (-0.0798 - 0.305) | **0.0334** | -0.00541 (-0.119 - 0.0938) | 0.0175 (-0.0988 - 0.181) | 0.7336 |
| % Time PAx > 0 | 45.2 (35.3 - 59.2) | 59.8 (40.5 - 80.5) | **0.0407** | 49.9 (35.5 - 64.3) | 50.6 (35.3 - 68.8) | 0.7256 |
| % Time PAx > 0.25 | 21.8 (12.5 - 31.7) | 33.1 (18.7 - 60.1) | **0.0314** | 23.4 (13.3 - 35.8) | 25.4 (13.5 - 46.2) | 0.6228 |
| Mean RAC | -0.274 (-0.401 - -0.132) | -0.122 (-0.283 - 0.0978) | **0.0089** | -0.227 (-0.381 - -0.0994) | -0.193 (-0.379 - -0.00444) | 0.6476 |
| % Time RAC > -0.10 | 30.8 (17.2 - 47.1) | 47.6 (29.7 - 68) | **0.0128** | 33.6 (18.5 - 48.9) | 35.5 (21.7 - 61.2) | 0.6327 |
| % Time RAC > -0.05 | 25.6 (14.6 - 40.4) | 41.2 (26 - 65) | **0.0147** | 29 (15.4 - 43.2) | 30.4 (19 - 54.8) | 0.6315 |
| CPPopt-PRx | 75.1 (69.5 - 81.9) | 73.1 (69.2 - 78.9) | 0.4780 | 75.3 (68.8 - 83.6) | 73 (70 - 78.4) | 0.6143 |
| % Time ΔCPPopt-PRx > 5 mmHg | 26.2 (17.2 - 34.2) | 21.7 (14.5 - 29.1) | 0.1738 | 23.9 (15.5 - 34) | 24.6 (17 - 30.9) | 0.8382 |
| % Time ΔCPPopt-PRx > 10 mmHg | 12 (7.17 - 19.9) | 10.1 (5.41 - 15.7) | 0.1694 | 10.2 (6.66 - 19.8) | 10.8 (7.06 - 17.4) | 0.9471 |
| % Time ΔCPPopt-PRx < -5 mmHg | 24.2 (15.7 - 33.2) | 25.5 (18 - 37.4) | 0.5472 | 24.2 (15.1 - 35.4) | 24.1 (17.1 - 35.4) | 0.7289 |
| % Time ΔCPPopt-PRx < -10 mmHg | 9.76 (4.61 - 19.4) | 10.4 (5.2 - 18.7) | 0.8349 | 11.1 (4.61 - 19.8) | 9.82 (5.05 - 18.5) | 1.0000 |
| % Time CPP > ULR-PRx | 13.3 (6.48 - 24) | 15.4 (8.55 - 20.7) | 0.9416 | 15 (7.05 - 24.9) | 13.6 (7.51 - 19) | 0.5985 |
| % Time CPP < LLR-PRx | 11.2 (4.58 - 26.4) | 14.4 (7.08 - 25.9) | 0.3556 | 14.6 (4.67 - 30.4) | 10.7 (6.07 - 20.8) | 0.9324 |
| CPPopt-PAx | 75 (71.8 - 82.8) | 75.8 (71.3 - 79.6) | 0.8265 | 76.2 (71.3 - 84.9) | 74.7 (71.8 - 79.4) | 0.6424 |
| % Time ΔCPPopt-PAx > 5 mmHg | 25.1 (15.6 - 34.7) | 17.9 (11.9 - 25.1) | **0.0349** | 23.3 (14.9 - 32) | 19.8 (13.1 - 30.5) | 0.6417 |
| % Time ΔCPPopt-PAx > 10 mmHg | 11.5 (6.3 - 20.9) | 6.59 (4.2 - 11.7) | **0.0293** | 10.9 (4.92 - 20.1) | 8.11 (4.62 - 15.9) | 0.5877 |
| % Time ΔCPPopt-PAx < -5 mmHg | 28.3 (17.1 - 37.4) | 36.3 (23.8 - 44.4) | 0.1021 | 26.2 (16.5 - 35.4) | 34.7 (21.7 - 44.2) | 0.1570 |
| % Time ΔCPPopt-PAx < -10 mmHg | 13.1 (5.49 - 23.7) | 18.4 (11.4 - 26.1) | 0.1182 | 12.7 (5.13 - 21.5) | 17 (9.89 - 26.3) | 0.1506 |
| % Time CPP > ULR-PAx | 5.14 (2.41 - 9.59) | 8.87 (3.42 - 16.2) | 0.1631 | 4.61 (2.65 - 11.2) | 7.2 (2.77 - 14.7) | 0.5755 |
| % Time CPP < LLR-PAx | 4.73 (2.12 - 10.5) | 14.2 (4.36 - 32.1) | **0.0177** | 4.45 (2.12 - 10.9) | 10.2 (3.68 - 18.4) | 0.1492 |
| CPPopt-RAC | 73.2 (69.6 - 79.5) | 73.1 (68.7 - 77.7) | 0.7339 | 75 (69.4 - 82.4) | 72.7 (69.9 - 77.5) | 0.5871 |
| % Time ΔCPPopt-RAC > 5 mmHg | 27.6 (17.1 - 40) | 23.3 (14.2 - 29) | 0.0597 | 26 (15.5 - 39.1) | 25.3 (14.8 - 33.9) | 0.7422 |
| % Time ΔCPPopt-RAC > 10 mmHg | 12.2 (7.67 - 24.4) | 9.75 (5.43 - 15.1) | 0.0930 | 11.2 (6.21 - 23.6) | 11.5 (6.47 - 18) | 1.0000 |
| % Time ΔCPPopt-RAC < -5 mmHg | 19.2 (9.87 - 33) | 26.8 (19.5 - 38.8) | 0.0643 | 18.6 (9.89 - 31.9) | 25.8 (17.8 - 37.4) | 0.1501 |
| % Time ΔCPPopt-RAC < -10 mmHg | 8.47 (2.54 - 18.2) | 13.6 (8.12 - 18.4) | 0.0824 | 7.69 (2.52 - 18) | 13.1 (6.74 - 18.4) | 0.1938 |
| % Time CPP > ULR-RAC | 3.45 (1.56 - 7.25) | 4.58 (1.71 - 12.8) | 0.3751 | 3.5 (1.25 - 7.72) | 4.13 (1.83 - 8.79) | 0.6443 |
| % Time CPP < LLR-RAC | 3.25 (0.971 - 6.98) | 6.36 (2.33 - 17.6) | 0.0669 | 3.06 (0.983 - 5.34) | 5.25 (1.99 - 13.6) | 0.1953 |

*All p-values have been adjusted using the False Discovery Rate (FDR) method. Bolded p-values are those reaching statistical significance, p < 0.05.*

*AMP = pulse amplitude of ICP, CPP = cerebral perfusion pressure, CPPopt = cerebral perfusion pressure optimum, ΔCPPopt = CPP - CPPopt, CT = computed tomography, GCS = Glasgow Coma Scale, ICP = intracranial pressure, ICU = intensive care unit, IQR = interquartile range, LLR = lower limit of reactivity, MAP = mean arterial pressure, mmHg = millimeters of mercury, PAx = pulse amplitude index, PRx = pressure reactivity index, RAC = correlation (R) between slow-waves of AMP (A) and CPP (C), SAH = subarachnoid hemorrhage, ULR = upper limit of reactivity.*

Appendix B. Mann-Whitney U/Chi-Square Testing of Physiologic and Demographic Data for Alive/Dead and Favorable/Unfavorable at 3-Months

| **Variable** | **Alive/Dead Outcome Groups** | | | **Favorable/Unfavorable Outcome Groups** | | |
| --- | --- | --- | --- | --- | --- | --- |
|  | **Alive**  **Median (IQR)** | **Dead**  **Median (IQR)** | **p-value** | **Dead**  **Median (IQR)** | **Alive**  **Median (IQR)** | **p-value** |
| Age (years) | 39 (25.5 - 50) | 58.5 (35.5 - 67) | **0.0032** | 39 (27 - 50) | 55.5 (29.5 - 66.8) | 0.0664 |
| Sex (% Male) | 82.10% | 77.80% | 0.9006 | 83.60% | 76.20% | 0.6718 |
| Admission GCS Total | 7 (5 - 8) | 5.5 (3.75 - 8) | 0.0966 | 7 (6 - 8) | 5.5 (3.25 - 7.75) | 0.0752 |
| Admission GCS Motor | 5 (3 - 5) | 3 (1 - 4) | **0.0208** | 5 (3 - 5) | 3 (1 - 4) | **0.0302** |
| Admission GCS Eyes | 1 (1 - 2) | 1 (1 - 2) | 1.0000 | 1 (1 - 2) | 1 (1 - 2) | 0.7608 |
| Admission GCS Verbal | 1 (1 - 2) | 1 (1 - 2) | 0.9464 | 1 (1 - 2) | 1 (1 - 1) | 0.6080 |
| Admission Pupil Response  (% Bilaterally Reactive) | 62.70% | 55.60% | 0.9073 | 63.90% | 54.80% | 0.7653 |
| Marshall CT Grade | 4 (3 - 5) | 5 (4 - 5) | 0.1031 | 4 (3 - 5) | 5 (4 - 5) | 0.2800 |
| Rotterdam CT Grade | 4 (3 - 5) | 5 (4 - 6) | 0.1036 | 4 (3 - 5) | 4.5 (4 - 6) | 0.2987 |
| Helsinki CT Score | 5 (3.5 - 9) | 8 (6 - 9.25) | **0.0383** | 5 (3 - 9) | 7 (5 - 9) | 0.1203 |
| Stockholm CT Score | 3.1 (2.45 - 3.6) | 3.45 (2.72 - 4) | 0.3075 | 3 (2.3 - 3.5) | 3.5 (2.82 - 4) | 0.1376 |
| Number with Hypoxia Episode | 34.30% | 36.10% | 1.0000 | 32.80% | 38.10% | 0.8159 |
| Number with Hypotension Episode | 13.40% | 5.60% | 0.4767 | 11.50% | 9.50% | 1.0000 |
| Number with Traumatic SAH | 95.50% | 97.20% | 1.0000 | 95.10% | 97.60% | 0.9079 |
| Number with Epidural Hematoma | 14.90% | 2.80% | 0.2223 | 14.80% | 4.80% | 0.3872 |
| Admission Hemoglobin | 135 (114 - 148) | 128 (113 - 140) | 0.3374 | 133 (114 - 146) | 130 (114 - 143) | 0.7529 |
| Admission Serum Glucose | 7.9 (6.7 - 9.8) | 9.4 (7.18 - 11.1) | 0.2206 | 8.1 (6.9 - 10.2) | 8.1 (7.1 - 10.9) | 0.6518 |
| Length of Hospital Stay | 35 (20.2 - 61.5) | 8 (4 - 13.8) | **<0.0001** | 32 (19.2 - 54.5) | 9.5 (4.75 - 22) | **0.0010** |
| Length of ICU Stay | 9 (4 - 15.5) | 6.5 (4 - 11) | 0.3501 | 8 (4 - 15) | 7 (4 - 15.5) | 0.8613 |
| Mean MAP (mmHg) | 83.7 (80.2 - 86.9) | 83.9 (78.6 - 88.7) | 0.7861 | 84 (80.2 - 88.3) | 83.6 (78.7 - 87.9) | 0.6687 |
| Mean ICP (mmHg) | 8.78 (5.05 - 12.1) | 9.1 (6.56 - 13.9) | 0.4750 | 8.33 (4.75 - 12) | 9.1 (6.82 - 14.4) | 0.3969 |
| % Time ICP > 20 mmHg | 1.12 (0.00747 - 5.14) | 1.41 (0.117 - 10.9) | 0.4812 | 1.06 (0 - 5.03) | 1.65 (0.142 - 8.91) | 0.3933 |
| % Time ICP > 22 mmHg | 0.605 (0 - 2.58) | 0.785 (0.0692 - 8.12) | 0.3525 | 0.571 (0 - 2.59) | 0.85 (0.0877 - 4.17) | 0.3929 |
| Mean CPP (mmHg) | 74.4 (70.9 - 80.2) | 73 (67.7 - 77.4) | 0.1494 | 74.6 (71.1 - 80.6) | 72.9 (68.5 - 75.2) | 0.1259 |
| % Time CPP < 60 mmHg | 4.24 (1.26 - 8.07) | 4.91 (1.27 - 17.9) | 0.3034 | 4.24 (0.636 - 8.98) | 4.16 (1.53 - 14.6) | 0.4507 |
| % Time CPP > 70 mmHg | 64.1 (52.2 - 79.4) | 56 (31.3 - 75.4) | 0.1077 | 65.9 (52.3 - 83.5) | 57.9 (36.5 - 72) | 0.1178 |
| Mean PRx | 0.136 (0.0359 - 0.258) | 0.189 (0.105 - 0.386) | 0.1010 | 0.146 (0.0376 - 0.27) | 0.149 (0.0571 - 0.283) | 0.6237 |
| % Time PRx > 0 | 62.5 (52.2 - 81.4) | 68.7 (62.8 - 86.8) | 0.1017 | 64.3 (52.4 - 82.3) | 66.1 (56.9 - 81) | 0.6600 |
| % Time PRx > 0.25 | 37.5 (27.1 - 55.5) | 45.4 (35.8 - 70) | 0.1013 | 39 (28.2 - 56.8) | 41.9 (27.9 - 57.5) | 0.6723 |
| % Time PRx > 0.35 | 27.4 (19.1 - 40.7) | 34.6 (25.6 - 58.8) | 0.1041 | 28.9 (19.9 - 42.8) | 31.7 (20.5 - 45.4) | 0.6783 |
| Mean PAx | -0.0434 (-0.136 - 0.0728) | 0.0499 (-0.0576 - 0.279) | **0.0400** | -0.00844 (-0.127 - 0.0791) | 0.0341 (-0.0916 - 0.248) | 0.2175 |
| % Time PAx > 0 | 45.2 (34.8 - 60.3) | 58 (42.8 - 77.6) | **0.0499** | 47.9 (35.3 - 61.5) | 52.9 (39.2 - 76) | 0.2617 |
| % Time PAx > 0.25 | 21.3 (12.2 - 32.7) | 31.7 (18.8 - 57.1) | **0.0469** | 22.9 (12.7 - 33.7) | 26.6 (15.2 - 53.4) | 0.2195 |
| Mean RAC | -0.274 (-0.404 - -0.133) | -0.122 (-0.282 - 0.0731) | **0.0068** | -0.274 (-0.401 - -0.132) | -0.123 (-0.314 - 0.0468) | 0.0775 |
| % Time RAC > -0.10 | 30.8 (16.9 - 46.7) | 47.6 (30.3 - 65.2) | **0.0093** | 31.1 (17.2 - 47.1) | 44.8 (27.3 - 63.3) | 0.0755 |
| % Time RAC > -0.05 | 25.6 (14.1 - 40.4) | 41.2 (25.7 - 61.1) | **0.0105** | 26.3 (14.6 - 40.4) | 39.4 (23.2 - 58.6) | 0.0716 |
| CPPopt-PRx | 75 (69.4 - 81.3) | 73.5 (69.3 - 79) | 0.7725 | 75.3 (69.2 - 83.5) | 73.1 (69.5 - 78.8) | 0.5596 |
| % Time ΔCPPopt-PRx > 5 mmHg | 25.7 (17.1 - 33.8) | 23.1 (14.8 - 31.2) | 0.3668 | 25.2 (17 - 34.1) | 24.2 (15.2 - 29.9) | 0.6119 |
| % Time ΔCPPopt-PRx > 10 mmHg | 11 (7.06 - 19.5) | 10.2 (5.45 - 16.8) | 0.4004 | 11 (6.96 - 19.9) | 10.2 (5.97 - 16) | 0.4413 |
| % Time ΔCPPopt-PRx < -5 mmHg | 24.2 (15.4 - 34.7) | 24.3 (18.7 - 36.1) | 0.6029 | 24.2 (16 - 36.2) | 24.3 (16.4 - 35.3) | 0.8682 |
| % Time ΔCPPopt-PRx < -10 mmHg | 9.76 (4.77 - 19.6) | 10.4 (4.91 - 18.6) | 0.8919 | 10.3 (4.94 - 20.7) | 9.92 (4.72 - 17.8) | 0.8705 |
| % Time CPP > ULR-PRx | 12.9 (6.26 - 22.4) | 16 (8.79 - 22.5) | 0.6605 | 14.5 (6.94 - 24) | 13.8 (7.74 - 20.7) | 0.7373 |
| % Time CPP < LLR-PRx | 11.2 (4.76 - 26.8) | 14.4 (6.23 - 25.2) | 0.4435 | 12.1 (4.93 - 28.3) | 11 (6.06 - 22.8) | 0.8744 |
| CPPopt-PAx | 74.9 (71.8 - 82.6) | 77.1 (71.4 - 81.3) | 0.9372 | 75.8 (71.7 - 82.8) | 74.8 (71.6 - 79.6) | 0.7991 |
| % Time ΔCPPopt-PAx > 5 mmHg | 25.1 (15.8 - 34.9) | 17.9 (12 - 26.1) | **0.0375** | 26.2 (15.6 - 34.7) | 18.4 (12.3 - 26.3) | 0.1166 |
| % Time ΔCPPopt-PAx > 10 mmHg | 11.5 (6.29 - 21) | 6.63 (4.2 - 12) | **0.0339** | 12.6 (5.76 - 22.1) | 7.03 (4.47 - 11.7) | 0.0656 |
| % Time ΔCPPopt-PAx < -5 mmHg | 28.2 (17.1 - 37) | 36.3 (24.6 - 44.8) | 0.0703 | 26.9 (17.1 - 37.4) | 34.9 (23.8 - 44.4) | 0.1821 |
| % Time ΔCPPopt-PAx < -10 mmHg | 12.9 (5.46 - 23.5) | 18.4 (11.5 - 27.1) | 0.0771 | 13.1 (5.49 - 23.7) | 17 (9.93 - 26.1) | 0.2703 |
| % Time CPP > ULR-PAx | 5.14 (2.41 - 10.2) | 7.53 (3.29 - 15.4) | 0.2298 | 5.42 (2.59 - 10.8) | 6.2 (2.84 - 15) | 0.6470 |
| % Time CPP < LLR-PAx | 4.51 (2.12 - 10.2) | 14.1 (5.04 - 31.4) | **0.0122** | 4.73 (2.12 - 10.2) | 12.3 (3.78 - 29.4) | 0.0571 |
| CPPopt-RAC | 73.2 (69.5 - 78.9) | 73.9 (69.5 - 79.8) | 1.0000 | 73.4 (69.6 - 79.5) | 72.9 (68.8 - 77.7) | 0.6760 |
| % Time ΔCPPopt-RAC > 5 mmHg | 27.6 (17.1 - 40.1) | 23.3 (14.4 - 29) | 0.0640 | 26.8 (17.1 - 39.9) | 24.4 (14.5 - 29.8) | 0.2708 |
| % Time ΔCPPopt-RAC > 10 mmHg | 12.2 (7.85 - 24.7) | 9.75 (5.91 - 15.4) | 0.1005 | 12.2 (7.67 - 24.2) | 10.5 (6.21 - 15.9) | 0.3091 |
| % Time ΔCPPopt-RAC < -5 mmHg | 19.2 (9.44 - 32.6) | 27.7 (19.7 - 39.8) | **0.0422** | 19.2 (9.95 - 33) | 26.2 (18.9 - 38.8) | 0.1845 |
| % Time ΔCPPopt-RAC < -10 mmHg | 7.94 (2.53 - 17.1) | 13.9 (8.12 - 18.4) | **0.0361** | 8.47 (2.59 - 17.3) | 13.4 (7.26 - 18.4) | 0.2291 |
| % Time CPP > ULR-RAC | 3.45 (1.47 - 7.23) | 4.58 (1.73 - 12.3) | 0.3420 | 3.68 (1.56 - 7.25) | 3.69 (1.71 - 11.7) | 0.6688 |
| % Time CPP < LLR-RAC | 2.87 (0.965 - 6.97) | 6.36 (2.67 - 16.5) | **0.0388** | 3.25 (0.971 - 6.96) | 5.79 (2.03 - 15) | 0.1391 |

*All p-values have been adjusted using the False Discovery Rate (FDR) method. Bolded p-values are those reaching statistical significance, p < 0.05.*

*AMP = pulse amplitude of ICP, CPP = cerebral perfusion pressure, CPPopt = cerebral perfusion pressure optimum, ΔCPPopt = CPP - CPPopt, CT = computed tomography, GCS = Glasgow Coma Scale, ICP = intracranial pressure, ICU = intensive care unit, IQR = interquartile range, LLR = lower limit of reactivity, MAP = mean arterial pressure, mmHg = millimeters of mercury, PAx = pulse amplitude index, PRx = pressure reactivity index, RAC = correlation (R) between slow-waves of AMP (A) and CPP (C), SAH = subarachnoid hemorrhage, ULR = upper limit of reactivity.*

Appendix C. Mann-Whitney U/Chi-Square Testing of Physiologic and Demographic Data for Alive/Dead and Favorable/Unfavorable at 6-Months

| **Variable** | **Alive/Dead Outcome Groups** | | | **Favorable/Unfavorable Outcome Groups** | | |
| --- | --- | --- | --- | --- | --- | --- |
|  | **Alive**  **Median (IQR)** | **Dead**  **Median (IQR)** | **p-value** | **Dead**  **Median (IQR)** | **Alive**  **Median (IQR)** | **p-value** |
| Age (years) | 39 (25.5 - 50) | 58.5 (35.5 - 67) | **0.0032** | 39 (26.5 - 50) | 57 (32.5 - 67) | **0.0253** |
| Sex (% Male) | 82.10% | 77.80% | 0.9006 | 84.10% | 75% | 0.5227 |
| Admission GCS Total | 7 (5 - 8) | 5.5 (3.75 - 8) | 0.0966 | 7 (5 - 8) | 6 (4 - 8) | 0.1031 |
| Admission GCS Motor | 5 (3 - 5) | 3 (1 - 4) | **0.0208** | 5 (3 - 5) | 3.5 (1 - 4.25) | **0.0474** |
| Admission GCS Eyes | 1 (1 - 2) | 1 (1 - 2) | 1.0000 | 1 (1 - 2) | 1 (1 - 2) | 0.9394 |
| Admission GCS Verbal | 1 (1 - 2) | 1 (1 - 2) | 0.9464 | 1 (1 - 2) | 1 (1 - 1.25) | 0.6518 |
| Admission Pupil Response  (% Bilaterally Reactive) | 62.70% | 55.60% | 0.9073 | 65.10% | 52.50% | 0.5635 |
| Marshall CT Grade | 4 (3 - 5) | 5 (4 - 5) | 0.1031 | 4 (3 - 5) | 5 (4 - 5) | 0.1007 |
| Rotterdam CT Grade | 4 (3 - 5) | 5 (4 - 6) | 0.1036 | 4 (3 - 5) | 5 (4 - 6) | 0.1414 |
| Helsinki CT Score | 5 (3.5 - 9) | 8 (6 - 9.25) | **0.0383** | 5 (3 - 9) | 7.5 (5 - 9.25) | 0.0502 |
| Stockholm CT Score | 3.1 (2.45 - 3.6) | 3.45 (2.72 - 4) | 0.3075 | 3 (2.35 - 3.5) | 3.5 (2.8 - 4) | 0.1097 |
| Number with Hypoxia Episode | 34.30% | 36.10% | 1.0000 | 33.30% | 37.50% | 0.9232 |
| Number with Hypotension Episode | 13.40% | 5.60% | 0.4767 | 12.70% | 7.50% | 0.7279 |
| Number with Traumatic SAH | 95.50% | 97.20% | 1.0000 | 95.20% | 97.50% | 0.9721 |
| Number with Epidural Hematoma | 14.90% | 2.80% | 0.2223 | 14.30% | 5% | 0.4249 |
| Admission Hemoglobin | 135 (114 - 148) | 128 (113 - 140) | 0.3374 | 135 (114 - 148) | 129 (113 - 142) | 0.4376 |
| Admission Serum Glucose | 7.9 (6.7 - 9.8) | 9.4 (7.18 - 11.1) | 0.2206 | 8.1 (6.9 - 10.1) | 8.1 (7.1 - 11) | 0.5016 |
| Length of Hospital Stay | 35 (20.2 - 61.5) | 8 (4 - 13.8) | **<0.0001** | 33 (20 - 55.5) | 9 (4.25 - 18) | **0.0002** |
| Length of ICU Stay | 9 (4 - 15.5) | 6.5 (4 - 11) | 0.3501 | 8 (4 - 15) | 7 (4 - 13.8) | 0.8903 |
| Mean MAP (mmHg) | 83.7 (80.2 - 86.9) | 83.9 (78.6 - 88.7) | 0.7861 | 84 (80.2 - 87.8) | 82.7 (78.6 - 88.1) | 0.5620 |
| Mean ICP (mmHg) | 8.78 (5.05 - 12.1) | 9.1 (6.56 - 13.9) | 0.4750 | 8.55 (5.05 - 12) | 9.1 (6.56 - 13.9) | 0.4397 |
| % Time ICP > 20 mmHg | 1.12 (0.00747 - 5.14) | 1.41 (0.117 - 10.9) | 0.4812 | 1.12 (0 - 5.14) | 1.41 (0.135 - 10.9) | 0.4545 |
| % Time ICP > 22 mmHg | 0.605 (0 - 2.58) | 0.785 (0.0692 - 8.12) | 0.3525 | 0.605 (0 - 2.77) | 0.785 (0.0718 - 4.66) | 0.4472 |
| Mean CPP (mmHg) | 74.4 (70.9 - 80.2) | 73 (67.7 - 77.4) | 0.1494 | 74.6 (71.1 - 80.5) | 72.9 (68.2 - 75.7) | 0.0865 |
| % Time CPP < 60 mmHg | 4.24 (1.26 - 8.07) | 4.91 (1.27 - 17.9) | 0.3034 | 4.24 (0.856 - 8.72) | 4.16 (1.59 - 16.2) | 0.3933 |
| % Time CPP > 70 mmHg | 64.1 (52.2 - 79.4) | 56 (31.3 - 75.4) | 0.1077 | 65.9 (52.8 - 83.1) | 56 (35 - 72.6) | 0.0814 |
| Mean PRx | 0.136 (0.0359 - 0.258) | 0.189 (0.105 - 0.386) | 0.1010 | 0.143 (0.0374 - 0.267) | 0.163 (0.0888 - 0.308) | 0.3800 |
| % Time PRx > 0 | 62.5 (52.2 - 81.4) | 68.7 (62.8 - 86.8) | 0.1017 | 63.3 (52.2 - 82.2) | 66.6 (59.3 - 82.6) | 0.3807 |
| % Time PRx > 0.25 | 37.5 (27.1 - 55.5) | 45.4 (35.8 - 70) | 0.1013 | 38.6 (28 - 56.6) | 42.7 (31.6 - 60.9) | 0.4346 |
| % Time PRx > 0.35 | 27.4 (19.1 - 40.7) | 34.6 (25.6 - 58.8) | 0.1041 | 28.3 (19.5 - 41.7) | 32.6 (22.9 - 48.8) | 0.4303 |
| Mean PAx | -0.0434 (-0.136 - 0.0728) | 0.0499 (-0.0576 - 0.279) | **0.0400** | -0.0403 (-0.129 - 0.0728) | 0.0463 (-0.0911 - 0.273) | 0.0817 |
| % Time PAx > 0 | 45.2 (34.8 - 60.3) | 58 (42.8 - 77.6) | **0.0499** | 47.4 (34.8 - 60.3) | 55.6 (39.2 - 77.6) | 0.1020 |
| % Time PAx > 0.25 | 21.3 (12.2 - 32.7) | 31.7 (18.8 - 57.1) | **0.0469** | 22.5 (12.6 - 32.7) | 29 (16.6 - 56.8) | 0.0927 |
| Mean RAC | -0.274 (-0.404 - -0.133) | -0.122 (-0.282 - 0.0731) | **0.0068** | -0.274 (-0.404 - -0.133) | -0.122 (-0.291 - 0.0726) | **0.0214** |
| % Time RAC > -0.10 | 30.8 (16.9 - 46.7) | 47.6 (30.3 - 65.2) | **0.0093** | 30.8 (16.9 - 46.7) | 46.5 (28.9 - 65.2) | **0.0259** |
| % Time RAC > -0.05 | 25.6 (14.1 - 40.4) | 41.2 (25.7 - 61.1) | **0.0105** | 25.6 (14.1 - 40.4) | 40.4 (25 - 61.1) | **0.0216** |
| CPPopt-PRx | 75 (69.4 - 81.3) | 73.5 (69.3 - 79) | 0.7725 | 75.1 (69.4 - 82.7) | 73.1 (69.3 - 78.9) | 0.5198 |
| % Time ΔCPPopt-PRx > 5 mmHg | 25.7 (17.1 - 33.8) | 23.1 (14.8 - 31.2) | 0.3668 | 25.7 (17.1 - 33.8) | 23.1 (14.8 - 31.2) | 0.4500 |
| % Time ΔCPPopt-PRx > 10 mmHg | 11 (7.06 - 19.5) | 10.2 (5.45 - 16.8) | 0.4004 | 12 (7.31 - 19.7) | 10.1 (5.64 - 16.8) | 0.3813 |
| % Time ΔCPPopt-PRx < -5 mmHg | 24.2 (15.4 - 34.7) | 24.3 (18.7 - 36.1) | 0.6029 | 24.2 (15.6 - 34.7) | 24.3 (17.2 - 35.9) | 0.7550 |
| % Time ΔCPPopt-PRx < -10 mmHg | 9.76 (4.77 - 19.6) | 10.4 (4.91 - 18.6) | 0.8919 | 9.76 (4.77 - 20.3) | 10.4 (4.9 - 18.4) | 0.9730 |
| % Time CPP > ULR-PRx | 12.9 (6.26 - 22.4) | 16 (8.79 - 22.5) | 0.6605 | 14.5 (6.71 - 23.2) | 14.5 (8.2 - 21.6) | 0.9228 |
| % Time CPP < LLR-PRx | 11.2 (4.76 - 26.8) | 14.4 (6.23 - 25.2) | 0.4435 | 11.5 (4.76 - 27.7) | 12.3 (6.23 - 23.8) | 0.7410 |
| CPPopt-PAx | 74.9 (71.8 - 82.6) | 77.1 (71.4 - 81.3) | 0.9372 | 75.7 (71.8 - 82.8) | 74.9 (71.4 - 79.9) | 0.9057 |
| % Time ΔCPPopt-PAx > 5 mmHg | 25.1 (15.8 - 34.9) | 17.9 (12 - 26.1) | **0.0375** | 26.2 (15.8 - 34.9) | 18.3 (12 - 26.1) | 0.0675 |
| % Time ΔCPPopt-PAx > 10 mmHg | 11.5 (6.29 - 21) | 6.63 (4.2 - 12) | **0.0339** | 12.6 (6.03 - 21.6) | 7.03 (4.37 - 11.2) | **0.0346** |
| % Time ΔCPPopt-PAx < -5 mmHg | 28.2 (17.1 - 37) | 36.3 (24.6 - 44.8) | 0.0703 | 26.9 (17.1 - 37) | 35.6 (24.6 - 44.8) | 0.0843 |
| % Time ΔCPPopt-PAx < -10 mmHg | 12.9 (5.46 - 23.5) | 18.4 (11.5 - 27.1) | 0.0771 | 12.9 (5.46 - 23.5) | 17.7 (11 - 27.1) | 0.1074 |
| % Time CPP > ULR-PAx | 5.14 (2.41 - 10.2) | 7.53 (3.29 - 15.4) | 0.2298 | 5.42 (2.5 - 10.2) | 6.75 (2.98 - 15.4) | 0.4560 |
| % Time CPP < LLR-PAx | 4.51 (2.12 - 10.2) | 14.1 (5.04 - 31.4) | **0.0122** | 4.51 (2.12 - 10.2) | 13.3 (3.96 - 31.4) | **0.0268** |
| CPPopt-RAC | 73.2 (69.5 - 78.9) | 73.9 (69.5 - 79.8) | 1.0000 | 73.2 (69.5 - 79.3) | 73.3 (69.5 - 78.2) | 0.8954 |
| % Time ΔCPPopt-RAC > 5 mmHg | 27.6 (17.1 - 40.1) | 23.3 (14.4 - 29) | 0.0640 | 27.8 (17.1 - 40.3) | 23.7 (14.4 - 29) | 0.0887 |
| % Time ΔCPPopt-RAC > 10 mmHg | 12.2 (7.85 - 24.7) | 9.75 (5.91 - 15.4) | 0.1005 | 12.5 (7.85 - 25.2) | 10.1 (6.15 - 14.9) | 0.1044 |
| % Time ΔCPPopt-RAC < -5 mmHg | 19.2 (9.44 - 32.6) | 27.7 (19.7 - 39.8) | **0.0422** | 18.5 (9.44 - 32.6) | 27 (19.7 - 39.4) | 0.0504 |
| % Time ΔCPPopt-RAC < -10 mmHg | 7.94 (2.53 - 17.1) | 13.9 (8.12 - 18.4) | **0.0361** | 7.94 (2.53 - 17.1) | 13.7 (8.01 - 18.4) | 0.0715 |
| % Time CPP > ULR-RAC | 3.45 (1.47 - 7.23) | 4.58 (1.73 - 12.3) | 0.3420 | 3.48 (1.57 - 7.23) | 4.35 (1.7 - 12) | 0.5725 |
| % Time CPP < LLR-RAC | 2.87 (0.965 - 6.97) | 6.36 (2.67 - 16.5) | **0.0388** | 2.87 (0.965 - 6.61) | 6.36 (2.15 - 15.3) | 0.0705 |

*All p-values have been adjusted using the False Discovery Rate (FDR) method. Bolded p-values are those reaching statistical significance, p < 0.05.*

*AMP = pulse amplitude of ICP, CPP = cerebral perfusion pressure, CPPopt = cerebral perfusion pressure optimum, ΔCPPopt = CPP - CPPopt, CT = computed tomography, GCS = Glasgow Coma Scale, ICP = intracranial pressure, ICU = intensive care unit, IQR = interquartile range, LLR = lower limit of reactivity, MAP = mean arterial pressure, mmHg = millimeters of mercury, PAx = pulse amplitude index, PRx = pressure reactivity index, RAC = correlation (R) between slow-waves of AMP (A) and CPP (C), SAH = subarachnoid hemorrhage, ULR = upper limit of reactivity.*

Appendix D. Mann-Whitney U/Chi-Square Testing of Physiologic and Demographic Data for Improved/Not Improved (3-6 Months)

| **Variable** | **3 Month 🡪 6 Month** | | |
| --- | --- | --- | --- |
|  | **Improved**  **Median (IQR)** | **Not Improved**  **Median (IQR)** | **p-value** |
| Age (years) | 38.5 (25.5 - 51) | 44 (32 - 61) | 0.3176 |
| Sex (% Male) | 86.70% | 78.10% | 0.8318 |
| Admission GCS Total | 7 (4.25 - 8.75) | 6 (4 - 8) | 0.6928 |
| Admission GCS Motor | 5 (2.25 - 5) | 4 (2 - 5) | 0.6156 |
| Admission GCS Eyes | 1 (1 - 2) | 1 (1 - 2) | 1.0000 |
| Admission GCS Verbal | 1 (1 - 2) | 1 (1 - 2) | 0.8874 |
| Admission Pupil Response  (% Bilaterally Reactive) | 63.30% | 58.90% | 0.9176 |
| Marshall CT Grade | 3.5 (3 - 5) | 5 (4 - 5) | 0.2059 |
| Rotterdam CT Grade | 4 (3.25 - 5) | 5 (4 - 6) | 0.3357 |
| Helsinki CT Score | 5 (3 - 7.75) | 7 (5 - 9) | 0.2009 |
| Stockholm CT Score | 3 (2.5 - 3.65) | 3.3 (2.5 - 3.9) | 0.5178 |
| Number with Hypoxia Episode | 36.70% | 34.20% | 1.0000 |
| Number with Hypotension Episode | 16.70% | 8.20% | 0.7390 |
| Number with Traumatic SAH | 96.70% | 95.90% | 1.0000 |
| Number with Epidural Hematoma | 16.70% | 8.20% | 0.7135 |
| Admission Hemoglobin | 140 (120 - 154) | 130 (113 - 142) | 0.2546 |
| Admission Serum Glucose | 7.65 (6.25 - 9.38) | 8.3 (7.1 - 11) | 0.3090 |
| Length of Hospital Stay | 31.5 (21 - 52.5) | 14.5 (7.25 - 42) | 0.1205 |
| Length of ICU Stay | 8 (4 - 15) | 7 (4 - 13.5) | 0.8769 |
| Mean MAP (mmHg) | 86 (82 - 89.2) | 82.4 (78.6 - 87.8) | 0.2869 |
| Mean ICP (mmHg) | 8.44 (5.51 - 14.6) | 9.05 (5.98 - 12) | 0.9856 |
| % Time ICP > 20 mmHg | 2.4 (0.00373 - 6.28) | 1.06 (0.0591 - 5.02) | 0.8724 |
| % Time ICP > 22 mmHg | 1.27 (0 - 3.95) | 0.566 (0 - 2.14) | 0.8631 |
| Mean CPP (mmHg) | 75.7 (71.9 - 80.3) | 73 (69.4 - 78.9) | 0.2920 |
| % Time CPP < 60 mmHg | 4 (0.428 - 8.26) | 4.5 (1.29 - 11) | 0.7030 |
| % Time CPP > 70 mmHg | 67.6 (56.8 - 75.4) | 61.4 (41.4 - 79.8) | 0.3221 |
| Mean PRx | 0.16 (0.0386 - 0.31) | 0.146 (0.0477 - 0.276) | 1.0000 |
| % Time PRx > 0 | 69.1 (52.1 - 84.5) | 64.4 (55.6 - 81.3) | 0.9922 |
| % Time PRx > 0.25 | 42 (28.5 - 62.9) | 40.2 (27.7 - 56.4) | 0.9541 |
| % Time PRx > 0.35 | 30.4 (19.9 - 50.5) | 30.1 (19.2 - 45.1) | 0.9198 |
| Mean PAx | 0.0272 (-0.0613 - 0.117) | -0.0129 (-0.105 - 0.117) | 0.8984 |
| % Time PAx > 0 | 53.8 (41 - 65.9) | 47.4 (35.3 - 65.5) | 0.8921 |
| % Time PAx > 0.25 | 26.8 (15.2 - 39.3) | 22.8 (12.7 - 38.1) | 1.0000 |
| Mean RAC | -0.223 (-0.335 - -0.0684) | -0.222 (-0.38 - -0.0375) | 0.9925 |
| % Time RAC > -0.10 | 33.9 (25.3 - 51) | 34.4 (19.6 - 55.9) | 1.0000 |
| % Time RAC > -0.05 | 29.4 (21.6 - 45.5) | 29.9 (17.7 - 50.7) | 1.0000 |
| CPPopt-PRx | 76.4 (71.2 - 83.6) | 73.1 (68.9 - 79.1) | 0.3187 |
| % Time ΔCPPopt-PRx > 5 mmHg | 28.1 (19 - 34.5) | 22.1 (15.1 - 32.7) | 0.3416 |
| % Time ΔCPPopt-PRx > 10 mmHg | 13.7 (7.99 - 20.5) | 10.1 (6.76 - 19) | 0.3445 |
| % Time ΔCPPopt-PRx < -5 mmHg | 23.9 (15.3 - 32.4) | 24.5 (16 - 38.1) | 0.8440 |
| % Time ΔCPPopt-PRx < -10 mmHg | 9.89 (4.97 - 18.4) | 10 (4.94 - 19.4) | 0.9424 |
| % Time CPP > ULR-PRx | 15 (8.79 - 36.6) | 13.6 (6.48 - 21) | 0.3300 |
| % Time CPP < LLR-PRx | 11.7 (6.65 - 31.5) | 11.5 (5.28 - 23.5) | 1.0000 |
| CPPopt-PAx | 75.8 (72.2 - 83.5) | 74.9 (71.2 - 80.8) | 0.8464 |
| % Time ΔCPPopt-PAx > 5 mmHg | 28.3 (20.9 - 37.2) | 18.9 (13.1 - 28.9) | 0.2394 |
| % Time ΔCPPopt-PAx > 10 mmHg | 14.6 (7.19 - 23.1) | 8.11 (4.6 - 15.1) | 0.1630 |
| % Time ΔCPPopt-PAx < -5 mmHg | 23.1 (17.1 - 31.1) | 34.4 (21.7 - 42.9) | 0.1387 |
| % Time ΔCPPopt-PAx < -10 mmHg | 10.9 (5.74 - 17.2) | 16.8 (9.77 - 25.9) | 0.2856 |
| % Time CPP > ULR-PAx | 9.23 (3.39 - 14.4) | 4.92 (2.41 - 11.3) | 0.2731 |
| % Time CPP < LLR-PAx | 5.76 (2.26 - 10.2) | 7.77 (2.63 - 17.1) | 0.8366 |
| CPPopt-RAC | 73.7 (70.1 - 79.4) | 73.1 (69.2 - 78.8) | 0.8865 |
| % Time ΔCPPopt-RAC > 5 mmHg | 32 (22.9 - 43.8) | 24.6 (14.4 - 33.9) | 0.1995 |
| % Time ΔCPPopt-RAC > 10 mmHg | 17.5 (8.77 - 26.7) | 10.5 (6.15 - 17.2) | 0.2856 |
| % Time ΔCPPopt-RAC < -5 mmHg | 18.9 (9.7 - 32.1) | 24.3 (14.1 - 35.6) | 0.3420 |
| % Time ΔCPPopt-RAC < -10 mmHg | 9.07 (2.78 - 17.2) | 10.7 (3.58 - 18.3) | 0.7537 |
| % Time CPP > ULR-RAC | 6 (2.81 - 9.53) | 3.25 (1.63 - 6.94) | 0.2930 |
| % Time CPP < LLR-RAC | 4.14 (1.69 - 8.56) | 4.18 (0.958 - 9.77) | 0.9191 |

*All p-values have been adjusted using the False Discovery Rate (FDR) method. Bolded p-values are those reaching statistical significance, p < 0.05.*

*AMP = pulse amplitude of ICP, CPP = cerebral perfusion pressure, CPPopt = cerebral perfusion pressure optimum, ΔCPPopt = CPP - CPPopt, CT = computed tomography, GCS = Glasgow Coma Scale, ICP = intracranial pressure, ICU = intensive care unit, IQR = interquartile range, LLR = lower limit of reactivity, MAP = mean arterial pressure, mmHg = millimeters of mercury, PAx = pulse amplitude index, PRx = pressure reactivity index, RAC = correlation (R) between slow-waves of AMP (A) and CPP (C), SAH = subarachnoid hemorrhage, ULR = upper limit of reactivity.*

Appendix E. Mann-Whitney U/Chi-Square Testing of Physiologic and Demographic Data for Improved/Not Improved (1-3 Months) with Those Who Died (GOSE=1) Removed

| **Variable** | **1 Month 🡪 3 Month** | | |
| --- | --- | --- | --- |
|  | **Improved**  **Median (IQR)** | **Not Improved**  **Median (IQR)** | **p-value** |
| Age (years) | 37.5 (25 - 49) | 44 (33 - 50.5) | 0.7477 |
| Sex (% Male) | 83.30% | 81.50% | 1.0000 |
| Admission GCS Total | 7 (5 - 8) | 7 (6 - 8) | 0.8699 |
| Admission GCS Motor | 4.5 (3 - 5) | 5 (4 - 5) | 0.8017 |
| Admission GCS Eyes | 1 (1 - 2) | 1 (1 - 2) | 0.9854 |
| Admission GCS Verbal | 1 (1 - 1) | 1 (1 - 2) | 0.8430 |
| Admission Pupil Response  (% Bilaterally Reactive) | 64.30% | 59.30% | 0.8436 |
| Marshall CT Grade | 4 (3 - 5) | 4 (3 - 5) | 0.9675 |
| Rotterdam CT Grade | 4 (3.25 - 5) | 4 (2.5 - 5) | 0.7220 |
| Helsinki CT Score | 6 (4 - 9) | 5 (3.5 - 7.5) | 0.5496 |
| Stockholm CT Score | 3.1 (2.6 - 3.77) | 3 (2.05 - 3.5) | 0.6840 |
| Number with Hypoxia Episode | 38.10% | 29.60% | 0.9660 |
| Number with Hypotension Episode | 14.30% | 11.10% | 1.0000 |
| Number with Traumatic SAH | 97.60% | 92.60% | 0.9875 |
| Number with Epidural Hematoma | 11.90% | 18.50% | 0.9953 |
| Admission Hemoglobin | 136 (120 - 148) | 132 (112 - 146) | 0.8322 |
| Admission Serum Glucose | 8.05 (7.15 - 10.1) | 7.5 (6.4 - 9.65) | 0.9504 |
| Length of Hospital Stay | 42 (30 - 79) | 22 (12.5 - 41.5) | 0.2839 |
| Length of ICU Stay | 11.5 (4.25 - 18) | 7 (4 - 12) | 0.6156 |
| Mean MAP (mmHg) | 84.1 (80.7 - 92.1) | 82.8 (79.2 - 86.4) | 0.8339 |
| Mean ICP (mmHg) | 8.95 (5.51 - 12) | 7.67 (3.77 - 12) | 0.8461 |
| % Time ICP > 20 mmHg | 1.06 (0 - 5.19) | 1.12 (0.126 - 3.63) | 0.9963 |
| % Time ICP > 22 mmHg | 0.489 (0 - 2.58) | 0.605 (0.0426 - 1.94) | 0.9634 |
| Mean CPP (mmHg) | 74.4 (71.2 - 80.3) | 75.2 (70.9 - 81.6) | 0.9907 |
| % Time CPP < 60 mmHg | 4.14 (1.47 - 8.85) | 3.95 (0.276 - 7.14) | 0.8275 |
| % Time CPP > 70 mmHg | 65.7 (54.5 - 80.7) | 64.4 (50.5 - 83.8) | 0.9975 |
| Mean PRx | 0.102 (0.0111 - 0.212) | 0.171 (0.0602 - 0.33) | 0.5804 |
| % Time PRx > 0 | 60.2 (51.6 - 74.1) | 68.3 (56.8 - 85.7) | 0.5525 |
| % Time PRx > 0.25 | 35.9 (26.4 - 46.9) | 40.2 (30.8 - 64.9) | 0.5558 |
| % Time PRx > 0.35 | 26.7 (19 - 35.3) | 30.3 (23.1 - 51.8) | 0.7196 |
| Mean PAx | -0.0507 (-0.142 - 0.0302) | 0.0346 (-0.125 - 0.179) | 1.0000 |
| % Time PAx > 0 | 43.2 (34 - 53.8) | 53.9 (35.8 - 71.6) | 0.7847 |
| % Time PAx > 0.25 | 17.7 (12.6 - 27.5) | 27.2 (14.2 - 46.4) | 0.5885 |
| Mean RAC | -0.328 (-0.432 - -0.175) | -0.214 (-0.348 - -0.0559) | 0.5803 |
| % Time RAC > -0.10 | 25.3 (13 - 42.2) | 35.9 (25.1 - 53.2) | 0.6148 |
| % Time RAC > -0.05 | 22.3 (10.8 - 37) | 30.3 (21.7 - 47.7) | 0.5748 |
| CPPopt-PRx | 74.4 (68.7 - 81.2) | 77 (70.5 - 82.1) | 0.8469 |
| % Time ΔCPPopt-PRx > 5 mmHg | 26.5 (17.9 - 33.8) | 25.2 (16.2 - 36.4) | 1.0000 |
| % Time ΔCPPopt-PRx > 10 mmHg | 12.5 (8.88 - 19.8) | 8.99 (6.16 - 20) | 0.8835 |
| % Time ΔCPPopt-PRx < -5 mmHg | 25.4 (15.7 - 32.8) | 24 (16.1 - 36.8) | 1.0000 |
| % Time ΔCPPopt-PRx < -10 mmHg | 11.9 (5.17 - 19) | 8.86 (4.46 - 19.9) | 0.9910 |
| % Time CPP > ULR-PRx | 12.9 (6.27 - 20.3) | 15 (6.93 - 29.9) | 0.8576 |
| % Time CPP < LLR-PRx | 9.15 (5.36 - 23.1) | 14.2 (4.22 - 28.7) | 0.8349 |
| CPPopt-PAx | 74.6 (71.8 - 82.3) | 75.7 (71.7 - 85.5) | 0.9500 |
| % Time ΔCPPopt-PAx > 5 mmHg | 26.3 (15.7 - 35.1) | 24 (16.3 - 31.3) | 0.8503 |
| % Time ΔCPPopt-PAx > 10 mmHg | 12.3 (6.38 - 22.5) | 10.8 (6.29 - 17.9) | 0.8037 |
| % Time ΔCPPopt-PAx < -5 mmHg | 29.9 (18.3 - 37.8) | 26.9 (16.7 - 31.2) | 0.8265 |
| % Time ΔCPPopt-PAx < -10 mmHg | 12.7 (6.42 - 25.5) | 14.6 (5.36 - 19.8) | 0.9804 |
| % Time CPP > ULR-PAx | 4.2 (2.46 - 8.94) | 7.19 (2.56 - 15) | 0.7766 |
| % Time CPP < LLR-PAx | 4.98 (2.1 - 10) | 4.73 (2.23 - 15.7) | 0.8375 |
| CPPopt-RAC | 72.9 (70.1 - 78.4) | 73.4 (69.1 - 85.1) | 0.9824 |
| % Time ΔCPPopt-RAC > 5 mmHg | 31.6 (15.4 - 41) | 25.8 (19.9 - 38.9) | 0.9888 |
| % Time ΔCPPopt-RAC > 10 mmHg | 15.1 (7.46 - 25.4) | 11.3 (7.85 - 21.5) | 0.8205 |
| % Time ΔCPPopt-RAC < -5 mmHg | 21.9 (11.2 - 33.7) | 17 (8.39 - 28.8) | 0.8764 |
| % Time ΔCPPopt-RAC < -10 mmHg | 9.01 (2.52 - 17.9) | 7.94 (2.78 - 17.8) | 1.0000 |
| % Time CPP > ULR-RAC | 3.3 (1.48 - 6.44) | 4.04 (1.57 - 9.21) | 0.8821 |
| % Time CPP < LLR-RAC | 3.7 (1.03 - 6.78) | 2.87 (0.889 - 7.89) | 1.0000 |

*All p-values have been adjusted using the False Discovery Rate (FDR) method. Bolded p-values are those reaching statistical significance, p < 0.05.*

*AMP = pulse amplitude of ICP, CPP = cerebral perfusion pressure, CPPopt = cerebral perfusion pressure optimum, ΔCPPopt = CPP - CPPopt, CT = computed tomography, GCS = Glasgow Coma Scale, GOSE = Glasgow Outcome Scale-Extended, ICP = intracranial pressure, ICU = intensive care unit, IQR = interquartile range, LLR = lower limit of reactivity, MAP = mean arterial pressure, mmHg = millimeters of mercury, PAx = pulse amplitude index, PRx = pressure reactivity index, RAC = correlation (R) between slow-waves of AMP (A) and CPP (C), SAH = subarachnoid hemorrhage, ULR = upper limit of reactivity.*

Appendix F. Mann-Whitney U/Chi-Square Testing of Physiologic and Demographic Data for Improved/Not Improved (3-6 Months) with Those Who Died (GOSE=1) Removed

| **Variable** | **3 Month 🡪 6 Month** | | |
| --- | --- | --- | --- |
|  | **Improved**  **Median (IQR)** | **Not Improved**  **Median (IQR)** | **p-value** |
| Age (years) | 38.5 (25.5 - 51) | 39 (26 - 46) | 0.8624 |
| Sex (% Male) | 86.70% | 78.40% | 0.8008 |
| Admission GCS Total | 7 (4.25 - 8.75) | 7 (6 - 8) | 0.9939 |
| Admission GCS Motor | 5 (2.25 - 5) | 5 (4 - 5) | 0.8526 |
| Admission GCS Eyes | 1 (1 - 2) | 1 (1 - 2) | 0.9438 |
| Admission GCS Verbal | 1 (1 - 2) | 1 (1 - 1) | 0.8265 |
| Admission Pupil Response  (% Bilaterally Reactive) | 63.30% | 62.20% | 0.8484 |
| Marshall CT Grade | 3.5 (3 - 5) | 5 (3 - 5) | 0.4510 |
| Rotterdam CT Grade | 4 (3.25 - 5) | 5 (3 - 5) | 0.7215 |
| Helsinki CT Score | 5 (3 - 7.75) | 6 (4 - 9) | 0.5059 |
| Stockholm CT Score | 3 (2.5 - 3.65) | 3.1 (2.4 - 3.5) | 0.7235 |
| Number with Hypoxia Episode | 36.70% | 32.40% | 0.9679 |
| Number with Hypotension Episode | 16.70% | 10.80% | 0.8728 |
| Number with Traumatic SAH | 96.70% | 94.60% | 1.0000 |
| Number with Epidural Hematoma | 16.70% | 13.50% | 1.0000 |
| Admission Hemoglobin | 140 (120 - 154) | 131 (113 - 143) | 0.5624 |
| Admission Serum Glucose | 7.65 (6.25 - 9.38) | 8.1 (7.1 - 10.5) | 0.5198 |
| Length of Hospital Stay | 31.5 (21 - 52.5) | 41 (17 - 66.5) | 0.6791 |
| Length of ICU Stay | 8 (4 - 15) | 11 (4 - 17) | 0.9561 |
| Mean MAP (mmHg) | 86 (82 - 89.2) | 81.9 (79 - 85.8) | 0.4697 |
| Mean ICP (mmHg) | 8.44 (5.51 - 14.6) | 9.05 (3.35 - 11.5) | 0.7331 |
| % Time ICP > 20 mmHg | 2.4 (0.00373 - 6.28) | 0.727 (0.0402 - 2.76) | 0.4893 |
| % Time ICP > 22 mmHg | 1.27 (0 - 3.95) | 0.407 (0 - 1.23) | 0.4560 |
| Mean CPP (mmHg) | 75.7 (71.9 - 80.3) | 73 (70.5 - 80) | 0.5278 |
| % Time CPP < 60 mmHg | 4 (0.428 - 8.26) | 4.5 (1.45 - 6.95) | 0.8744 |
| % Time CPP > 70 mmHg | 67.6 (56.8 - 75.4) | 62.3 (51.6 - 82.8) | 0.6391 |
| Mean PRx | 0.16 (0.0386 - 0.31) | 0.117 (0.00649 - 0.182) | 0.4690 |
| % Time PRx > 0 | 69.1 (52.1 - 84.5) | 62.4 (52.4 - 74.2) | 0.5847 |
| % Time PRx > 0.25 | 42 (28.5 - 62.9) | 34.5 (25.2 - 42.9) | 0.4370 |
| % Time PRx > 0.35 | 30.4 (19.9 - 50.5) | 24.8 (18.6 - 33.3) | 0.5054 |
| Mean PAx | 0.0272 (-0.0613 - 0.117) | -0.0883 (-0.146 - 0.0332) | 0.4427 |
| % Time PAx > 0 | 53.8 (41 - 65.9) | 39.2 (34.2 - 53.1) | 0.4090 |
| % Time PAx > 0.25 | 26.8 (15.2 - 39.3) | 16.6 (11.9 - 28.7) | 0.4385 |
| Mean RAC | -0.223 (-0.335 - -0.0684) | -0.366 (-0.425 - -0.193) | 0.4275 |
| % Time RAC > -0.10 | 33.9 (25.3 - 51) | 24.8 (14.3 - 37.9) | 0.3851 |
| % Time RAC > -0.05 | 29.4 (21.6 - 45.5) | 21.7 (12 - 33.1) | 0.3466 |
| CPPopt-PRx | 76.4 (71.2 - 83.6) | 72.8 (68.6 - 79.1) | 0.4972 |
| % Time ΔCPPopt-PRx > 5 mmHg | 28.1 (19 - 34.5) | 22 (16.7 - 32.7) | 0.5042 |
| % Time ΔCPPopt-PRx > 10 mmHg | 13.7 (7.99 - 20.5) | 9.8 (6.96 - 19) | 0.5054 |
| % Time ΔCPPopt-PRx < -5 mmHg | 23.9 (15.3 - 32.4) | 24.9 (15.7 - 38.4) | 0.8382 |
| % Time ΔCPPopt-PRx < -10 mmHg | 9.89 (4.97 - 18.4) | 9.76 (4.94 - 19.9) | 0.9243 |
| % Time CPP > ULR-PRx | 15 (8.79 - 36.6) | 11.1 (5.77 - 17.6) | 0.5515 |
| % Time CPP < LLR-PRx | 11.7 (6.65 - 31.5) | 9.86 (4.05 - 22.3) | 0.7256 |
| CPPopt-PAx | 75.8 (72.2 - 83.5) | 74.7 (71.2 - 80.6) | 0.6890 |
| % Time ΔCPPopt-PAx > 5 mmHg | 28.3 (20.9 - 37.2) | 24 (13.4 - 31.3) | 0.4182 |
| % Time ΔCPPopt-PAx > 10 mmHg | 14.6 (7.19 - 23.1) | 9.27 (5.76 - 17.7) | 0.4710 |
| % Time ΔCPPopt-PAx < -5 mmHg | 23.1 (17.1 - 31.1) | 31 (19.6 - 37.9) | 0.4641 |
| % Time ΔCPPopt-PAx < -10 mmHg | 10.9 (5.74 - 17.2) | 15.6 (5.44 - 25.6) | 0.5664 |
| % Time CPP > ULR-PAx | 9.23 (3.39 - 14.4) | 3.74 (1.81 - 6.3) | 0.1129 |
| % Time CPP < LLR-PAx | 5.76 (2.26 - 10.2) | 3.99 (2.1 - 9.37) | 0.5211 |
| CPPopt-RAC | 73.7 (70.1 - 79.4) | 72.7 (69.2 - 77.4) | 0.7154 |
| % Time ΔCPPopt-RAC > 5 mmHg | 32 (22.9 - 43.8) | 26.8 (14.4 - 37.2) | 0.4795 |
| % Time ΔCPPopt-RAC > 10 mmHg | 17.5 (8.77 - 26.7) | 11.1 (7.2 - 19.2) | 0.5130 |
| % Time ΔCPPopt-RAC < -5 mmHg | 18.9 (9.7 - 32.1) | 19.2 (9.87 - 33) | 0.8550 |
| % Time ΔCPPopt-RAC < -10 mmHg | 9.07 (2.78 - 17.2) | 5.89 (2.29 - 17) | 0.8608 |
| % Time CPP > ULR-RAC | 6 (2.81 - 9.53) | 2.41 (1.14 - 4.34) | 0.1787 |
| % Time CPP < LLR-RAC | 4.14 (1.69 - 8.56) | 2.12 (0.775 - 4.7) | 0.4185 |

*All p-values have been adjusted using the False Discovery Rate (FDR) method. Bolded p-values are those reaching statistical significance, p < 0.05.*

*AMP = pulse amplitude of ICP, CPP = cerebral perfusion pressure, CPPopt = cerebral perfusion pressure optimum, ΔCPPopt = CPP - CPPopt, CT = computed tomography, GCS = Glasgow Coma Scale, GOSE = Glasgow Outcome Scale-Extended, ICP = intracranial pressure, ICU = intensive care unit, IQR = interquartile range, LLR = lower limit of reactivity, MAP = mean arterial pressure, mmHg = millimeters of mercury, PAx = pulse amplitude index, PRx = pressure reactivity index, RAC = correlation (R) between slow-waves of AMP (A) and CPP (C), SAH = subarachnoid hemorrhage, ULR = upper limit of reactivity.*

Appendix G. Mann-Whitney U/Chi-Square Testing of Physiologic and Demographic Data for Improved/Not Improved (1-6 Months) with Those Who Died (GOSE=1) Removed

| **Variable** | **1 Month 🡪 6 Month** | | |
| --- | --- | --- | --- |
|  | **Improved**  **Median (IQR)** | **Not Improved**  **Median (IQR)** | **p-value** |
| Age (years) | 36 (24 - 50) | 45.5 (42.5 - 49.8) | 0.6555 |
| Sex (% Male) | 84.90% | 75% | 0.7298 |
| Admission GCS Total | 7 (5 - 8) | 7 (6 - 8.25) | 0.6638 |
| Admission GCS Motor | 4 (3 - 5) | 5 (4 - 5) | 0.6182 |
| Admission GCS Eyes | 1 (1 - 2) | 1 (1 - 2) | 0.8550 |
| Admission GCS Verbal | 1 (1 - 1) | 1 (1 - 2) | 0.8282 |
| Admission Pupil Response  (% Bilaterally Reactive) | 62.30% | 62.50% | 0.6899 |
| Marshall CT Grade | 4 (3 - 5) | 3.5 (3 - 5) | 0.8194 |
| Rotterdam CT Grade | 4 (4 - 5) | 3 (2 - 5) | 0.7154 |
| Helsinki CT Score | 6 (4 - 9) | 4.5 (3.5 - 5.25) | 0.8713 |
| Stockholm CT Score | 3.1 (2.9 - 3.8) | 2.2 (2 - 3.3) | 1.0000 |
| Number with Hypoxia Episode | 37.70% | 25% | 0.7271 |
| Number with Hypotension Episode | 17% | 0% | 0.6377 |
| Number with Traumatic SAH | 98.10% | 87.50% | 0.6762 |
| Number with Epidural Hematoma | 13.20% | 18.80% | 0.8988 |
| Admission Hemoglobin | 138 (116 - 149) | 130 (110 - 138) | 0.7182 |
| Admission Serum Glucose | 8.1 (7 - 10.2) | 7.4 (6.38 - 9.2) | 0.6538 |
| Length of Hospital Stay | 36.5 (22 - 62.5) | 23.5 (10.5 - 47.8) | 0.6365 |
| Length of ICU Stay | 11 (4 - 17) | 6 (3.75 - 11) | 0.6581 |
| Mean MAP (mmHg) | 84.6 (81.1 - 89.4) | 80.5 (77.9 - 84.8) | 0.6869 |
| Mean ICP (mmHg) | 9.13 (5.95 - 12.5) | 7.36 (3.02 - 10.1) | 0.8550 |
| % Time ICP > 20 mmHg | 1.66 (0 - 5.76) | 0.821 (0.177 - 1.63) | 0.7185 |
| % Time ICP > 22 mmHg | 0.923 (0 - 3.02) | 0.552 (0.0878 - 0.902) | 0.7216 |
| Mean CPP (mmHg) | 75.1 (71.3 - 80.6) | 73.4 (70.3 - 79.3) | 0.7030 |
| % Time CPP < 60 mmHg | 3.81 (1.45 - 7.7) | 4.78 (0.554 - 6.74) | 0.9260 |
| % Time CPP > 70 mmHg | 67.2 (54.9 - 82.8) | 64.2 (46.6 - 83.6) | 0.6529 |
| Mean PRx | 0.136 (0.0347 - 0.237) | 0.137 (0.048 - 0.307) | 0.7081 |
| % Time PRx > 0 | 62.5 (51.9 - 78.4) | 64.3 (55.3 - 86.2) | 0.6400 |
| % Time PRx > 0.25 | 38.6 (27.3 - 54.5) | 38.2 (29.5 - 60.3) | 0.7424 |
| % Time PRx > 0.35 | 27.4 (19.1 - 39.6) | 28.8 (21.5 - 47.1) | 0.7329 |
| Mean PAx | -0.0437 (-0.142 - 0.0607) | -0.0201 (-0.125 - 0.122) | 0.6591 |
| % Time PAx > 0 | 44.8 (33.9 - 58.9) | 47.1 (35.9 - 68) | 0.7434 |
| % Time PAx > 0.25 | 18.5 (12.5 - 29.4) | 22.9 (15.4 - 40.2) | 0.7583 |
| Mean RAC | -0.285 (-0.425 - -0.153) | -0.223 (-0.366 - -0.0581) | 0.6810 |
| % Time RAC > -0.10 | 29.4 (14.3 - 46) | 33.5 (23 - 52.4) | 0.6779 |
| % Time RAC > -0.05 | 25.6 (12 - 39.6) | 28 (19.7 - 46.9) | 0.7136 |
| CPPopt-PRx | 74.4 (70 - 83.6) | 76.3 (69.3 - 80) | 0.8799 |
| % Time ΔCPPopt-PRx > 5 mmHg | 27.9 (18.6 - 34.9) | 20.9 (14.4 - 27.5) | 0.7600 |
| % Time ΔCPPopt-PRx > 10 mmHg | 12.5 (8.99 - 20.8) | 7.06 (5.65 - 13) | 0.7961 |
| % Time ΔCPPopt-PRx < -5 mmHg | 23.6 (14.7 - 32.9) | 27 (20.1 - 37.7) | 0.6636 |
| % Time ΔCPPopt-PRx < -10 mmHg | 9.09 (4.5 - 17.9) | 14.6 (6.49 - 21.6) | 0.6236 |
| % Time CPP > ULR-PRx | 14.5 (6.94 - 24) | 12.1 (6.14 - 22.7) | 0.8322 |
| % Time CPP < LLR-PRx | 9.86 (4.58 - 23.4) | 16.7 (4.66 - 31.4) | 0.6745 |
| CPPopt-PAx | 75.7 (71.8 - 82.8) | 74.8 (70.6 - 79.7) | 0.8594 |
| % Time ΔCPPopt-PAx > 5 mmHg | 27.4 (18.7 - 35.1) | 20.4 (13.9 - 30.3) | 0.6194 |
| % Time ΔCPPopt-PAx > 10 mmHg | 12.9 (6.57 - 22.7) | 8.34 (5.76 - 16.1) | 0.6033 |
| % Time ΔCPPopt-PAx < -5 mmHg | 28.2 (17.1 - 37.5) | 29.1 (22.2 - 31.6) | 0.8613 |
| % Time ΔCPPopt-PAx < -10 mmHg | 12 (5.44 - 25.2) | 16.7 (8.56 - 19.1) | 0.6686 |
| % Time CPP > ULR-PAx | 5.68 (2.59 - 10.8) | 4.09 (2.4 - 8.28) | 0.7725 |
| % Time CPP < LLR-PAx | 4.51 (2.1 - 10.1) | 6.96 (2.47 - 13.6) | 0.6993 |
| CPPopt-RAC | 73.2 (70 - 79.5) | 73.2 (69 - 80.2) | 0.8394 |
| % Time ΔCPPopt-RAC > 5 mmHg | 31.4 (17.5 - 41.3) | 25.1 (14.4 - 37.2) | 0.6612 |
| % Time ΔCPPopt-RAC > 10 mmHg | 14.5 (8.62 - 25.8) | 8.94 (7.09 - 17.4) | 0.7990 |
| % Time ΔCPPopt-RAC < -5 mmHg | 19.2 (9.01 - 32.1) | 27 (13 - 36.7) | 0.6630 |
| % Time ΔCPPopt-RAC < -10 mmHg | 7.94 (2.51 - 15.4) | 13.7 (2.94 - 21.7) | 0.6921 |
| % Time CPP > ULR-RAC | 3.48 (1.83 - 7.47) | 3.28 (1.18 - 4.62) | 0.6349 |
| % Time CPP < LLR-RAC | 3.73 (1.02 - 7.4) | 2.2 (0.924 - 4.69) | 0.7162 |

*All p-values have been adjusted using the False Discovery Rate (FDR) method. Bolded p-values are those reaching statistical significance, p < 0.05.*

*AMP = pulse amplitude of ICP, CPP = cerebral perfusion pressure, CPPopt = cerebral perfusion pressure optimum, ΔCPPopt = CPP - CPPopt, CT = computed tomography, GCS = Glasgow Coma Scale, GOSE = Glasgow Outcome Scale-Extended, ICP = intracranial pressure, ICU = intensive care unit, IQR = interquartile range, LLR = lower limit of reactivity, MAP = mean arterial pressure, mmHg = millimeters of mercury, PAx = pulse amplitude index, PRx = pressure reactivity index, RAC = correlation (R) between slow-waves of AMP (A) and CPP (C), SAH = subarachnoid hemorrhage, ULR = upper limit of reactivity.*

Appendix H. Mann-Whitney U/Chi-Square Testing of Physiologic and Demographic Data for Improved/Not Improved (1-3 Months) with Patients Trichotomized by Age

| **Variable** | **Age < 30 (n = 29)** | | | **Age 30 – 60 (n = 53)** | | | **Age > 60 (n = 21)** | | |
| --- | --- | --- | --- | --- | --- | --- | --- | --- | --- |
|  | **Improved**  **Median (IQR)** | **Not Improved**  **Median (IQR)** | **p-value** | **Improved**  **Median (IQR)** | **Not Improved**  **Median (IQR)** | **p-value** | **Improved**  **Median (IQR)** | **Not Improved**  **Median (IQR)** | **p-value** |
| Age (years) | 24 (20.5 - 25) | 23 (21.2 - 24.8) | 1 | 42 (37.8 - 50) | 47 (41 - 52) | 0.8225 | 63 (62 - 63) | 67 (63.8 - 72.8) | 0.788 |
| Sex (% Male) | 80% | 85.70% | 1 | 87.50% | 75.90% | 1 | 66.70% | 77.80% | 1 |
| Admission GCS Total | 6 (5 - 7.5) | 6 (4 - 6.75) | 0.6162 | 7 (4.75 - 8) | 7 (4 - 8) | 1 | 9 (8 - 9.5) | 6.5 (4 - 8) | 0.9908 |
| Admission GCS Motor | 4 (3 - 5) | 4 (2 - 4.75) | 0.7117 | 4.5 (2.75 - 5) | 4 (2 - 5) | 1 | 5 (4.5 - 5) | 3.5 (2 - 4.75) | 0.9755 |
| Admission GCS Eyes | 1 (1 - 1.5) | 1 (1 - 1) | 0.4241 | 1 (1 - 2) | 1 (1 - 2) | 1 | 2 (1.5 - 2.5) | 1.5 (1 - 2) | 1 |
| Admission GCS Verbal | 1 (1 - 1) | 1 (1 - 1) | 0.5535 | 1 (1 - 1.25) | 1 (1 - 2) | 1 | 2 (2 - 2) | 1 (1 - 2) | 0.9755 |
| Admission Pupil Response  (% Bilaterally Reactive) | 66.70% | 50% | 0.6898 | 58.30% | 58.60% | 1 | 100% | 61.10% | 1 |
| Marshall CT Grade | 3 (3 - 4.5) | 4.5 (4 - 5) | 0.2284 | 4.5 (3 - 5) | 4 (3 - 5) | 1 | 5 (5 - 5) | 5 (4 - 5) | 1 |
| Rotterdam CT Grade | 4 (3 - 4) | 5 (4 - 6) | 0.2284 | 5 (3.75 - 6) | 4 (3 - 5) | 0.8225 | 5 (4.5 - 5.5) | 4.5 (4 - 5.75) | 1 |
| Helsinki CT Score | 5 (3 - 5.5) | 7.5 (5.25 - 10.5) | 0.2284 | 8 (5.75 - 9) | 5 (4 - 9) | 0.6076 | 7 (6 - 7.5) | 7.5 (6 - 9) | 1 |
| Stockholm CT Score | 3 (2.5 - 3.5) | 3.4 (2.5 - 3.5) | 0.8783 | 3.35 (2.78 - 3.9) | 3 (2.2 - 3.5) | 0.841 | 3.1 (3.05 - 3.55) | 3.55 (2.85 - 4.22) | 1 |
| Number with Hypoxia Episode | 40% | 42.90% | 1 | 41.70% | 34.50% | 1 | 0% | 22.20% | 1 |
| Number with Hypotension Episode | 26.70% | 7.10% | 0.5945 | 8.30% | 6.90% | 1 | 0% | 11.10% | 1 |
| Number with Traumatic SAH | 100% | 92.90% | 1 | 95.80% | 96.60% | 1 | 100% | 94.40% | 1 |
| Admission Hemoglobin | 140 (124 - 149) | 135 (124 - 150) | 0.9422 | 136 (122 - 144) | 129 (113 - 144) | 1 | 129 (122 - 140) | 125 (112 - 136) | 1 |
| Admission Serum Glucose | 8.2 (7.45 - 10.4) | 9.2 (7.08 - 12.4) | 0.9199 | 7.8 (6.4 - 9.7) | 7.7 (6.4 - 9.9) | 1 | 8.4 (8.1 - 10.8) | 8.05 (7.12 - 11.8) | 1 |
| Length of Hospital Stay | 68 (35 - 87.5) | 11 (7 - 31) | **0.0268** | 34 (28 - 63) | 19 (9 - 39) | 0.29 | 42 (26 - 48) | 8 (3 - 12) | 0.5011 |
| Length of ICU Stay | 15 (10.5 - 24) | 6 (4 - 9) | 0.1146 | 7 (3.75 - 16.2) | 7 (4 - 13) | 1 | 8 (5 - 16) | 6 (3 - 10) | 1 |
| Mean MAP (mmHg) | 85.3 (82.2 - 94.3) | 82.1 (77.2 - 86.4) | 0.3886 | 83.8 (80.6 - 92) | 86 (80.8 - 89.4) | 1 | 80.4 (75.3 - 81.4) | 79.5 (77.9 - 84.8) | 1 |
| Mean ICP (mmHg) | 10.9 (8.55 - 13.9) | 13.3 (7.39 - 16.4) | 0.6162 | 8.44 (3.49 - 11) | 7.92 (4.19 - 12.4) | 1 | 5.95 (2.27 - 7.06) | 8.54 (5.5 - 10.8) | 1 |
| % Time ICP > 20 mmHg | 3.32 (0.223 - 6.2) | 6.37 (1.6 - 36.7) | 0.3818 | 0.734 (0 - 3.39) | 1.12 (0.0591 - 4.09) | 1 | 0.0149 (0.00747 - 0.0619) | 0.353 (0.142 - 1.91) | 0.788 |
| % Time ICP > 22 mmHg | 1.22 (0.0723 - 2.98) | 4.88 (0.739 - 27.8) | 0.3314 | 0.31 (0 - 1.9) | 0.605 (0 - 2.29) | 1 | 0 (0 - 0) | 0.22 (0.0908 - 0.975) | 0.5011 |
| Mean CPP (mmHg) | 74.6 (69.4 - 80.2) | 68.4 (65.3 - 72.9) | 0.129 | 74.6 (71.5 - 80.6) | 75.2 (71.8 - 81.4) | 1 | 74.4 (73.2 - 74.4) | 72.6 (68.7 - 76.8) | 1 |
| % Time CPP < 60 mmHg | 4.04 (2.98 - 9.75) | 12.2 (3.69 - 28.4) | 0.2284 | 3.44 (0.964 - 9.54) | 2.76 (0.241 - 7.49) | 0.9831 | 5.88 (3.66 - 6.35) | 3.34 (1.51 - 14.6) | 1 |
| % Time CPP > 70 mmHg | 67.5 (44.6 - 77.3) | 39.5 (29.9 - 56) | 0.1146 | 68.4 (56.4 - 83.6) | 70.4 (52.3 - 84.1) | 1 | 57 (54.3 - 60.2) | 55.1 (41.5 - 69.4) | 1 |
| Mean PRx | 0.0205 (-0.0944 - 0.112) | 0.163 (0.103 - 0.397) | 0.129 | 0.151 (0.0541 - 0.257) | 0.199 (0.0731 - 0.363) | 0.9831 | 0.0477 (0.0278 - 0.186) | 0.137 (0.079 - 0.257) | 1 |
| % Time PRx > 0 | 49.2 (37.1 - 62.8) | 66.2 (61 - 86.2) | 0.2284 | 66.2 (56.3 - 82.3) | 73.6 (58.2 - 87.2) | 0.9831 | 54 (52.8 - 67.4) | 65.2 (59.4 - 79.2) | 1 |
| % Time PRx > 0.25 | 23.3 (17.4 - 33.7) | 42.1 (33.6 - 69.1) | 0.129 | 40.6 (31.2 - 56.5) | 49.6 (34.5 - 68.2) | 1 | 27.7 (27.5 - 45.7) | 39.4 (31.3 - 56) | 1 |
| % Time PRx > 0.35 | 17.5 (10.9 - 25) | 31.7 (24.5 - 59.6) | 0.1146 | 30.2 (22.8 - 41.2) | 38.7 (24.6 - 57.3) | 0.9831 | 19.1 (19.1 - 36) | 30 (22.7 - 44.3) | 1 |
| Mean PAx | -0.0917 (-0.225 - -0.0492) | 0.0162 (-0.197 - 0.22) | 0.3886 | -0.0214 (-0.102 - 0.0362) | 0.0678 (-0.0918 - 0.232) | 0.29 | 0.0332 (-0.0276 - 0.048) | 0.0848 (-0.0425 - 0.195) | 1 |
| % Time PAx > 0 | 35.3 (25 - 42.8) | 48.1 (27.8 - 71.9) | 0.3886 | 49 (37 - 54.4) | 59.1 (39.4 - 80.3) | 0.29 | 53.1 (44.7 - 56.1) | 61.3 (44.3 - 74.8) | 1 |
| % Time PAx > 0.25 | 12.7 (7.77 - 17.7) | 23.7 (11.4 - 49.8) | 0.3837 | 23.2 (15.5 - 28.6) | 33.4 (18.6 - 51.3) | 0.29 | 35.7 (24.1 - 35.8) | 31.2 (19.4 - 46.8) | 1 |
| Mean RAC | -0.406 (-0.528 - -0.229) | -0.186 (-0.365 - -0.0476) | 0.2284 | -0.295 (-0.391 - -0.168) | -0.197 (-0.313 - -0.0516) | 0.29 | -0.0122 (-0.0957 - 0.00186) | -0.0209 (-0.232 - 0.109) | 1 |
| % Time RAC > -0.10 | 18.4 (10.7 - 33.6) | 33.6 (19.5 - 58.1) | 0.2845 | 27.4 (18.1 - 43.6) | 37.9 (28.3 - 54.8) | 0.29 | 59.5 (48.6 - 59.6) | 57.9 (31.9 - 71.5) | 1 |
| % Time RAC > -0.05 | 16.1 (8.53 - 28.2) | 30.8 (16.1 - 52.4) | 0.2732 | 24.1 (14.7 - 38.1) | 33.1 (24.2 - 49.3) | 0.29 | 55.6 (42.7 - 55.6) | 52.3 (26.4 - 66.9) | 1 |
| CPPopt-PRx | 71.9 (68.5 - 80.1) | 70.7 (69.7 - 80) | 1 | 75.5 (71.5 - 82.4) | 77.2 (71.3 - 80.1) | 1 | 68.3 (67 - 68.7) | 72.5 (69.7 - 77.8) | 0.5011 |
| % Time ΔCPPopt-PRx > 5 mmHg | 26.8 (20.3 - 34.6) | 23.2 (9.21 - 29.1) | 0.3837 | 25.8 (16.8 - 32.2) | 24.1 (15.1 - 34.1) | 1 | 16.7 (14.1 - 33.8) | 21.7 (18.4 - 33.5) | 1 |
| % Time ΔCPPopt-PRx > 10 mmHg | 12 (9.45 - 19.5) | 9.03 (2.95 - 12.1) | 0.2958 | 12.8 (8.92 - 20.9) | 8.99 (6.63 - 19) | 0.9831 | 9.65 (6.66 - 20.7) | 10.7 (5.87 - 18.5) | 1 |
| % Time ΔCPPopt-PRx < -5 mmHg | 23.6 (15.8 - 30.9) | 18.2 (15 - 41) | 0.9422 | 29.4 (18.6 - 40.8) | 24.5 (19 - 37.4) | 1 | 7.3 (6.4 - 7.88) | 24.7 (16.8 - 33.7) | 0.3492 |
| % Time ΔCPPopt-PRx < -10 mmHg | 6.62 (5.55 - 12.3) | 5.33 (2.5 - 20.5) | 0.6105 | 15.9 (7.17 - 22.7) | 13.9 (6.06 - 20.9) | 1 | 2.58 (1.98 - 3.49) | 10.3 (6.17 - 17.4) | 0.5011 |
| % Time CPP > ULR-PRx | 6.94 (5.17 - 13.1) | 15.5 (10.1 - 17.8) | 0.2732 | 15.3 (9.03 - 24.3) | 14.9 (7.37 - 27.1) | 1 | 15 (8.95 - 18.7) | 15 (7.74 - 22.3) | 1 |
| % Time CPP < LLR-PRx | 7.32 (5.6 - 10.5) | 7.95 (4.67 - 22.1) | 0.9422 | 12.3 (6.79 - 29.2) | 18 (8.59 - 31.6) | 1 | 2.57 (1.77 - 9.36) | 14.8 (9.62 - 19.8) | 1 |
| CPPopt-PAx | 72.5 (68.8 - 78.7) | 73 (67.7 - 78.8) | 0.672 | 76.4 (73.5 - 82.1) | 78.5 (73.2 - 83.8) | 1 | 72.3 (68.7 - 79) | 74.6 (71.6 - 78.1) | 1 |
| % Time ΔCPPopt-PAx > 5 mmHg | 30.5 (22.5 - 35.9) | 19 (13.7 - 21.8) | 0.129 | 24.1 (15.2 - 35.3) | 22.2 (14.2 - 30.3) | 1 | 7.5 (5.9 - 21.3) | 17.7 (11.2 - 29.5) | 1 |
| % Time ΔCPPopt-PAx > 10 mmHg | 15.1 (8.9 - 22.2) | 6.38 (5.06 - 11.7) | 0.2284 | 12.3 (6.18 - 22.3) | 10.3 (4.2 - 16.1) | 0.9831 | 2.5 (2.33 - 12.6) | 7.73 (3.86 - 11.8) | 1 |
| % Time ΔCPPopt-PAx < -5 mmHg | 23.6 (15.1 - 28.7) | 27.8 (21.1 - 43.8) | 0.4241 | 34.6 (22.8 - 43.2) | 31 (21.1 - 37.1) | 0.9831 | 31.2 (22.3 - 50) | 32.6 (22.1 - 40.8) | 1 |
| % Time ΔCPPopt-PAx < -10 mmHg | 9.46 (5.77 - 12.7) | 9.97 (5.5 - 24.2) | 0.6105 | 17.4 (9.07 - 30.5) | 16.9 (9.58 - 29.3) | 1 | 14 (8.93 - 36) | 16.5 (12.2 - 23.8) | 1 |
| % Time CPP > ULR-PAx | 3.12 (2.72 - 3.96) | 6.22 (1.92 - 14.4) | 0.6434 | 7.2 (3.75 - 9.84) | 7.19 (3.35 - 16.6) | 1 | 1.61 (1.08 - 6.5) | 8.87 (4.97 - 15) | 0.9755 |
| % Time CPP < LLR-PAx | 3.68 (2.32 - 6.69) | 3.72 (2.08 - 38.2) | 0.6228 | 5.76 (2.11 - 10.3) | 9.91 (4.09 - 31) | 0.8184 | 10.1 (5.47 - 17.7) | 14.4 (4.78 - 22.8) | 1 |
| CPPopt-RAC | 70.5 (68.3 - 75.7) | 70.5 (64.5 - 76.1) | 0.6657 | 75 (72 - 77.7) | 74.7 (71.1 - 82.4) | 1 | 73.2 (70 - 80.1) | 72.5 (70.8 - 79.3) | 1 |
| % Time ΔCPPopt-RAC > 5 mmHg | 35 (27.1 - 42.7) | 24.9 (17.1 - 28.7) | 0.2284 | 24.9 (14.8 - 40.5) | 25.6 (17.1 - 33.9) | 1 | 6.63 (5.36 - 19.2) | 18.7 (14.4 - 32) | 0.9755 |
| % Time ΔCPPopt-RAC > 10 mmHg | 17.1 (10.5 - 26.8) | 12.5 (8.04 - 15.1) | 0.3818 | 11.7 (6.06 - 25.6) | 10.5 (6.85 - 17.7) | 0.9831 | 2.83 (2.44 - 11.9) | 9.14 (5.42 - 15.6) | 1 |
| % Time ΔCPPopt-RAC < -5 mmHg | 14 (8.19 - 22.4) | 22.6 (10.3 - 36.8) | 0.4745 | 27 (17.5 - 36.4) | 23.5 (14 - 35.2) | 1 | 35.9 (22.9 - 50.3) | 22.4 (18.2 - 36.8) | 1 |
| % Time ΔCPPopt-RAC < -10 mmHg | 5.04 (2.17 - 11.2) | 7.62 (3.26 - 17.4) | 0.3533 | 11.3 (3.98 - 23.2) | 13.3 (2.97 - 18.3) | 1 | 20.7 (11.3 - 34.2) | 13.5 (9.15 - 18.1) | 1 |
| % Time CPP > ULR-RAC | 3.28 (1.57 - 3.9) | 6.14 (1.75 - 10.9) | 0.3886 | 3.8 (1.66 - 7.23) | 3.31 (1.59 - 8.87) | 1 | 1.89 (1.07 - 5.02) | 6.18 (2.44 - 13.1) | 1 |
| % Time CPP < LLR-RAC | 3.73 (1.22 - 4.92) | 3.77 (1.71 - 28.3) | 0.5535 | 3.17 (0.867 - 7.07) | 3.82 (0.95 - 9.05) | 1 | 7.82 (4.42 - 23.3) | 6.57 (3.22 - 15.8) | 1 |

*All p-values have been adjusted using the False Discovery Rate (FDR) method. Bolded p-values are those reaching statistical significance, p < 0.05.*

*AMP = pulse amplitude of ICP, CPP = cerebral perfusion pressure, CPPopt = cerebral perfusion pressure optimum, ΔCPPopt = CPP - CPPopt, CT = computed tomography, GCS = Glasgow Coma Scale, GOSE = Glasgow Outcome Scale-Extended, ICP = intracranial pressure, ICU = intensive care unit, IQR = interquartile range, LLR = lower limit of reactivity, MAP = mean arterial pressure, mmHg = millimeters of mercury, PAx = pulse amplitude index, PRx = pressure reactivity index, RAC = correlation (R) between slow-waves of AMP (A) and CPP (C), SAH = subarachnoid hemorrhage, ULR = upper limit of reactivity.*

Appendix I. Mann-Whitney U/Chi-Square Testing of Physiologic and Demographic Data for Improved/Not Improved (3-6 Months) with Patients Trichotomized by Age

| **Variable** | **Age < 30 (n = 29)** | | | **Age 30 – 60 (n = 53)** | | | **Age > 60 (n = 21)** | | |
| --- | --- | --- | --- | --- | --- | --- | --- | --- | --- |
|  | **Improved**  **Median (IQR)** | **Not Improved**  **Median (IQR)** | **p-value** | **Improved**  **Median (IQR)** | **Not Improved**  **Median (IQR)** | **p-value** | **Improved**  **Median (IQR)** | **Not Improved**  **Median (IQR)** | **p-value** |
| Age (years) | 24 (21.5 - 26) | 23 (21 - 24.8) | 0.866 | 50 (41 - 51) | 43.5 (37.8 - 50.5) | 0.6976 | 62.5 (62.2 - 62.8) | 67 (63 - 72.5) | 0.8593 |
| Sex (% Male) | 100% | 72.20% | 0.5425 | 82.40% | 80.60% | 1 | 50% | 78.90% | 1 |
| Admission GCS Total | 6 (4.5 - 7) | 6 (4.25 - 7) | 1 | 8 (6 - 10) | 6 (4 - 8) | 0.4021 | 6.5 (5.25 - 7.75) | 7 (4.5 - 8.5) | 1 |
| Admission GCS Motor | 4 (2.5 - 5) | 4 (2.25 - 5) | 0.928 | 5 (3 - 5) | 4 (1.75 - 5) | 0.6359 | 3.5 (2.75 - 4.25) | 4 (2 - 5) | 1 |
| Admission GCS Eyes | 1 (1 - 1) | 1 (1 - 1) | 0.7369 | 1 (1 - 2) | 1 (1 - 2) | 0.6359 | 1.5 (1.25 - 1.75) | 2 (1 - 2.5) | 1 |
| Admission GCS Verbal | 1 (1 - 1) | 1 (1 - 1) | 0.7685 | 1 (1 - 2) | 1 (1 - 1) | 0.3644 | 1.5 (1.25 - 1.75) | 2 (1 - 2) | 1 |
| Admission Pupil Response  (% Bilaterally Reactive) | 54.50% | 61.10% | 0.9141 | 64.70% | 55.60% | 0.7952 | 100% | 63.20% | 1 |
| Marshall CT Grade | 3 (3 - 4.5) | 4 (3 - 5) | 0.6567 | 4 (3 - 5) | 4.5 (3 - 5) | 0.6359 | 4 (3.5 - 4.5) | 5 (4.5 - 5) | 0.8593 |
| Rotterdam CT Grade | 4 (3.5 - 4.5) | 4 (4 - 5.75) | 0.7685 | 4 (3 - 5) | 5 (3 - 6) | 0.6976 | 4 (4 - 4) | 5 (4 - 6) | 0.8593 |
| Helsinki CT Score | 4 (3 - 6) | 6 (5 - 9) | 0.552 | 5 (3 - 9) | 8 (4 - 9) | 0.4096 | 6 (5 - 7) | 7 (6 - 9) | 0.8593 |
| Stockholm CT Score | 2.9 (2.5 - 3.5) | 3.2 (2.62 - 3.5) | 0.8205 | 3 (2.3 - 3.7) | 3.26 (2.35 - 3.6) | 0.6888 | 3.5 (3.25 - 3.75) | 3.5 (2.9 - 4.15) | 1 |
| Number with Hypoxia Episode | 45.50% | 38.90% | 1 | 29.40% | 41.70% | 0.7952 | 50% | 15.80% | 1 |
| Number with Hypotension Episode | 18.20% | 16.70% | 1 | 11.80% | 5.60% | 0.8853 | 50% | 5.30% | 0.872 |
| Number with Traumatic SAH | 100% | 94.40% | 1 | 94.10% | 97.20% | 1 | 100% | 94.70% | 1 |
| Admission Hemoglobin | 140 (132 - 154) | 132 (114 - 148) | 0.5425 | 142 (115 - 154) | 130 (113 - 140) | 0.2615 | 114 (107 - 122) | 127 (113 - 138) | 0.872 |
| Admission Serum Glucose | 7.6 (6.4 - 8.75) | 9.7 (7.62 - 12.2) | 0.385 | 7.5 (6.2 - 9.4) | 7.8 (6.65 - 10.3) | 0.672 | 11.2 (9.52 - 13) | 8.2 (7.15 - 11.6) | 0.9542 |
| Length of Hospital Stay | 31 (21 - 66) | 38 (8 - 68) | 0.8687 | 31 (21 - 41) | 26 (9 - 45.5) | 0.7952 | 37 (34.5 - 39.5) | 8 (3 - 11.5) | 0.5754 |
| Length of ICU Stay | 12 (7.5 - 17.5) | 9 (5 - 22) | 0.928 | 7 (4 - 15) | 9.5 (4 - 13.5) | 0.7952 | 10.5 (9.25 - 11.8) | 5.5 (3 - 9.5) | 0.8593 |
| Mean MAP (mmHg) | 84.3 (81 - 94.3) | 85.1 (80.3 - 86.5) | 0.8942 | 86.3 (82.8 - 88.3) | 83.7 (79 - 90.4) | 0.6888 | 83.2 (81.8 - 84.7) | 79 (77.3 - 83.4) | 0.8593 |
| Mean ICP (mmHg) | 9.64 (6.93 - 15.1) | 11.9 (8.87 - 15.8) | 0.6579 | 8.55 (4.19 - 13.7) | 8.1 (2.89 - 11) | 0.7952 | 6.93 (6.44 - 7.41) | 8.17 (5.06 - 10.7) | 1 |
| % Time ICP > 20 mmHg | 6.04 (0.888 - 7.53) | 4.15 (0.624 - 16.1) | 1 | 1.66 (0 - 5.24) | 0.986 (0 - 2.76) | 0.8596 | 1.15 (0.582 - 1.72) | 0.226 (0.0544 - 1.46) | 1 |
| % Time ICP > 22 mmHg | 2.95 (0.569 - 5.26) | 1.66 (0.268 - 5.95) | 1 | 1.06 (0 - 2.57) | 0.552 (0 - 1.41) | 0.8845 | 0.796 (0.398 - 1.19) | 0.13 (0 - 0.874) | 1 |
| Mean CPP (mmHg) | 75.7 (69.5 - 80.2) | 69.9 (67.6 - 74.1) | 0.552 | 75.7 (73.6 - 83.3) | 74.9 (71.1 - 80.8) | 0.6976 | 76.1 (75.3 - 76.9) | 72.4 (69 - 74.2) | 0.8593 |
| % Time CPP < 60 mmHg | 4.04 (2.45 - 9.75) | 8.85 (3.31 - 15.6) | 0.552 | 3.95 (0.079 - 7.7) | 2.7 (0.607 - 7.14) | 0.8596 | 4.14 (2.79 - 5.48) | 3.43 (1.57 - 13.4) | 1 |
| % Time CPP > 70 mmHg | 59.1 (45.6 - 77.3) | 44.3 (33.5 - 61.8) | 0.4861 | 70.4 (57.7 - 76.1) | 68.4 (53.9 - 84.2) | 0.8757 | 66.3 (64.9 - 67.8) | 51.6 (42.3 - 68.8) | 0.9029 |
| Mean PRx | 0.0516 (-0.0417 - 0.124) | 0.143 (0.01 - 0.345) | 0.552 | 0.21 (0.12 - 0.4) | 0.151 (0.0566 - 0.272) | 0.4408 | 0.232 (0.12 - 0.343) | 0.126 (0.0609 - 0.238) | 1 |
| % Time PRx > 0 | 51.1 (41.8 - 65.5) | 65.5 (48.1 - 82.4) | 0.552 | 73.6 (68.1 - 87.2) | 64.4 (57.1 - 82.3) | 0.4716 | 70 (60.7 - 79.3) | 64 (57.3 - 76.8) | 1 |
| % Time PRx > 0.25 | 28.8 (18 - 36.1) | 40.1 (21.9 - 64.3) | 0.552 | 53.4 (38.6 - 70.4) | 40.3 (30 - 56.2) | 0.4221 | 51.6 (39.5 - 63.7) | 36.3 (29.4 - 54) | 1 |
| % Time PRx > 0.35 | 23.7 (13.2 - 25.4) | 29.7 (15.4 - 55.5) | 0.552 | 39.6 (27.4 - 62.3) | 30.2 (21.9 - 43) | 0.4021 | 43.7 (31.4 - 56) | 26.6 (20.8 - 43.4) | 1 |
| Mean PAx | -0.142 (-0.315 - -0.0492) | -0.0597 (-0.164 - 0.131) | 0.4861 | 0.097 (0.0175 - 0.211) | -0.0364 (-0.125 - 0.0625) | 0.0863 | 0.216 (0.14 - 0.293) | 0.0332 (-0.0674 - 0.184) | 0.8593 |
| % Time PAx > 0 | 29.6 (15.9 - 43.5) | 42.5 (29.9 - 68) | 0.4861 | 64.6 (53.8 - 73.3) | 46.8 (35.5 - 59.7) | 0.0863 | 73.1 (66.1 - 80) | 54.2 (40.1 - 69.8) | 0.8593 |
| % Time PAx > 0.25 | 9.9 (5.47 - 16.3) | 18.1 (11.5 - 36.7) | 0.385 | 34.1 (26.4 - 51) | 22.7 (14.9 - 30) | 0.0863 | 51.3 (43.6 - 59) | 27.2 (18 - 46.3) | 0.8593 |
| Mean RAC | -0.447 (-0.528 - -0.261) | -0.208 (-0.396 - -0.0476) | 0.385 | -0.134 (-0.274 - -0.0306) | -0.298 (-0.389 - -0.154) | 0.0863 | 0.127 (0.0574 - 0.197) | -0.0375 (-0.223 - 0.0383) | 0.8593 |
| % Time RAC > -0.10 | 13.6 (10.7 - 31.1) | 34.9 (18.4 - 58.1) | 0.385 | 46.4 (32.5 - 55.9) | 30 (18.1 - 45.8) | 0.0863 | 72.1 (65.9 - 78.3) | 55.9 (32.9 - 62.8) | 0.8593 |
| % Time RAC > -0.05 | 11.4 (8.53 - 26.6) | 29.8 (15.4 - 52.4) | 0.385 | 39.6 (28.7 - 49.6) | 25.6 (15 - 39) | 0.0863 | 68.5 (62.1 - 74.8) | 50.7 (26.6 - 58.1) | 0.8593 |
| CPPopt-PRx | 74.1 (68.6 - 85) | 70.6 (68.9 - 75.2) | 0.7712 | 77.3 (74.4 - 83.6) | 75.4 (69.1 - 79.5) | 0.4502 | 69.5 (68.9 - 70.1) | 72 (69 - 77.6) | 0.8593 |
| % Time ΔCPPopt-PRx > 5 mmHg | 28.3 (26.2 - 34.2) | 21.2 (10.2 - 28.6) | 0.385 | 26.7 (14.2 - 30.9) | 24 (18.4 - 34.4) | 0.8596 | 47 (45 - 48.9) | 21.1 (15.5 - 28.8) | 0.551 |
| % Time ΔCPPopt-PRx > 10 mmHg | 14.4 (11.1 - 18.9) | 8.58 (5.15 - 11.5) | 0.385 | 11 (4.74 - 17.3) | 10.9 (7.11 - 19.5) | 0.7952 | 30.9 (30.4 - 31.3) | 9.65 (5.43 - 14.9) | 0.5524 |
| % Time ΔCPPopt-PRx < -5 mmHg | 17.4 (10.9 - 23.9) | 20.3 (15.9 - 43) | 0.5425 | 32.1 (21.8 - 41.9) | 25.6 (18.3 - 37.7) | 0.6976 | 10.2 (8.73 - 11.6) | 22.2 (15.6 - 32.5) | 0.8593 |
| % Time ΔCPPopt-PRx < -10 mmHg | 6.12 (3.08 - 10.4) | 6.15 (4.97 - 22) | 0.552 | 16.9 (9.49 - 23.7) | 14 (5.25 - 20.1) | 0.6359 | 3.54 (3.06 - 4.02) | 9.82 (5.07 - 16.5) | 0.8593 |
| % Time CPP > ULR-PRx | 12.9 (5.8 - 15.5) | 12.3 (4.93 - 17.1) | 0.9141 | 18.2 (9.56 - 40.1) | 13.7 (8.4 - 24) | 0.4221 | 38.8 (26.9 - 50.8) | 14.1 (6.82 - 21.9) | 0.8593 |
| % Time CPP < LLR-PRx | 7.31 (4.41 - 9.8) | 8.59 (5.32 - 29.6) | 0.6802 | 31.1 (10.6 - 44.2) | 12.9 (4.72 - 25.2) | 0.4021 | 11 (5.98 - 16) | 14.2 (7.84 - 16.5) | 1 |
| CPPopt-PAx | 73.4 (70.2 - 83.8) | 71.2 (68 - 76.3) | 0.552 | 76.9 (73.6 - 83.8) | 76.8 (73.2 - 82.8) | 0.8893 | 74 (73.2 - 74.9) | 74.4 (71.4 - 80.4) | 1 |
| % Time ΔCPPopt-PAx > 5 mmHg | 28.2 (21.2 - 37) | 20 (13.7 - 29.7) | 0.5413 | 26.2 (19.3 - 37.7) | 22 (13.6 - 29.2) | 0.4408 | 34.9 (34.8 - 34.9) | 16.8 (10.6 - 22.4) | 0.8284 |
| % Time ΔCPPopt-PAx > 10 mmHg | 14 (8.87 - 24.8) | 8.59 (6.1 - 14.3) | 0.5425 | 12.9 (6.32 - 20.9) | 9.44 (5.35 - 16.5) | 0.6888 | 23 (22.8 - 23.1) | 6.78 (2.85 - 10.8) | 0.551 |
| % Time ΔCPPopt-PAx < -5 mmHg | 23.6 (18.1 - 26.4) | 28.2 (16.9 - 41.4) | 0.6567 | 21.1 (17.1 - 34.3) | 34.6 (26.5 - 43.2) | 0.2413 | 30.5 (30.1 - 30.9) | 35.1 (20.5 - 42.7) | 1 |
| % Time ΔCPPopt-PAx < -10 mmHg | 9.69 (6.14 - 12.2) | 9.94 (5.2 - 22.8) | 0.8687 | 13.1 (5.39 - 25.2) | 18.3 (11.8 - 30.8) | 0.4716 | 14.9 (14.4 - 15.3) | 17 (10.7 - 24.4) | 1 |
| % Time CPP > ULR-PAx | 2.85 (2.5 - 6.94) | 3.6 (2.46 - 11.4) | 0.866 | 10.8 (8.09 - 16.4) | 4.71 (2.4 - 9.55) | 0.0863 | 30.6 (21 - 40.2) | 6.28 (3.31 - 13.4) | 0.8593 |
| % Time CPP < LLR-PAx | 2.23 (1.43 - 6.1) | 4.01 (2.7 - 17.3) | 0.5425 | 7.28 (4.17 - 18.8) | 6.04 (2.12 - 14.8) | 0.6976 | 19.9 (15 - 24.8) | 14.2 (3.41 - 21.2) | 1 |
| CPPopt-RAC | 72 (65.2 - 83) | 69.9 (67.1 - 73.3) | 0.8179 | 75.9 (71.1 - 79.1) | 74.6 (72 - 79.3) | 0.9197 | 73.2 (73.2 - 73.2) | 71.7 (70.3 - 79.9) | 1 |
| % Time ΔCPPopt-RAC > 5 mmHg | 32.2 (25.3 - 48.9) | 27.7 (17.1 - 34.1) | 0.552 | 25.8 (17.6 - 43.7) | 25.3 (14.4 - 35.9) | 0.6359 | 36.2 (34 - 38.4) | 17.3 (12.8 - 26.3) | 0.8593 |
| % Time ΔCPPopt-RAC > 10 mmHg | 17.1 (9.22 - 34.6) | 14.2 (9.15 - 16.4) | 0.552 | 11.5 (8.62 - 24.2) | 10.2 (6.63 - 18.2) | 0.7952 | 24 (22.5 - 25.5) | 6.47 (3.99 - 12.9) | 0.5524 |
| % Time ΔCPPopt-RAC < -5 mmHg | 14 (8.07 - 21.9) | 21 (8.39 - 32.8) | 0.552 | 19.5 (15.9 - 35.2) | 26.1 (15.4 - 35.4) | 0.7952 | 27 (22.5 - 31.4) | 22.6 (17.1 - 39.3) | 1 |
| % Time ΔCPPopt-RAC < -10 mmHg | 5.97 (2.84 - 11.2) | 6.5 (2.55 - 16.3) | 0.8725 | 9.55 (2.59 - 22.8) | 12.7 (4.07 - 18.3) | 0.9197 | 15.5 (12.9 - 18.1) | 13.9 (8.35 - 19.9) | 1 |
| % Time CPP > ULR-RAC | 3.33 (1.21 - 6.47) | 3.48 (1.78 - 6.91) | 0.8725 | 7.25 (3.92 - 12.5) | 2.7 (1.14 - 4.6) | 0.0863 | 25.4 (16.8 - 34) | 4.6 (1.81 - 12) | 0.8593 |
| % Time CPP < LLR-RAC | 1.58 (0.934 - 3.92) | 5.22 (2.12 - 12.3) | 0.385 | 6.96 (3.25 - 14.3) | 2.14 (0.679 - 4.86) | 0.1328 | 14.9 (11.3 - 18.4) | 6.39 (3.01 - 15.5) | 0.8593 |

*All p-values have been adjusted using the False Discovery Rate (FDR) method. Bolded p-values are those reaching statistical significance, p < 0.05.*

*AMP = pulse amplitude of ICP, CPP = cerebral perfusion pressure, CPPopt = cerebral perfusion pressure optimum, ΔCPPopt = CPP - CPPopt, CT = computed tomography, GCS = Glasgow Coma Scale, GOSE = Glasgow Outcome Scale-Extended, ICP = intracranial pressure, ICU = intensive care unit, IQR = interquartile range, LLR = lower limit of reactivity, MAP = mean arterial pressure, mmHg = millimeters of mercury, PAx = pulse amplitude index, PRx = pressure reactivity index, RAC = correlation (R) between slow-waves of AMP (A) and CPP (C), SAH = subarachnoid hemorrhage, ULR = upper limit of reactivity.*

Appendix J. Mann-Whitney U/Chi-Square Testing of Physiologic and Demographic Data for Improved/Not Improved (1-6 Months) with Patients Trichotomized by Age

| **Variable** | **Age < 30 (n = 29)** | | | **Age 30 – 60 (n = 53)** | | | **Age > 60 (n = 21)** | | |
| --- | --- | --- | --- | --- | --- | --- | --- | --- | --- |
|  | **Improved**  **Median (IQR)** | **Not Improved**  **Median (IQR)** | **p-value** | **Improved**  **Median (IQR)** | **Not Improved**  **Median (IQR)** | **p-value** | **Improved**  **Median (IQR)** | **Not Improved**  **Median (IQR)** | **p-value** |
| Age (years) | 22 (20.5 - 24.5) | 23 (22.2 - 25.8) | 0.555 | 42 (36.2 - 50) | 47 (42 - 53.5) | 0.8136 | 62.5 (61.8 - 63) | 67 (66 - 73) | 0.4466 |
| Sex (% Male) | 84.20% | 80% | 1 | 86.70% | 73.90% | 0.8136 | 75% | 76.50% | 1 |
| Admission GCS Total | 6 (5 - 7) | 6 (4 - 6) | 0.5192 | 7 (5.25 - 8) | 6 (4 - 8) | 0.8136 | 8 (6.25 - 9.25) | 7 (4 - 8) | 1 |
| Admission GCS Motor | 4 (3 - 5) | 4 (2 - 4) | 0.555 | 4.5 (3 - 5) | 4 (1.5 - 5) | 0.8805 | 4.5 (3.5 - 5) | 4 (2 - 5) | 1 |
| Admission GCS Eyes | 1 (1 - 1) | 1 (1 - 1) | 0.712 | 1 (1 - 2) | 1 (1 - 1.5) | 0.8136 | 1.5 (1 - 2.25) | 2 (1 - 2) | 1 |
| Admission GCS Verbal | 1 (1 - 1) | 1 (1 - 1) | 0.3375 | 1 (1 - 2) | 1 (1 - 1.5) | 0.8136 | 2 (1.75 - 2) | 1 (1 - 2) | 1 |
| Admission Pupil Response  (% Bilaterally Reactive) | 63.20% | 50% | 0.7749 | 56.70% | 60.90% | 0.8136 | 100% | 58.80% | 1 |
| Marshall CT Grade | 3 (3 - 4.5) | 5 (4 - 5) | 0.0524 | 5 (3 - 5) | 4 (3 - 5) | 0.8136 | 5 (4.5 - 5) | 5 (4 - 5) | 1 |
| Rotterdam CT Grade | 4 (3 - 4.5) | 5 (4 - 6) | 0.1898 | 5 (4 - 5.75) | 4 (3 - 5) | 0.8136 | 4.5 (4 - 5.25) | 5 (4 - 6) | 1 |
| Helsinki CT Score | 5 (3 - 6) | 7.5 (5.25 - 10.5) | 0.2552 | 8 (4.25 - 9) | 5 (4 - 8.5) | 0.8136 | 6 (4.75 - 7.25) | 8 (6 - 9) | 1 |
| Stockholm CT Score | 3.1 (2.5 - 3.5) | 3.18 (2.5 - 3.5) | 0.9596 | 3.2 (2.9 - 3.88) | 3 (2.15 - 3.5) | 0.8136 | 3.05 (3 - 3.32) | 3.6 (2.8 - 4.3) | 1 |
| Number with Hypoxia Episode | 42.10% | 40% | 1 | 36.70% | 39.10% | 1 | 25% | 17.60% | 1 |
| Number with Hypotension Episode | 26.30% | 0% | 0.3414 | 10% | 4.30% | 0.9155 | 25% | 5.90% | 1 |
| Number with Traumatic SAH | 100% | 90% | 0.8416 | 96.70% | 95.70% | 1 | 100% | 94.10% | 1 |
| Admission Hemoglobin | 140 (124 - 150) | 131 (124 - 139) | 0.5607 | 138 (118 - 147) | 129 (108 - 140) | 0.8136 | 122 (110 - 134) | 127 (113 - 136) | 1 |
| Admission Serum Glucose | 8.2 (7.3 - 10.4) | 9.5 (7.35 - 13.7) | 0.5064 | 7.8 (6.6 - 9.57) | 7.7 (6.4 - 10) | 1 | 10.8 (8.25 - 13.5) | 7.9 (7.1 - 11.1) | 1 |
| Length of Hospital Stay | 59 (29 - 82.5) | 8 (6 - 11) | **0.026** | 32 (21 - 46) | 19 (8.5 - 39.5) | 0.8136 | 37 (26.5 - 45) | 7.5 (3 - 10.5) | 0.3422 |
| Length of ICU Stay | 14 (9 - 24) | 5 (4 - 7) | **0.0321** | 7.5 (3.25 - 15) | 7 (4.5 - 13) | 0.8805 | 10.5 (6.5 - 15.8) | 5.5 (3 - 8.5) | 1 |
| Mean MAP (mmHg) | 84.9 (81.3 - 90.3) | 83.9 (79.8 - 88.9) | 0.8577 | 85.3 (82 - 91.3) | 83.5 (78.4 - 89.2) | 0.8136 | 81.4 (77.8 - 83.3) | 79 (77.6 - 84.4) | 1 |
| Mean ICP (mmHg) | 10.9 (8.23 - 14.7) | 13.4 (9.32 - 20.5) | 0.3673 | 9.11 (3.18 - 12) | 7.67 (4.72 - 11.5) | 0.9155 | 6.93 (4.11 - 7.97) | 9.01 (5.34 - 10.9) | 1 |
| % Time ICP > 20 mmHg | 4.99 (0.223 - 6.67) | 6.88 (1.6 - 41.4) | 0.3414 | 0.734 (0 - 4.73) | 1.12 (0.0721 - 3.42) | 0.8136 | 0.0619 (0.0112 - 0.653) | 0.351 (0.136 - 1.75) | 1 |
| % Time ICP > 22 mmHg | 2.14 (0.0723 - 4.05) | 4.38 (0.739 - 34.9) | 0.4076 | 0.31 (0 - 2.34) | 0.605 (0.0524 - 1.81) | 0.8136 | 0 (0 - 0.398) | 0.211 (0.0857 - 0.947) | 1 |
| Mean CPP (mmHg) | 73 (68.9 - 79.1) | 67.7 (65.3 - 72.8) | 0.1036 | 76.4 (72.1 - 83.1) | 74.7 (70.9 - 78.7) | 0.8136 | 74.4 (73.8 - 75.3) | 72.4 (68.3 - 73.9) | 1 |
| % Time CPP < 60 mmHg | 4.04 (3.08 - 9.75) | 15.5 (8.76 - 28.4) | 0.1036 | 2.37 (0.349 - 7.64) | 3.95 (0.587 - 6.81) | 0.9155 | 3.67 (1.45 - 6.12) | 3.43 (1.69 - 15.8) | 1 |
| % Time CPP > 70 mmHg | 59.1 (44.6 - 70.2) | 34.5 (28.5 - 50.5) | **0.0323** | 71.2 (58.5 - 87.9) | 64.6 (50.5 - 82.4) | 0.8136 | 60.2 (55.7 - 64.9) | 51.4 (40.7 - 69.4) | 1 |
| Mean PRx | 0.0376 (-0.0944 - 0.121) | 0.204 (0.144 - 0.444) | **0.0321** | 0.166 (0.0729 - 0.261) | 0.244 (0.0622 - 0.355) | 0.8136 | 0.186 (0.0378 - 0.357) | 0.126 (0.074 - 0.2) | 1 |
| % Time PRx > 0 | 51.1 (37.1 - 64.5) | 68.2 (65.4 - 87.4) | **0.0372** | 69.1 (58 - 82.3) | 76.4 (56.8 - 88) | 0.8136 | 67.4 (53.4 - 82.7) | 64 (58.9 - 72.8) | 1 |
| % Time PRx > 0.25 | 26.3 (17.4 - 36.1) | 44.2 (39.5 - 73) | **0.0321** | 42.1 (32.6 - 56) | 49.6 (32.5 - 67.3) | 0.8805 | 45.7 (27.6 - 66.7) | 36.3 (31.1 - 50.2) | 1 |
| % Time PRx > 0.35 | 18.9 (11.7 - 25.4) | 35.1 (29.5 - 65.8) | **0.028** | 32.7 (24.5 - 40.7) | 37.8 (23.5 - 54.7) | 0.8685 | 36 (19.1 - 56.7) | 26.6 (22.6 - 41.7) | 1 |
| Mean PAx | -0.131 (-0.268 - -0.0492) | 0.0656 (-0.11 - 0.309) | 0.0524 | 0.0201 (-0.0613 - 0.0818) | 0.0346 (-0.104 - 0.22) | 0.8136 | 0.048 (0.00279 - 0.14) | 0.0525 (-0.0465 - 0.186) | 1 |
| % Time PAx > 0 | 34.2 (19.8 - 42.8) | 55.6 (37.1 - 80.4) | 0.0511 | 53 (41.2 - 63.2) | 53.9 (38.9 - 79.2) | 0.8136 | 56.1 (48.9 - 66.1) | 57 (44 - 70.9) | 1 |
| % Time PAx > 0.25 | 11.9 (6.82 - 17.7) | 31.7 (14 - 60.1) | **0.0358** | 26.2 (16.3 - 32.6) | 29.3 (17 - 49.3) | 0.8136 | 35.8 (29.9 - 43.6) | 27.2 (18.8 - 46.4) | 1 |
| Mean RAC | -0.406 (-0.528 - -0.234) | -0.109 (-0.306 - 0.0747) | **0.0321** | -0.274 (-0.383 - -0.139) | -0.227 (-0.322 - -0.0559) | 0.8136 | 0.00186 (-0.054 - 0.0785) | -0.0375 (-0.267 - 0.0607) | 1 |
| % Time RAC > -0.10 | 18.4 (10.7 - 32.2) | 45.9 (26 - 67.4) | **0.0329** | 31 (21.5 - 46.3) | 34.4 (27.4 - 53.2) | 0.8136 | 59.6 (54 - 65.9) | 55.9 (28 - 63.8) | 1 |
| % Time RAC > -0.05 | 16.1 (8.53 - 27.5) | 41.8 (22.6 - 63.9) | **0.0329** | 27.1 (19 - 40.1) | 30.3 (23.8 - 47.7) | 0.8136 | 55.6 (49.1 - 62.1) | 50.7 (23.3 - 59.2) | 1 |
| CPPopt-PRx | 71.9 (68.5 - 80.1) | 70.6 (69.7 - 80) | 1 | 76.4 (72.1 - 83.6) | 75.4 (69 - 79.2) | 0.8136 | 68.7 (67.7 - 69.5) | 73 (69.4 - 77.9) | 0.6009 |
| % Time ΔCPPopt-PRx > 5 mmHg | 28.3 (23.2 - 34.6) | 13.2 (8.3 - 23.9) | **0.0321** | 27.3 (17.4 - 32.5) | 23.7 (15 - 32.1) | 0.8136 | 29.9 (15.4 - 45) | 21.4 (18.3 - 29.3) | 1 |
| % Time ΔCPPopt-PRx > 10 mmHg | 12.2 (10.2 - 19.5) | 5.69 (1.09 - 9.56) | **0.028** | 12.8 (7.99 - 20.9) | 8.6 (6.72 - 17.4) | 0.8136 | 19.8 (8.15 - 30.4) | 10.5 (5.47 - 16) | 1 |
| % Time ΔCPPopt-PRx < -5 mmHg | 17.4 (14.9 - 28.5) | 20.3 (17.9 - 49.1) | 0.2582 | 27.6 (17.1 - 41.5) | 26.9 (20.9 - 36.4) | 0.9452 | 7.88 (6.85 - 9.6) | 27.2 (19.5 - 34.8) | 0.1357 |
| % Time ΔCPPopt-PRx < -10 mmHg | 6.37 (4.77 - 12.2) | 5.63 (4.16 - 28.9) | 0.7685 | 14.3 (6.23 - 22.2) | 14.2 (6.83 - 21.1) | 0.9797 | 3.49 (2.28 - 4.43) | 10.8 (7.48 - 18.4) | 0.3422 |
| % Time CPP > ULR-PRx | 10.9 (5.19 - 14.8) | 15.5 (10.1 - 17.8) | 0.4908 | 15.6 (8.76 - 26.1) | 12.5 (7.89 - 25.4) | 0.8685 | 18.7 (12 - 32.5) | 14.1 (7.51 - 21.3) | 1 |
| % Time CPP < LLR-PRx | 7.31 (4.41 - 10.5) | 10 (5.93 - 41.9) | 0.3244 | 13.3 (7.11 - 31.5) | 18 (7.13 - 35.7) | 0.9155 | 9.36 (2.17 - 17.4) | 14.2 (9.37 - 16.9) | 1 |
| CPPopt-PAx | 72.5 (68.8 - 79.1) | 71 (67.7 - 78.8) | 0.575 | 76.8 (73.6 - 83.6) | 76.9 (73 - 81.2) | 0.8136 | 74 (70.5 - 78.2) | 74.4 (71.5 - 78.2) | 1 |
| % Time ΔCPPopt-PAx > 5 mmHg | 28.5 (22.5 - 35.9) | 16.1 (13.1 - 19) | **0.0179** | 24.1 (15.7 - 35.7) | 22.2 (13.7 - 29.6) | 0.8136 | 21.1 (6.7 - 34.8) | 17.2 (10.9 - 26.4) | 1 |
| % Time ΔCPPopt-PAx > 10 mmHg | 15.1 (8.9 - 22.2) | 6.17 (4.65 - 6.39) | **0.0265** | 12.3 (5.9 - 21.8) | 9.47 (4.2 - 15.8) | 0.8136 | 12.6 (2.41 - 22.8) | 7.45 (3.75 - 10.9) | 1 |
| % Time ΔCPPopt-PAx < -5 mmHg | 22.1 (14.4 - 28.3) | 39.3 (25.6 - 46.1) | 0.0524 | 34.4 (18.5 - 42.5) | 31 (24 - 36.8) | 0.9324 | 30.5 (25.6 - 40.6) | 35.1 (21.7 - 41) | 1 |
| % Time ΔCPPopt-PAx < -10 mmHg | 9.34 (5.03 - 12.2) | 16.9 (9.89 - 26.1) | 0.0785 | 17 (6.55 - 30) | 16.9 (12.9 - 25.9) | 0.9191 | 14.9 (11.4 - 26.4) | 17 (11.5 - 24.4) | 1 |
| % Time CPP > ULR-PAx | 3.12 (2.5 - 4.92) | 8.56 (2.39 - 14.4) | 0.5064 | 8.39 (4.44 - 11.8) | 4.46 (2.76 - 14.9) | 0.8136 | 6.5 (1.35 - 21) | 7.22 (4.92 - 14.8) | 1 |
| % Time CPP < LLR-PAx | 2.91 (1.91 - 6.04) | 16.2 (3.57 - 45.8) | **0.0409** | 5.76 (2.32 - 10.4) | 11.1 (3.79 - 23.9) | 0.8136 | 17.7 (7.79 - 26.4) | 14.2 (3.78 - 17.9) | 1 |
| CPPopt-RAC | 70.5 (66.8 - 77.6) | 70.5 (64.9 - 75.5) | 0.7749 | 75.7 (72.3 - 80.4) | 74.2 (70.1 - 78.2) | 0.8136 | 73.2 (71.6 - 76.7) | 71.7 (70.8 - 79.9) | 1 |
| % Time ΔCPPopt-RAC > 5 mmHg | 35 (26.4 - 42.7) | 23.7 (14.7 - 27.2) | **0.0321** | 25.5 (17.2 - 42) | 25.6 (14.4 - 33.6) | 0.8136 | 19.2 (6 - 34) | 18.3 (14.1 - 28) | 1 |
| % Time ΔCPPopt-RAC > 10 mmHg | 17.1 (10.5 - 30.1) | 10.3 (6.53 - 14.5) | 0.0559 | 11.4 (6.77 - 25.4) | 9.83 (6.8 - 17.4) | 0.8136 | 11.9 (2.63 - 22.5) | 8.83 (5.18 - 14.3) | 1 |
| % Time ΔCPPopt-RAC < -5 mmHg | 11.8 (7.56 - 21.9) | 28.9 (21 - 42.6) | **0.0321** | 22.3 (16.3 - 34.6) | 26.6 (14.9 - 35.5) | 0.9155 | 27 (16 - 43.1) | 22.6 (18.7 - 37.4) | 1 |
| % Time ΔCPPopt-RAC < -10 mmHg | 2.96 (2.17 - 8.44) | 11.3 (7.37 - 18.5) | **0.0321** | 9.98 (3.05 - 21.7) | 13.4 (4.86 - 19.9) | 0.8805 | 15.5 (8.27 - 27.4) | 13.9 (9.02 - 18.3) | 1 |
| % Time CPP > ULR-RAC | 3.28 (1.57 - 4.63) | 6.14 (1.75 - 10.8) | 0.4365 | 4.68 (1.89 - 8.52) | 2.95 (1.16 - 4.34) | 0.8136 | 5.02 (1.48 - 16.8) | 6.04 (2.28 - 13.1) | 1 |
| % Time CPP < LLR-RAC | 2.28 (1.06 - 4.48) | 9.96 (2.94 - 34.1) | **0.0372** | 4.23 (0.916 - 8.46) | 2.22 (0.885 - 7.19) | 0.8227 | 14.9 (6.12 - 26.1) | 6.39 (3.19 - 15.1) | 1 |

*All p-values have been adjusted using the False Discovery Rate (FDR) method. Bolded p-values are those reaching statistical significance, p < 0.05.*

*AMP = pulse amplitude of ICP, CPP = cerebral perfusion pressure, CPPopt = cerebral perfusion pressure optimum, ΔCPPopt = CPP - CPPopt, CT = computed tomography, GCS = Glasgow Coma Scale, GOSE = Glasgow Outcome Scale-Extended, ICP = intracranial pressure, ICU = intensive care unit, IQR = interquartile range, LLR = lower limit of reactivity, MAP = mean arterial pressure, mmHg = millimeters of mercury, PAx = pulse amplitude index, PRx = pressure reactivity index, RAC = correlation (R) between slow-waves of AMP (A) and CPP (C), SAH = subarachnoid hemorrhage, ULR = upper limit of reactivity.*

Appendix K. Histograms Comparing Improved and Not Improved Patients for % time with ΔCPPopt above/below 10mmHg


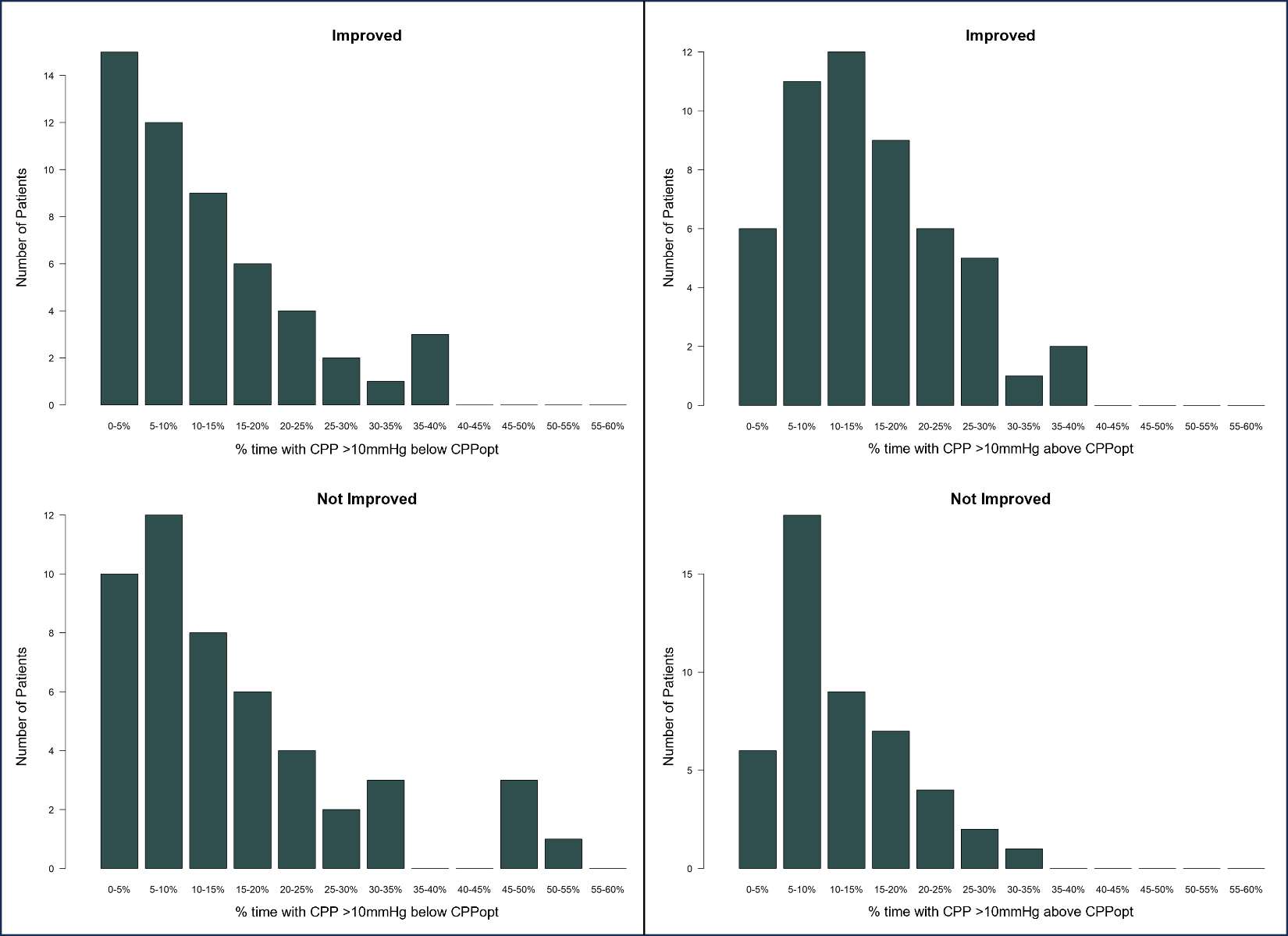


*Left panel illustrates the distributions of Improved (top) vs Not Improved (bottom) patients for % time with ΔCPPopt below 10mmHg. Right panel illustrates the distributions of Improved (top) vs Not Improved (bottom) patients for % time with ΔCPPopt above 10mmHg.*

*CPPopt = cerebral perfusion pressure optimum.*

Appendix L. Univariate Models of Cerebrovascular Reactivity Measures for Transition in Outcome with Those Who Died (GOSE=1) Removed

| **1 Month 🡪 3 Month** | | | | |
| --- | --- | --- | --- | --- |
| **Model** | **AUC (95% CI)** | **AIC** | **p-value** | **Nagelkerke's R^2^** |
| CPPopt-PRx | 0.559 (0.421 - 0.697) | 96.2 | 0.5463 | 0.003 |
| % Time ΔCPPopt-PRx > 5 mmHg | 0.504 (0.355 - 0.645) | 96.3 | 0.5906 | 0.001 |
| % Time ΔCPPopt-PRx > 10 mmHg | 0.578 (0.434 - 0.717) | 95.9 | 1.0000 | 0.010 |
| % Time ΔCPPopt-PRx < -5 mmHg | 0.516 (0.372 - 0.657) | 96.3 | 0.5452 | 0 |
| % Time ΔCPPopt-PRx < -10 mmHg | 0.522 (0.383 - 0.66) | 96.3 | 0.5736 | 0 |
| % Time CPP > ULR-PRx | 0.571 (0.423 - 0.71) | 93.4 | 0.6943 | 0.057 |
| % Time CPP < LLR-PRx | 0.549 (0.399 - 0.685) | 95.6 | 0.4750 | 0.015 |
| CPPopt-PAx | 0.528 (0.386 - 0.664) | 96.4 | 0.5657 | 0 |
| % Time ΔCPPopt-PAx > 5 mmHg | 0.545 (0.4 - 0.677) | 95.9 | 0.4701 | 0.010 |
| % Time ΔCPPopt-PAx > 10 mmHg | 0.558 (0.423 - 0.698) | 95.8 | 0.4937 | 0.011 |
| % Time ΔCPPopt-PAx < -5 mmHg | 0.577 (0.44 - 0.714) | 95.4 | 1.0000 | 0.019 |
| % Time ΔCPPopt-PAx < -10 mmHg | 0.521 (0.331 - 0.623) | 95.9 | 0.5419 | 0.009 |
| % Time CPP > ULR-PAx | 0.589 (0.431 - 0.732) | 90.4 | 1.0000 | 0.112 |
| % Time CPP < LLR-PAx | 0.563 (0.301 - 0.583) | 93.3 | 0.5727 | 0.059 |
| CPPopt-RAC | 0.489 (0.363 - 0.66) | 96.4 | 0.5938 | 0 |
| % Time ΔCPPopt-RAC > 5 mmHg | 0.486 (0.377 - 0.661) | 96.4 | 0.5798 | 0 |
| % Time ΔCPPopt-RAC > 10 mmHg | 0.552 (0.424 - 0.689) | 95.4 | 0.4983 | 0.018 |
| % Time ΔCPPopt-RAC < -5 mmHg | 0.565 (0.423 - 0.701) | 95.6 | 0.6451 | 0.016 |
| % Time ΔCPPopt-RAC < -10 mmHg | 0.502 (0.357 - 0.638) | 96.3 | 0.5748 | 0.001 |
| % Time CPP > ULR-RAC | 0.571 (0.422 - 0.711) | 93.2 | 0.8521 | 0.061 |
| % Time CPP < LLR-RAC | 0.501 (0.356 - 0.647) | 96.0 | 0.5472 | 0.008 |
| **3 Month 🡪 6 Month** | | | | |
| **Model** | **AUC (95% CI)** | **AIC** | **p-value** | **Nagelkerke's R^2^** |
| CPPopt-PRx | 0.602 (0.462 - 0.736) | 93.8 | 0.2061 | 0.047 |
| % Time ΔCPPopt-PRx > 5 mmHg | 0.586 (0.447 - 0.724) | 95.1 | 0.2417 | 0.020 |
| % Time ΔCPPopt-PRx > 10 mmHg | 0.58 (0.441 - 0.723) | 94.9 | 0.2152 | 0.025 |
| % Time ΔCPPopt-PRx < -5 mmHg | 0.533 (0.395 - 0.67) | 95.8 | 0.3777 | 0.006 |
| % Time ΔCPPopt-PRx < -10 mmHg | 0.516 (0.346 - 0.63) | 96.1 | 0.4133 | 0 |
| % Time CPP > ULR-PRx | 0.648 (0.506 - 0.776) | 89.0 | 0.1351 | 0.135 |
| % Time CPP < LLR-PRx | 0.552 (0.409 - 0.691) | 95.3 | 0.3091 | 0.018 |
| CPPopt-PAx | 0.559 (0.416 - 0.699) | 94.9 | 0.2880 | 0.025 |
| % Time ΔCPPopt-PAx > 5 mmHg | 0.625 (0.49 - 0.761) | 93.3 | 0.1697 | 0.056 |
| % Time ΔCPPopt-PAx > 10 mmHg | 0.602 (0.457 - 0.744) | 93.3 | 0.1832 | 0.055 |
| % Time ΔCPPopt-PAx < -5 mmHg | 0.614 (0.476 - 0.746) | 93.8 | 0.1995 | 0.046 |
| % Time ΔCPPopt-PAx < -10 mmHg | 0.572 (0.438 - 0.703) | 94.8 | 0.2388 | 0.026 |
| % Time CPP > ULR-PAx | 0.718 (0.586 - 0.839) | 85.1 | **0.0210** | 0.203 |
| % Time CPP < LLR-PAx | 0.582 (0.441 - 0.713) | 94.8 | 0.2440 | 0.026 |
| CPPopt-RAC | 0.549 (0.4 - 0.682) | 95.1 | 0.3103 | 0.021 |
| % Time ΔCPPopt-RAC > 5 mmHg | 0.605 (0.461 - 0.737) | 93.8 | 0.2139 | 0.045 |
| % Time ΔCPPopt-RAC > 10 mmHg | 0.581 (0.442 - 0.713) | 94.8 | 0.2284 | 0.026 |
| % Time ΔCPPopt-RAC < -5 mmHg | 0.531 (0.378 - 0.672) | 95.8 | 0.3729 | 0.007 |
| % Time ΔCPPopt-RAC < -10 mmHg | 0.524 (0.377 - 0.67) | 96.1 | 0.3885 | 0 |
| % Time CPP > ULR-RAC | 0.694 (0.568 - 0.824) | 87.4 | **0.0326** | 0.164 |
| % Time CPP < LLR-RAC | 0.64 (0.499 - 0.768) | 92.5 | 0.1349 | 0.072 |
| **1 Month 🡪 6 Month** | | | | |
| **Model** | **AUC (95% CI)** | **AIC** | **p-value** | **Nagelkerke's R^2^** |
| CPPopt-PRx | 0.483 (0.362 - 0.671) | 78.7 | 0.5817 | 0.001 |
| % Time ΔCPPopt-PRx > 5 mmHg | 0.63 (0.467 - 0.784) | 76.5 | 0.4179 | 0.048 |
| % Time ΔCPPopt-PRx > 10 mmHg | 0.669 (0.505 - 0.816) | 75.7 | 0.4494 | 0.065 |
| % Time ΔCPPopt-PRx < -5 mmHg | 0.617 (0.482 - 0.755) | 77.2 | 0.3389 | 0.033 |
| % Time ΔCPPopt-PRx < -10 mmHg | 0.611 (0.459 - 0.758) | 77.6 | 0.2757 | 0.025 |
| % Time CPP > ULR-PRx | 0.529 (0.373 - 0.685) | 78.3 | 0.4494 | 0.009 |
| % Time CPP < LLR-PRx | 0.578 (0.407 - 0.745) | 77.3 | 0.3358 | 0.030 |
| CPPopt-PAx | 0.524 (0.357 - 0.684) | 78.3 | 0.4319 | 0.010 |
| % Time ΔCPPopt-PAx > 5 mmHg | 0.617 (0.458 - 0.758) | 76.6 | 0.2825 | 0.047 |
| % Time ΔCPPopt-PAx > 10 mmHg | 0.627 (0.483 - 0.764) | 75.5 | 0.3318 | 0.068 |
| % Time ΔCPPopt-PAx < -5 mmHg | 0.52 (0.368 - 0.672) | 78.7 | 0.4277 | 0 |
| % Time ΔCPPopt-PAx < -10 mmHg | 0.566 (0.421 - 0.716) | 78.7 | 0.3473 | 0 |
| % Time CPP > ULR-PAx | 0.54 (0.389 - 0.697) | 78.7 | 0.4159 | 0.002 |
| % Time CPP < LLR-PAx | 0.584 (0.419 - 0.738) | 78.0 | 0.3320 | 0.016 |
| CPPopt-RAC | 0.527 (0.357 - 0.702) | 78.5 | 0.4369 | 0.005 |
| % Time ΔCPPopt-RAC > 5 mmHg | 0.6 (0.448 - 0.755) | 77.3 | 0.3014 | 0.031 |
| % Time ΔCPPopt-RAC > 10 mmHg | 0.644 (0.505 - 0.778) | 74.9 | 0.4410 | 0.081 |
| % Time ΔCPPopt-RAC < -5 mmHg | 0.565 (0.408 - 0.724) | 78.2 | 0.3288 | 0.012 |
| % Time ΔCPPopt-RAC < -10 mmHg | 0.596 (0.432 - 0.749) | 78.1 | 0.2945 | 0.014 |
| % Time CPP > ULR-RAC | 0.571 (0.408 - 0.728) | 77.4 | 0.3479 | 0.029 |
| % Time CPP < LLR-RAC | 0.558 (0.399 - 0.709) | 78.3 | 0.3433 | 0.010 |

*All p-values have been adjusted using the False Discovery Rate (FDR) method. Bolded p-values are those reaching statistical significance, p < 0.05.*

*AIC = Akaike information criterion, AMP = pulse amplitude of ICP, AUC = area under the curve, CI = confidence interval, CPP = cerebral perfusion pressure, CPPopt = cerebral perfusion pressure optimum, ΔCPPopt = CPP - CPPopt, GOSE = Glasgow Outcome Scale-Extended, ICP = intracranial pressure, LLR = lower limit of reactivity, mmHg = millimeters of mercury, PAx = pulse amplitude index, PRx = pressure reactivity index, RAC = correlation (R) between slow-waves of AMP (A) and CPP (C), ULR = upper limit of reactivity.*

Appendix M. Univariate Models of Cerebrovascular Reactivity Measures for Transition in Outcome with Patients Trichotomized by Age

| **1 Month 🡪 3 Month** | | | | | | | | | | | | |
| --- | --- | --- | --- | --- | --- | --- | --- | --- | --- | --- | --- | --- |
|  | **Age < 30** | | | | **Age 30 – 60** | | | | **Age > 60** | | | |
| **Model** | **AUC (95% CI)** | **AIC** | **p-value** | **Nagelkerke's R^2^** | **AUC (95% CI)** | **AIC** | **p-value** | **Nagelkerke's R^2^** | **AUC (95% CI)** | **AIC** | **p-value** | **Nagelkerke's R^2^** |
| CPPopt-PRx | 0.51 (0.3 - 0.724) | 44.1 | 0.5242 | 0.002 | 0.487 (0.359 - 0.674) | 76.9 | 0.5951 | 0.004 | 0.889 (0.722 - 1) | 17.7 | 0.1211 | 0.273 |
| % Time ΔCPPopt-PRx > 5 mmHg | 0.652 (0.433 - 0.833) | 41.6 | 0.2170 | 0.113 | 0.523 (0.365 - 0.677) | 76.7 | 0.5877 | 0.008 | 0.444 (0 - 0.944) | 21.2 | 0.6616 | 0.003 |
| % Time ΔCPPopt-PRx > 10 mmHg | 0.681 (0.471 - 0.871) | 41.7 | 0.2150 | 0.11 | 0.589 (0.438 - 0.739) | 75.6 | 0.5786 | 0.036 | 0.481 (0.056 - 0.944) | 21.1 | 0.6150 | 0.011 |
| % Time ΔCPPopt-PRx < -5 mmHg | 0.476 (0.305 - 0.757) | 43.8 | 0.6231 | 0.018 | 0.539 (0.389 - 0.694) | 76.8 | 0.5786 | 0.005 | 0.963 (0.852 - 1) | 10.5 | 0.0630 | 0.716 |
| % Time ΔCPPopt-PRx < -10 mmHg | 0.405 (0.371 - 0.814) | 43.4 | 0.8117 | 0.037 | 0.549 (0.395 - 0.708) | 76.8 | 0.5786 | 0.006 | 0.907 (0.759 - 1) | 14.2 | 0.1211 | 0.506 |
| % Time CPP > ULR-PRx | 0.69 (0.481 - 0.891) | 42.7 | 0.2150 | 0.067 | 0.501 (0.343 - 0.661) | 76.8 | 0.5951 | 0.004 | 0.537 (0.222 - 0.852) | 21 | 0.5614 | 0.018 |
| % Time CPP < LLR-PRx | 0.524 (0.29 - 0.752) | 42.6 | 0.4939 | 0.069 | 0.54 (0.382 - 0.698) | 76.7 | 0.5786 | 0.009 | 0.722 (0.333 - 1) | 18.6 | 0.3924 | 0.208 |
| CPPopt-PAx | 0.571 (0.219 - 0.657) | 43.8 | 0.3290 | 0.018 | 0.474 (0.375 - 0.687) | 77 | 0.6285 | 0.001 | 0.611 (0.204 - 1) | 21 | 0.5250 | 0.02 |
| % Time ΔCPPopt-PAx > 5 mmHg | 0.767 (0.576 - 0.938) | 39.6 | 0.1449 | 0.195 | 0.56 (0.404 - 0.713) | 75.9 | 0.5786 | 0.028 | 0.667 (0.111 - 1) | 20.8 | 0.4807 | 0.035 |
| % Time ΔCPPopt-PAx > 10 mmHg | 0.719 (0.505 - 0.9) | 41.4 | 0.1603 | 0.123 | 0.598 (0.442 - 0.749) | 75 | 0.5786 | 0.05 | 0.611 (0.056 - 1) | 21.2 | 0.5250 | 0 |
| % Time ΔCPPopt-PAx < -5 mmHg | 0.633 (0.419 - 0.848) | 42.2 | 0.2457 | 0.085 | 0.579 (0.43 - 0.733) | 76.5 | 0.5786 | 0.013 | 0.519 (0.13 - 0.833) | 21.1 | 0.5614 | 0.007 |
| % Time ΔCPPopt-PAx < -10 mmHg | 0.595 (0.367 - 0.805) | 43 | 0.3234 | 0.053 | 0.514 (0.365 - 0.67) | 76.9 | 0.5951 | 0.003 | 0.407 (0.167 - 0.963) | 21.1 | 0.7000 | 0.011 |
| % Time CPP > ULR-PAx | 0.581 (0.348 - 0.814) | 39.3 | 0.3290 | 0.205 | 0.547 (0.299 - 0.612) | 74.9 | 0.5786 | 0.053 | 0.759 (0.444 - 1) | 19.1 | 0.3885 | 0.175 |
| % Time CPP < LLR-PAx | 0.586 (0.338 - 0.8) | 38.4 | 0.3290 | 0.241 | 0.624 (0.465 - 0.769) | 73.4 | 0.5786 | 0.089 | 0.611 (0.24 - 0.944) | 20.8 | 0.5250 | 0.034 |
| CPPopt-RAC | 0.576 (0.214 - 0.652) | 43.6 | 0.3290 | 0.027 | 0.494 (0.335 - 0.66) | 76.9 | 0.5951 | 0.002 | 0.519 (0.13 - 0.889) | 21.2 | 0.5614 | 0.001 |
| % Time ΔCPPopt-RAC > 5 mmHg | 0.719 (0.514 - 0.905) | 42.7 | 0.1603 | 0.066 | 0.532 (0.306 - 0.632) | 76.6 | 0.5786 | 0.01 | 0.759 (0.278 - 1) | 19.6 | 0.3885 | 0.133 |
| % Time ΔCPPopt-RAC > 10 mmHg | 0.657 (0.443 - 0.857) | 42.5 | 0.2170 | 0.076 | 0.589 (0.427 - 0.753) | 74.1 | 0.5786 | 0.072 | 0.685 (0.222 - 1) | 20.9 | 0.4678 | 0.027 |
| % Time ΔCPPopt-RAC < -5 mmHg | 0.624 (0.4 - 0.829) | 42.5 | 0.2579 | 0.073 | 0.547 (0.395 - 0.707) | 76.9 | 0.5786 | 0.004 | 0.556 (0 - 0.944) | 20.7 | 0.5614 | 0.043 |
| % Time ΔCPPopt-RAC < -10 mmHg | 0.667 (0.452 - 0.848) | 42.2 | 0.2170 | 0.087 | 0.49 (0.339 - 0.655) | 77 | 0.5951 | 0 | 0.574 (0 - 0.963) | 20.7 | 0.5614 | 0.042 |
| % Time CPP > ULR-RAC | 0.648 (0.419 - 0.852) | 38.8 | 0.2170 | 0.225 | 0.487 (0.348 - 0.67) | 76.6 | 0.5951 | 0.01 | 0.722 (0.389 - 0.963) | 19.3 | 0.3924 | 0.156 |
| % Time CPP < LLR-RAC | 0.61 (0.39 - 0.81) | 39.6 | 0.2900 | 0.195 | 0.53 (0.378 - 0.691) | 75.8 | 0.5786 | 0.029 | 0.556 (0.167 - 0.907) | 21 | 0.5614 | 0.022 |
| **3 Month 🡪 6 Month** | | | | | | | | | | | | |
|  | **Age < 30** | | | | **Age 30 – 60** | | | | **Age > 60** | | | |
| **Model** | **AUC (95% CI)** | **AIC** | **p-value** | **Nagelkerke's R^2^** | **AUC (95% CI)** | **AIC** | **p-value** | **Nagelkerke's R^2^** | **AUC (95% CI)** | **AIC** | **p-value** | **Nagelkerke's R^2^** |
| CPPopt-PRx | 0.581 (0.338 - 0.818) | 41.6 | 0.3447 | 0.04 | 0.621 (0.464 - 0.766) | 68.1 | 0.2112 | 0.063 | 0.737 (0.421 - 1) | 16.3 | 0.2769 | 0.086 |
| % Time ΔCPPopt-PRx > 5 mmHg | 0.732 (0.53 - 0.899) | 37.8 | 0.1379 | 0.204 | 0.529 (0.297 - 0.654) | 70.3 | 0.4324 | 0.006 | 0.974 (0.895 - 1) | 9.1 | 0.0997 | 0.683 |
| % Time ΔCPPopt-PRx > 10 mmHg | 0.747 (0.551 - 0.909) | 37.9 | 0.1379 | 0.2 | 0.551 (0.27 - 0.619) | 70.3 | 0.3811 | 0.006 | 0.947 (0.842 - 1) | 9.4 | 0.0997 | 0.664 |
| % Time ΔCPPopt-PRx < -5 mmHg | 0.662 (0.449 - 0.848) | 37.9 | 0.2388 | 0.198 | 0.57 (0.397 - 0.73) | 69.9 | 0.3164 | 0.017 | 0.868 (0.684 - 1) | 13.9 | 0.1998 | 0.315 |
| % Time ΔCPPopt-PRx < -10 mmHg | 0.626 (0.399 - 0.818) | 39.2 | 0.2413 | 0.145 | 0.591 (0.422 - 0.752) | 69.6 | 0.2812 | 0.023 | 0.816 (0.632 - 1) | 14.1 | 0.2221 | 0.291 |
| % Time CPP > ULR-PRx | 0.535 (0.313 - 0.743) | 41.5 | 0.3869 | 0.047 | 0.631 (0.441 - 0.794) | 65.9 | 0.2112 | 0.116 | 0.763 (0.368 - 1) | 12.7 | 0.2728 | 0.414 |
| % Time CPP < LLR-PRx | 0.601 (0.389 - 0.798) | 39.2 | 0.2902 | 0.146 | 0.641 (0.466 - 0.806) | 67.2 | 0.2112 | 0.085 | 0.553 (0.053 - 1) | 16.8 | 0.5263 | 0.043 |
| CPPopt-PAx | 0.626 (0.409 - 0.833) | 40.4 | 0.2413 | 0.096 | 0.52 (0.346 - 0.688) | 70.2 | 0.4438 | 0.007 | 0.526 (0.263 - 0.789) | 17 | 0.5263 | 0.022 |
| % Time ΔCPPopt-PAx > 5 mmHg | 0.682 (0.455 - 0.869) | 40.5 | 0.2388 | 0.091 | 0.626 (0.458 - 0.776) | 68.5 | 0.2112 | 0.052 | 0.895 (0.737 - 1) | 14.3 | 0.1802 | 0.278 |
| % Time ΔCPPopt-PAx > 10 mmHg | 0.667 (0.424 - 0.874) | 39 | 0.2388 | 0.155 | 0.578 (0.397 - 0.75) | 69.4 | 0.3164 | 0.028 | 1 (1 - 1) | 4 | **0.0021** | 1 |
| % Time ΔCPPopt-PAx < -5 mmHg | 0.611 (0.379 - 0.808) | 41.1 | 0.2743 | 0.066 | 0.675 (0.498 - 0.842) | 67 | 0.1092 | 0.09 | 0.553 (0.316 - 0.789) | 17.1 | 0.5263 | 0.013 |
| % Time ΔCPPopt-PAx < -10 mmHg | 0.551 (0.333 - 0.758) | 41.4 | 0.3712 | 0.051 | 0.616 (0.44 - 0.783) | 69.1 | 0.2112 | 0.036 | 0.632 (0.421 - 0.842) | 16.8 | 0.4572 | 0.046 |
| % Time CPP > ULR-PAx | 0.556 (0.323 - 0.788) | 42.4 | 0.3712 | 0.003 | 0.743 (0.592 - 0.877) | 63.5 | 0.0420 | 0.174 | 0.816 (0.526 - 1) | 15.8 | 0.2221 | 0.135 |
| % Time CPP < LLR-PAx | 0.677 (0.465 - 0.859) | 37.9 | 0.2388 | 0.198 | 0.57 (0.402 - 0.724) | 69.8 | 0.3164 | 0.019 | 0.605 (0.262 - 0.947) | 17.2 | 0.4801 | 0.003 |
| CPPopt-RAC | 0.571 (0.328 - 0.788) | 40.9 | 0.3609 | 0.072 | 0.515 (0.343 - 0.681) | 70.1 | 0.4438 | 0.012 | 0.474 (0.316 - 0.737) | 17 | 0.6000 | 0.017 |
| % Time ΔCPPopt-RAC > 5 mmHg | 0.636 (0.399 - 0.854) | 39.8 | 0.2413 | 0.119 | 0.596 (0.435 - 0.757) | 69.5 | 0.2812 | 0.026 | 0.842 (0.632 - 1) | 13.9 | 0.2221 | 0.316 |
| % Time ΔCPPopt-RAC > 10 mmHg | 0.631 (0.384 - 0.859) | 39.6 | 0.2413 | 0.129 | 0.546 (0.376 - 0.721) | 70.3 | 0.3811 | 0.004 | 0.947 (0.789 - 1) | 11.2 | 0.0997 | 0.53 |
| % Time ΔCPPopt-RAC < -5 mmHg | 0.631 (0.414 - 0.823) | 40.7 | 0.2413 | 0.081 | 0.544 (0.373 - 0.709) | 70 | 0.3811 | 0.014 | 0.526 (0.158 - 0.842) | 17.1 | 0.5263 | 0.009 |
| % Time ΔCPPopt-RAC < -10 mmHg | 0.545 (0.333 - 0.753) | 41.7 | 0.3712 | 0.037 | 0.513 (0.328 - 0.69) | 70.5 | 0.4438 | 0 | 0.421 (0.263 - 0.842) | 17.1 | 0.6571 | 0.006 |
| % Time CPP > ULR-RAC | 0.545 (0.323 - 0.773) | 42.4 | 0.3712 | 0.003 | 0.714 (0.56 - 0.858) | 66.5 | 0.0620 | 0.102 | 0.789 (0.474 - 1) | 15.9 | 0.2499 | 0.133 |
| % Time CPP < LLR-RAC | 0.747 (0.556 - 0.914) | 38.4 | 0.1379 | 0.181 | 0.698 (0.536 - 0.843) | 66.1 | 0.0721 | 0.111 | 0.737 (0.474 - 1) | 17.1 | 0.2769 | 0.009 |
| **1 Month 🡪 6 Month** | | | | | | | | | | | | |
|  | **Age < 30** | | | | **Age 30 – 60** | | | | **Age > 60** | | | |
| **Model** | **AUC (95% CI)** | **AIC** | **p-value** | **Nagelkerke's R^2^** | **AUC (95% CI)** | **AIC** | **p-value** | **Nagelkerke's R^2^** | **AUC (95% CI)** | **AIC** | **p-value** | **Nagelkerke's R^2^** |
| CPPopt-PRx | 0.505 (0.279 - 0.732) | 41.4 | 0.5156 | 0 | 0.578 (0.422 - 0.728) | 74.5 | 0.4284 | 0.052 | 0.824 (0.618 - 0.985) | 21 | 0.1813 | 0.244 |
| % Time ΔCPPopt-PRx > 5 mmHg | 0.826 (0.642 - 0.963) | 31.2 | **0.0089** | 0.41 | 0.562 (0.401 - 0.712) | 75.7 | 0.4284 | 0.022 | 0.574 (0.103 - 0.985) | 23.6 | 0.6831 | 0.064 |
| % Time ΔCPPopt-PRx > 10 mmHg | 0.842 (0.653 - 0.979) | 30.2 | **0.0070** | 0.441 | 0.58 (0.417 - 0.73) | 75.2 | 0.4284 | 0.033 | 0.603 (0.221 - 0.941) | 22.8 | 0.6831 | 0.12 |
| % Time ΔCPPopt-PRx < -5 mmHg | 0.674 (0.463 - 0.868) | 37.2 | 0.1113 | 0.185 | 0.513 (0.351 - 0.674) | 76.5 | 0.4862 | 0.001 | 0.956 (0.824 - 1) | 13.3 | **0.0252** | 0.661 |
| % Time ΔCPPopt-PRx < -10 mmHg | 0.558 (0.205 - 0.695) | 37.7 | 0.3693 | 0.163 | 0.508 (0.352 - 0.667) | 76.5 | 0.4875 | 0 | 0.882 (0.706 - 1) | 16.2 | 0.0935 | 0.521 |
| % Time CPP > ULR-PRx | 0.384 (0.379 - 0.837) | 41.4 | 0.8462 | 0 | 0.543 (0.377 - 0.697) | 75.8 | 0.4371 | 0.019 | 0.618 (0.294 - 0.912) | 22.3 | 0.6831 | 0.156 |
| % Time CPP < LLR-PRx | 0.658 (0.426 - 0.858) | 37.4 | 0.1342 | 0.175 | 0.529 (0.37 - 0.681) | 76.3 | 0.4750 | 0.007 | 0.574 (0.206 - 0.941) | 23.2 | 0.6831 | 0.089 |
| CPPopt-PAx | 0.589 (0.332 - 0.811) | 40.9 | 0.2815 | 0.021 | 0.555 (0.291 - 0.597) | 75.1 | 0.4353 | 0.036 | 0.559 (0.235 - 0.882) | 24.2 | 0.6831 | 0.017 |
| % Time ΔCPPopt-PAx > 5 mmHg | 0.889 (0.753 - 0.995) | 29.7 | **0.0042** | 0.457 | 0.575 (0.409 - 0.726) | 75.1 | 0.4284 | 0.036 | 0.471 (0 - 0.882) | 24.4 | 0.6831 | 0.001 |
| % Time ΔCPPopt-PAx > 10 mmHg | 0.853 (0.695 - 0.979) | 29.8 | **0.0070** | 0.454 | 0.597 (0.43 - 0.744) | 74.2 | 0.4284 | 0.059 | 0.559 (0.118 - 1) | 23.5 | 0.6831 | 0.074 |
| % Time ΔCPPopt-PAx < -5 mmHg | 0.763 (0.547 - 0.937) | 34.5 | **0.0223** | 0.292 | 0.484 (0.364 - 0.68) | 76.5 | 0.5811 | 0 | 0.485 (0.221 - 0.809) | 24.4 | 0.6831 | 0.001 |
| % Time ΔCPPopt-PAx < -10 mmHg | 0.742 (0.526 - 0.916) | 35.9 | **0.0308** | 0.238 | 0.519 (0.319 - 0.646) | 76.5 | 0.4807 | 0 | 0.412 (0.235 - 0.897) | 24.4 | 0.7128 | 0.001 |
| % Time CPP > ULR-PAx | 0.611 (0.347 - 0.853) | 38.8 | 0.2318 | 0.118 | 0.564 (0.4 - 0.72) | 76.5 | 0.4284 | 0.001 | 0.426 (0.176 - 0.971) | 24.4 | 0.7128 | 0.004 |
| % Time CPP < LLR-PAx | 0.779 (0.563 - 0.958) | 29.4 | **0.0163** | 0.466 | 0.583 (0.42 - 0.738) | 75.5 | 0.4284 | 0.026 | 0.485 (0.147 - 0.868) | 24.4 | 0.6831 | 0.002 |
| CPPopt-RAC | 0.553 (0.232 - 0.695) | 40.8 | 0.3693 | 0.025 | 0.565 (0.403 - 0.719) | 75.2 | 0.4284 | 0.034 | 0.485 (0.221 - 0.824) | 24.4 | 0.6831 | 0 |
| % Time ΔCPPopt-RAC > 5 mmHg | 0.805 (0.637 - 0.947) | 34 | **0.0099** | 0.31 | 0.568 (0.281 - 0.594) | 75.5 | 0.4284 | 0.027 | 0.588 (0 - 0.853) | 24.4 | 0.6831 | 0.002 |
| % Time ΔCPPopt-RAC > 10 mmHg | 0.758 (0.558 - 0.905) | 34.7 | **0.0231** | 0.285 | 0.601 (0.442 - 0.749) | 73.4 | 0.4284 | 0.077 | 0.5 (0 - 0.971) | 24.1 | 0.6831 | 0.029 |
| % Time ΔCPPopt-RAC < -5 mmHg | 0.805 (0.616 - 0.958) | 33.4 | **0.0099** | 0.331 | 0.525 (0.365 - 0.686) | 76.3 | 0.4750 | 0.006 | 0.471 (0.088 - 0.868) | 24.4 | 0.6831 | 0.005 |
| % Time ΔCPPopt-RAC < -10 mmHg | 0.811 (0.642 - 0.947) | 34.4 | **0.0099** | 0.294 | 0.541 (0.377 - 0.696) | 76.4 | 0.4371 | 0.004 | 0.529 (0.147 - 0.882) | 24.3 | 0.6831 | 0.01 |
| % Time CPP > ULR-RAC | 0.626 (0.389 - 0.837) | 39.2 | 0.2005 | 0.098 | 0.612 (0.455 - 0.77) | 75.8 | 0.4284 | 0.019 | 0.456 (0.191 - 0.882) | 24.4 | 0.6836 | 0.006 |
| % Time CPP < LLR-RAC | 0.784 (0.584 - 0.953) | 31.5 | 0.0160 | 0.397 | 0.551 (0.388 - 0.7) | 76.5 | 0.4353 | 0.001 | 0.662 (0.309 - 0.941) | 23.7 | 0.6831 | 0.054 |

*All p-values have been adjusted using the False Discovery Rate (FDR) method. Bolded p-values are those reaching statistical significance, p < 0.05.*

*AIC = Akaike information criterion, AMP = pulse amplitude of ICP, AUC = area under the curve, CI = confidence interval, CPP = cerebral perfusion pressure, CPPopt = cerebral perfusion pressure optimum, ΔCPPopt = CPP - CPPopt, GOSE = Glasgow Outcome Scale-Extended, ICP = intracranial pressure, LLR = lower limit of reactivity, mmHg = millimeters of mercury, PAx = pulse amplitude index, PRx = pressure reactivity index, RAC = correlation (R) between slow-waves of AMP (A) and CPP (C), ULR = upper limit of reactivity.*

Appendix N. Multivariable Models of Cerebrovascular Reactivity Measures + IMPACT Core ± CT ± ICP > 20 or 22 mmHg for Transition in Outcome from 1 to 3 Months

| **Model** | **AUC (95% CI)** | **AIC** | **p-value** | **Nagelkerke's R^2^** |
| --- | --- | --- | --- | --- |
| Core | 0.686 (0.582 - 0.783) | 136.7 | **0.0007** | 0.155 |
| Core + CPPopt-PRx | 0.686 (0.574 - 0.783) | 138.7 | **0.0007** | 0.155 |
| Core + % Time ΔCPPopt-PRx > 5 mmHg | 0.703 (0.603 - 0.797) | 137.5 | **0.0003** | 0.169 |
| Core + % Time ΔCPPopt-PRx > 10 mmHg | 0.719 (0.616 - 0.813) | 135.5 | **0.0002** | 0.191 |
| Core + % Time ΔCPPopt-PRx < -5 mmHg | 0.696 (0.594 - 0.79) | 138.5 | **0.0005** | 0.157 |
| Core + % Time ΔCPPopt-PRx < -10 mmHg | 0.692 (0.588 - 0.783) | 138.6 | **0.0006** | 0.156 |
| Core + % Time CPP > ULR-PRx | 0.689 (0.584 - 0.788) | 138.4 | **0.0006** | 0.158 |
| Core + % Time CPP < LLR-PRx | 0.701 (0.603 - 0.798) | 137.6 | **0.0004** | 0.167 |
| Core + CPPopt-PAx | 0.697 (0.592 - 0.792) | 137.9 | **0.0005** | 0.164 |
| Core + % Time ΔCPPopt-PAx > 5 mmHg | 0.69 (0.585 - 0.787) | 137.1 | **0.0006** | 0.173 |
| Core + % Time ΔCPPopt-PAx > 10 mmHg | 0.72 (0.623 - 0.811) | 135.6 | **0.0002** | 0.19 |
| Core + % Time ΔCPPopt-PAx < -5 mmHg | 0.695 (0.596 - 0.792) | 138.1 | **0.0005** | 0.162 |
| Core + % Time ΔCPPopt-PAx < -10 mmHg | 0.7 (0.598 - 0.799) | 138.0 | **0.0004** | 0.163 |
| Core + % Time CPP > ULR-PAx | 0.731 (0.634 - 0.819) | 132.0 | **<0.0001** | 0.23 |
| Core + % Time CPP < LLR-PAx | 0.738 (0.642 - 0.834) | 130.9 | **<0.0001** | 0.242 |
| Core + CPPopt-RAC | 0.707 (0.605 - 0.8) | 137.4 | **0.0003** | 0.17 |
| Core + % Time ΔCPPopt-RAC > 5 mmHg | 0.69 (0.585 - 0.786) | 138.4 | **0.0005** | 0.158 |
| Core + % Time ΔCPPopt-RAC > 10 mmHg | 0.715 (0.61 - 0.811) | 135.9 | **0.0002** | 0.187 |
| Core + % Time ΔCPPopt-RAC < -5 mmHg | 0.693 (0.592 - 0.786) | 138.3 | **0.0005** | 0.16 |
| Core + % Time ΔCPPopt-RAC < -10 mmHg | 0.689 (0.583 - 0.787) | 138.5 | **0.0006** | 0.157 |
| Core + % Time CPP > ULR-RAC | 0.706 (0.601 - 0.802) | 135.5 | **0.0003** | 0.191 |
| Core + % Time CPP < LLR-RAC | 0.718 (0.616 - 0.812) | 135.3 | **0.0002** | 0.194 |
| Core + CT | 0.697 (0.595 - 0.797) | 141.7 | **0.0005** | 0.166 |
| Core + CT + CPPopt-PRx | 0.701 (0.59 - 0.795) | 143.7 | **0.0004** | 0.166 |
| Core + CT + % Time ΔCPPopt-PRx > 5 mmHg | 0.714 (0.613 - 0.811) | 142.5 | **0.0002** | 0.18 |
| Core + CT + % Time ΔCPPopt-PRx > 10 mmHg | 0.733 (0.632 - 0.827) | 140.2 | **<0.0001** | 0.206 |
| Core + CT + % Time ΔCPPopt-PRx < -5 mmHg | 0.7 (0.594 - 0.794) | 143.6 | **0.0004** | 0.168 |
| Core + CT + % Time ΔCPPopt-PRx < -10 mmHg | 0.698 (0.592 - 0.803) | 143.6 | **0.0004** | 0.167 |
| Core + CT + % Time CPP > ULR-PRx | 0.7 (0.596 - 0.797) | 143.5 | **0.0004** | 0.169 |
| Core + CT + % Time CPP < LLR-PRx | 0.712 (0.606 - 0.806) | 142.6 | **0.0002** | 0.18 |
| Core + CT + CPPopt-PAx | 0.709 (0.605 - 0.804) | 143.1 | **0.0003** | 0.174 |
| Core + CT + % Time ΔCPPopt-PAx > 5 mmHg | 0.708 (0.602 - 0.803) | 141.7 | **0.0003** | 0.189 |
| Core + CT + % Time ΔCPPopt-PAx > 10 mmHg | 0.734 (0.63 - 0.825) | 139.7 | **<0.0001** | 0.212 |
| Core + CT + % Time ΔCPPopt-PAx < -5 mmHg | 0.711 (0.605 - 0.807) | 143.3 | **0.0002** | 0.172 |
| Core + CT + % Time ΔCPPopt-PAx < -10 mmHg | 0.712 (0.606 - 0.809) | 143.1 | **0.0002** | 0.173 |
| Core + CT + % Time CPP > ULR-PAx | 0.74 (0.642 - 0.831) | 136.8 | **<0.0001** | 0.243 |
| Core + CT + % Time CPP < LLR-PAx | 0.756 (0.664 - 0.844) | 134.8 | **<0.0001** | 0.264 |
| Core + CT + CPPopt-RAC | 0.719 (0.618 - 0.819) | 142.6 | **0.0002** | 0.179 |
| Core + CT + % Time ΔCPPopt-RAC > 5 mmHg | 0.706 (0.602 - 0.802) | 143.2 | **0.0003** | 0.172 |
| Core + CT + % Time ΔCPPopt-RAC > 10 mmHg | 0.73 (0.626 - 0.823) | 140.0 | **<0.0001** | 0.208 |
| Core + CT + % Time ΔCPPopt-RAC < -5 mmHg | 0.707 (0.603 - 0.804) | 143.5 | **0.0003** | 0.169 |
| Core + CT + % Time ΔCPPopt-RAC < -10 mmHg | 0.703 (0.598 - 0.799) | 143.6 | **0.0004** | 0.168 |
| Core + CT + % Time CPP > ULR-RAC | 0.716 (0.607 - 0.811) | 140.6 | **0.0002** | 0.202 |
| Core + CT + % Time CPP < LLR-RAC | 0.736 (0.635 - 0.827) | 140.1 | **<0.0001** | 0.207 |
| Core + CT + ICP > 20 mmHg | 0.743 (0.653 - 0.835) | 136.0 | **<0.0001** | 0.252 |
| Core + CT + ICP > 20 mmHg + CPPopt-PRx | 0.746 (0.649 - 0.84) | 137.8 | **<0.0001** | 0.254 |
| Core + CT + ICP > 20 mmHg + % Time ΔCPPopt-PRx > 5 mmHg | 0.742 (0.639 - 0.833) | 137.8 | **<0.0001** | 0.253 |
| Core + CT + ICP > 20 mmHg + % Time ΔCPPopt-PRx > 10 mmHg | 0.754 (0.66 - 0.841) | 136.1 | **<0.0001** | 0.271 |
| Core + CT + ICP > 20 mmHg + % Time ΔCPPopt-PRx < -5 mmHg | 0.743 (0.646 - 0.831) | 137.1 | **<0.0001** | 0.261 |
| Core + CT + ICP > 20 mmHg + % Time ΔCPPopt-PRx < -10 mmHg | 0.753 (0.656 - 0.837) | 135.9 | **<0.0001** | 0.273 |
| Core + CT + ICP > 20 mmHg + % Time CPP > ULR-PRx | 0.747 (0.651 - 0.835) | 137.8 | **<0.0001** | 0.254 |
| Core + CT + ICP > 20 mmHg + % Time CPP < LLR-PRx | 0.742 (0.647 - 0.832) | 138.0 | **<0.0001** | 0.252 |
| Core + CT + ICP > 20 mmHg + CPPopt-PAx | 0.749 (0.652 - 0.835) | 137.6 | **<0.0001** | 0.255 |
| Core + CT + ICP > 20 mmHg + % Time ΔCPPopt-PAx > 5 mmHg | 0.744 (0.64 - 0.833) | 136.7 | **<0.0001** | 0.265 |
| Core + CT + ICP > 20 mmHg + % Time ΔCPPopt-PAx > 10 mmHg | 0.756 (0.663 - 0.845) | 134.7 | **<0.0001** | 0.286 |
| Core + CT + ICP > 20 mmHg + % Time ΔCPPopt-PAx < -5 mmHg | 0.756 (0.659 - 0.843) | 136.1 | **<0.0001** | 0.272 |
| Core + CT + ICP > 20 mmHg + % Time ΔCPPopt-PAx < -10 mmHg | 0.76 (0.661 - 0.845) | 135.8 | **<0.0001** | 0.275 |
| Core + CT + ICP > 20 mmHg + % Time CPP > ULR-PAx | 0.757 (0.663 - 0.84) | 135.0 | **<0.0001** | 0.283 |
| Core + CT + ICP > 20 mmHg + % Time CPP < LLR-PAx | 0.763 (0.671 - 0.853) | 135.4 | **<0.0001** | 0.279 |
| Core + CT + ICP > 20 mmHg + CPPopt-RAC | 0.746 (0.651 - 0.836) | 137.4 | **<0.0001** | 0.258 |
| Core + CT + ICP > 20 mmHg + % Time ΔCPPopt-RAC > 5 mmHg | 0.742 (0.646 - 0.833) | 137.8 | **<0.0001** | 0.253 |
| Core + CT + ICP > 20 mmHg + % Time ΔCPPopt-RAC > 10 mmHg | 0.764 (0.669 - 0.85) | 134.6 | **<0.0001** | 0.287 |
| Core + CT + ICP > 20 mmHg + % Time ΔCPPopt-RAC < -5 mmHg | 0.756 (0.66 - 0.847) | 136.3 | **<0.0001** | 0.269 |
| Core + CT + ICP > 20 mmHg + % Time ΔCPPopt-RAC < -10 mmHg | 0.751 (0.658 - 0.836) | 136.5 | **<0.0001** | 0.267 |
| Core + CT + ICP > 20 mmHg + % Time CPP > ULR-RAC | 0.744 (0.646 - 0.827) | 137.4 | **<0.0001** | 0.258 |
| Core + CT + ICP > 20 mmHg + % Time CPP < LLR-RAC | 0.743 (0.643 - 0.83) | 138.0 | **<0.0001** | 0.252 |
| Core + CT + ICP > 22 mmHg | 0.752 (0.651 – 0.84) | 134.5 | **<0.0001** | 0.268 |
| Core + CT + ICP > 22 mmHg + CPPopt-PRx | 0.754 (0.658 – 0.842) | 136.2 | **<0.0001** | 0.27 |
| Core + CT + ICP > 22 mmHg + % Time ΔCPPopt-PRx > 5 mmHg | 0.754 (0.657 – 0.844) | 136.3 | **<0.0001** | 0.269 |
| Core + CT + ICP > 22 mmHg + % Time ΔCPPopt-PRx > 10 mmHg | 0.765 (0.669 – 0.851) | 134.4 | **<0.0001** | 0.289 |
| Core + CT + ICP > 22 mmHg + % Time ΔCPPopt-PRx < -5 mmHg | 0.757 (0.663 – 0.845) | 135.4 | **<0.0001** | 0.279 |
| Core + CT + ICP > 22 mmHg + % Time ΔCPPopt-PRx < -10 mmHg | 0.769 (0.676 – 0.855) | 134.1 | **<0.0001** | 0.292 |
| Core + CT + ICP > 22 mmHg + % Time CPP > ULR-PRx | 0.753 (0.653 – 0.841) | 136.4 | **<0.0001** | 0.269 |
| Core + CT + ICP > 22 mmHg + % Time CPP < LLR-PRx | 0.752 (0.652 – 0.842) | 136.4 | **<0.0001** | 0.268 |
| Core + CT + ICP > 22 mmHg + CPPopt-PAx | 0.76 (0.665 – 0.845) | 136.0 | **<0.0001** | 0.272 |
| Core + CT + ICP > 22 mmHg + % Time ΔCPPopt-PAx > 5 mmHg | 0.749 (0.651 - 0.831) | 135.4 | **<0.0001** | 0.279 |
| Core + CT + ICP > 22 mmHg + % Time ΔCPPopt-PAx > 10 mmHg | 0.766 (0.673 - 0.852) | 132.9 | **<0.0001** | 0.305 |
| Core + CT + ICP > 22 mmHg + % Time ΔCPPopt-PAx < -5 mmHg | 0.77 (0.678 - 0.858) | 134.4 | **<0.0001** | 0.29 |
| Core + CT + ICP > 22 mmHg + % Time ΔCPPopt-PAx < -10 mmHg | 0.771 (0.675 - 0.856) | 134.2 | **<0.0001** | 0.292 |
| Core + CT + ICP > 22 mmHg + % Time CPP > ULR-PAx | 0.765 (0.662 - 0.849) | 133.7 | **<0.0001** | 0.296 |
| Core + CT + ICP > 22 mmHg + % Time CPP < LLR-PAx | 0.77 (0.674 - 0.851) | 134.2 | **<0.0001** | 0.292 |
| Core + CT + ICP > 22 mmHg + CPPopt-RAC | 0.756 (0.663 - 0.843) | 135.7 | **<0.0001** | 0.276 |
| Core + CT + ICP > 22 mmHg + % Time ΔCPPopt-RAC > 5 mmHg | 0.753 (0.653 - 0.84) | 136.4 | **<0.0001** | 0.269 |
| Core + CT + ICP > 22 mmHg + % Time ΔCPPopt-RAC > 10 mmHg | 0.77 (0.671 - 0.854) | 133.1 | **<0.0001** | 0.303 |
| Core + CT + ICP > 22 mmHg + % Time ΔCPPopt-RAC < -5 mmHg | 0.768 (0.668 - 0.852) | 134.6 | **<0.0001** | 0.287 |
| Core + CT + ICP > 22 mmHg + % Time ΔCPPopt-RAC < -10 mmHg | 0.764 (0.667 - 0.846) | 134.9 | **<0.0001** | 0.284 |
| Core + CT + ICP > 22 mmHg + % Time CPP > ULR-RAC | 0.749 (0.65 - 0.838) | 136.0 | **<0.0001** | 0.272 |
| Core + CT + ICP > 22 mmHg + % Time CPP < LLR-RAC | 0.751 (0.659 - 0.837) | 136.5 | **<0.0001** | 0.268 |

*Core model consisted of age, admission Glasgow Coma Scale - motor score, and admission pupillary response. CT variables consisted of admission Marshall CT grade, presence of traumatic subarachnoid hemorrhage, and presence of epidural hematoma. All p-values have been adjusted using the False Discovery Rate (FDR) method. Bolded p-values are those reaching statistical significance, p < 0.05.*

*AIC = Akaike information criterion, AMP = pulse amplitude of ICP, AUC = area under the curve, CI = confidence interval, CPP = cerebral perfusion pressure, CPPopt = cerebral perfusion pressure optimum, ΔCPPopt = CPP - CPPopt, CT = computed tomography, ICP = intracranial pressure, IMPACT = International Mission for Prognosis and Analysis of Clinical Trials, mmHg = millimeters of mercury, LLR = lower limit of reactivity, PAx = pulse amplitude index, PRx = pressure reactivity index, RAC = correlation (R) between slow-waves of AMP (A) and CPP (C), ULR = upper limit of reactivity.*

Appendix O. Multivariable Models of Cerebrovascular Reactivity Measures + IMPACT Core ± CT ± ICP > 20 or 22 mmHg for Transition in Outcome from 3 to 6 Months

| **Model** | **AUC (95% CI)** | **AIC** | **p-value** | **Nagelkerke's R^2^** |
| --- | --- | --- | --- | --- |
| Core | 0.629 (0.512 - 0.743) | 129.4 | **0.0206** | 0.067 |
| Core + CPPopt-PRx | 0.663 (0.552 - 0.771) | 128.7 | **0.0057** | 0.101 |
| Core + % Time ΔCPPopt-PRx > 5 mmHg | 0.66 (0.552 - 0.763) | 129.3 | **0.0067** | 0.093 |
| Core + % Time ΔCPPopt-PRx > 10 mmHg | 0.667 (0.55 - 0.776) | 128.7 | **0.0050** | 0.101 |
| Core + % Time ΔCPPopt-PRx < -5 mmHg | 0.638 (0.514 - 0.746) | 130.6 | **0.0155** | 0.077 |
| Core + % Time ΔCPPopt-PRx < -10 mmHg | 0.63 (0.51 - 0.741) | 131.1 | **0.0201** | 0.069 |
| Core + % Time CPP > ULR-PRx | 0.73 (0.621 - 0.828) | 120 | **0.0006** | 0.209 |
| Core + % Time CPP < LLR-PRx | 0.639 (0.517 - 0.746) | 131.2 | **0.0152** | 0.069 |
| Core + CPPopt-PAx | 0.65 (0.526 - 0.764) | 129.5 | **0.0100** | 0.091 |
| Core + % Time ΔCPPopt-PAx > 5 mmHg | 0.689 (0.58 - 0.792) | 126.3 | **0.0019** | 0.132 |
| Core + % Time ΔCPPopt-PAx > 10 mmHg | 0.693 (0.583 - 0.8) | 125 | **0.0015** | 0.148 |
| Core + % Time ΔCPPopt-PAx < -5 mmHg | 0.67 (0.551 - 0.782) | 128 | **0.0044** | 0.11 |
| Core + % Time ΔCPPopt-PAx < -10 mmHg | 0.652 (0.534 - 0.763) | 129.8 | **0.0095** | 0.087 |
| Core + % Time CPP > ULR-PAx | 0.705 (0.598 - 0.8) | 126 | **0.0009** | 0.136 |
| Core + % Time CPP < LLR-PAx | 0.633 (0.52 - 0.745) | 131 | **0.0183** | 0.071 |
| Core + CPPopt-RAC | 0.647 (0.531 - 0.764) | 129.6 | **0.0114** | 0.09 |
| Core + % Time ΔCPPopt-RAC > 5 mmHg | 0.677 (0.564 - 0.783) | 126.4 | **0.0033** | 0.131 |
| Core + % Time ΔCPPopt-RAC > 10 mmHg | 0.67 (0.559 - 0.773) | 127.9 | **0.0044** | 0.112 |
| Core + % Time ΔCPPopt-RAC < -5 mmHg | 0.641 (0.526 - 0.749) | 130.2 | **0.0141** | 0.082 |
| Core + % Time ΔCPPopt-RAC < -10 mmHg | 0.626 (0.504 - 0.735) | 131.3 | **0.0224** | 0.067 |
| Core + % Time CPP > ULR-RAC | 0.696 (0.595 - 0.795) | 127.4 | **0.0013** | 0.118 |
| Core + % Time CPP < LLR-RAC | 0.632 (0.513 - 0.737) | 131.3 | **0.0186** | 0.067 |
| Core + CT | 0.7 (0.586 - 0.805) | 130.1 | **0.0012** | 0.135 |
| Core + CT + CPPopt-PRx | 0.718 (0.614 - 0.814) | 130.1 | **0.0008** | 0.159 |
| Core + CT + % Time ΔCPPopt-PRx > 5 mmHg | 0.731 (0.629 - 0.825) | 130.4 | **0.0006** | 0.155 |
| Core + CT + % Time ΔCPPopt-PRx > 10 mmHg | 0.733 (0.632 - 0.825) | 130.2 | **0.0005** | 0.157 |
| Core + CT + % Time ΔCPPopt-PRx < -5 mmHg | 0.712 (0.606 - 0.804) | 130.9 | **0.0008** | 0.149 |
| Core + CT + % Time ΔCPPopt-PRx < -10 mmHg | 0.708 (0.6 - 0.809) | 131.5 | **0.0008** | 0.142 |
| Core + CT + % Time CPP > ULR-PRx | 0.787 (0.693 - 0.869) | 121.1 | **<0.0001** | 0.265 |
| Core + CT + % Time CPP < LLR-PRx | 0.702 (0.596 - 0.803) | 132 | **0.0011** | 0.135 |
| Core + CT + CPPopt-PAx | 0.708 (0.598 - 0.81) | 130.6 | **0.0008** | 0.153 |
| Core + CT + % Time ΔCPPopt-PAx > 5 mmHg | 0.745 (0.641 - 0.839) | 126.9 | **<0.0001** | 0.198 |
| Core + CT + % Time ΔCPPopt-PAx > 10 mmHg | 0.756 (0.652 - 0.851) | 126.1 | **<0.0001** | 0.208 |
| Core + CT + % Time ΔCPPopt-PAx < -5 mmHg | 0.726 (0.622 - 0.825) | 128.5 | **0.0007** | 0.178 |
| Core + CT + % Time ΔCPPopt-PAx < -10 mmHg | 0.712 (0.61 - 0.811) | 130 | **0.0008** | 0.16 |
| Core + CT + % Time CPP > ULR-PAx | 0.727 (0.625 - 0.829) | 126.7 | **0.0007** | 0.2 |
| Core + CT + % Time CPP < LLR-PAx | 0.712 (0.595 - 0.811) | 131.4 | **0.0008** | 0.143 |
| Core + CT + CPPopt-RAC | 0.71 (0.608 - 0.81) | 130.6 | **0.0008** | 0.153 |
| Core + CT + % Time ΔCPPopt-RAC > 5 mmHg | 0.748 (0.645 - 0.839) | 127 | **<0.0001** | 0.196 |
| Core + CT + % Time ΔCPPopt-RAC > 10 mmHg | 0.732 (0.625 - 0.826) | 129 | **0.0005** | 0.172 |
| Core + CT + % Time ΔCPPopt-RAC < -5 mmHg | 0.711 (0.6 - 0.81) | 130.7 | **0.0007** | 0.152 |
| Core + CT + % Time ΔCPPopt-RAC < -10 mmHg | 0.701 (0.595 - 0.803) | 131.9 | **0.0011** | 0.137 |
| Core + CT + % Time CPP > ULR-RAC | 0.724 (0.618 - 0.818) | 127.8 | **0.0007** | 0.188 |
| Core + CT + % Time CPP < LLR-RAC | 0.699 (0.585 - 0.799) | 131.9 | **0.0011** | 0.136 |
| Core + CT + ICP > 20 mmHg | 0.71 (0.609 - 0.808) | 131.3 | **0.0007** | 0.144 |
| Core + CT + ICP > 20 mmHg + CPPopt-PRx | 0.727 (0.619 - 0.825) | 131.1 | **0.0006** | 0.171 |
| Core + CT + ICP > 20 mmHg + % Time ΔCPPopt-PRx > 5 mmHg | 0.732 (0.632 - 0.828) | 132.1 | **0.0004** | 0.159 |
| Core + CT + ICP > 20 mmHg + % Time ΔCPPopt-PRx > 10 mmHg | 0.737 (0.641 - 0.829) | 131.8 | **0.0004** | 0.162 |
| Core + CT + ICP > 20 mmHg + % Time ΔCPPopt-PRx < -5 mmHg | 0.714 (0.613 - 0.81) | 132.7 | **0.0008** | 0.152 |
| Core + CT + ICP > 20 mmHg + % Time ΔCPPopt-PRx < -10 mmHg | 0.718 (0.613 - 0.817) | 133.2 | **0.0008** | 0.146 |
| Core + CT + ICP > 20 mmHg + % Time CPP > ULR-PRx | 0.792 (0.705 - 0.87) | 122.3 | **<0.0001** | 0.274 |
| Core + CT + ICP > 20 mmHg + % Time CPP < LLR-PRx | 0.716 (0.616 - 0.811) | 132.8 | **0.0007** | 0.15 |
| Core + CT + ICP > 20 mmHg + CPPopt-PAx | 0.719 (0.611 - 0.814) | 132 | **0.0007** | 0.16 |
| Core + CT + ICP > 20 mmHg + % Time ΔCPPopt-PAx > 5 mmHg | 0.751 (0.643 - 0.842) | 128.6 | **<0.0001** | 0.202 |
| Core + CT + ICP > 20 mmHg + % Time ΔCPPopt-PAx > 10 mmHg | 0.765 (0.662 - 0.855) | 127.7 | **<0.0001** | 0.212 |
| Core + CT + ICP > 20 mmHg + % Time ΔCPPopt-PAx < -5 mmHg | 0.725 (0.628 - 0.818) | 130.2 | **0.0006** | 0.182 |
| Core + CT + ICP > 20 mmHg + % Time ΔCPPopt-PAx < -10 mmHg | 0.716 (0.609 - 0.811) | 131.7 | **0.0007** | 0.164 |
| Core + CT + ICP > 20 mmHg + % Time CPP > ULR-PAx | 0.761 (0.662 - 0.849) | 126.2 | **<0.0001** | 0.23 |
| Core + CT + ICP > 20 mmHg + % Time CPP < LLR-PAx | 0.713 (0.605 - 0.81) | 133.2 | **0.0007** | 0.146 |
| Core + CT + ICP > 20 mmHg + CPPopt-RAC | 0.721 (0.618 - 0.82) | 132.1 | **0.0006** | 0.159 |
| Core + CT + ICP > 20 mmHg + % Time ΔCPPopt-RAC > 5 mmHg | 0.752 (0.653 - 0.843) | 128.7 | **<0.0001** | 0.201 |
| Core + CT + ICP > 20 mmHg + % Time ΔCPPopt-RAC > 10 mmHg | 0.74 (0.635 - 0.83) | 130.4 | **0.0004** | 0.18 |
| Core + CT + ICP > 20 mmHg + % Time ΔCPPopt-RAC < -5 mmHg | 0.716 (0.611 - 0.814) | 132.2 | **0.0007** | 0.158 |
| Core + CT + ICP > 20 mmHg + % Time ΔCPPopt-RAC < -10 mmHg | 0.711 (0.604 - 0.808) | 133.3 | **0.0007** | 0.145 |
| Core + CT + ICP > 20 mmHg + % Time CPP > ULR-RAC | 0.763 (0.667 - 0.856) | 126.9 | **<0.0001** | 0.221 |
| Core + CT + ICP > 20 mmHg + % Time CPP < LLR-RAC | 0.718 (0.606 - 0.816) | 131.8 | **0.0006** | 0.163 |
| Core + CT + ICP > 22 mmHg | 0.71 (0.607 - 0.806) | 131.2 | **0.0006** | 0.146 |
| Core + CT + ICP > 22 mmHg + CPPopt-PRx | 0.729 (0.623 - 0.824) | 131.1 | **0.0003** | 0.172 |
| Core + CT + ICP > 22 mmHg + % Time ΔCPPopt-PRx > 5 mmHg | 0.73 (0.63 - 0.821) | 132 | **0.0003** | 0.16 |
| Core + CT + ICP > 22 mmHg + % Time ΔCPPopt-PRx > 10 mmHg | 0.737 (0.63 - 0.832) | 131.8 | **0.0003** | 0.163 |
| Core + CT + ICP > 22 mmHg + % Time ΔCPPopt-PRx < -5 mmHg | 0.715 (0.615 - 0.811) | 132.6 | **0.0005** | 0.153 |
| Core + CT + ICP > 22 mmHg + % Time ΔCPPopt-PRx < -10 mmHg | 0.716 (0.611 - 0.812) | 133.1 | **0.0005** | 0.147 |
| Core + CT + ICP > 22 mmHg + % Time CPP > ULR-PRx | 0.79 (0.701 - 0.873) | 122.1 | **<0.0001** | 0.277 |
| Core + CT + ICP > 22 mmHg + % Time CPP < LLR-PRx | 0.72 (0.609 - 0.813) | 132.7 | **0.0005** | 0.152 |
| Core + CT + ICP > 22 mmHg + CPPopt-PAx | 0.718 (0.61 - 0.815) | 131.9 | **0.0005** | 0.161 |
| Core + CT + ICP > 22 mmHg + % Time ΔCPPopt-PAx > 5 mmHg | 0.75 (0.647 - 0.84) | 128.6 | **<0.0001** | 0.202 |
| Core + CT + ICP > 22 mmHg + % Time ΔCPPopt-PAx > 10 mmHg | 0.765 (0.663 - 0.854) | 127.6 | **<0.0001** | 0.213 |
| Core + CT + ICP > 22 mmHg + % Time ΔCPPopt-PAx < -5 mmHg | 0.726 (0.628 - 0.816) | 130.2 | **0.0005** | 0.182 |
| Core + CT + ICP > 22 mmHg + % Time ΔCPPopt-PAx < -10 mmHg | 0.718 (0.619 - 0.813) | 131.6 | **0.0005** | 0.165 |
| Core + CT + ICP > 22 mmHg + % Time CPP > ULR-PAx | 0.762 (0.664 - 0.853) | 126 | **<0.0001** | 0.232 |
| Core + CT + ICP > 22 mmHg + % Time CPP < LLR-PAx | 0.711 (0.602 - 0.807) | 133.1 | **0.0006** | 0.147 |
| Core + CT + ICP > 22 mmHg + CPPopt-RAC | 0.719 (0.611 - 0.816) | 132 | **0.0005** | 0.16 |
| Core + CT + ICP > 22 mmHg + % Time ΔCPPopt-RAC > 5 mmHg | 0.75 (0.652 - 0.836) | 128.7 | **<0.0001** | 0.201 |
| Core + CT + ICP > 22 mmHg + % Time ΔCPPopt-RAC > 10 mmHg | 0.737 (0.634 - 0.83) | 130.4 | **0.0003** | 0.18 |
| Core + CT + ICP > 22 mmHg + % Time ΔCPPopt-RAC < -5 mmHg | 0.716 (0.611 - 0.816) | 132.2 | **0.0005** | 0.158 |
| Core + CT + ICP > 22 mmHg + % Time ΔCPPopt-RAC < -10 mmHg | 0.708 (0.604 - 0.805) | 133.2 | **0.0007** | 0.146 |
| Core + CT + ICP > 22 mmHg + % Time CPP > ULR-RAC | 0.759 (0.654 - 0.849) | 126.7 | **<0.0001** | 0.224 |
| Core + CT + ICP > 22 mmHg + % Time CPP < LLR-RAC | 0.719 (0.612 - 0.813) | 131.6 | **0.0005** | 0.165 |

*Core model consisted of age, admission Glasgow Coma Scale - motor score, and admission pupillary response. CT variables consisted of admission Marshall CT grade, presence of traumatic subarachnoid hemorrhage, and presence of epidural hematoma. All p-values have been adjusted using the False Discovery Rate (FDR) method. Bolded p-values are those reaching statistical significance, p < 0.05.*

*AIC = Akaike information criterion, AMP = pulse amplitude of ICP, AUC = area under the curve, CI = confidence interval, CPP = cerebral perfusion pressure, CPPopt = cerebral perfusion pressure optimum, ΔCPPopt = CPP - CPPopt, CT = computed tomography, ICP = intracranial pressure, IMPACT = International Mission for Prognosis and Analysis of Clinical Trials, mmHg = millimeters of mercury, LLR = lower limit of reactivity, PAx = pulse amplitude index, PRx = pressure reactivity index, RAC = correlation (R) between slow-waves of AMP (A) and CPP (C), ULR = upper limit of reactivity.*

Appendix P. Multivariable Models of Cerebrovascular Reactivity Measures + IMPACT Core ± CT ± ICP > 20 or 22 mmHg for Transition in Outcome from 1 to 6 Months

| **Model** | **AUC (95% CI)** | **AIC** | **p-value** | **Nagelkerke's R^2^** |
| --- | --- | --- | --- | --- |
| Core | 0.727 (0.625 - 0.825) | 134.4 | **<0.0001** | 0.217 |
| Core + CPPopt-PRx | 0.734 (0.636 - 0.822) | 135.6 | **<0.0001** | 0.226 |
| Core + % Time ΔCPPopt-PRx > 5 mmHg | 0.75 (0.655 - 0.84) | 130.4 | **<0.0001** | 0.28 |
| Core + % Time ΔCPPopt-PRx > 10 mmHg | 0.771 (0.67 - 0.852) | 127.9 | **<0.0001** | 0.306 |
| Core + % Time ΔCPPopt-PRx < -5 mmHg | 0.743 (0.64 - 0.835) | 133.7 | **<0.0001** | 0.246 |
| Core + % Time ΔCPPopt-PRx < -10 mmHg | 0.739 (0.641 - 0.831) | 134.7 | **<0.0001** | 0.236 |
| Core + % Time CPP > ULR-PRx | 0.748 (0.653 - 0.839) | 132 | **<0.0001** | 0.264 |
| Core + % Time CPP < LLR-PRx | 0.741 (0.641 - 0.831) | 134.6 | **<0.0001** | 0.236 |
| Core + CPPopt-PAx | 0.748 (0.652 - 0.84) | 133.4 | **<0.0001** | 0.249 |
| Core + % Time ΔCPPopt-PAx > 5 mmHg | 0.74 (0.643 - 0.829) | 132 | **<0.0001** | 0.264 |
| Core + % Time ΔCPPopt-PAx > 10 mmHg | 0.77 (0.68 - 0.856) | 127.7 | **<0.0001** | 0.308 |
| Core + % Time ΔCPPopt-PAx < -5 mmHg | 0.73 (0.624 - 0.823) | 136.3 | **<0.0001** | 0.218 |
| Core + % Time ΔCPPopt-PAx < -10 mmHg | 0.726 (0.622 - 0.82) | 136.4 | **<0.0001** | 0.217 |
| Core + % Time CPP > ULR-PAx | 0.727 (0.625 - 0.814) | 136.4 | **<0.0001** | 0.217 |
| Core + % Time CPP < LLR-PAx | 0.771 (0.681 - 0.854) | 130.3 | **<0.0001** | 0.281 |
| Core + CPPopt-RAC | 0.751 (0.662 - 0.842) | 133.2 | **<0.0001** | 0.251 |
| Core + % Time ΔCPPopt-RAC > 5 mmHg | 0.74 (0.639 - 0.832) | 132.5 | **<0.0001** | 0.258 |
| Core + % Time ΔCPPopt-RAC > 10 mmHg | 0.762 (0.668 - 0.849) | 128.9 | **<0.0001** | 0.295 |
| Core + % Time ΔCPPopt-RAC < -5 mmHg | 0.731 (0.626 - 0.822) | 135.6 | **<0.0001** | 0.225 |
| Core + % Time ΔCPPopt-RAC < -10 mmHg | 0.726 (0.627 - 0.821) | 136.3 | **<0.0001** | 0.218 |
| Core + % Time CPP > ULR-RAC | 0.729 (0.633 - 0.822) | 136 | **<0.0001** | 0.221 |
| Core + % Time CPP < LLR-RAC | 0.746 (0.645 - 0.838) | 134.6 | **<0.0001** | 0.236 |
| Core + CT | 0.745 (0.643 - 0.837) | 137.9 | **<0.0001** | 0.244 |
| Core + CT + CPPopt-PRx | 0.758 (0.659 - 0.844) | 139.1 | **<0.0001** | 0.252 |
| Core + CT + % Time ΔCPPopt-PRx > 5 mmHg | 0.78 (0.69 - 0.866) | 133.7 | **<0.0001** | 0.308 |
| Core + CT + % Time ΔCPPopt-PRx > 10 mmHg | 0.796 (0.705 - 0.875) | 130.8 | **<0.0001** | 0.336 |
| Core + CT + % Time ΔCPPopt-PRx < -5 mmHg | 0.766 (0.677 - 0.851) | 137.3 | **<0.0001** | 0.271 |
| Core + CT + % Time ΔCPPopt-PRx < -10 mmHg | 0.762 (0.666 - 0.847) | 138 | **<0.0001** | 0.264 |
| Core + CT + % Time CPP > ULR-PRx | 0.768 (0.673 - 0.855) | 135.2 | **<0.0001** | 0.293 |
| Core + CT + % Time CPP < LLR-PRx | 0.762 (0.666 - 0.852) | 137.7 | **<0.0001** | 0.267 |
| Core + CT + CPPopt-PAx | 0.778 (0.681 - 0.867) | 137.4 | **<0.0001** | 0.27 |
| Core + CT + % Time ΔCPPopt-PAx > 5 mmHg | 0.77 (0.674 - 0.853) | 134.4 | **<0.0001** | 0.3 |
| Core + CT + % Time ΔCPPopt-PAx > 10 mmHg | 0.795 (0.707 - 0.876) | 128.7 | **<0.0001** | 0.356 |
| Core + CT + % Time ΔCPPopt-PAx < -5 mmHg | 0.751 (0.657 - 0.839) | 139.7 | **<0.0001** | 0.246 |
| Core + CT + % Time ΔCPPopt-PAx < -10 mmHg | 0.745 (0.647 - 0.83) | 139.9 | **<0.0001** | 0.244 |
| Core + CT + % Time CPP > ULR-PAx | 0.745 (0.644 - 0.837) | 139.9 | **<0.0001** | 0.244 |
| Core + CT + % Time CPP < LLR-PAx | 0.8 (0.708 - 0.882) | 132.2 | **<0.0001** | 0.323 |
| Core + CT + CPPopt-RAC | 0.778 (0.688 - 0.863) | 137.2 | **<0.0001** | 0.272 |
| Core + CT + % Time ΔCPPopt-RAC > 5 mmHg | 0.763 (0.667 - 0.846) | 135.1 | **<0.0001** | 0.293 |
| Core + CT + % Time ΔCPPopt-RAC > 10 mmHg | 0.791 (0.697 - 0.865) | 130.4 | **<0.0001** | 0.34 |
| Core + CT + % Time ΔCPPopt-RAC < -5 mmHg | 0.754 (0.657 - 0.838) | 138.7 | **<0.0001** | 0.256 |
| Core + CT + % Time ΔCPPopt-RAC < -10 mmHg | 0.749 (0.651 - 0.837) | 139.7 | **<0.0001** | 0.247 |
| Core + CT + % Time CPP > ULR-RAC | 0.755 (0.657 - 0.842) | 139.4 | **<0.0001** | 0.249 |
| Core + CT + % Time CPP < LLR-RAC | 0.772 (0.681 - 0.854) | 138 | **<0.0001** | 0.264 |
| Core + CT + ICP > 20 mmHg | 0.778 (0.685 - 0.86) | 131.8 | **<0.0001** | 0.326 |
| Core + CT + ICP > 20 mmHg + CPPopt-PRx | 0.792 (0.698 - 0.874) | 132.8 | **<0.0001** | 0.336 |
| Core + CT + ICP > 20 mmHg + % Time ΔCPPopt-PRx > 5 mmHg | 0.797 (0.706 - 0.877) | 130.7 | **<0.0001** | 0.356 |
| Core + CT + ICP > 20 mmHg + % Time ΔCPPopt-PRx > 10 mmHg | 0.811 (0.725 - 0.89) | 127.9 | **<0.0001** | 0.382 |
| Core + CT + ICP > 20 mmHg + % Time ΔCPPopt-PRx < -5 mmHg | 0.779 (0.686 - 0.863) | 133.7 | **<0.0001** | 0.327 |
| Core + CT + ICP > 20 mmHg + % Time ΔCPPopt-PRx < -10 mmHg | 0.779 (0.683 - 0.861) | 133.8 | **<0.0001** | 0.327 |
| Core + CT + ICP > 20 mmHg + % Time CPP > ULR-PRx | 0.803 (0.718 - 0.874) | 129.2 | **<0.0001** | 0.371 |
| Core + CT + ICP > 20 mmHg + % Time CPP < LLR-PRx | 0.78 (0.687 - 0.865) | 133.8 | **<0.0001** | 0.326 |
| Core + CT + ICP > 20 mmHg + CPPopt-PAx | 0.801 (0.714 - 0.886) | 132.2 | **<0.0001** | 0.342 |
| Core + CT + ICP > 20 mmHg + % Time ΔCPPopt-PAx > 5 mmHg | 0.791 (0.702 - 0.871) | 130 | **<0.0001** | 0.363 |
| Core + CT + ICP > 20 mmHg + % Time ΔCPPopt-PAx > 10 mmHg | 0.817 (0.732 - 0.889) | 124.7 | **<0.0001** | 0.412 |
| Core + CT + ICP > 20 mmHg + % Time ΔCPPopt-PAx < -5 mmHg | 0.779 (0.688 - 0.861) | 133.8 | **<0.0001** | 0.326 |
| Core + CT + ICP > 20 mmHg + % Time ΔCPPopt-PAx < -10 mmHg | 0.787 (0.697 - 0.866) | 133.4 | **<0.0001** | 0.33 |
| Core + CT + ICP > 20 mmHg + % Time CPP > ULR-PAx | 0.785 (0.687 - 0.865) | 133.2 | **<0.0001** | 0.332 |
| Core + CT + ICP > 20 mmHg + % Time CPP < LLR-PAx | 0.802 (0.72 - 0.881) | 132.4 | **<0.0001** | 0.34 |
| Core + CT + ICP > 20 mmHg + CPPopt-RAC | 0.798 (0.711 - 0.878) | 132.3 | **<0.0001** | 0.341 |
| Core + CT + ICP > 20 mmHg + % Time ΔCPPopt-RAC > 5 mmHg | 0.792 (0.706 - 0.871) | 130.4 | **<0.0001** | 0.359 |
| Core + CT + ICP > 20 mmHg + % Time ΔCPPopt-RAC > 10 mmHg | 0.818 (0.734 - 0.892) | 125.2 | **<0.0001** | 0.407 |
| Core + CT + ICP > 20 mmHg + % Time ΔCPPopt-RAC < -5 mmHg | 0.783 (0.69 - 0.865) | 133.7 | **<0.0001** | 0.328 |
| Core + CT + ICP > 20 mmHg + % Time ΔCPPopt-RAC < -10 mmHg | 0.779 (0.689 - 0.86) | 133.7 | **<0.0001** | 0.327 |
| Core + CT + ICP > 20 mmHg + % Time CPP > ULR-RAC | 0.796 (0.707 - 0.877) | 130.8 | **<0.0001** | 0.356 |
| Core + CT + ICP > 20 mmHg + % Time CPP < LLR-RAC | 0.773 (0.678 - 0.859) | 133.4 | **<0.0001** | 0.33 |
| Core + CT + ICP > 22 mmHg | 0.778 (0.68 - 0.863) | 131.2 | **<0.0001** | 0.332 |
| Core + CT + ICP > 22 mmHg + CPPopt-PRx | 0.796 (0.709 - 0.876) | 132.2 | **<0.0001** | 0.342 |
| Core + CT + ICP > 22 mmHg + % Time ΔCPPopt-PRx > 5 mmHg | 0.799 (0.71 - 0.877) | 130.3 | **<0.0001** | 0.36 |
| Core + CT + ICP > 22 mmHg + % Time ΔCPPopt-PRx > 10 mmHg | 0.815 (0.722 - 0.889) | 127.3 | **<0.0001** | 0.388 |
| Core + CT + ICP > 22 mmHg + % Time ΔCPPopt-PRx < -5 mmHg | 0.781 (0.691 - 0.863) | 133.1 | **<0.0001** | 0.333 |
| Core + CT + ICP > 22 mmHg + % Time ΔCPPopt-PRx < -10 mmHg | 0.778 (0.684 - 0.862) | 133.1 | **<0.0001** | 0.333 |
| Core + CT + ICP > 22 mmHg + % Time CPP > ULR-PRx | 0.805 (0.716 - 0.885) | 128.2 | **<0.0001** | 0.379 |
| Core + CT + ICP > 22 mmHg + % Time CPP < LLR-PRx | 0.778 (0.682 - 0.863) | 133.2 | **<0.0001** | 0.332 |
| Core + CT + ICP > 22 mmHg + CPPopt-PAx | 0.805 (0.719 - 0.879) | 131.6 | **<0.0001** | 0.348 |
| Core + CT + ICP > 22 mmHg + % Time ΔCPPopt-PAx > 5 mmHg | 0.793 (0.699 - 0.872) | 129.6 | **<0.0001** | 0.367 |
| Core + CT + ICP > 22 mmHg + % Time ΔCPPopt-PAx > 10 mmHg | 0.821 (0.741 - 0.893) | 124.1 | **<0.0001** | 0.417 |
| Core + CT + ICP > 22 mmHg + % Time ΔCPPopt-PAx < -5 mmHg | 0.778 (0.689 - 0.857) | 133.2 | **<0.0001** | 0.333 |
| Core + CT + ICP > 22 mmHg + % Time ΔCPPopt-PAx < -10 mmHg | 0.788 (0.7 - 0.868) | 132.8 | **<0.0001** | 0.336 |
| Core + CT + ICP > 22 mmHg + % Time CPP > ULR-PAx | 0.786 (0.695 - 0.868) | 132.6 | **<0.0001** | 0.338 |
| Core + CT + ICP > 22 mmHg + % Time CPP < LLR-PAx | 0.803 (0.72 - 0.881) | 131.8 | **<0.0001** | 0.345 |
| Core + CT + ICP > 22 mmHg + CPPopt-RAC | 0.803 (0.713 - 0.882) | 131.6 | **<0.0001** | 0.347 |
| Core + CT + ICP > 22 mmHg + % Time ΔCPPopt-RAC > 5 mmHg | 0.793 (0.704 - 0.88) | 130.1 | **<0.0001** | 0.362 |
| Core + CT + ICP > 22 mmHg + % Time ΔCPPopt-RAC > 10 mmHg | 0.819 (0.736 - 0.89) | 124.9 | **<0.0001** | 0.41 |
| Core + CT + ICP > 22 mmHg + % Time ΔCPPopt-RAC < -5 mmHg | 0.783 (0.691 - 0.866) | 133.1 | **<0.0001** | 0.334 |
| Core + CT + ICP > 22 mmHg + % Time ΔCPPopt-RAC < -10 mmHg | 0.781 (0.688 - 0.864) | 133.1 | **<0.0001** | 0.333 |
| Core + CT + ICP > 22 mmHg + % Time CPP > ULR-RAC | 0.801 (0.711 - 0.878) | 130 | **<0.0001** | 0.363 |
| Core + CT + ICP > 22 mmHg + % Time CPP < LLR-RAC | 0.778 (0.683 - 0.86) | 132.7 | **<0.0001** | 0.337 |

*Core model consisted of age, admission Glasgow Coma Scale - motor score, and admission pupillary response. CT variables consisted of admission Marshall CT grade, presence of traumatic subarachnoid hemorrhage, and presence of epidural hematoma. All p-values have been adjusted using the False Discovery Rate (FDR) method. Bolded p-values are those reaching statistical significance, p < 0.05.*

*AIC = Akaike information criterion, AMP = pulse amplitude of ICP, AUC = area under the curve, CI = confidence interval, CPP = cerebral perfusion pressure, CPPopt = cerebral perfusion pressure optimum, ΔCPPopt = CPP - CPPopt, CT = computed tomography, ICP = intracranial pressure, IMPACT = International Mission for Prognosis and Analysis of Clinical Trials, mmHg = millimeters of mercury, LLR = lower limit of reactivity, PAx = pulse amplitude index, PRx = pressure reactivity index, RAC = correlation (R) between slow-waves of AMP (A) and CPP (C), ULR = upper limit of reactivity.*

Appendix Q. Multivariable Models of Cerebrovascular Reactivity Measures + IMPACT Core ± CT ± ICP > 20 or 22 mmHg for Transition in Outcome from 1 to 3 Months with Those Who Died (GOSE=1) Removed

| **Model** | **AUC (95% CI)** | **AIC** | **p-value** | **Nagelkerke's R^2^** |
| --- | --- | --- | --- | --- |
| Core | 0.635 (0.493 - 0.768) | 98.6 | **0.0314** | 0.072 |
| Core + CPPopt-PRx | 0.637 (0.496 - 0.765) | 100.4 | **0.0315** | 0.075 |
| Core + % Time ΔCPPopt-PRx > 5 mmHg | 0.635 (0.49 - 0.764) | 100.6 | **0.0311** | 0.072 |
| Core + % Time ΔCPPopt-PRx > 10 mmHg | 0.645 (0.508 - 0.772) | 100.4 | **0.0273** | 0.076 |
| Core + % Time ΔCPPopt-PRx < -5 mmHg | 0.635 (0.496 - 0.771) | 100.6 | **0.0307** | 0.073 |
| Core + % Time ΔCPPopt-PRx < -10 mmHg | 0.643 (0.501 - 0.773) | 100.5 | **0.0275** | 0.074 |
| Core + % Time CPP > ULR-PRx | 0.689 (0.546 - 0.815) | 96.7 | **0.0056** | 0.142 |
| Core + % Time CPP < LLR-PRx | 0.654 (0.513 - 0.785) | 100 | **0.0202** | 0.084 |
| Core + CPPopt-PAx | 0.635 (0.492 - 0.77) | 100.6 | **0.0302** | 0.073 |
| Core + % Time ΔCPPopt-PAx > 5 mmHg | 0.636 (0.501 - 0.764) | 100.5 | **0.0318** | 0.073 |
| Core + % Time ΔCPPopt-PAx > 10 mmHg | 0.638 (0.496 - 0.775) | 100.5 | **0.0311** | 0.073 |
| Core + % Time ΔCPPopt-PAx < -5 mmHg | 0.669 (0.537 - 0.79) | 98.1 | **0.0122** | 0.118 |
| Core + % Time ΔCPPopt-PAx < -10 mmHg | 0.661 (0.527 - 0.79) | 99 | **0.0160** | 0.102 |
| Core + % Time CPP > ULR-PAx | 0.721 (0.6 - 0.838) | 93.1 | **0.0033** | 0.204 |
| Core + % Time CPP < LLR-PAx | 0.653 (0.508 - 0.78) | 98.5 | **0.0211** | 0.111 |
| Core + CPPopt-RAC | 0.636 (0.485 - 0.764) | 100.4 | **0.0313** | 0.075 |
| Core + % Time ΔCPPopt-RAC > 5 mmHg | 0.645 (0.505 - 0.778) | 100.1 | **0.0268** | 0.082 |
| Core + % Time ΔCPPopt-RAC > 10 mmHg | 0.644 (0.497 - 0.775) | 100.5 | **0.0271** | 0.074 |
| Core + % Time ΔCPPopt-RAC < -5 mmHg | 0.664 (0.532 - 0.788) | 97.9 | **0.0147** | 0.122 |
| Core + % Time ΔCPPopt-RAC < -10 mmHg | 0.639 (0.501 - 0.772) | 99.8 | **0.0298** | 0.086 |
| Core + % Time CPP > ULR-RAC | 0.69 (0.556 - 0.812) | 96.6 | **0.0053** | 0.145 |
| Core + % Time CPP < LLR-RAC | 0.641 (0.502 - 0.772) | 100.4 | **0.0286** | 0.075 |
| Core + CT | 0.711 (0.578 - 0.834) | 99.2 | **0.0024** | 0.169 |
| Core + CT + CPPopt-PRx | 0.712 (0.578 - 0.831) | 101.1 | **0.0024** | 0.17 |
| Core + CT + % Time ΔCPPopt-PRx > 5 mmHg | 0.712 (0.581 - 0.839) | 101.2 | **0.0023** | 0.169 |
| Core + CT + % Time ΔCPPopt-PRx > 10 mmHg | 0.708 (0.572 - 0.834) | 100.8 | **0.0025** | 0.176 |
| Core + CT + % Time ΔCPPopt-PRx < -5 mmHg | 0.721 (0.596 - 0.842) | 100.5 | **0.0031** | 0.18 |
| Core + CT + % Time ΔCPPopt-PRx < -10 mmHg | 0.723 (0.586 - 0.84) | 100.3 | **0.0035** | 0.185 |
| Core + CT + % Time CPP > ULR-PRx | 0.747 (0.623 - 0.856) | 97.7 | **0.0044** | 0.228 |
| Core + CT + % Time CPP < LLR-PRx | 0.713 (0.578 - 0.832) | 101 | **0.0023** | 0.171 |
| Core + CT + CPPopt-PAx | 0.714 (0.577 - 0.838) | 101.2 | **0.0025** | 0.169 |
| Core + CT + % Time ΔCPPopt-PAx > 5 mmHg | 0.72 (0.588 - 0.845) | 100.7 | **0.0030** | 0.177 |
| Core + CT + % Time ΔCPPopt-PAx > 10 mmHg | 0.71 (0.576 - 0.833) | 100.7 | **0.0023** | 0.178 |
| Core + CT + % Time ΔCPPopt-PAx < -5 mmHg | 0.738 (0.608 - 0.855) | 99.2 | **0.0038** | 0.203 |
| Core + CT + % Time ΔCPPopt-PAx < -10 mmHg | 0.728 (0.6 - 0.847) | 99.6 | **0.0033** | 0.196 |
| Core + CT + % Time CPP > ULR-PAx | 0.764 (0.65 - 0.87) | 94.1 | **0.0066** | 0.284 |
| Core + CT + % Time CPP < LLR-PAx | 0.719 (0.584 - 0.846) | 99.4 | **0.0030** | 0.199 |
| Core + CT + CPPopt-RAC | 0.72 (0.589 - 0.84) | 101.1 | **0.0029** | 0.17 |
| Core + CT + % Time ΔCPPopt-RAC > 5 mmHg | 0.716 (0.592 - 0.832) | 100.8 | **0.0028** | 0.175 |
| Core + CT + % Time ΔCPPopt-RAC > 10 mmHg | 0.717 (0.574 - 0.837) | 100.7 | **0.0027** | 0.178 |
| Core + CT + % Time ΔCPPopt-RAC < -5 mmHg | 0.735 (0.604 - 0.853) | 98.6 | **0.0033** | 0.212 |
| Core + CT + % Time ΔCPPopt-RAC < -10 mmHg | 0.714 (0.578 - 0.837) | 100.3 | **0.0024** | 0.184 |
| Core + CT + % Time CPP > ULR-RAC | 0.732 (0.607 - 0.848) | 97.6 | **0.0030** | 0.229 |
| Core + CT + % Time CPP < LLR-RAC | 0.72 (0.578 - 0.839) | 101.1 | **0.0028** | 0.171 |
| Core + CT + ICP > 20 mmHg | 0.716 (0.587 - 0.835) | 101 | **0.0023** | 0.172 |
| Core + CT + ICP > 20 mmHg + CPPopt-PRx | 0.714 (0.585 - 0.832) | 103 | **0.0023** | 0.172 |
| Core + CT + ICP > 20 mmHg + % Time ΔCPPopt-PRx > 5 mmHg | 0.715 (0.59 - 0.83) | 103 | **0.0023** | 0.172 |
| Core + CT + ICP > 20 mmHg + % Time ΔCPPopt-PRx > 10 mmHg | 0.712 (0.58 - 0.833) | 102.6 | **0.0023** | 0.178 |
| Core + CT + ICP > 20 mmHg + % Time ΔCPPopt-PRx < -5 mmHg | 0.727 (0.594 - 0.847) | 102.3 | **0.0033** | 0.184 |
| Core + CT + ICP > 20 mmHg + % Time ΔCPPopt-PRx < -10 mmHg | 0.73 (0.604 - 0.843) | 101.9 | **0.0030** | 0.19 |
| Core + CT + ICP > 20 mmHg + % Time CPP > ULR-PRx | 0.746 (0.62 - 0.863) | 99.6 | **0.0033** | 0.229 |
| Core + CT + ICP > 20 mmHg + % Time CPP < LLR-PRx | 0.717 (0.59 - 0.835) | 102.9 | **0.0026** | 0.174 |
| Core + CT + ICP > 20 mmHg + CPPopt-PAx | 0.717 (0.591 - 0.832) | 103 | **0.0025** | 0.172 |
| Core + CT + ICP > 20 mmHg + % Time ΔCPPopt-PAx > 5 mmHg | 0.723 (0.597 - 0.843) | 102.4 | **0.0033** | 0.182 |
| Core + CT + ICP > 20 mmHg + % Time ΔCPPopt-PAx > 10 mmHg | 0.72 (0.585 - 0.835) | 102.4 | **0.0026** | 0.183 |
| Core + CT + ICP > 20 mmHg + % Time ΔCPPopt-PAx < -5 mmHg | 0.744 (0.623 - 0.854) | 101 | **0.0040** | 0.205 |
| Core + CT + ICP > 20 mmHg + % Time ΔCPPopt-PAx < -10 mmHg | 0.735 (0.612 - 0.849) | 101.4 | **0.0029** | 0.2 |
| Core + CT + ICP > 20 mmHg + % Time CPP > ULR-PAx | 0.771 (0.652 - 0.874) | 96.1 | **0.0033** | 0.285 |
| Core + CT + ICP > 20 mmHg + % Time CPP < LLR-PAx | 0.723 (0.595 - 0.851) | 101.4 | **0.0031** | 0.2 |
| Core + CT + ICP > 20 mmHg + CPPopt-RAC | 0.716 (0.593 - 0.831) | 103 | **0.0024** | 0.172 |
| Core + CT + ICP > 20 mmHg + % Time ΔCPPopt-RAC > 5 mmHg | 0.717 (0.596 - 0.832) | 102.7 | **0.0023** | 0.177 |
| Core + CT + ICP > 20 mmHg + % Time ΔCPPopt-RAC > 10 mmHg | 0.712 (0.574 - 0.834) | 102.4 | **0.0022** | 0.181 |
| Core + CT + ICP > 20 mmHg + % Time ΔCPPopt-RAC < -5 mmHg | 0.743 (0.616 - 0.851) | 100.3 | **0.0033** | 0.217 |
| Core + CT + ICP > 20 mmHg + % Time ΔCPPopt-RAC < -10 mmHg | 0.721 (0.591 - 0.84) | 101.9 | **0.0028** | 0.19 |
| Core + CT + ICP > 20 mmHg + % Time CPP > ULR-RAC | 0.735 (0.615 - 0.848) | 99.6 | **0.0026** | 0.229 |
| Core + CT + ICP > 20 mmHg + % Time CPP < LLR-RAC | 0.715 (0.582 - 0.827) | 102.9 | **0.0022** | 0.173 |
| Core + CT + ICP > 22 mmHg | 0.729 (0.602 - 0.85) | 100.2 | **0.0017** | 0.186 |
| Core + CT + ICP > 22 mmHg + CPPopt-PRx | 0.728 (0.609 - 0.843) | 102.2 | **0.0016** | 0.186 |
| Core + CT + ICP > 22 mmHg + % Time ΔCPPopt-PRx > 5 mmHg | 0.728 (0.6 - 0.849) | 102.2 | **0.0016** | 0.186 |
| Core + CT + ICP > 22 mmHg + % Time ΔCPPopt-PRx > 10 mmHg | 0.731 (0.607 - 0.847) | 101.6 | **0.0016** | 0.195 |
| Core + CT + ICP > 22 mmHg + % Time ΔCPPopt-PRx < -5 mmHg | 0.736 (0.61 - 0.844) | 101.4 | **0.0016** | 0.199 |
| Core + CT + ICP > 22 mmHg + % Time ΔCPPopt-PRx < -10 mmHg | 0.741 (0.619 - 0.855) | 101 | **0.0016** | 0.206 |
| Core + CT + ICP > 22 mmHg + % Time CPP > ULR-PRx | 0.75 (0.625 - 0.862) | 99.1 | **0.0016** | 0.237 |
| Core + CT + ICP > 22 mmHg + % Time CPP < LLR-PRx | 0.728 (0.609 - 0.848) | 102 | **0.0016** | 0.189 |
| Core + CT + ICP > 22 mmHg + CPPopt-PAx | 0.729 (0.608 - 0.843) | 102.2 | **0.0016** | 0.186 |
| Core + CT + ICP > 22 mmHg + % Time ΔCPPopt-PAx > 5 mmHg | 0.733 (0.608 - 0.848) | 101.5 | **0.0016** | 0.197 |
| Core + CT + ICP > 22 mmHg + % Time ΔCPPopt-PAx > 10 mmHg | 0.736 (0.62 - 0.847) | 101.2 | **0.0016** | 0.203 |
| Core + CT + ICP > 22 mmHg + % Time ΔCPPopt-PAx < -5 mmHg | 0.748 (0.63 - 0.862) | 100.2 | **0.0016** | 0.219 |
| Core + CT + ICP > 22 mmHg + % Time ΔCPPopt-PAx < -10 mmHg | 0.739 (0.614 - 0.855) | 100.4 | **0.0016** | 0.215 |
| Core + CT + ICP > 22 mmHg + % Time CPP > ULR-PAx | 0.765 (0.642 - 0.865) | 96 | **0.0016** | 0.286 |
| Core + CT + ICP > 22 mmHg + % Time CPP < LLR-PAx | 0.737 (0.616 - 0.857) | 100.7 | **0.0016** | 0.211 |
| Core + CT + ICP > 22 mmHg + CPPopt-RAC | 0.73 (0.609 - 0.843) | 102 | **0.0016** | 0.189 |
| Core + CT + ICP > 22 mmHg + % Time ΔCPPopt-RAC > 5 mmHg | 0.735 (0.601 - 0.841) | 101.8 | **0.0016** | 0.193 |
| Core + CT + ICP > 22 mmHg + % Time ΔCPPopt-RAC > 10 mmHg | 0.734 (0.602 - 0.843) | 101.5 | **0.0016** | 0.197 |
| Core + CT + ICP > 22 mmHg + % Time ΔCPPopt-RAC < -5 mmHg | 0.752 (0.621 - 0.859) | 99.3 | **0.0016** | 0.234 |
| Core + CT + ICP > 22 mmHg + % Time ΔCPPopt-RAC < -10 mmHg | 0.74 (0.62 - 0.852) | 100.9 | **0.0016** | 0.207 |
| Core + CT + ICP > 22 mmHg + % Time CPP > ULR-RAC | 0.738 (0.619 - 0.848) | 99.1 | **0.0016** | 0.237 |
| Core + CT + ICP > 22 mmHg + % Time CPP < LLR-RAC | 0.731 (0.608 - 0.845) | 102.1 | **0.0016** | 0.187 |

*Core model consisted of age, admission Glasgow Coma Scale - motor score, and admission pupillary response. CT variables consisted of admission Marshall CT grade, presence of traumatic subarachnoid hemorrhage, and presence of epidural hematoma. All p-values have been adjusted using the False Discovery Rate (FDR) method. Bolded p-values are those reaching statistical significance, p < 0.05.*

*AIC = Akaike information criterion, AMP = pulse amplitude of ICP, AUC = area under the curve, CI = confidence interval, CPP = cerebral perfusion pressure, CPPopt = cerebral perfusion pressure optimum, ΔCPPopt = CPP - CPPopt, CT = computed tomography, GOSE = Glasgow Outcome Scale-Extended, ICP = intracranial pressure, IMPACT = International Mission for Prognosis and Analysis of Clinical Trials, mmHg = millimeters of mercury, LLR = lower limit of reactivity, PAx = pulse amplitude index, PRx = pressure reactivity index, RAC = correlation (R) between slow-waves of AMP (A) and CPP (C), ULR = upper limit of reactivity.*

Appendix R. Multivariable Models of Cerebrovascular Reactivity Measures + IMPACT Core ± CT ± ICP > 20 or 22 mmHg for Transition in Outcome from 3 to 6 Months with Those Who Died (GOSE=1) Removed

| **Model** | **AUC (95% CI)** | **AIC** | **p-value** | **Nagelkerke's R^2^** |
| --- | --- | --- | --- | --- |
| Core | 0.577 (0.434 - 0.711) | 100.7 | 0.1501 | 0.028 |
| Core + CPPopt-PRx | 0.626 (0.489 - 0.754) | 100.6 | 0.0590 | 0.069 |
| Core + % Time ΔCPPopt-PRx > 5 mmHg | 0.604 (0.463 - 0.732) | 101.8 | 0.0866 | 0.046 |
| Core + % Time ΔCPPopt-PRx > 10 mmHg | 0.593 (0.453 - 0.734) | 101.9 | 0.1069 | 0.044 |
| Core + % Time ΔCPPopt-PRx < -5 mmHg | 0.572 (0.287 - 0.571) | 102.3 | 0.1616 | 0.036 |
| Core + % Time ΔCPPopt-PRx < -10 mmHg | 0.564 (0.302 - 0.577) | 102.7 | 0.1882 | 0.03 |
| Core + % Time CPP > ULR-PRx | 0.648 (0.509 - 0.775) | 96 | **0.0344** | 0.153 |
| Core + % Time CPP < LLR-PRx | 0.594 (0.452 - 0.732) | 102 | 0.1063 | 0.041 |
| Core + CPPopt-PAx | 0.596 (0.455 - 0.733) | 101.8 | 0.1010 | 0.047 |
| Core + % Time ΔCPPopt-PAx > 5 mmHg | 0.622 (0.487 - 0.755) | 99.8 | 0.0632 | 0.084 |
| Core + % Time ΔCPPopt-PAx > 10 mmHg | 0.614 (0.473 - 0.743) | 100.3 | 0.0691 | 0.074 |
| Core + % Time ΔCPPopt-PAx < -5 mmHg | 0.648 (0.509 - 0.775) | 99.3 | **0.0335** | 0.094 |
| Core + % Time ΔCPPopt-PAx < -10 mmHg | 0.618 (0.477 - 0.748) | 100.7 | 0.0675 | 0.068 |
| Core + % Time CPP > ULR-PAx | 0.739 (0.608 - 0.847) | 91.9 | **0.0099** | 0.224 |
| Core + % Time CPP < LLR-PAx | 0.617 (0.479 - 0.755) | 101.4 | 0.0665 | 0.053 |
| Core + CPPopt-RAC | 0.587 (0.443 - 0.725) | 101.9 | 0.1200 | 0.044 |
| Core + % Time ΔCPPopt-RAC > 5 mmHg | 0.629 (0.493 - 0.763) | 100.6 | 0.0554 | 0.07 |
| Core + % Time ΔCPPopt-RAC > 10 mmHg | 0.608 (0.47 - 0.748) | 101.8 | 0.0781 | 0.046 |
| Core + % Time ΔCPPopt-RAC < -5 mmHg | 0.598 (0.459 - 0.735) | 102 | 0.0981 | 0.042 |
| Core + % Time ΔCPPopt-RAC < -10 mmHg | 0.574 (0.431 - 0.718) | 102.7 | 0.1580 | 0.029 |
| Core + % Time CPP > ULR-RAC | 0.705 (0.566 - 0.825) | 94.5 | **0.0157** | 0.18 |
| Core + % Time CPP < LLR-RAC | 0.677 (0.545 - 0.802) | 99.1 | **0.0238** | 0.097 |
| Core + CT | 0.609 (0.468 - 0.748) | 104.6 | 0.0762 | 0.069 |
| Core + CT + CPPopt-PRx | 0.646 (0.512 - 0.777) | 104.6 | **0.0349** | 0.106 |
| Core + CT + % Time ΔCPPopt-PRx > 5 mmHg | 0.653 (0.522 - 0.78) | 105.7 | **0.0320** | 0.086 |
| Core + CT + % Time ΔCPPopt-PRx > 10 mmHg | 0.641 (0.504 - 0.765) | 106 | **0.0396** | 0.081 |
| Core + CT + % Time ΔCPPopt-PRx < -5 mmHg | 0.636 (0.505 - 0.766) | 105.9 | **0.0451** | 0.083 |
| Core + CT + % Time ΔCPPopt-PRx < -10 mmHg | 0.616 (0.477 - 0.751) | 106.3 | 0.0669 | 0.075 |
| Core + CT + % Time CPP > ULR-PRx | 0.704 (0.565 - 0.823) | 99.2 | **0.0147** | 0.201 |
| Core + CT + % Time CPP < LLR-PRx | 0.618 (0.474 - 0.749) | 106.2 | 0.0661 | 0.076 |
| Core + CT + CPPopt-PAx | 0.625 (0.489 - 0.76) | 105.7 | 0.0580 | 0.086 |
| Core + CT + % Time ΔCPPopt-PAx > 5 mmHg | 0.683 (0.551 - 0.798) | 103.2 | **0.0254** | 0.131 |
| Core + CT + % Time ΔCPPopt-PAx > 10 mmHg | 0.661 (0.522 - 0.785) | 103.9 | **0.0281** | 0.118 |
| Core + CT + % Time ΔCPPopt-PAx < -5 mmHg | 0.677 (0.552 - 0.798) | 102.4 | **0.0260** | 0.145 |
| Core + CT + % Time ΔCPPopt-PAx < -10 mmHg | 0.652 (0.511 - 0.781) | 104 | **0.0311** | 0.116 |
| Core + CT + % Time CPP > ULR-PAx | 0.734 (0.61 - 0.841) | 96.5 | **0.0088** | 0.247 |
| Core + CT + % Time CPP < LLR-PAx | 0.626 (0.493 - 0.756) | 106.1 | 0.0576 | 0.079 |
| Core + CT + CPPopt-RAC | 0.62 (0.474 - 0.752) | 105.8 | 0.0653 | 0.083 |
| Core + CT + % Time ΔCPPopt-RAC > 5 mmHg | 0.677 (0.537 - 0.8) | 104.1 | **0.0245** | 0.115 |
| Core + CT + % Time ΔCPPopt-RAC > 10 mmHg | 0.649 (0.504 - 0.774) | 105.7 | **0.0343** | 0.086 |
| Core + CT + % Time ΔCPPopt-RAC < -5 mmHg | 0.641 (0.504 - 0.769) | 105.5 | **0.0398** | 0.089 |
| Core + CT + % Time ΔCPPopt-RAC < -10 mmHg | 0.612 (0.475 - 0.743) | 106.5 | 0.0733 | 0.071 |
| Core + CT + % Time CPP > ULR-RAC | 0.714 (0.585 - 0.835) | 98.8 | **0.0132** | 0.209 |
| Core + CT + % Time CPP < LLR-RAC | 0.667 (0.532 - 0.794) | 103.5 | **0.0244** | 0.126 |
| Core + CT + ICP > 20 mmHg | 0.654 (0.52 - 0.783) | 103.2 | **0.0311** | 0.133 |
| Core + CT + ICP > 20 mmHg + CPPopt-PRx | 0.672 (0.538 - 0.792) | 103.7 | **0.0234** | 0.158 |
| Core + CT + ICP > 20 mmHg + % Time ΔCPPopt-PRx > 5 mmHg | 0.677 (0.541 - 0.806) | 104.5 | **0.0226** | 0.145 |
| Core + CT + ICP > 20 mmHg + % Time ΔCPPopt-PRx > 10 mmHg | 0.668 (0.531 - 0.793) | 104.7 | **0.0248** | 0.141 |
| Core + CT + ICP > 20 mmHg + % Time ΔCPPopt-PRx < -5 mmHg | 0.664 (0.533 - 0.795) | 104.1 | **0.0262** | 0.152 |
| Core + CT + ICP > 20 mmHg + % Time ΔCPPopt-PRx < -10 mmHg | 0.673 (0.545 - 0.803) | 104.2 | **0.0236** | 0.15 |
| Core + CT + ICP > 20 mmHg + % Time CPP > ULR-PRx | 0.73 (0.6 - 0.844) | 97.2 | **0.0066** | 0.268 |
| Core + CT + ICP > 20 mmHg + % Time CPP < LLR-PRx | 0.661 (0.521 - 0.786) | 104.7 | **0.0271** | 0.141 |
| Core + CT + ICP > 20 mmHg + CPPopt-PAx | 0.67 (0.536 - 0.795) | 104.3 | **0.0241** | 0.148 |
| Core + CT + ICP > 20 mmHg + % Time ΔCPPopt-PAx > 5 mmHg | 0.698 (0.561 - 0.819) | 103.1 | **0.0156** | 0.17 |
| Core + CT + ICP > 20 mmHg + % Time ΔCPPopt-PAx > 10 mmHg | 0.682 (0.546 - 0.803) | 103.6 | **0.0245** | 0.16 |
| Core + CT + ICP > 20 mmHg + % Time ΔCPPopt-PAx < -5 mmHg | 0.707 (0.574 - 0.82) | 101 | **0.0160** | 0.206 |
| Core + CT + ICP > 20 mmHg + % Time ΔCPPopt-PAx < -10 mmHg | 0.704 (0.571 - 0.82) | 102.1 | **0.0132** | 0.187 |
| Core + CT + ICP > 20 mmHg + % Time CPP > ULR-PAx | 0.755 (0.629 - 0.863) | 97 | **0.0066** | 0.27 |
| Core + CT + ICP > 20 mmHg + % Time CPP < LLR-PAx | 0.656 (0.522 - 0.787) | 105 | **0.0299** | 0.135 |
| Core + CT + ICP > 20 mmHg + CPPopt-RAC | 0.659 (0.521 - 0.784) | 104.5 | **0.0288** | 0.145 |
| Core + CT + ICP > 20 mmHg + % Time ΔCPPopt-RAC > 5 mmHg | 0.687 (0.554 - 0.81) | 103.3 | **0.0226** | 0.165 |
| Core + CT + ICP > 20 mmHg + % Time ΔCPPopt-RAC > 10 mmHg | 0.657 (0.514 - 0.783) | 104.7 | **0.0298** | 0.141 |
| Core + CT + ICP > 20 mmHg + % Time ΔCPPopt-RAC < -5 mmHg | 0.676 (0.542 - 0.804) | 103.8 | **0.0224** | 0.156 |
| Core + CT + ICP > 20 mmHg + % Time ΔCPPopt-RAC < -10 mmHg | 0.668 (0.541 - 0.79) | 104.7 | **0.0246** | 0.141 |
| Core + CT + ICP > 20 mmHg + % Time CPP > ULR-RAC | 0.737 (0.617 - 0.841) | 98.7 | **0.0066** | 0.244 |
| Core + CT + ICP > 20 mmHg + % Time CPP < LLR-RAC | 0.682 (0.547 - 0.804) | 102.6 | **0.0229** | 0.177 |
| Core + CT + ICP > 22 mmHg | 0.668 (0.532 - 0.789) | 102.3 | **0.0176** | 0.148 |
| Core + CT + ICP > 22 mmHg + CPPopt-PRx | 0.682 (0.546 - 0.802) | 103.2 | **0.0153** | 0.167 |
| Core + CT + ICP > 22 mmHg + % Time ΔCPPopt-PRx > 5 mmHg | 0.683 (0.549 - 0.804) | 103.7 | **0.0153** | 0.159 |
| Core + CT + ICP > 22 mmHg + % Time ΔCPPopt-PRx > 10 mmHg | 0.677 (0.545 - 0.803) | 103.9 | **0.0158** | 0.154 |
| Core + CT + ICP > 22 mmHg + % Time ΔCPPopt-PRx < -5 mmHg | 0.681 (0.551 - 0.808) | 103.0 | **0.0153** | 0.17 |
| Core + CT + ICP > 22 mmHg + % Time ΔCPPopt-PRx < -10 mmHg | 0.687 (0.553 - 0.811) | 103.2 | **0.0153** | 0.167 |
| Core + CT + ICP > 22 mmHg + % Time CPP > ULR-PRx | 0.732 (0.605 - 0.842) | 97.0 | **0.0055** | 0.27 |
| Core + CT + ICP > 22 mmHg + % Time CPP < LLR-PRx | 0.675 (0.538 - 0.803) | 103.9 | **0.0158** | 0.155 |
| Core + CT + ICP > 22 mmHg + CPPopt-PAx | 0.685 (0.549 - 0.806) | 103.7 | **0.0153** | 0.159 |
| Core + CT + ICP > 22 mmHg + % Time ΔCPPopt-PAx > 5 mmHg | 0.708 (0.579 - 0.826) | 102 | **0.0110** | 0.189 |
| Core + CT + ICP > 22 mmHg + % Time ΔCPPopt-PAx > 10 mmHg | 0.689 (0.556 - 0.814) | 102.9 | **0.0153** | 0.173 |
| Core + CT + ICP > 22 mmHg + % Time ΔCPPopt-PAx < -5 mmHg | 0.716 (0.587 - 0.826) | 99.8 | **0.0106** | 0.226 |
| Core + CT + ICP > 22 mmHg + % Time ΔCPPopt-PAx < -10 mmHg | 0.71 (0.583 - 0.825) | 101 | **0.0110** | 0.205 |
| Core + CT + ICP > 22 mmHg + % Time CPP > ULR-PAx | 0.765 (0.648 - 0.875) | 96.5 | **0.0044** | 0.279 |
| Core + CT + ICP > 22 mmHg + % Time CPP < LLR-PAx | 0.668 (0.531 - 0.793) | 104.2 | **0.0176** | 0.15 |
| Core + CT + ICP > 22 mmHg + CPPopt-RAC | 0.684 (0.546 - 0.805) | 103.8 | **0.0153** | 0.156 |
| Core + CT + ICP > 22 mmHg + % Time ΔCPPopt-RAC > 5 mmHg | 0.695 (0.562 - 0.816) | 102.2 | **0.0142** | 0.185 |
| Core + CT + ICP > 22 mmHg + % Time ΔCPPopt-RAC > 10 mmHg | 0.669 (0.528 - 0.789) | 103.8 | **0.0176** | 0.156 |
| Core + CT + ICP > 22 mmHg + % Time ΔCPPopt-RAC < -5 mmHg | 0.684 (0.551 - 0.801) | 102.9 | **0.0153** | 0.173 |
| Core + CT + ICP > 22 mmHg + % Time ΔCPPopt-RAC < -10 mmHg | 0.675 (0.542 - 0.802) | 103.8 | **0.0158** | 0.157 |
| Core + CT + ICP > 22 mmHg + % Time CPP > ULR-RAC | 0.743 (0.626 - 0.854) | 98.1 | **0.0055** | 0.253 |
| Core + CT + ICP > 22 mmHg + % Time CPP < LLR-RAC | 0.694 (0.559 - 0.823) | 101.8 | **0.0144** | 0.191 |

*Core model consisted of age, admission Glasgow Coma Scale - motor score, and admission pupillary response. CT variables consisted of admission Marshall CT grade, presence of traumatic subarachnoid hemorrhage, and presence of epidural hematoma. All p-values have been adjusted using the False Discovery Rate (FDR) method. Bolded p-values are those reaching statistical significance, p < 0.05.*

*AIC = Akaike information criterion, AMP = pulse amplitude of ICP, AUC = area under the curve, CI = confidence interval, CPP = cerebral perfusion pressure, CPPopt = cerebral perfusion pressure optimum, ΔCPPopt = CPP - CPPopt, CT = computed tomography, GOSE = Glasgow Outcome Scale-Extended, ICP = intracranial pressure, IMPACT = International Mission for Prognosis and Analysis of Clinical Trials, mmHg = millimeters of mercury, LLR = lower limit of reactivity, PAx = pulse amplitude index, PRx = pressure reactivity index, RAC = correlation (R) between slow-waves of AMP (A) and CPP (C), ULR = upper limit of reactivity.*

Appendix S. Multivariable Models of Cerebrovascular Reactivity Measures + IMPACT Core ± CT ± ICP > 20 or 22 mmHg for Transition in Outcome from 1 to 6 Months with Those Who Died (GOSE=1) Removed

| **Model** | **AUC (95% CI)** | **AIC** | **p-value** | **Nagelkerke's R^2^** |
| --- | --- | --- | --- | --- |
| Core | 0.723 (0.59 - 0.844) | 77.3 | **0.0040** | 0.155 |
| Core + CPPopt-PRx | 0.722 (0.575 - 0.848) | 79 | **0.0040** | 0.16 |
| Core + % Time ΔCPPopt-PRx > 5 mmHg | 0.745 (0.606 - 0.862) | 77.7 | **0.0023** | 0.186 |
| Core + % Time ΔCPPopt-PRx > 10 mmHg | 0.736 (0.596 - 0.853) | 77.3 | **0.0032** | 0.194 |
| Core + % Time ΔCPPopt-PRx < -5 mmHg | 0.728 (0.585 - 0.861) | 78 | **0.0039** | 0.179 |
| Core + % Time ΔCPPopt-PRx < -10 mmHg | 0.723 (0.58 - 0.847) | 78.8 | **0.0040** | 0.164 |
| Core + % Time CPP > ULR-PRx | 0.724 (0.583 - 0.849) | 78.8 | **0.0041** | 0.164 |
| Core + % Time CPP < LLR-PRx | 0.724 (0.581 - 0.851) | 77.9 | **0.0041** | 0.181 |
| Core + CPPopt-PAx | 0.742 (0.59 - 0.873) | 77.6 | **0.0026** | 0.187 |
| Core + % Time ΔCPPopt-PAx > 5 mmHg | 0.726 (0.595 - 0.843) | 78.4 | **0.0039** | 0.173 |
| Core + % Time ΔCPPopt-PAx > 10 mmHg | 0.732 (0.599 - 0.85) | 77.8 | **0.0034** | 0.184 |
| Core + % Time ΔCPPopt-PAx < -5 mmHg | 0.719 (0.578 - 0.846) | 79.1 | **0.0043** | 0.159 |
| Core + % Time ΔCPPopt-PAx < -10 mmHg | 0.724 (0.592 - 0.853) | 79 | **0.0040** | 0.16 |
| Core + % Time CPP > ULR-PAx | 0.722 (0.578 - 0.854) | 79.1 | **0.0040** | 0.157 |
| Core + % Time CPP < LLR-PAx | 0.715 (0.573 - 0.84) | 79.2 | **0.0049** | 0.156 |
| Core + CPPopt-RAC | 0.735 (0.586 - 0.868) | 77.9 | **0.0032** | 0.181 |
| Core + % Time ΔCPPopt-RAC > 5 mmHg | 0.722 (0.586 - 0.842) | 79.1 | **0.0039** | 0.159 |
| Core + % Time ΔCPPopt-RAC > 10 mmHg | 0.728 (0.593 - 0.841) | 77.7 | **0.0039** | 0.185 |
| Core + % Time ΔCPPopt-RAC < -5 mmHg | 0.724 (0.579 - 0.856) | 79.3 | **0.0039** | 0.155 |
| Core + % Time ΔCPPopt-RAC < -10 mmHg | 0.725 (0.583 - 0.854) | 79.3 | **0.0041** | 0.155 |
| Core + % Time CPP > ULR-RAC | 0.736 (0.604 - 0.861) | 78 | **0.0031** | 0.18 |
| Core + % Time CPP < LLR-RAC | 0.739 (0.595 - 0.864) | 78 | **0.0028** | 0.179 |
| Core + CT | 0.814 (0.674 - 0.929) | 73.2 | **0.0002** | 0.339 |
| Core + CT + CPPopt-PRx | 0.836 (0.706 - 0.945) | 74.5 | **<0.0001** | 0.352 |
| Core + CT + % Time ΔCPPopt-PRx > 5 mmHg | 0.828 (0.703 - 0.93) | 73.6 | **<0.0001** | 0.367 |
| Core + CT + % Time ΔCPPopt-PRx > 10 mmHg | 0.842 (0.725 - 0.935) | 72.6 | **<0.0001** | 0.382 |
| Core + CT + % Time ΔCPPopt-PRx < -5 mmHg | 0.814 (0.684 - 0.927) | 75 | **0.0002** | 0.342 |
| Core + CT + % Time ΔCPPopt-PRx < -10 mmHg | 0.812 (0.678 - 0.925) | 75.2 | **0.0002** | 0.339 |
| Core + CT + % Time CPP > ULR-PRx | 0.831 (0.709 - 0.936) | 74.3 | **<0.0001** | 0.354 |
| Core + CT + % Time CPP < LLR-PRx | 0.825 (0.695 - 0.935) | 74.7 | **<0.0001** | 0.348 |
| Core + CT + CPPopt-PAx | 0.841 (0.713 - 0.943) | 74 | **<0.0001** | 0.36 |
| Core + CT + % Time ΔCPPopt-PAx > 5 mmHg | 0.836 (0.704 - 0.945) | 72.7 | **<0.0001** | 0.381 |
| Core + CT + % Time ΔCPPopt-PAx > 10 mmHg | 0.843 (0.72 - 0.94) | 71.5 | **<0.0001** | 0.401 |
| Core + CT + % Time ΔCPPopt-PAx < -5 mmHg | 0.814 (0.678 - 0.926) | 75.2 | **0.0002** | 0.339 |
| Core + CT + % Time ΔCPPopt-PAx < -10 mmHg | 0.823 (0.693 - 0.927) | 75.1 | **0.0002** | 0.342 |
| Core + CT + % Time CPP > ULR-PAx | 0.822 (0.69 - 0.933) | 74.9 | **0.0002** | 0.344 |
| Core + CT + % Time CPP < LLR-PAx | 0.814 (0.679 - 0.928) | 75.2 | **0.0002** | 0.34 |
| Core + CT + CPPopt-RAC | 0.842 (0.718 - 0.948) | 74.1 | **<0.0001** | 0.357 |
| Core + CT + % Time ΔCPPopt-RAC > 5 mmHg | 0.822 (0.691 - 0.926) | 74.7 | **0.0002** | 0.348 |
| Core + CT + % Time ΔCPPopt-RAC > 10 mmHg | 0.842 (0.73 - 0.929) | 71.9 | **<0.0001** | 0.394 |
| Core + CT + % Time ΔCPPopt-RAC < -5 mmHg | 0.812 (0.683 - 0.925) | 75.1 | **0.0002** | 0.34 |
| Core + CT + % Time ΔCPPopt-RAC < -10 mmHg | 0.815 (0.688 - 0.935) | 75.2 | **0.0002** | 0.339 |
| Core + CT + % Time CPP > ULR-RAC | 0.843 (0.724 - 0.94) | 73.5 | **<0.0001** | 0.368 |
| Core + CT + % Time CPP < LLR-RAC | 0.83 (0.705 - 0.934) | 73.6 | **<0.0001** | 0.367 |
| Core + CT + ICP > 20 mmHg | 0.824 (0.702 - 0.923) | 73.4 | **<0.0001** | 0.37 |
| Core + CT + ICP > 20 mmHg + CPPopt-PRx | 0.851 (0.743 - 0.943) | 74.5 | **<0.0001** | 0.384 |
| Core + CT + ICP > 20 mmHg + % Time ΔCPPopt-PRx > 5 mmHg | 0.854 (0.751 - 0.938) | 73.7 | **<0.0001** | 0.397 |
| Core + CT + ICP > 20 mmHg + % Time ΔCPPopt-PRx > 10 mmHg | 0.85 (0.744 - 0.938) | 72.6 | **<0.0001** | 0.415 |
| Core + CT + ICP > 20 mmHg + % Time ΔCPPopt-PRx < -5 mmHg | 0.827 (0.711 - 0.922) | 75 | **<0.0001** | 0.376 |
| Core + CT + ICP > 20 mmHg + % Time ΔCPPopt-PRx < -10 mmHg | 0.825 (0.693 - 0.928) | 75.3 | **<0.0001** | 0.371 |
| Core + CT + ICP > 20 mmHg + % Time CPP > ULR-PRx | 0.851 (0.748 - 0.939) | 74.3 | **<0.0001** | 0.388 |
| Core + CT + ICP > 20 mmHg + % Time CPP < LLR-PRx | 0.827 (0.708 - 0.929) | 74.9 | **<0.0001** | 0.378 |
| Core + CT + ICP > 20 mmHg + CPPopt-PAx | 0.86 (0.758 - 0.945) | 73.7 | **<0.0001** | 0.397 |
| Core + CT + ICP > 20 mmHg + % Time ΔCPPopt-PAx > 5 mmHg | 0.848 (0.73 - 0.941) | 73.3 | **<0.0001** | 0.404 |
| Core + CT + ICP > 20 mmHg + % Time ΔCPPopt-PAx > 10 mmHg | 0.86 (0.748 - 0.948) | 72 | **<0.0001** | 0.425 |
| Core + CT + ICP > 20 mmHg + % Time ΔCPPopt-PAx < -5 mmHg | 0.824 (0.696 - 0.926) | 75.4 | **<0.0001** | 0.37 |
| Core + CT + ICP > 20 mmHg + % Time ΔCPPopt-PAx < -10 mmHg | 0.825 (0.713 - 0.927) | 75.3 | **<0.0001** | 0.371 |
| Core + CT + ICP > 20 mmHg + % Time CPP > ULR-PAx | 0.825 (0.697 - 0.923) | 75.2 | **<0.0001** | 0.372 |
| Core + CT + ICP > 20 mmHg + % Time CPP < LLR-PAx | 0.816 (0.692 - 0.925) | 75.2 | **0.0002** | 0.373 |
| Core + CT + ICP > 20 mmHg + CPPopt-RAC | 0.855 (0.751 - 0.945) | 73.9 | **<0.0001** | 0.394 |
| Core + CT + ICP > 20 mmHg + % Time ΔCPPopt-RAC > 5 mmHg | 0.824 (0.709 - 0.925) | 75 | **<0.0001** | 0.376 |
| Core + CT + ICP > 20 mmHg + % Time ΔCPPopt-RAC > 10 mmHg | 0.857 (0.755 - 0.936) | 72.3 | **<0.0001** | 0.42 |
| Core + CT + ICP > 20 mmHg + % Time ΔCPPopt-RAC < -5 mmHg | 0.824 (0.705 - 0.926) | 75.3 | **<0.0001** | 0.372 |
| Core + CT + ICP > 20 mmHg + % Time ΔCPPopt-RAC < -10 mmHg | 0.823 (0.699 - 0.926) | 75.4 | **0.0002** | 0.37 |
| Core + CT + ICP > 20 mmHg + % Time CPP > ULR-RAC | 0.849 (0.743 - 0.934) | 73.7 | **<0.0001** | 0.398 |
| Core + CT + ICP > 20 mmHg + % Time CPP < LLR-RAC | 0.84 (0.728 - 0.933) | 74.1 | **<0.0001** | 0.39 |
| Core + CT + ICP > 22 mmHg | 0.825 (0.699 - 0.923) | 73.6 | **<0.0001** | 0.366 |
| Core + CT + ICP > 22 mmHg + CPPopt-PRx | 0.847 (0.736 - 0.94) | 74.9 | **<0.0001** | 0.378 |
| Core + CT + ICP > 22 mmHg + % Time ΔCPPopt-PRx > 5 mmHg | 0.85 (0.742 - 0.941) | 74 | **<0.0001** | 0.393 |
| Core + CT + ICP > 22 mmHg + % Time ΔCPPopt-PRx > 10 mmHg | 0.85 (0.743 - 0.935) | 72.9 | **<0.0001** | 0.411 |
| Core + CT + ICP > 22 mmHg + % Time ΔCPPopt-PRx < -5 mmHg | 0.824 (0.699 - 0.926) | 75.2 | **<0.0001** | 0.372 |
| Core + CT + ICP > 22 mmHg + % Time ΔCPPopt-PRx < -10 mmHg | 0.828 (0.702 - 0.927) | 75.6 | **<0.0001** | 0.367 |
| Core + CT + ICP > 22 mmHg + % Time CPP > ULR-PRx | 0.853 (0.743 - 0.941) | 74.6 | **<0.0001** | 0.382 |
| Core + CT + ICP > 22 mmHg + % Time CPP < LLR-PRx | 0.83 (0.71 - 0.932) | 75 | **<0.0001** | 0.376 |
| Core + CT + ICP > 22 mmHg + CPPopt-PAx | 0.86 (0.75 - 0.948) | 74.1 | **<0.0001** | 0.391 |
| Core + CT + ICP > 22 mmHg + % Time ΔCPPopt-PAx > 5 mmHg | 0.844 (0.728 - 0.94) | 73.4 | **<0.0001** | 0.402 |
| Core + CT + ICP > 22 mmHg + % Time ΔCPPopt-PAx > 10 mmHg | 0.854 (0.739 - 0.942) | 72.2 | **<0.0001** | 0.421 |
| Core + CT + ICP > 22 mmHg + % Time ΔCPPopt-PAx < -5 mmHg | 0.825 (0.703 - 0.928) | 75.6 | **<0.0001** | 0.366 |
| Core + CT + ICP > 22 mmHg + % Time ΔCPPopt-PAx < -10 mmHg | 0.824 (0.708 - 0.927) | 75.5 | **<0.0001** | 0.367 |
| Core + CT + ICP > 22 mmHg + % Time CPP > ULR-PAx | 0.83 (0.71 - 0.932) | 75.4 | **<0.0001** | 0.369 |
| Core + CT + ICP > 22 mmHg + % Time CPP < LLR-PAx | 0.82 (0.699 - 0.925) | 75.4 | **0.0001** | 0.369 |
| Core + CT + ICP > 22 mmHg + CPPopt-RAC | 0.85 (0.743 - 0.942) | 74.3 | **<0.0001** | 0.388 |
| Core + CT + ICP > 22 mmHg + % Time ΔCPPopt-RAC > 5 mmHg | 0.827 (0.709 - 0.927) | 75.2 | **<0.0001** | 0.373 |
| Core + CT + ICP > 22 mmHg + % Time ΔCPPopt-RAC > 10 mmHg | 0.86 (0.759 - 0.94) | 72.4 | **<0.0001** | 0.418 |
| Core + CT + ICP > 22 mmHg + % Time ΔCPPopt-RAC < -5 mmHg | 0.823 (0.698 - 0.926) | 75.5 | **0.0001** | 0.368 |
| Core + CT + ICP > 22 mmHg + % Time ΔCPPopt-RAC < -10 mmHg | 0.824 (0.709 - 0.922) | 75.6 | **<0.0001** | 0.366 |
| Core + CT + ICP > 22 mmHg + % Time CPP > ULR-RAC | 0.841 (0.739 - 0.93) | 73.9 | **<0.0001** | 0.394 |
| Core + CT + ICP > 22 mmHg + % Time CPP < LLR-RAC | 0.838 (0.721 - 0.936) | 74.3 | **<0.0001** | 0.388 |

*Core model consisted of age, admission Glasgow Coma Scale - motor score, and admission pupillary response. CT variables consisted of admission Marshall CT grade, presence of traumatic subarachnoid hemorrhage, and presence of epidural hematoma. All p-values have been adjusted using the False Discovery Rate (FDR) method. Bolded p-values are those reaching statistical significance, p < 0.05.*

*AIC = Akaike information criterion, AMP = pulse amplitude of ICP, AUC = area under the curve, CI = confidence interval, CPP = cerebral perfusion pressure, CPPopt = cerebral perfusion pressure optimum, ΔCPPopt = CPP - CPPopt, CT = computed tomography, GOSE = Glasgow Outcome Scale-Extended, ICP = intracranial pressure, IMPACT = International Mission for Prognosis and Analysis of Clinical Trials, mmHg = millimeters of mercury, LLR = lower limit of reactivity, PAx = pulse amplitude index, PRx = pressure reactivity index, RAC = correlation (R) between slow-waves of AMP (A) and CPP (C), ULR = upper limit of reactivity.*

Appendix T. Multivariable Models of Cerebrovascular Reactivity Measures for Transition in Outcome from 1 to 3 Months with Patients Trichotomized by Age

| **Model** | **Age < 30** | | | | **Age 30 – 60** | | | | **Age > 60** | | | |
| --- | --- | --- | --- | --- | --- | --- | --- | --- | --- | --- | --- | --- |
|  | **AUC (95% CI)** | **AIC** | **p-value** | **Nagelkerke's R^2^** | **AUC (95% CI)** | **AIC** | **p-value** | **Nagelkerke's R^2^** | **AUC (95% CI)** | **AIC** | **p-value** | **Nagelkerke's R^2^** |
| Core | 0.643 (0.433 - 0.843) | 48.7 | 0.1012 | 0.065 | 0.639 (0.484 - 0.779) | 79.8 | **0.0465** | 0.078 | 0.954 (0.833 - 1) | 18.4 | **0.0086** | 0.612 |
| Core + CPPopt-PRx | 0.648 (0.429 - 0.843) | 50.7 | 0.0963 | 0.066 | 0.648 (0.497 - 0.792) | 81.6 | **0.0387** | 0.082 | 1 (1 - 1) | 12 | **0.0032** | 1 |
| Core + % Time ΔCPPopt-PRx > 5 mmHg | 0.676 (0.471 - 0.867) | 48.4 | 0.0616 | 0.161 | 0.638 (0.483 - 0.787) | 81.7 | **0.0472** | 0.082 | 0.963 (0.852 - 1) | 20.3 | **0.0046** | 0.616 |
| Core + % Time ΔCPPopt-PRx > 10 mmHg | 0.71 (0.51 - 0.895) | 48.7 | **0.0338** | 0.151 | 0.654 (0.506 - 0.793) | 80.5 | **0.0351** | 0.108 | 0.944 (0.833 - 1) | 20.4 | **0.0069** | 0.612 |
| Core + % Time ΔCPPopt-PRx < -5 mmHg | 0.652 (0.438 - 0.838) | 50 | 0.0911 | 0.094 | 0.649 (0.493 - 0.792) | 81.6 | **0.0375** | 0.083 | 1 (1 - 1) | 12 | **0.0032** | 1 |
| Core + % Time ΔCPPopt-PRx < -10 mmHg | 0.652 (0.438 - 0.852) | 49.6 | 0.0911 | 0.113 | 0.644 (0.494 - 0.787) | 81.4 | **0.0424** | 0.087 | 1 (1 - 1) | 12 | **0.0032** | 1 |
| Core + % Time CPP > ULR-PRx | 0.719 (0.505 - 0.886) | 48.6 | **0.0276** | 0.155 | 0.639 (0.491 - 0.78) | 81.8 | **0.0465** | 0.079 | 0.944 (0.833 - 1) | 20.3 | **0.0069** | 0.621 |
| Core + % Time CPP < LLR-PRx | 0.681 (0.462 - 0.862) | 48.9 | 0.0578 | 0.143 | 0.636 (0.48 - 0.774) | 81.7 | **0.0485** | 0.081 | 0.944 (0.833 - 1) | 19.9 | **0.0069** | 0.64 |
| Core + CPPopt-PAx | 0.629 (0.414 - 0.833) | 50.4 | 0.1259 | 0.077 | 0.651 (0.497 - 0.786) | 81.5 | **0.037** | 0.085 | 0.944 (0.833 - 1) | 20.2 | **0.0093** | 0.624 |
| Core + % Time ΔCPPopt-PAx > 5 mmHg | 0.795 (0.59 - 0.952) | 45.9 | **0.0048** | 0.259 | 0.649 (0.497 - 0.793) | 81.5 | **0.0375** | 0.085 | 0.963 (0.833 - 1) | 19.6 | **0.0046** | 0.657 |
| Core + % Time ΔCPPopt-PAx > 10 mmHg | 0.729 (0.529 - 0.905) | 48.7 | **0.0228** | 0.152 | 0.667 (0.519 - 0.816) | 80.8 | **0.0305** | 0.101 | 0.963 (0.852 - 1) | 20.3 | **0.0046** | 0.62 |
| Core + % Time ΔCPPopt-PAx < -5 mmHg | 0.671 (0.462 - 0.862) | 48.3 | 0.0666 | 0.165 | 0.631 (0.471 - 0.78) | 80.2 | 0.0543 | 0.117 | 0.944 (0.833 - 1) | 20.4 | **0.0069** | 0.612 |
| Core + % Time ΔCPPopt-PAx < -10 mmHg | 0.643 (0.429 - 0.838) | 49.2 | 0.1018 | 0.13 | 0.634 (0.47 - 0.787) | 81 | 0.0516 | 0.098 | 0.944 (0.833 - 1) | 20.4 | **0.0069** | 0.612 |
| Core + % Time CPP > ULR-PAx | 0.81 (0.638 - 0.938) | 42.1 | **0.0033** | 0.392 | 0.661 (0.511 - 0.8) | 79.7 | **0.0305** | 0.127 | 0.963 (0.852 - 1) | 20 | **0.0046** | 0.637 |
| Core + % Time CPP < LLR-PAx | 0.81 (0.633 - 0.952) | 42.4 | **0.0033** | 0.383 | 0.667 (0.516 - 0.805) | 79.6 | **0.0305** | 0.129 | 0.963 (0.852 - 1) | 20.4 | **0.0046** | 0.612 |
| Core + CPPopt-RAC | 0.648 (0.424 - 0.867) | 50.3 | 0.0963 | 0.085 | 0.651 (0.5 - 0.792) | 81.4 | **0.037** | 0.088 | 0.963 (0.833 - 1) | 20.4 | **0.0046** | 0.613 |
| Core + % Time ΔCPPopt-RAC > 5 mmHg | 0.686 (0.462 - 0.867) | 49.4 | 0.0533 | 0.123 | 0.645 (0.491 - 0.789) | 81.7 | **0.0414** | 0.08 | 0.981 (0.889 - 1) | 18.6 | **0.0032** | 0.71 |
| Core + % Time ΔCPPopt-RAC > 10 mmHg | 0.676 (0.467 - 0.862) | 49.4 | 0.0616 | 0.122 | 0.698 (0.549 - 0.828) | 79.9 | **0.0305** | 0.123 | 0.963 (0.852 - 1) | 19.4 | **0.0046** | 0.666 |
| Core + % Time ΔCPPopt-RAC < -5 mmHg | 0.686 (0.481 - 0.872) | 48.9 | 0.0533 | 0.141 | 0.621 (0.451 - 0.772) | 80.8 | 0.0691 | 0.101 | 0.944 (0.815 - 1) | 19.9 | **0.0069** | 0.64 |
| Core + % Time ΔCPPopt-RAC < -10 mmHg | 0.7 (0.486 - 0.881) | 48.4 | **0.0408** | 0.161 | 0.631 (0.47 - 0.772) | 81.5 | 0.0543 | 0.086 | 0.944 (0.815 - 1) | 19.8 | **0.0069** | 0.648 |
| Core + % Time CPP > ULR-RAC | 0.824 (0.671 - 0.948) | 39.1 | **0.0028** | 0.484 | 0.638 (0.486 - 0.779) | 81.6 | **0.0472** | 0.082 | 0.963 (0.833 - 1) | 19.8 | **0.0046** | 0.646 |
| Core + % Time CPP < LLR-RAC | 0.748 (0.571 - 0.91) | 45.2 | **0.0151** | 0.285 | 0.614 (0.457 - 0.762) | 81.4 | 0.081 | 0.088 | 0.963 (0.889 - 1) | 20.3 | **0.0046** | 0.616 |
| Core + CT | 0.738 (0.538 - 0.924) | 47.2 | **0.0196** | 0.21 | 0.675 (0.53 - 0.815) | 80.6 | **0.0305** | 0.106 | 0.954 (0.833 - 1) | 19.2 | **0.0086** | 0.679 |
| Core + CT + CPPopt-PRx | 0.786 (0.595 - 0.943) | 47.7 | **0.0064** | 0.268 | 0.69 (0.539 - 0.836) | 82.5 | **0.0305** | 0.108 | 1 (1 - 1) | 14 | **0.0032** | 1 |
| Core + CT + % Time ΔCPPopt-PRx > 5 mmHg | 0.743 (0.548 - 0.914) | 48.8 | **0.0167** | 0.224 | 0.678 (0.52 - 0.812) | 82.5 | **0.0305** | 0.11 | 0.963 (0.852 - 1) | 21.2 | **0.0077** | 0.68 |
| Core + CT + % Time ΔCPPopt-PRx > 10 mmHg | 0.752 (0.557 - 0.924) | 49.2 | **0.0139** | 0.211 | 0.69 (0.545 - 0.82) | 81.1 | **0.0305** | 0.142 | 0.944 (0.833 - 1) | 21.1 | **0.0093** | 0.683 |
| Core + CT + % Time ΔCPPopt-PRx < -5 mmHg | 0.748 (0.548 - 0.933) | 49.2 | **0.0151** | 0.211 | 0.688 (0.542 - 0.828) | 82.4 | **0.0305** | 0.11 | 1 (1 - 1) | 14 | **0.0032** | 1 |
| Core + CT + % Time ΔCPPopt-PRx < -10 mmHg | 0.719 (0.519 - 0.89) | 49 | **0.0276** | 0.216 | 0.691 (0.54 - 0.835) | 82.2 | **0.0305** | 0.115 | 1 (1 - 1) | 14 | **0.0032** | 1 |
| Core + CT + % Time CPP > ULR-PRx | 0.886 (0.752 - 0.981) | 37.4 | **0.0011** | 0.585 | 0.68 (0.526 - 0.816) | 82.6 | **0.0305** | 0.107 | 0.963 (0.889 - 1) | 21.2 | **0.0077** | 0.68 |
| Core + CT + % Time CPP < LLR-PRx | 0.729 (0.524 - 0.905) | 48.4 | **0.0228** | 0.24 | 0.678 (0.523 - 0.813) | 82.6 | **0.0305** | 0.107 | 0.944 (0.833 - 1) | 21.2 | **0.0093** | 0.68 |
| Core + CT + CPPopt-PAx | 0.757 (0.567 - 0.938) | 48.8 | **0.0126** | 0.226 | 0.688 (0.536 - 0.83) | 82.5 | **0.0305** | 0.109 | 0.944 (0.833 - 1) | 21.2 | **0.0093** | 0.681 |
| Core + CT + % Time ΔCPPopt-PAx > 5 mmHg | 0.814 (0.633 - 0.957) | 46.7 | **0.0033** | 0.304 | 0.678 (0.523 - 0.823) | 82.3 | **0.0305** | 0.114 | 0.963 (0.852 - 1) | 20.4 | **0.0077** | 0.718 |
| Core + CT + % Time ΔCPPopt-PAx > 10 mmHg | 0.757 (0.557 - 0.924) | 48.7 | **0.0126** | 0.228 | 0.688 (0.534 - 0.826) | 81.6 | **0.0305** | 0.13 | 0.963 (0.833 - 1) | 21.1 | **0.0077** | 0.684 |
| Core + CT + % Time ΔCPPopt-PAx < -5 mmHg | 0.79 (0.6 - 0.957) | 47.4 | **0.0055** | 0.278 | 0.693 (0.536 - 0.83) | 81.3 | **0.0305** | 0.136 | 0.963 (0.851 - 1) | 21 | **0.0077** | 0.688 |
| Core + CT + % Time ΔCPPopt-PAx < -10 mmHg | 0.757 (0.557 - 0.924) | 48.2 | **0.0126** | 0.248 | 0.684 (0.527 - 0.822) | 82 | **0.0305** | 0.121 | 0.963 (0.852 - 1) | 21 | **0.0077** | 0.687 |
| Core + CT + % Time CPP > ULR-PAx | 0.876 (0.714 - 1) | 39.6 | **0.0011** | 0.528 | 0.681 (0.532 - 0.815) | 80.7 | **0.0305** | 0.149 | 0.963 (0.889 - 1) | 21.1 | **0.0077** | 0.685 |
| Core + CT + % Time CPP < LLR-PAx | 0.81 (0.628 - 0.938) | 43.2 | **0.0033** | 0.421 | 0.701 (0.549 - 0.839) | 80.8 | **0.0305** | 0.147 | 0.963 (0.852 - 1) | 20.5 | **0.0077** | 0.716 |
| Core + CT + CPPopt-RAC | 0.757 (0.552 - 0.929) | 49.1 | **0.0126** | 0.216 | 0.684 (0.53 - 0.826) | 82.3 | **0.0305** | 0.113 | 0.963 (0.833 - 1) | 21.1 | **0.0077** | 0.683 |
| Core + CT + % Time ΔCPPopt-RAC > 5 mmHg | 0.762 (0.567 - 0.929) | 48.8 | **0.012** | 0.224 | 0.685 (0.532 - 0.825) | 82.6 | **0.0305** | 0.107 | 0.963 (0.852 - 1) | 19.8 | **0.0077** | 0.75 |
| Core + CT + % Time ΔCPPopt-RAC > 10 mmHg | 0.752 (0.562 - 0.919) | 49 | **0.0139** | 0.216 | 0.716 (0.572 - 0.848) | 80.6 | **0.0305** | 0.152 | 0.944 (0.815 - 1) | 20.6 | **0.0093** | 0.71 |
| Core + CT + % Time ΔCPPopt-RAC < -5 mmHg | 0.757 (0.567 - 0.919) | 48.3 | **0.0126** | 0.244 | 0.675 (0.533 - 0.818) | 81.7 | **0.0305** | 0.127 | 0.981 (0.889 - 1) | 20.1 | **0.0069** | 0.734 |
| Core + CT + % Time ΔCPPopt-RAC < -10 mmHg | 0.748 (0.552 - 0.914) | 48.4 | **0.0151** | 0.241 | 0.662 (0.517 - 0.806) | 82.2 | **0.0305** | 0.115 | 0.981 (0.889 - 1) | 19.7 | **0.0069** | 0.753 |
| Core + CT + % Time CPP > ULR-RAC | 0.867 (0.714 - 0.971) | 36.8 | **0.0014** | 0.602 | 0.668 (0.517 - 0.81) | 82.5 | **0.0305** | 0.108 | 0.963 (0.889 - 1) | 20.9 | **0.0077** | 0.693 |
| Core + CT + % Time CPP < LLR-RAC | 0.781 (0.595 - 0.929) | 45.8 | **0.0072** | 0.334 | 0.671 (0.526 - 0.813) | 82.4 | **0.0305** | 0.11 | 0.981 (0.889 - 1) | 20.2 | **0.0069** | 0.73 |
| Core + CT + ICP > 20 mmHg | 0.81 (0.633 - 0.948) | 44.4 | **0.0033** | 0.38 | 0.657 (0.501 - 0.803) | 81.9 | **0.0323** | 0.123 | 1 (1 - 1) | 14 | **0.0032** | 1 |
| Core + CT + ICP > 20 mmHg + CPPopt-PRx | 0.814 (0.643 - 0.952) | 46 | **0.0033** | 0.394 | 0.668 (0.513 - 0.809) | 83.9 | **0.0305** | 0.124 | 1 (1 - 1) | 16 | **0.0036** | 1 |
| Core + CT + ICP > 20 mmHg + % Time ΔCPPopt-PRx > 5 mmHg | 0.829 (0.652 - 0.962) | 46.4 | **0.0028** | 0.381 | 0.662 (0.507 - 0.809) | 83.8 | **0.0305** | 0.125 | 1 (1 - 1) | 16 | **0.0032** | 1 |
| Core + CT + ICP > 20 mmHg + % Time ΔCPPopt-PRx > 10 mmHg | 0.824 (0.648 - 0.967) | 46.3 | **0.0028** | 0.385 | 0.691 (0.539 - 0.826) | 82.5 | **0.0305** | 0.155 | 1 (1 - 1) | 16 | **0.0032** | 1 |
| Core + CT + ICP > 20 mmHg + % Time ΔCPPopt-PRx < -5 mmHg | 0.848 (0.7 - 0.971) | 44 | **0.0023** | 0.456 | 0.68 (0.532 - 0.818) | 83.5 | **0.0305** | 0.131 | 1 (1 - 1) | 16 | **0.0032** | 1 |
| Core + CT + ICP > 20 mmHg + % Time ΔCPPopt-PRx < -10 mmHg | 0.829 (0.671 - 0.962) | 44.6 | **0.0028** | 0.439 | 0.697 (0.555 - 0.829) | 82.9 | **0.0305** | 0.146 | 1 (1 - 1) | 16 | **0.0036** | 1 |
| Core + CT + ICP > 20 mmHg + % Time CPP > ULR-PRx | 0.938 (0.838 - 1) | 34.5 | **<0.0001** | 0.703 | 0.665 (0.513 - 0.813) | 83.7 | **0.0305** | 0.126 | 1 (1 - 1) | 16 | **0.0032** | 1 |
| Core + CT + ICP > 20 mmHg + % Time CPP < LLR-PRx | 0.824 (0.657 - 0.957) | 46.4 | **0.0028** | 0.381 | 0.659 (0.501 - 0.797) | 83.9 | **0.0305** | 0.124 | 1 (1 - 1) | 16 | **0.0032** | 1 |
| Core + CT + ICP > 20 mmHg + CPPopt-PAx | 0.819 (0.648 - 0.952) | 46.1 | **0.003** | 0.39 | 0.659 (0.51 - 0.792) | 83.9 | **0.0305** | 0.123 | 1 (1 - 1) | 16 | **0.0032** | 1 |
| Core + CT + ICP > 20 mmHg + % Time ΔCPPopt-PAx > 5 mmHg | 0.819 (0.657 - 0.943) | 44.6 | **0.003** | 0.438 | 0.671 (0.513 - 0.812) | 83.5 | **0.0305** | 0.131 | 1 (1 - 1) | 16 | **0.0032** | 1 |
| Core + CT + ICP > 20 mmHg + % Time ΔCPPopt-PAx > 10 mmHg | 0.814 (0.633 - 0.957) | 46.3 | **0.0033** | 0.385 | 0.685 (0.534 - 0.822) | 82.8 | **0.0305** | 0.148 | 1 (1 - 1) | 16 | **0.0032** | 1 |
| Core + CT + ICP > 20 mmHg + % Time ΔCPPopt-PAx < -5 mmHg | 0.819 (0.652 - 0.952) | 46 | **0.003** | 0.394 | 0.69 (0.555 - 0.825) | 82.7 | **0.0305** | 0.151 | 1 (1 - 1) | 16 | **0.0032** | 1 |
| Core + CT + ICP > 20 mmHg + % Time ΔCPPopt-PAx < -10 mmHg | 0.805 (0.628 - 0.943) | 46.4 | **0.0037** | 0.38 | 0.674 (0.529 - 0.809) | 83.2 | **0.0305** | 0.138 | 1 (1 - 1) | 16 | **0.0032** | 1 |
| Core + CT + ICP > 20 mmHg + % Time CPP > ULR-PAx | 0.871 (0.714 - 0.981) | 41 | **0.0014** | 0.543 | 0.674 (0.52 - 0.819) | 82.4 | **0.0305** | 0.156 | 1 (1 - 1) | 16 | **0.0032** | 1 |
| Core + CT + ICP > 20 mmHg + % Time CPP < LLR-PAx | 0.824 (0.657 - 0.952) | 44.5 | **0.0028** | 0.443 | 0.701 (0.56 - 0.845) | 82.6 | **0.0305** | 0.152 | 0.5 (0.5 - 0.5) | 232.3 | 1 | -23348.4 |
| Core + CT + ICP > 20 mmHg + CPPopt-RAC | 0.81 (0.629 - 0.948) | 46.4 | **0.0033** | 0.383 | 0.659 (0.497 - 0.8) | 83.8 | **0.0305** | 0.125 | 1 (1 - 1) | 16 | **0.0032** | 1 |
| Core + CT + ICP > 20 mmHg + % Time ΔCPPopt-RAC > 5 mmHg | 0.814 (0.648 - 0.957) | 46.4 | **0.0033** | 0.382 | 0.67 (0.523 - 0.806) | 83.8 | **0.0305** | 0.124 | 1 (1 - 1) | 16 | **0.0032** | 1 |
| Core + CT + ICP > 20 mmHg + % Time ΔCPPopt-RAC > 10 mmHg | 0.81 (0.643 - 0.948) | 46.3 | **0.0033** | 0.383 | 0.713 (0.568 - 0.842) | 81.9 | **0.0305** | 0.167 | 1 (1 - 1) | 16 | **0.0032** | 1 |
| Core + CT + ICP > 20 mmHg + % Time ΔCPPopt-RAC < -5 mmHg | 0.81 (0.629 - 0.957) | 46.4 | **0.0033** | 0.38 | 0.67 (0.529 - 0.797) | 83.1 | **0.0305** | 0.14 | 1 (1 - 1) | 16 | **0.0032** | 1 |
| Core + CT + ICP > 20 mmHg + % Time ΔCPPopt-RAC < -10 mmHg | 0.81 (0.633 - 0.948) | 46.2 | **0.0033** | 0.387 | 0.659 (0.504 - 0.802) | 83.5 | **0.0305** | 0.131 | 1 (1 - 1) | 16 | **0.0032** | 1 |
| Core + CT + ICP > 20 mmHg + % Time CPP > ULR-RAC | 0.867 (0.719 - 0.971) | 38.7 | **0.0014** | 0.604 | 0.662 (0.507 - 0.8) | 83.9 | **0.0305** | 0.123 | 1 (1 - 1) | 16 | **0.0036** | 1 |
| Core + CT + ICP > 20 mmHg + % Time CPP < LLR-RAC | 0.805 (0.624 - 0.943) | 46.3 | **0.0037** | 0.385 | 0.661 (0.513 - 0.802) | 83.9 | **0.0305** | 0.123 | 1 (1 - 1) | 16 | **0.0032** | 1 |
| Core + CT + ICP > 22 mmHg | 0.838 (0.657 - 0.957) | 41.6 | **0.0023** | 0.47 | 0.663 (0.511 - 0.807) | 81.7 | **0.0305** | 0.127 | 1 (1 - 1) | 14 | **0.0032** | 1 |
| Core + CT + ICP > 22 mmHg + CPPopt-PRx | 0.848 (0.7 - 0.967) | 43.1 | **0.0023** | 0.483 | 0.67 (0.514 - 0.809) | 83.7 | **0.0305** | 0.128 | 1 (1 - 1) | 16 | **0.0036** | 1 |
| Core + CT + ICP > 22 mmHg + % Time ΔCPPopt-PRx > 5 mmHg | 0.852 (0.7 - 0.967) | 43.5 | **0.0021** | 0.471 | 0.664 (0.517 - 0.808) | 83.6 | **0.0305** | 0.13 | 1 (1 - 1) | 16 | **0.0032** | 1 |
| Core + CT + ICP > 22 mmHg + % Time ΔCPPopt-PRx > 10 mmHg | 0.852 (0.695 - 0.967) | 43.5 | **0.0021** | 0.473 | 0.695 (0.547 - 0.828) | 82.3 | **0.0305** | 0.16 | 1 (1 - 1) | 16 | **0.0032** | 1 |
| Core + CT + ICP > 22 mmHg + % Time ΔCPPopt-PRx < -5 mmHg | 0.89 (0.748 - 0.991) | 39.7 | **0.0011** | 0.578 | 0.682 (0.526 - 0.812) | 83.3 | **0.0305** | 0.135 | 1 (1 - 1) | 16 | **0.0032** | 1 |
| Core + CT + ICP > 22 mmHg + % Time ΔCPPopt-PRx < -10 mmHg | 0.876 (0.733 - 0.971) | 40.8 | **0.0011** | 0.548 | 0.705 (0.55 - 0.838) | 82.6 | **0.0305** | 0.151 | 1 (1 - 1) | 16 | **0.0036** | 1 |
| Core + CT + ICP > 22 mmHg + % Time CPP > ULR-PRx | 0.948 (0.857 - 1) | 33.2 | **<0.0001** | 0.729 | 0.667 (0.516 - 0.8) | 83.5 | **0.0305** | 0.131 | 1 (1 - 1) | 16 | **0.0032** | 1 |
| Core + CT + ICP > 22 mmHg + % Time CPP < LLR-PRx | 0.843 (0.676 - 0.962) | 43.5 | **0.0023** | 0.472 | 0.662 (0.489 - 0.802) | 83.7 | **0.0305** | 0.128 | 1 (1 - 1) | 16 | **0.0032** | 1 |
| Core + CT + ICP > 22 mmHg + CPPopt-PAx | 0.838 (0.686 - 0.957) | 43 | **0.0023** | 0.486 | 0.667 (0.517 - 0.81) | 83.7 | **0.0305** | 0.128 | 1 (1 - 1) | 16 | **0.0032** | 1 |
| Core + CT + ICP > 22 mmHg + % Time ΔCPPopt-PAx > 5 mmHg | 0.862 (0.705 - 0.976) | 41.6 | **0.0019** | 0.526 | 0.677 (0.527 - 0.812) | 83.3 | **0.0305** | 0.136 | 1 (1 - 1) | 16 | **0.0032** | 1 |
| Core + CT + ICP > 22 mmHg + % Time ΔCPPopt-PAx > 10 mmHg | 0.833 (0.667 - 0.962) | 43.2 | **0.0025** | 0.48 | 0.687 (0.543 - 0.822) | 82.6 | **0.0305** | 0.152 | 1 (1 - 1) | 16 | **0.0032** | 1 |
| Core + CT + ICP > 22 mmHg + % Time ΔCPPopt-PAx < -5 mmHg | 0.871 (0.719 - 0.981) | 43 | **0.0014** | 0.487 | 0.691 (0.552 - 0.826) | 82.6 | **0.0305** | 0.153 | 1 (1 - 1) | 16 | **0.0032** | 1 |
| Core + CT + ICP > 22 mmHg + % Time ΔCPPopt-PAx < -10 mmHg | 0.838 (0.671 - 0.962) | 43.6 | **0.0023** | 0.47 | 0.68 (0.532 - 0.828) | 83.1 | **0.0305** | 0.142 | 1 (1 - 1) | 16 | **0.0032** | 1 |
| Core + CT + ICP > 22 mmHg + % Time CPP > ULR-PAx | 0.881 (0.743 - 0.976) | 39.2 | **0.0011** | 0.591 | 0.674 (0.516 - 0.815) | 82.3 | **0.0305** | 0.159 | 1 (1 - 1) | 16 | **0.0032** | 1 |
| Core + CT + ICP > 22 mmHg + % Time CPP < LLR-PAx | 0.843 (0.671 - 0.957) | 42 | **0.0023** | 0.515 | 0.695 (0.536 - 0.828) | 82.5 | **0.0305** | 0.155 | 0.5 (0.5 - 0.5) | 232.3 | 1 | -23348.4 |
| Core + CT + ICP > 22 mmHg + CPPopt-RAC | 0.852 (0.705 - 0.971) | 43.3 | **0.0021** | 0.478 | 0.662 (0.509 - 0.806) | 83.6 | **0.0305** | 0.129 | 1 (1 - 1) | 16 | **0.0032** | 1 |
| Core + CT + ICP > 22 mmHg + % Time ΔCPPopt-RAC > 5 mmHg | 0.838 (0.676 - 0.962) | 43.6 | **0.0023** | 0.47 | 0.667 (0.513 - 0.81) | 83.6 | **0.0305** | 0.129 | 1 (1 - 1) | 16 | **0.0032** | 1 |
| Core + CT + ICP > 22 mmHg + % Time ΔCPPopt-RAC > 10 mmHg | 0.829 (0.667 - 0.962) | 43.4 | **0.0028** | 0.474 | 0.713 (0.559 - 0.843) | 81.7 | **0.0305** | 0.171 | 1 (1 - 1) | 16 | **0.0032** | 1 |
| Core + CT + ICP > 22 mmHg + % Time ΔCPPopt-RAC < -5 mmHg | 0.838 (0.676 - 0.962) | 43.6 | **0.0023** | 0.47 | 0.672 (0.517 - 0.806) | 83 | **0.0305** | 0.143 | 1 (1 - 1) | 16 | **0.0032** | 1 |
| Core + CT + ICP > 22 mmHg + % Time ΔCPPopt-RAC < -10 mmHg | 0.871 (0.733 - 0.976) | 43.2 | **0.0014** | 0.482 | 0.664 (0.514 - 0.803) | 83.4 | **0.0305** | 0.134 | 1 (1 - 1) | 16 | **0.0032** | 1 |
| Core + CT + ICP > 22 mmHg + % Time CPP > ULR-RAC | 0.914 (0.781 - 0.99) | 37.4 | **<0.0001** | 0.635 | 0.665 (0.513 - 0.808) | 83.7 | **0.0305** | 0.127 | 1 (1 - 1) | 16 | **0.0032** | 1 |
| Core + CT + ICP > 22 mmHg + % Time CPP < LLR-RAC | 0.843 (0.686 - 0.967) | 43.6 | **0.0023** | 0.47 | 0.661 (0.503 - 0.796) | 83.7 | **0.0305** | 0.128 | 1 (1 - 1) | 16 | **0.0032** | 1 |

*Core model consisted of age, admission Glasgow Coma Scale - motor score, and admission pupillary response. CT variables consisted of admission Marshall CT grade, presence of traumatic subarachnoid hemorrhage, and presence of epidural hematoma. All p-values have been adjusted using the False Discovery Rate (FDR) method. Bolded p-values are those reaching statistical significance, p < 0.05.*

*AIC = Akaike information criterion, AMP = pulse amplitude of ICP, AUC = area under the curve, CI = confidence interval, CPP = cerebral perfusion pressure, CPPopt = cerebral perfusion pressure optimum, ΔCPPopt = CPP - CPPopt, CT = computed tomography, GOSE = Glasgow Outcome Scale-Extended, ICP = intracranial pressure, IMPACT = International Mission for Prognosis and Analysis of Clinical Trials, mmHg = millimeters of mercury, LLR = lower limit of reactivity, PAx = pulse amplitude index, PRx = pressure reactivity index, RAC = correlation (R) between slow-waves of AMP (A) and CPP (C), ULR = upper limit of reactivity.*

Appendix U. Multivariable Models of Cerebrovascular Reactivity Measures for Transition in Outcome from 3 to 6 Months with Patients Trichotomized by Age

| **Model** | **Age < 30** | | | | **Age 30 – 60** | | | | **Age > 60** | | | |
| --- | --- | --- | --- | --- | --- | --- | --- | --- | --- | --- | --- | --- |
|  | **AUC (95% CI)** | **AIC** | **p-value** | **Nagelkerke's R^2^** | **AUC (95% CI)** | **AIC** | **p-value** | **Nagelkerke's R^2^** | **AUC (95% CI)** | **AIC** | **p-value** | **Nagelkerke's R^2^** |
| Core | 0.636 (0.404 - 0.854) | 47.6 | 0.1262 | 0.04 | 0.63 (0.472 - 0.794) | 73.7 | 0.0701 | 0.073 | 0.789 (0.579 - 0.947) | 19.5 | 0.1053 | 0.345 |
| Core + CPPopt-PRx | 0.581 (0.338 - 0.798) | 49.3 | 0.2462 | 0.056 | 0.642 (0.485 - 0.788) | 74.2 | 0.0577 | 0.109 | 0.868 (0.684 - 1) | 20.8 | 0.0636 | 0.405 |
| Core + % Time ΔCPPopt-PRx > 5 mmHg | 0.793 (0.606 - 0.939) | 43.9 | **0.0293** | 0.276 | 0.632 (0.467 - 0.792) | 75.4 | 0.0676 | 0.081 | 1 (1 - 1) | 12 | **0.0164** | 1 |
| Core + % Time ΔCPPopt-PRx > 10 mmHg | 0.737 (0.545 - 0.894) | 44.7 | 0.0513 | 0.248 | 0.627 (0.459 - 0.778) | 75.3 | 0.0729 | 0.081 | 1 (1 - 1) | 12 | **0.0164** | 1 |
| Core + % Time ΔCPPopt-PRx < -5 mmHg | 0.783 (0.601 - 0.934) | 42.8 | **0.0339** | 0.319 | 0.619 (0.464 - 0.775) | 75.4 | 0.0854 | 0.079 | 0.921 (0.789 - 1) | 19.6 | 0.0525 | 0.501 |
| Core + % Time ΔCPPopt-PRx < -10 mmHg | 0.707 (0.505 - 0.879) | 45.5 | 0.0682 | 0.216 | 0.618 (0.446 - 0.776) | 75.5 | 0.0874 | 0.077 | 0.974 (0.895 - 1) | 17.6 | **0.033** | 0.651 |
| Core + % Time CPP > ULR-PRx | 0.596 (0.359 - 0.808) | 49 | 0.2084 | 0.068 | 0.719 (0.551 - 0.863) | 70 | **0.0275** | 0.208 | 0.974 (0.895 - 1) | 17.7 | **0.0199** | 0.645 |
| Core + % Time CPP < LLR-PRx | 0.697 (0.495 - 0.879) | 45.5 | 0.0736 | 0.215 | 0.686 (0.505 - 0.835) | 71.9 | **0.0407** | 0.163 | 0.816 (0.579 - 1) | 21.5 | 0.0974 | 0.346 |
| Core + CPPopt-PAx | 0.646 (0.429 - 0.839) | 48.3 | 0.118 | 0.101 | 0.636 (0.477 - 0.784) | 75.5 | 0.0658 | 0.077 | 0.816 (0.579 - 1) | 21.3 | 0.0974 | 0.36 |
| Core + % Time ΔCPPopt-PAx > 5 mmHg | 0.641 (0.409 - 0.839) | 46.2 | 0.1259 | 0.189 | 0.694 (0.539 - 0.833) | 73.9 | **0.0367** | 0.118 | 1 (1 - 1) | 12 | **0.0164** | 1 |
| Core + % Time ΔCPPopt-PAx > 10 mmHg | 0.692 (0.46 - 0.874) | 45.4 | 0.0765 | 0.218 | 0.673 (0.515 - 0.832) | 74.6 | **0.0426** | 0.1 | 1 (1 - 1) | 12 | **0.0166** | 1 |
| Core + % Time ΔCPPopt-PAx < -5 mmHg | 0.702 (0.485 - 0.879) | 47.5 | 0.0723 | 0.136 | 0.693 (0.547 - 0.828) | 72.2 | **0.0367** | 0.157 | 0.816 (0.632 - 0.974) | 21.4 | 0.0974 | 0.357 |
| Core + % Time ΔCPPopt-PAx < -10 mmHg | 0.732 (0.52 - 0.914) | 47.6 | 0.0559 | 0.13 | 0.662 (0.511 - 0.797) | 74.3 | **0.0477** | 0.107 | 0.842 (0.684 - 1) | 21 | 0.0818 | 0.391 |
| Core + % Time CPP > ULR-PAx | 0.631 (0.419 - 0.838) | 49.4 | 0.1343 | 0.049 | 0.727 (0.572 - 0.856) | 67.9 | **0.0233** | 0.253 | 1 (1 - 1) | 12 | **0.0164** | 1 |
| Core + % Time CPP < LLR-PAx | 0.788 (0.601 - 0.939) | 43.9 | **0.0318** | 0.275 | 0.623 (0.454 - 0.781) | 74.9 | 0.0804 | 0.093 | 0.868 (0.684 - 1) | 20.7 | 0.0636 | 0.414 |
| Core + CPPopt-RAC | 0.621 (0.384 - 0.828) | 48.8 | 0.1515 | 0.077 | 0.629 (0.464 - 0.776) | 75.4 | 0.0711 | 0.08 | 0.842 (0.658 - 1) | 21.3 | 0.0818 | 0.365 |
| Core + % Time ΔCPPopt-RAC > 5 mmHg | 0.672 (0.449 - 0.879) | 44.6 | 0.1051 | 0.249 | 0.66 (0.507 - 0.802) | 75.2 | **0.0477** | 0.084 | 1 (1 - 1) | 12 | **0.0164** | 1 |
| Core + % Time ΔCPPopt-RAC > 10 mmHg | 0.692 (0.47 - 0.884) | 45 | 0.0765 | 0.237 | 0.632 (0.469 - 0.781) | 75.7 | 0.0676 | 0.073 | 1 (1 - 1) | 12 | **0.0164** | 1 |
| Core + % Time ΔCPPopt-RAC < -5 mmHg | 0.742 (0.535 - 0.919) | 46.4 | 0.0513 | 0.18 | 0.649 (0.5 - 0.788) | 75.1 | 0.0524 | 0.086 | 0.816 (0.605 - 1) | 21.5 | 0.0974 | 0.348 |
| Core + % Time ΔCPPopt-RAC < -10 mmHg | 0.662 (0.455 - 0.848) | 48.3 | 0.1121 | 0.101 | 0.634 (0.464 - 0.781) | 75.7 | 0.0667 | 0.073 | 0.789 (0.579 - 0.947) | 21.5 | 0.119 | 0.348 |
| Core + % Time CPP > ULR-RAC | 0.636 (0.419 - 0.839) | 49.4 | 0.1262 | 0.048 | 0.711 (0.56 - 0.85) | 70.6 | **0.0306** | 0.194 | 0.974 (0.895 - 1) | 16.1 | **0.033** | 0.755 |
| Core + % Time CPP < LLR-RAC | 0.793 (0.611 - 0.949) | 45.8 | **0.0293** | 0.203 | 0.737 (0.582 - 0.869) | 69.9 | **0.02** | 0.21 | 0.842 (0.632 - 1) | 21.5 | 0.0818 | 0.349 |
| Core + CT | 0.636 (0.414 - 0.833) | 47.6 | 0.1262 | 0.13 | 0.638 (0.477 - 0.782) | 75 | 0.0625 | 0.088 | 0.895 (0.684 - 1) | 19.9 | 0.0547 | 0.48 |
| Core + CT + CPPopt-PRx | 0.636 (0.419 - 0.838) | 49.6 | 0.1262 | 0.13 | 0.691 (0.544 - 0.824) | 75.3 | **0.0373** | 0.131 | 0.868 (0.579 - 1) | 21.3 | 0.0603 | 0.525 |
| Core + CT + % Time ΔCPPopt-PRx > 5 mmHg | 0.798 (0.616 - 0.939) | 45.7 | **0.0293** | 0.283 | 0.655 (0.5 - 0.799) | 76.7 | **0.0477** | 0.098 | 1 (1 - 1) | 14 | **0.0164** | 1 |
| Core + CT + % Time ΔCPPopt-PRx > 10 mmHg | 0.737 (0.535 - 0.904) | 46.7 | 0.0513 | 0.248 | 0.655 (0.5 - 0.802) | 76.6 | **0.0477** | 0.1 | 1 (1 - 1) | 14 | **0.0164** | 1 |
| Core + CT + % Time ΔCPPopt-PRx < -5 mmHg | 0.828 (0.657 - 0.96) | 43.8 | **0.0293** | 0.353 | 0.645 (0.492 - 0.789) | 76.8 | 0.0553 | 0.094 | 1 (1 - 1) | 14 | **0.0164** | 1 |
| Core + CT + % Time ΔCPPopt-PRx < -10 mmHg | 0.768 (0.566 - 0.939) | 46.2 | **0.0401** | 0.267 | 0.644 (0.485 - 0.789) | 76.9 | 0.0562 | 0.092 | 0.974 (0.895 - 1) | 18.7 | **0.033** | 0.71 |
| Core + CT + % Time CPP > ULR-PRx | 0.636 (0.409 - 0.843) | 49.6 | 0.1262 | 0.13 | 0.75 (0.598 - 0.873) | 71 | **0.0189** | 0.231 | 0.974 (0.895 - 1) | 19.7 | **0.0199** | 0.646 |
| Core + CT + % Time CPP < LLR-PRx | 0.742 (0.551 - 0.909) | 46 | 0.0513 | 0.273 | 0.703 (0.542 - 0.851) | 73.4 | **0.0352** | 0.176 | 0.947 (0.789 - 1) | 20.6 | **0.0387** | 0.581 |
| Core + CT + CPPopt-PAx | 0.621 (0.394 - 0.823) | 49.4 | 0.1515 | 0.139 | 0.657 (0.508 - 0.793) | 76.8 | **0.0477** | 0.095 | 0.868 (0.632 - 1) | 21.5 | 0.0603 | 0.509 |
| Core + CT + % Time ΔCPPopt-PAx > 5 mmHg | 0.702 (0.495 - 0.879) | 47.3 | 0.0723 | 0.222 | 0.698 (0.546 - 0.832) | 75.4 | **0.0367** | 0.129 | 1 (1 - 1) | 14 | **0.0164** | 1 |
| Core + CT + % Time ΔCPPopt-PAx > 10 mmHg | 0.697 (0.475 - 0.874) | 47 | 0.0736 | 0.236 | 0.685 (0.531 - 0.824) | 76 | **0.041** | 0.113 | 1 (1 - 1) | 14 | **0.0164** | 1 |
| Core + CT + % Time ΔCPPopt-PAx < -5 mmHg | 0.712 (0.505 - 0.889) | 47.6 | 0.0628 | 0.212 | 0.694 (0.547 - 0.824) | 73.7 | **0.0367** | 0.167 | 0.895 (0.684 - 1) | 21.4 | 0.0547 | 0.517 |
| Core + CT + % Time ΔCPPopt-PAx < -10 mmHg | 0.712 (0.505 - 0.889) | 47.7 | 0.0628 | 0.208 | 0.676 (0.529 - 0.817) | 75.7 | **0.0415** | 0.12 | 0.921 (0.737 - 1) | 20.7 | 0.0525 | 0.574 |
| Core + CT + % Time CPP > ULR-PAx | 0.636 (0.409 - 0.838) | 49.4 | 0.1262 | 0.139 | 0.739 (0.595 - 0.856) | 69.3 | **0.02** | 0.268 | 1 (1 - 1) | 14 | **0.0164** | 1 |
| Core + CT + % Time CPP < LLR-PAx | 0.773 (0.586 - 0.924) | 45.2 | **0.0401** | 0.303 | 0.634 (0.469 - 0.788) | 76.4 | 0.0667 | 0.104 | 0.895 (0.684 - 1) | 21.8 | 0.0547 | 0.487 |
| Core + CT + CPPopt-RAC | 0.646 (0.429 - 0.848) | 49.5 | 0.118 | 0.132 | 0.663 (0.506 - 0.804) | 76.6 | **0.0477** | 0.099 | 0.868 (0.579 - 1) | 21.7 | 0.0603 | 0.495 |
| Core + CT + % Time ΔCPPopt-RAC > 5 mmHg | 0.727 (0.5 - 0.899) | 45.6 | 0.0604 | 0.288 | 0.67 (0.52 - 0.807) | 76.6 | **0.0447** | 0.1 | 1 (1 - 1) | 14 | **0.0164** | 1 |
| Core + CT + % Time ΔCPPopt-RAC > 10 mmHg | 0.737 (0.54 - 0.904) | 46.4 | 0.0513 | 0.258 | 0.645 (0.495 - 0.789) | 77 | 0.0553 | 0.089 | 1 (1 - 1) | 14 | **0.0164** | 1 |
| Core + CT + % Time ΔCPPopt-RAC < -5 mmHg | 0.722 (0.525 - 0.884) | 46.9 | 0.0604 | 0.238 | 0.658 (0.513 - 0.801) | 76.5 | **0.0477** | 0.103 | 0.895 (0.684 - 1) | 21.9 | 0.0547 | 0.481 |
| Core + CT + % Time ΔCPPopt-RAC < -10 mmHg | 0.657 (0.439 - 0.833) | 49 | 0.1121 | 0.155 | 0.655 (0.508 - 0.796) | 77 | **0.0477** | 0.089 | 0.895 (0.684 - 1) | 21.9 | 0.0547 | 0.482 |
| Core + CT + % Time CPP > ULR-RAC | 0.646 (0.399 - 0.843) | 49.4 | 0.118 | 0.136 | 0.711 (0.559 - 0.84) | 72.1 | **0.0306** | 0.206 | 1 (1 - 1) | 14 | **0.0164** | 1 |
| Core + CT + % Time CPP < LLR-RAC | 0.763 (0.581 - 0.919) | 46.9 | **0.0435** | 0.238 | 0.729 (0.575 - 0.868) | 71.8 | **0.0233** | 0.213 | 0.895 (0.684 - 1) | 21.7 | 0.0547 | 0.496 |
| Core + CT + ICP > 20 mmHg | 0.662 (0.444 - 0.854) | 49.2 | 0.1121 | 0.147 | 0.654 (0.508 - 0.792) | 76.4 | **0.0481** | 0.104 | 0.895 (0.684 - 1) | 21.9 | 0.0547 | 0.482 |
| Core + CT + ICP > 20 mmHg + CPPopt-PRx | 0.657 (0.439 - 0.848) | 51.2 | 0.1121 | 0.148 | 0.676 (0.523 - 0.814) | 77 | **0.0415** | 0.139 | 0.868 (0.579 - 1) | 23.3 | 0.0603 | 0.525 |
| Core + CT + ICP > 20 mmHg + % Time ΔCPPopt-PRx > 5 mmHg | 0.798 (0.621 - 0.934) | 47.7 | **0.0293** | 0.283 | 0.67 (0.515 - 0.81) | 77.8 | **0.0447** | 0.119 | 1 (1 - 1) | 16 | **0.0164** | 1 |
| Core + CT + ICP > 20 mmHg + % Time ΔCPPopt-PRx > 10 mmHg | 0.737 (0.54 - 0.899) | 48.6 | 0.0513 | 0.25 | 0.662 (0.503 - 0.809) | 77.8 | **0.0477** | 0.12 | 1 (1 - 1) | 16 | **0.0166** | 1 |
| Core + CT + ICP > 20 mmHg + % Time ΔCPPopt-PRx < -5 mmHg | 0.838 (0.662 - 0.965) | 45.6 | **0.0293** | 0.36 | 0.658 (0.503 - 0.802) | 77.9 | **0.0477** | 0.116 | 0.75 (0.5 - 1) | 88.1 | **0.0164** | -33.214 |
| Core + CT + ICP > 20 mmHg + % Time ΔCPPopt-PRx < -10 mmHg | 0.768 (0.586 - 0.924) | 48 | **0.0401** | 0.272 | 0.655 (0.5 - 0.797) | 77.8 | **0.0477** | 0.118 | 0.974 (0.895 - 1) | 20.7 | **0.033** | 0.711 |
| Core + CT + ICP > 20 mmHg + % Time CPP > ULR-PRx | 0.652 (0.439 - 0.838) | 51.1 | 0.1165 | 0.15 | 0.742 (0.593 - 0.868) | 72.9 | **0.02** | 0.233 | 0.974 (0.895 - 1) | 21.1 | **0.033** | 0.685 |
| Core + CT + ICP > 20 mmHg + % Time CPP < LLR-PRx | 0.742 (0.556 - 0.909) | 48 | 0.0513 | 0.273 | 0.722 (0.573 - 0.864) | 74 | **0.0258** | 0.207 | 1 (1 - 1) | 16 | **0.0164** | 1 |
| Core + CT + ICP > 20 mmHg + CPPopt-PAx | 0.657 (0.449 - 0.838) | 50.9 | 0.1121 | 0.159 | 0.654 (0.5 - 0.796) | 78.3 | **0.0483** | 0.106 | 1 (1 - 1) | 16 | **0.0164** | 1 |
| Core + CT + ICP > 20 mmHg + % Time ΔCPPopt-PAx > 5 mmHg | 0.687 (0.475 - 0.869) | 49.2 | 0.0828 | 0.227 | 0.701 (0.551 - 0.843) | 76.8 | **0.0356** | 0.142 | 1 (1 - 1) | 16 | **0.0164** | 1 |
| Core + CT + ICP > 20 mmHg + % Time ΔCPPopt-PAx > 10 mmHg | 0.697 (0.49 - 0.879) | 48.9 | 0.0736 | 0.24 | 0.693 (0.547 - 0.83) | 77.4 | **0.0367** | 0.128 | 1 (1 - 1) | 16 | **0.0164** | 1 |
| Core + CT + ICP > 20 mmHg + % Time ΔCPPopt-PAx < -5 mmHg | 0.712 (0.51 - 0.884) | 49.6 | 0.0628 | 0.212 | 0.703 (0.552 - 0.832) | 74.8 | **0.0352** | 0.189 | 1 (1 - 1) | 16 | **0.0164** | 1 |
| Core + CT + ICP > 20 mmHg + % Time ΔCPPopt-PAx < -10 mmHg | 0.712 (0.51 - 0.889) | 49.7 | 0.0628 | 0.208 | 0.683 (0.539 - 0.817) | 77 | **0.041** | 0.137 | 1 (1 - 1) | 16 | **0.0164** | 1 |
| Core + CT + ICP > 20 mmHg + % Time CPP > ULR-PAx | 0.652 (0.434 - 0.843) | 51.2 | 0.1165 | 0.147 | 0.775 (0.639 - 0.887) | 69.4 | **0.0103** | 0.307 | 1 (1 - 1) | 16 | **0.0166** | 1 |
| Core + CT + ICP > 20 mmHg + % Time CPP < LLR-PAx | 0.803 (0.611 - 0.949) | 45.9 | **0.0293** | 0.35 | 0.686 (0.539 - 0.82) | 77.2 | **0.0407** | 0.133 | 0.895 (0.632 - 1) | 23.7 | 0.0547 | 0.491 |
| Core + CT + ICP > 20 mmHg + CPPopt-RAC | 0.662 (0.449 - 0.848) | 51.1 | 0.1121 | 0.15 | 0.649 (0.5 - 0.788) | 78.2 | 0.0524 | 0.108 | 0.868 (0.632 - 1) | 23.6 | 0.0603 | 0.503 |
| Core + CT + ICP > 20 mmHg + % Time ΔCPPopt-RAC > 5 mmHg | 0.717 (0.49 - 0.909) | 47.6 | 0.0623 | 0.289 | 0.68 (0.536 - 0.81) | 78 | **0.0415** | 0.114 | 1 (1 - 1) | 16 | **0.0164** | 1 |
| Core + CT + ICP > 20 mmHg + % Time ΔCPPopt-RAC > 10 mmHg | 0.747 (0.545 - 0.909) | 48.3 | 0.0513 | 0.263 | 0.663 (0.518 - 0.796) | 78.4 | **0.0477** | 0.104 | 1 (1 - 1) | 16 | **0.0166** | 1 |
| Core + CT + ICP > 20 mmHg + % Time ΔCPPopt-RAC < -5 mmHg | 0.722 (0.53 - 0.889) | 48.9 | 0.0604 | 0.238 | 0.667 (0.518 - 0.809) | 77.6 | **0.0477** | 0.124 | 0.895 (0.684 - 1) | 23.8 | 0.0547 | 0.485 |
| Core + CT + ICP > 20 mmHg + % Time ΔCPPopt-RAC < -10 mmHg | 0.667 (0.465 - 0.843) | 51 | 0.1109 | 0.157 | 0.65 (0.502 - 0.786) | 78.3 | 0.0518 | 0.106 | 0.895 (0.684 - 1) | 23.8 | 0.0547 | 0.486 |
| Core + CT + ICP > 20 mmHg + % Time CPP > ULR-RAC | 0.646 (0.429 - 0.833) | 51.2 | 0.118 | 0.148 | 0.771 (0.642 - 0.882) | 71.6 | **0.0103** | 0.261 | 1 (1 - 1) | 16 | **0.0187** | 1 |
| Core + CT + ICP > 20 mmHg + % Time CPP < LLR-RAC | 0.803 (0.606 - 0.949) | 48.1 | **0.0293** | 0.269 | 0.768 (0.614 - 0.899) | 69.2 | **0.0103** | 0.311 | 0.921 (0.737 - 1) | 23.2 | 0.0506 | 0.53 |
| Core + CT + ICP > 22 mmHg | 0.657 (0.439 - 0.854) | 49.2 | 0.1121 | 0.146 | 0.658 (0.505 - 0.797) | 76.1 | **0.0477** | 0.111 | 0.895 (0.684 - 1) | 21.9 | 0.0547 | 0.481 |
| Core + CT + ICP > 22 mmHg + CPPopt-PRx | 0.652 (0.424 - 0.843) | 51.2 | 0.1165 | 0.146 | 0.676 (0.539 - 0.814) | 76.7 | **0.0415** | 0.145 | 0.868 (0.632 - 1) | 23.3 | 0.0603 | 0.526 |
| Core + CT + ICP > 22 mmHg + % Time ΔCPPopt-PRx > 5 mmHg | 0.798 (0.611 - 0.939) | 47.7 | **0.0293** | 0.283 | 0.676 (0.523 - 0.814) | 77.5 | **0.0415** | 0.127 | 1 (1 - 1) | 16 | **0.0164** | 1 |
| Core + CT + ICP > 22 mmHg + % Time ΔCPPopt-PRx > 10 mmHg | 0.737 (0.545 - 0.899) | 48.6 | 0.0513 | 0.25 | 0.678 (0.526 - 0.82) | 77.4 | **0.0415** | 0.128 | 1 (1 - 1) | 16 | **0.0166** | 1 |
| Core + CT + ICP > 22 mmHg + % Time ΔCPPopt-PRx < -5 mmHg | 0.838 (0.672 - 0.97) | 45.3 | **0.0293** | 0.368 | 0.658 (0.493 - 0.802) | 77.6 | **0.0477** | 0.123 | 1 (1 - 1) | 16 | **0.0164** | 1 |
| Core + CT + ICP > 22 mmHg + % Time ΔCPPopt-PRx < -10 mmHg | 0.768 (0.581 - 0.929) | 47.8 | **0.0401** | 0.281 | 0.66 (0.495 - 0.806) | 77.5 | **0.0477** | 0.127 | 0.974 (0.895 - 1) | 20.7 | **0.033** | 0.712 |
| Core + CT + ICP > 22 mmHg + % Time CPP > ULR-PRx | 0.646 (0.434 - 0.833) | 51.1 | 0.118 | 0.149 | 0.737 (0.583 - 0.871) | 72.7 | **0.02** | 0.237 | 0.974 (0.895 - 1) | 21.2 | **0.0199** | 0.68 |
| Core + CT + ICP > 22 mmHg + % Time CPP < LLR-PRx | 0.742 (0.545 - 0.904) | 47.9 | 0.0513 | 0.275 | 0.729 (0.58 - 0.864) | 73.5 | **0.0233** | 0.219 | 0.947 (0.789 - 1) | 22.6 | **0.0387** | 0.581 |
| Core + CT + ICP > 22 mmHg + CPPopt-PAx | 0.662 (0.429 - 0.859) | 51 | 0.1121 | 0.157 | 0.662 (0.516 - 0.797) | 78.1 | **0.0477** | 0.113 | 0.868 (0.579 - 1) | 23.5 | 0.0603 | 0.509 |
| Core + CT + ICP > 22 mmHg + % Time ΔCPPopt-PAx > 5 mmHg | 0.692 (0.49 - 0.874) | 49.2 | 0.0765 | 0.226 | 0.703 (0.552 - 0.835) | 76.6 | **0.0352** | 0.149 | 1 (1 - 1) | 16 | **0.0164** | 1 |
| Core + CT + ICP > 22 mmHg + % Time ΔCPPopt-PAx > 10 mmHg | 0.697 (0.495 - 0.879) | 48.9 | 0.0736 | 0.24 | 0.696 (0.544 - 0.833) | 77.2 | **0.0367** | 0.134 | 1 (1 - 1) | 16 | **0.0164** | 1 |
| Core + CT + ICP > 22 mmHg + % Time ΔCPPopt-PAx < -5 mmHg | 0.717 (0.52 - 0.884) | 49.6 | 0.0623 | 0.212 | 0.714 (0.574 - 0.843) | 74.3 | **0.0305** | 0.2 | 0.895 (0.684 - 1) | 23.4 | 0.0547 | 0.521 |
| Core + CT + ICP > 22 mmHg + % Time ΔCPPopt-PAx < -10 mmHg | 0.717 (0.525 - 0.894) | 49.7 | 0.0623 | 0.208 | 0.683 (0.534 - 0.815) | 76.7 | **0.041** | 0.146 | 1 (1 - 1) | 16 | **0.0164** | 1 |
| Core + CT + ICP > 22 mmHg + % Time CPP > ULR-PAx | 0.657 (0.444 - 0.848) | 51.2 | 0.1121 | 0.147 | 0.786 (0.65 - 0.897) | 69 | **0.0103** | 0.315 | 1 (1 - 1) | 16 | **0.0166** | 1 |
| Core + CT + ICP > 22 mmHg + % Time CPP < LLR-PAx | 0.798 (0.616 - 0.949) | 46 | **0.0293** | 0.345 | 0.675 (0.529 - 0.819) | 76.9 | **0.0416** | 0.141 | 0.895 (0.684 - 1) | 23.8 | 0.0547 | 0.49 |
| Core + CT + ICP > 22 mmHg + CPPopt-RAC | 0.667 (0.449 - 0.854) | 51.1 | 0.1109 | 0.148 | 0.644 (0.485 - 0.786) | 78 | 0.0562 | 0.114 | 0.868 (0.579 - 1) | 23.7 | 0.0603 | 0.496 |
| Core + CT + ICP > 22 mmHg + % Time ΔCPPopt-RAC > 5 mmHg | 0.722 (0.51 - 0.899) | 47.6 | 0.0604 | 0.288 | 0.68 (0.541 - 0.815) | 77.7 | **0.0415** | 0.121 | 1 (1 - 1) | 16 | **0.0164** | 1 |
| Core + CT + ICP > 22 mmHg + % Time ΔCPPopt-RAC > 10 mmHg | 0.737 (0.525 - 0.909) | 48.3 | 0.0513 | 0.261 | 0.662 (0.51 - 0.796) | 78.1 | **0.0477** | 0.111 | 1 (1 - 1) | 16 | **0.0166** | 1 |
| Core + CT + ICP > 22 mmHg + % Time ΔCPPopt-RAC < -5 mmHg | 0.722 (0.53 - 0.889) | 48.9 | 0.0604 | 0.239 | 0.675 (0.533 - 0.814) | 77.2 | **0.0416** | 0.133 | 0.895 (0.684 - 1) | 23.9 | 0.0547 | 0.482 |
| Core + CT + ICP > 22 mmHg + % Time ΔCPPopt-RAC < -10 mmHg | 0.672 (0.465 - 0.848) | 51 | 0.1051 | 0.156 | 0.663 (0.518 - 0.801) | 78 | **0.0477** | 0.114 | 0.895 (0.632 - 1) | 23.8 | 0.0547 | 0.482 |
| Core + CT + ICP > 22 mmHg + % Time CPP > ULR-RAC | 0.657 (0.439 - 0.838) | 51.2 | 0.1121 | 0.146 | 0.776 (0.647 - 0.889) | 70.8 | **0.0103** | 0.277 | 1 (1 - 1) | 16 | **0.0187** | 1 |
| Core + CT + ICP > 22 mmHg + % Time CPP < LLR-RAC | 0.818 (0.636 - 0.965) | 47.9 | **0.0293** | 0.278 | 0.779 (0.624 - 0.904) | 68.5 | **0.0103** | 0.324 | 0.895 (0.684 - 1) | 23.6 | 0.0547 | 0.498 |

*Core model consisted of age, admission Glasgow Coma Scale - motor score, and admission pupillary response. CT variables consisted of admission Marshall CT grade, presence of traumatic subarachnoid hemorrhage, and presence of epidural hematoma. All p-values have been adjusted using the False Discovery Rate (FDR) method. Bolded p-values are those reaching statistical significance, p < 0.05.*

*AIC = Akaike information criterion, AMP = pulse amplitude of ICP, AUC = area under the curve, CI = confidence interval, CPP = cerebral perfusion pressure, CPPopt = cerebral perfusion pressure optimum, ΔCPPopt = CPP - CPPopt, CT = computed tomography, GOSE = Glasgow Outcome Scale-Extended, ICP = intracranial pressure, IMPACT = International Mission for Prognosis and Analysis of Clinical Trials, mmHg = millimeters of mercury, LLR = lower limit of reactivity, PAx = pulse amplitude index, PRx = pressure reactivity index, RAC = correlation (R) between slow-waves of AMP (A) and CPP (C), ULR = upper limit of reactivity.*

Appendix V. Multivariable Models of Cerebrovascular Reactivity Measures for Transition in Outcome from 1 to 6 Months with Patients Trichotomized by Age

| **Model** | **Age < 30** | | | | **Age 30 – 60** | | | | **Age > 60** | | | |
| --- | --- | --- | --- | --- | --- | --- | --- | --- | --- | --- | --- | --- |
|  | **AUC (95% CI)** | **AIC** | **p-value** | **Nagelkerke's R^2^** | **AUC (95% CI)** | **AIC** | **p-value** | **Nagelkerke's R^2^** | **AUC (95% CI)** | **AIC** | **p-value** | **Nagelkerke's R^2^** |
| Core | 0.653 (0.4 - 0.879) | 45.4 | 0.0999 | 0.09 | 0.654 (0.494 - 0.799) | 77.7 | **0.0298** | 0.117 | 0.949 (0.824 - 1) | 20.2 | **0.0041** | 0.62 |
| Core + CPPopt-PRx | 0.642 (0.4 - 0.874) | 47.4 | 0.1142 | 0.091 | 0.697 (0.533 - 0.842) | 77.3 | **0.0113** | 0.172 | 1 (1 - 1) | 12 | **0.0041** | 1 |
| Core + % Time ΔCPPopt-PRx > 5 mmHg | 0.826 (0.642 - 0.968) | 38.8 | **0.002** | 0.422 | 0.651 (0.49 - 0.796) | 79.1 | **0.0314** | 0.131 | 0.941 (0.824 - 1) | 22.2 | **0.0041** | 0.622 |
| Core + % Time ΔCPPopt-PRx > 10 mmHg | 0.853 (0.674 - 0.979) | 37.9 | **0.0009** | 0.452 | 0.668 (0.519 - 0.81) | 78.5 | **0.0219** | 0.144 | 0.941 (0.824 - 1) | 21.4 | **0.0041** | 0.659 |
| Core + % Time ΔCPPopt-PRx < -5 mmHg | 0.674 (0.421 - 0.879) | 43.8 | 0.0739 | 0.24 | 0.655 (0.503 - 0.801) | 79.7 | **0.0287** | 0.117 | 1 (1 - 1) | 12 | **0.0041** | 1 |
| Core + % Time ΔCPPopt-PRx < -10 mmHg | 0.647 (0.384 - 0.889) | 44.5 | 0.1067 | 0.213 | 0.652 (0.5 - 0.797) | 79.7 | **0.0305** | 0.118 | 1 (1 - 1) | 12 | **0.0041** | 1 |
| Core + % Time CPP > ULR-PRx | 0.653 (0.4 - 0.874) | 47.4 | 0.1007 | 0.091 | 0.694 (0.548 - 0.826) | 77.8 | **0.0113** | 0.161 | 0.956 (0.853 - 1) | 20.5 | **0.0041** | 0.699 |
| Core + % Time CPP < LLR-PRx | 0.7 (0.447 - 0.916) | 44.3 | **0.0464** | 0.221 | 0.661 (0.501 - 0.806) | 79.7 | **0.0257** | 0.118 | 0.956 (0.868 - 1) | 22.1 | **0.0041** | 0.624 |
| Core + CPPopt-PAx | 0.663 (0.442 - 0.874) | 46.9 | 0.0872 | 0.113 | 0.696 (0.546 - 0.828) | 77 | **0.0113** | 0.178 | 0.941 (0.824 - 1) | 22.1 | **0.0041** | 0.624 |
| Core + % Time ΔCPPopt-PAx > 5 mmHg | 0.889 (0.737 - 1) | 35.7 | **0.0003** | 0.519 | 0.657 (0.504 - 0.803) | 79.3 | **0.0282** | 0.126 | 0.956 (0.824 - 1) | 22 | **0.0041** | 0.631 |
| Core + % Time ΔCPPopt-PAx > 10 mmHg | 0.868 (0.705 - 0.979) | 36.8 | **0.0005** | 0.487 | 0.678 (0.522 - 0.817) | 78.6 | **0.0163** | 0.143 | 0.941 (0.824 - 1) | 22.2 | **0.0041** | 0.623 |
| Core + % Time ΔCPPopt-PAx < -5 mmHg | 0.805 (0.595 - 0.963) | 40.9 | **0.0038** | 0.35 | 0.667 (0.507 - 0.807) | 79.4 | **0.0227** | 0.125 | 0.956 (0.824 - 1) | 22.2 | **0.0041** | 0.621 |
| Core + % Time ΔCPPopt-PAx < -10 mmHg | 0.742 (0.516 - 0.916) | 42 | **0.0194** | 0.311 | 0.675 (0.525 - 0.814) | 79.3 | **0.0177** | 0.126 | 0.956 (0.824 - 1) | 22.2 | **0.0041** | 0.62 |
| Core + % Time CPP > ULR-PAx | 0.758 (0.553 - 0.926) | 43.8 | **0.0135** | 0.24 | 0.658 (0.503 - 0.799) | 79.4 | **0.0274** | 0.123 | 0.956 (0.824 - 1) | 20.7 | **0.0041** | 0.69 |
| Core + % Time CPP < LLR-PAx | 0.895 (0.731 - 1) | 32.6 | **0.0002** | 0.606 | 0.661 (0.501 - 0.801) | 79.6 | **0.0257** | 0.12 | 0.956 (0.824 - 1) | 21 | **0.0041** | 0.675 |
| Core + CPPopt-RAC | 0.647 (0.405 - 0.874) | 47 | 0.1067 | 0.108 | 0.683 (0.522 - 0.816) | 77.2 | **0.0143** | 0.174 | 0.956 (0.824 - 1) | 22.2 | **0.0041** | 0.62 |
| Core + % Time ΔCPPopt-RAC > 5 mmHg | 0.832 (0.663 - 0.958) | 39.6 | **0.0017** | 0.395 | 0.661 (0.501 - 0.793) | 79.2 | **0.0257** | 0.13 | 0.941 (0.824 - 1) | 21.8 | **0.0041** | 0.639 |
| Core + % Time ΔCPPopt-RAC > 10 mmHg | 0.832 (0.653 - 0.979) | 39.2 | **0.0017** | 0.408 | 0.706 (0.561 - 0.841) | 77.6 | **0.0113** | 0.164 | 0.956 (0.824 - 1) | 22.2 | **0.0041** | 0.621 |
| Core + % Time ΔCPPopt-RAC < -5 mmHg | 0.821 (0.632 - 0.963) | 39.6 | **0.0023** | 0.395 | 0.655 (0.496 - 0.791) | 79.7 | **0.0287** | 0.117 | 0.956 (0.824 - 1) | 22 | **0.0041** | 0.632 |
| Core + % Time ΔCPPopt-RAC < -10 mmHg | 0.779 (0.584 - 0.926) | 41.2 | **0.008** | 0.337 | 0.658 (0.499 - 0.804) | 79.6 | **0.0274** | 0.118 | 0.956 (0.824 - 1) | 21.8 | **0.0041** | 0.641 |
| Core + % Time CPP > ULR-RAC | 0.763 (0.568 - 0.921) | 43.4 | **0.012** | 0.255 | 0.684 (0.538 - 0.812) | 77.8 | **0.0138** | 0.16 | 0.956 (0.824 - 1) | 20.7 | **0.0041** | 0.687 |
| Core + % Time CPP < LLR-RAC | 0.832 (0.626 - 0.979) | 36.5 | **0.0017** | 0.494 | 0.665 (0.522 - 0.804) | 79.1 | **0.0234** | 0.132 | 0.956 (0.824 - 1) | 21.1 | **0.0041** | 0.671 |
| Core + CT | 0.847 (0.679 - 0.989) | 38.2 | **0.0016** | 0.441 | 0.707 (0.562 - 0.849) | 78.2 | **0.0113** | 0.151 | 0.949 (0.824 - 1) | 21.8 | **0.0041** | 0.64 |
| Core + CT + CPPopt-PRx | 0.9 (0.774 - 0.989) | 35.4 | **0.0002** | 0.585 | 0.741 (0.591 - 0.872) | 78.1 | **0.0113** | 0.197 | 1 (1 - 1) | 14 | **0.0041** | 1 |
| Core + CT + % Time ΔCPPopt-PRx > 5 mmHg | 0.874 (0.721 - 0.979) | 37.4 | **0.0004** | 0.527 | 0.687 (0.536 - 0.82) | 79.5 | **0.0127** | 0.166 | 0.941 (0.824 - 1) | 23.8 | **0.0041** | 0.64 |
| Core + CT + % Time ΔCPPopt-PRx > 10 mmHg | 0.879 (0.726 - 0.979) | 37.3 | **0.0003** | 0.531 | 0.709 (0.554 - 0.843) | 78.7 | **0.0113** | 0.184 | 0.941 (0.824 - 1) | 23.3 | **0.0041** | 0.663 |
| Core + CT + % Time ΔCPPopt-PRx < -5 mmHg | 0.847 (0.663 - 0.979) | 39.5 | **0.001** | 0.462 | 0.709 (0.567 - 0.841) | 80.2 | **0.0113** | 0.151 | 1 (1 - 1) | 14 | **0.0041** | 1 |
| Core + CT + % Time ΔCPPopt-PRx < -10 mmHg | 0.858 (0.689 - 0.984) | 39.9 | **0.0008** | 0.45 | 0.703 (0.557 - 0.842) | 80.2 | **0.0113** | 0.152 | 1 (1 - 1) | 14 | **0.0041** | 1 |
| Core + CT + % Time CPP > ULR-PRx | 0.9 (0.774 - 0.984) | 36.3 | **0.0002** | 0.559 | 0.717 (0.58 - 0.846) | 78.6 | **0.0113** | 0.187 | 0.956 (0.838 - 1) | 22.3 | **0.0041** | 0.705 |
| Core + CT + % Time CPP < LLR-PRx | 0.847 (0.679 - 0.979) | 39.6 | **0.001** | 0.459 | 0.712 (0.571 - 0.845) | 80.2 | **0.0113** | 0.151 | 0.941 (0.824 - 1) | 23.8 | **0.0041** | 0.641 |
| Core + CT + CPPopt-PAx | 0.879 (0.732 - 0.979) | 37.7 | **0.0003** | 0.518 | 0.723 (0.578 - 0.851) | 77.8 | **0.0113** | 0.203 | 0.941 (0.824 - 1) | 23.6 | **0.0041** | 0.648 |
| Core + CT + % Time ΔCPPopt-PAx > 5 mmHg | 0.926 (0.816 - 0.995) | 31.4 | **<0.0001** | 0.686 | 0.72 (0.578 - 0.854) | 79.7 | **0.0113** | 0.162 | 0.956 (0.824 - 1) | 23.4 | **0.0041** | 0.658 |
| Core + CT + % Time ΔCPPopt-PAx > 10 mmHg | 0.953 (0.853 - 1) | 31.1 | **<0.0001** | 0.694 | 0.712 (0.567 - 0.845) | 79 | **0.0113** | 0.178 | 0.941 (0.824 - 1) | 23.8 | **0.0041** | 0.64 |
| Core + CT + % Time ΔCPPopt-PAx < -5 mmHg | 0.921 (0.795 - 1) | 33.1 | **<0.0001** | 0.645 | 0.707 (0.562 - 0.841) | 80 | **0.0113** | 0.156 | 0.941 (0.824 - 1) | 23.8 | **0.0041** | 0.64 |
| Core + CT + % Time ΔCPPopt-PAx < -10 mmHg | 0.895 (0.742 - 1) | 35.4 | **0.0002** | 0.584 | 0.704 (0.552 - 0.836) | 79.9 | **0.0113** | 0.158 | 0.941 (0.824 - 1) | 23.8 | **0.0041** | 0.641 |
| Core + CT + % Time CPP > ULR-PAx | 0.905 (0.789 - 0.989) | 35.9 | **0.0002** | 0.572 | 0.696 (0.539 - 0.838) | 79.9 | **0.0113** | 0.158 | 0.956 (0.824 - 1) | 22.5 | **0.0041** | 0.699 |
| Core + CT + % Time CPP < LLR-PAx | 0.958 (0.863 - 1) | 27.3 | **<0.0001** | 0.779 | 0.71 (0.561 - 0.841) | 80.2 | **0.0113** | 0.152 | 0.956 (0.824 - 1) | 23 | **0.0041** | 0.676 |
| Core + CT + CPPopt-RAC | 0.874 (0.732 - 0.979) | 37.9 | **0.0004** | 0.512 | 0.725 (0.568 - 0.854) | 78 | **0.0113** | 0.201 | 0.956 (0.824 - 1) | 23.8 | **0.0041** | 0.64 |
| Core + CT + % Time ΔCPPopt-RAC > 5 mmHg | 0.884 (0.742 - 0.984) | 35.9 | **0.0003** | 0.571 | 0.694 (0.548 - 0.828) | 79.7 | **0.0113** | 0.163 | 0.941 (0.824 - 1) | 22.8 | **0.0041** | 0.683 |
| Core + CT + % Time ΔCPPopt-RAC > 10 mmHg | 0.884 (0.737 - 0.984) | 35 | **0.0003** | 0.596 | 0.729 (0.578 - 0.859) | 78 | **0.0113** | 0.2 | 0.956 (0.824 - 1) | 23.5 | **0.0041** | 0.652 |
| Core + CT + % Time ΔCPPopt-RAC < -5 mmHg | 0.916 (0.789 - 0.995) | 33.8 | **<0.0001** | 0.627 | 0.709 (0.559 - 0.848) | 80.2 | **0.0113** | 0.151 | 0.956 (0.824 - 1) | 23.6 | **0.0041** | 0.65 |
| Core + CT + % Time ΔCPPopt-RAC < -10 mmHg | 0.879 (0.726 - 0.984) | 37.6 | **0.0003** | 0.522 | 0.7 (0.562 - 0.839) | 80.1 | **0.0113** | 0.153 | 0.956 (0.824 - 1) | 23.4 | **0.0041** | 0.659 |
| Core + CT + % Time CPP > ULR-RAC | 0.9 (0.758 - 1) | 35.9 | **0.0002** | 0.57 | 0.719 (0.572 - 0.849) | 78 | **0.0113** | 0.199 | 0.956 (0.824 - 1) | 22.6 | **0.0041** | 0.692 |
| Core + CT + % Time CPP < LLR-RAC | 0.916 (0.784 - 1) | 32.7 | **<0.0001** | 0.654 | 0.72 (0.572 - 0.852) | 79.1 | **0.0113** | 0.176 | 0.956 (0.824 - 1) | 23.1 | **0.0041** | 0.671 |
| Core + CT + ICP > 20 mmHg | 0.9 (0.747 - 1) | 36 | **0.0002** | 0.566 | 0.693 (0.535 - 0.835) | 79.7 | **0.0113** | 0.162 | 0.941 (0.824 - 1) | 23.1 | **0.0041** | 0.673 |
| Core + CT + ICP > 20 mmHg + CPPopt-PRx | 0.911 (0.779 - 0.989) | 35.3 | **0.0002** | 0.639 | 0.733 (0.581 - 0.865) | 80 | **0.0113** | 0.201 | 1 (1 - 1) | 16 | **0.0041** | 1 |
| Core + CT + ICP > 20 mmHg + % Time ΔCPPopt-PRx > 5 mmHg | 0.895 (0.758 - 0.989) | 37.2 | **0.0002** | 0.589 | 0.691 (0.545 - 0.822) | 81.2 | **0.0113** | 0.174 | 0.941 (0.809 - 1) | 25 | **0.0041** | 0.673 |
| Core + CT + ICP > 20 mmHg + % Time ΔCPPopt-PRx > 10 mmHg | 0.9 (0.758 - 0.995) | 37.1 | **0.0002** | 0.591 | 0.712 (0.561 - 0.846) | 80.4 | **0.0113** | 0.192 | 0.956 (0.853 - 1) | 24.7 | **0.0041** | 0.69 |
| Core + CT + ICP > 20 mmHg + % Time ΔCPPopt-PRx < -5 mmHg | 0.905 (0.763 - 0.995) | 37.8 | **0.0002** | 0.574 | 0.7 (0.549 - 0.838) | 81.6 | **0.0113** | 0.164 | 1 (1 - 1) | 16 | **0.0041** | 1 |
| Core + CT + ICP > 20 mmHg + % Time ΔCPPopt-PRx < -10 mmHg | 0.916 (0.789 - 1) | 37.5 | **<0.0001** | 0.583 | 0.701 (0.558 - 0.841) | 81.4 | **0.0113** | 0.168 | 1 (1 - 1) | 16 | **0.0041** | 1 |
| Core + CT + ICP > 20 mmHg + % Time CPP > ULR-PRx | 0.932 (0.816 - 1) | 32.1 | **<0.0001** | 0.717 | 0.716 (0.574 - 0.841) | 80.3 | **0.0113** | 0.193 | 0.956 (0.824 - 1) | 22 | **0.0041** | 0.801 |
| Core + CT + ICP > 20 mmHg + % Time CPP < LLR-PRx | 0.895 (0.747 - 1) | 38 | **0.0002** | 0.567 | 0.694 (0.548 - 0.83) | 81.7 | **0.0113** | 0.162 | 0.941 (0.809 - 1) | 25 | **0.0041** | 0.676 |
| Core + CT + ICP > 20 mmHg + CPPopt-PAx | 0.921 (0.816 - 1) | 36.1 | **<0.0001** | 0.619 | 0.72 (0.558 - 0.857) | 79.8 | **0.0113** | 0.204 | 0.956 (0.824 - 1) | 24.6 | **0.0041** | 0.693 |
| Core + CT + ICP > 20 mmHg + % Time ΔCPPopt-PAx > 5 mmHg | 0.926 (0.816 - 0.995) | 32.5 | **<0.0001** | 0.709 | 0.706 (0.561 - 0.845) | 81.2 | **0.0113** | 0.173 | 0.941 (0.824 - 1) | 24.4 | **0.0041** | 0.703 |
| Core + CT + ICP > 20 mmHg + % Time ΔCPPopt-PAx > 10 mmHg | 0.947 (0.853 - 1) | 31.7 | **<0.0001** | 0.726 | 0.701 (0.549 - 0.833) | 80.5 | **0.0113** | 0.189 | 0.941 (0.809 - 1) | 25.1 | **0.0041** | 0.673 |
| Core + CT + ICP > 20 mmHg + % Time ΔCPPopt-PAx < -5 mmHg | 0.926 (0.795 - 1) | 34.4 | **<0.0001** | 0.664 | 0.7 (0.546 - 0.838) | 81.6 | **0.0113** | 0.165 | 0.941 (0.809 - 1) | 24.7 | **0.0041** | 0.688 |
| Core + CT + ICP > 20 mmHg + % Time ΔCPPopt-PAx < -10 mmHg | 0.905 (0.747 - 1) | 36.3 | **0.0002** | 0.614 | 0.694 (0.548 - 0.828) | 81.4 | **0.0113** | 0.168 | 0.941 (0.824 - 1) | 24.5 | **0.0041** | 0.699 |
| Core + CT + ICP > 20 mmHg + % Time CPP > ULR-PAx | 0.916 (0.789 - 1) | 35.3 | **<0.0001** | 0.64 | 0.699 (0.552 - 0.839) | 81.2 | **0.0113** | 0.174 | 0.956 (0.824 - 1) | 22.6 | **0.0041** | 0.776 |
| Core + CT + ICP > 20 mmHg + % Time CPP < LLR-PAx | 0.968 (0.889 - 1) | 26.7 | **<0.0001** | 0.83 | 0.693 (0.545 - 0.829) | 81.7 | **0.0113** | 0.162 | 0.971 (0.882 - 1) | 23.8 | **0.0041** | 0.727 |
| Core + CT + ICP > 20 mmHg + CPPopt-RAC | 0.905 (0.768 - 0.989) | 36.3 | **0.0002** | 0.615 | 0.72 (0.577 - 0.851) | 79.9 | **0.0113** | 0.201 | 0.941 (0.824 - 1) | 25 | **0.0041** | 0.674 |
| Core + CT + ICP > 20 mmHg + % Time ΔCPPopt-RAC > 5 mmHg | 0.905 (0.779 - 0.989) | 35.7 | **0.0002** | 0.629 | 0.691 (0.529 - 0.832) | 81.2 | **0.0113** | 0.173 | 0.956 (0.853 - 1) | 23.4 | **0.0041** | 0.743 |
| Core + CT + ICP > 20 mmHg + % Time ΔCPPopt-RAC > 10 mmHg | 0.926 (0.821 - 1) | 34.1 | **<0.0001** | 0.671 | 0.725 (0.584 - 0.855) | 79.6 | **0.0113** | 0.209 | 0.941 (0.824 - 1) | 24.6 | **0.0041** | 0.694 |
| Core + CT + ICP > 20 mmHg + % Time ΔCPPopt-RAC < -5 mmHg | 0.926 (0.811 - 1) | 34.9 | **<0.0001** | 0.65 | 0.693 (0.539 - 0.835) | 81.7 | **0.0113** | 0.162 | 0.941 (0.824 - 1) | 25 | **0.0041** | 0.673 |
| Core + CT + ICP > 20 mmHg + % Time ΔCPPopt-RAC < -10 mmHg | 0.9 (0.758 - 1) | 37.8 | **0.0002** | 0.574 | 0.691 (0.538 - 0.828) | 81.7 | **0.0113** | 0.164 | 0.941 (0.823 - 1) | 24.9 | **0.0041** | 0.678 |
| Core + CT + ICP > 20 mmHg + % Time CPP > ULR-RAC | 0.905 (0.774 - 0.995) | 36 | **0.0002** | 0.623 | 0.72 (0.571 - 0.849) | 78.1 | **0.0113** | 0.24 | 0.956 (0.824 - 1) | 22.4 | **0.0041** | 0.782 |
| Core + CT + ICP > 20 mmHg + % Time CPP < LLR-RAC | 0.911 (0.779 - 1) | 34.7 | **0.0002** | 0.655 | 0.722 (0.577 - 0.851) | 79.3 | **0.0113** | 0.215 | 0.956 (0.838 - 1) | 24.5 | **0.0041** | 0.695 |
| Core + CT + ICP > 22 mmHg | 0.9 (0.737 - 1) | 35.7 | **0.0002** | 0.576 | 0.694 (0.546 - 0.83) | 79.5 | **0.0113** | 0.168 | 0.941 (0.824 - 1) | 23.2 | **0.0041** | 0.667 |
| Core + CT + ICP > 22 mmHg + CPPopt-PRx | 0.937 (0.832 - 1) | 35 | **<0.0001** | 0.648 | 0.728 (0.583 - 0.861) | 79.8 | **0.0113** | 0.204 | 1 (1 - 1) | 16 | **0.0041** | 1 |
| Core + CT + ICP > 22 mmHg + % Time ΔCPPopt-PRx > 5 mmHg | 0.905 (0.758 - 1) | 37 | **0.0002** | 0.594 | 0.691 (0.542 - 0.823) | 81 | **0.0113** | 0.179 | 0.941 (0.824 - 1) | 25.2 | **0.0041** | 0.667 |
| Core + CT + ICP > 22 mmHg + % Time ΔCPPopt-PRx > 10 mmHg | 0.9 (0.758 - 0.989) | 36.9 | **0.0002** | 0.598 | 0.71 (0.565 - 0.838) | 80.1 | **0.0113** | 0.197 | 0.956 (0.838 - 1) | 24.7 | **0.0041** | 0.69 |
| Core + CT + ICP > 22 mmHg + % Time ΔCPPopt-PRx < -5 mmHg | 0.916 (0.774 - 1) | 37.2 | **<0.0001** | 0.589 | 0.703 (0.548 - 0.841) | 81.4 | **0.0113** | 0.17 | 1 (1 - 1) | 16 | **0.0041** | 1 |
| Core + CT + ICP > 22 mmHg + % Time ΔCPPopt-PRx < -10 mmHg | 0.905 (0.779 - 1) | 36.9 | **0.0002** | 0.599 | 0.707 (0.554 - 0.845) | 81.1 | **0.0113** | 0.175 | 1 (1 - 1) | 16 | **0.0041** | 1 |
| Core + CT + ICP > 22 mmHg + % Time CPP > ULR-PRx | 0.942 (0.842 - 1) | 32.1 | **<0.0001** | 0.718 | 0.719 (0.574 - 0.851) | 80.1 | **0.0113** | 0.198 | 1 (1 - 1) | 16 | **0.0041** | 1 |
| Core + CT + ICP > 22 mmHg + % Time CPP < LLR-PRx | 0.905 (0.747 - 1) | 37.7 | **0.0002** | 0.577 | 0.694 (0.545 - 0.829) | 81.5 | **0.0113** | 0.168 | 0.941 (0.824 - 1) | 25.2 | **0.0041** | 0.667 |
| Core + CT + ICP > 22 mmHg + CPPopt-PAx | 0.916 (0.795 - 0.995) | 35.8 | **<0.0001** | 0.628 | 0.72 (0.577 - 0.849) | 79.7 | **0.0113** | 0.205 | 0.956 (0.824 - 1) | 24.8 | **0.0041** | 0.685 |
| Core + CT + ICP > 22 mmHg + % Time ΔCPPopt-PAx > 5 mmHg | 0.932 (0.821 - 1) | 32.7 | **<0.0001** | 0.703 | 0.706 (0.558 - 0.836) | 81 | **0.0113** | 0.179 | 0.941 (0.824 - 1) | 24.8 | **0.0041** | 0.686 |
| Core + CT + ICP > 22 mmHg + % Time ΔCPPopt-PAx > 10 mmHg | 0.942 (0.847 - 1) | 31.7 | **<0.0001** | 0.727 | 0.706 (0.552 - 0.846) | 80.2 | **0.0113** | 0.194 | 0.941 (0.824 - 1) | 25.2 | **0.0041** | 0.668 |
| Core + CT + ICP > 22 mmHg + % Time ΔCPPopt-PAx < -5 mmHg | 0.926 (0.789 - 1) | 34.5 | **<0.0001** | 0.661 | 0.701 (0.548 - 0.841) | 81.3 | **0.0113** | 0.17 | 0.956 (0.824 - 1) | 24.9 | **0.0041** | 0.681 |
| Core + CT + ICP > 22 mmHg + % Time ΔCPPopt-PAx < -10 mmHg | 0.911 (0.747 - 1) | 36.2 | **0.0002** | 0.616 | 0.699 (0.542 - 0.832) | 81.2 | **0.0113** | 0.173 | 0.956 (0.824 - 1) | 24.6 | **0.0041** | 0.694 |
| Core + CT + ICP > 22 mmHg + % Time CPP > ULR-PAx | 0.921 (0.795 - 1) | 34.9 | **<0.0001** | 0.65 | 0.7 (0.549 - 0.839) | 80.9 | **0.0113** | 0.18 | 1 (1 - 1) | 16 | **0.0041** | 1 |
| Core + CT + ICP > 22 mmHg + % Time CPP < LLR-PAx | 0.963 (0.879 - 1) | 29 | **<0.0001** | 0.785 | 0.7 (0.561 - 0.839) | 81.5 | **0.0113** | 0.168 | 0.971 (0.882 - 1) | 23.7 | **0.0041** | 0.732 |
| Core + CT + ICP > 22 mmHg + CPPopt-RAC | 0.916 (0.795 - 0.995) | 35.7 | **<0.0001** | 0.63 | 0.722 (0.587 - 0.852) | 79.8 | **0.0113** | 0.203 | 0.941 (0.824 - 1) | 25.2 | **0.0041** | 0.667 |
| Core + CT + ICP > 22 mmHg + % Time ΔCPPopt-RAC > 5 mmHg | 0.916 (0.795 - 0.989) | 35.9 | **<0.0001** | 0.624 | 0.69 (0.533 - 0.823) | 81 | **0.0116** | 0.179 | 0.956 (0.853 - 1) | 24.1 | **0.0041** | 0.716 |
| Core + CT + ICP > 22 mmHg + % Time ΔCPPopt-RAC > 10 mmHg | 0.911 (0.784 - 0.995) | 34.5 | **0.0002** | 0.661 | 0.728 (0.575 - 0.858) | 79.3 | **0.0113** | 0.214 | 0.956 (0.824 - 1) | 24.9 | **0.0041** | 0.679 |
| Core + CT + ICP > 22 mmHg + % Time ΔCPPopt-RAC < -5 mmHg | 0.921 (0.795 - 1) | 34.8 | **<0.0001** | 0.653 | 0.694 (0.546 - 0.829) | 81.5 | **0.0113** | 0.168 | 0.941 (0.824 - 1) | 25.2 | **0.0041** | 0.667 |
| Core + CT + ICP > 22 mmHg + % Time ΔCPPopt-RAC < -10 mmHg | 0.905 (0.753 - 1) | 37.5 | **0.0002** | 0.581 | 0.693 (0.545 - 0.829) | 81.4 | **0.0113** | 0.169 | 0.941 (0.824 - 1) | 25 | **0.0041** | 0.674 |
| Core + CT + ICP > 22 mmHg + % Time CPP > ULR-RAC | 0.911 (0.779 - 1) | 35.8 | **0.0002** | 0.627 | 0.733 (0.59 - 0.857) | 77.4 | **0.0113** | 0.255 | 1 (1 - 1) | 16 | **0.0041** | 1 |
| Core + CT + ICP > 22 mmHg + % Time CPP < LLR-RAC | 0.916 (0.789 - 1) | 34.7 | **<0.0001** | 0.654 | 0.72 (0.572 - 0.854) | 78.9 | **0.0113** | 0.224 | 0.956 (0.838 - 1) | 24.5 | **0.0041** | 0.695 |

*Core model consisted of age, admission Glasgow Coma Scale - motor score, and admission pupillary response. CT variables consisted of admission Marshall CT grade, presence of traumatic subarachnoid hemorrhage, and presence of epidural hematoma. All p-values have been adjusted using the False Discovery Rate (FDR) method. Bolded p-values are those reaching statistical significance, p < 0.05.*

*AIC = Akaike information criterion, AMP = pulse amplitude of ICP, AUC = area under the curve, CI = confidence interval, CPP = cerebral perfusion pressure, CPPopt = cerebral perfusion pressure optimum, ΔCPPopt = CPP - CPPopt, CT = computed tomography, GOSE = Glasgow Outcome Scale-Extended, ICP = intracranial pressure, IMPACT = International Mission for Prognosis and Analysis of Clinical Trials, mmHg = millimeters of mercury, LLR = lower limit of reactivity, PAx = pulse amplitude index, PRx = pressure reactivity index, RAC = correlation (R) between slow-waves of AMP (A) and CPP (C), ULR = upper limit of reactivity.*

Appendix W. Added Variance in Transition in Outcome of Cerebrovascular Reactivity Measures Over IMPACT Core ± CT ± ICP > 20 or 22 mmHg with Those Who Died (GOSE=1) Removed

| **Δ Nagelkerke’s R^2^** | | | |
| --- | --- | --- | --- |
| **Core** | | | |
| **Variable** | **1 Month 🡪 3 Month** | **3 Month 🡪 6 Month** | **1 Month 🡪 6 Month** |
| CPPopt-PRx | 0.003 | 0.041 | 0.005 |
| % Time ΔCPPopt-PRx > 5 mmHg | 0 | 0.017 | 0.031 |
| % Time ΔCPPopt-PRx > 10 mmHg | 0.004 | 0.016 | 0.039 |
| % Time ΔCPPopt-PRx < -5 mmHg | 0.001 | 0.008 | 0.024 |
| % Time ΔCPPopt-PRx < -10 mmHg | 0.002 | 0.001 | 0.009 |
| % Time CPP > ULR-PRx | 0.07 | 0.125 | 0.009 |
| % Time CPP < LLR-PRx | 0.012 | 0.013 | 0.026 |
| CPPopt-PAx | 0.001 | 0.019 | 0.032 |
| % Time ΔCPPopt-PAx > 5 mmHg | 0.001 | 0.056 | 0.018 |
| % Time ΔCPPopt-PAx > 10 mmHg | 0.001 | 0.046 | 0.029 |
| % Time ΔCPPopt-PAx < -5 mmHg | 0.046 | 0.066 | 0.004 |
| % Time ΔCPPopt-PAx < -10 mmHg | 0.03 | 0.039 | 0.005 |
| % Time CPP > ULR-PAx | 0.132 | 0.196 | 0.002 |
| % Time CPP < LLR-PAx | 0.039 | 0.025 | 0.001 |
| CPPopt-RAC | 0.003 | 0.016 | 0.026 |
| % Time ΔCPPopt-RAC > 5 mmHg | 0.01 | 0.042 | 0.004 |
| % Time ΔCPPopt-RAC > 10 mmHg | 0.002 | 0.017 | 0.03 |
| % Time ΔCPPopt-RAC < -5 mmHg | 0.05 | 0.014 | 0 |
| % Time ΔCPPopt-RAC < -10 mmHg | 0.014 | 0 | 0 |
| % Time CPP > ULR-RAC | 0.073 | 0.152 | 0.025 |
| % Time CPP < LLR-RAC | 0.003 | 0.068 | 0.024 |
| **Core + CT** | | | |
| **Variable** | **1 Month 🡪 3 Month** | **3 Month 🡪 6 Month** | **1 Month 🡪 6 Month** |
| CPPopt-PRx | 0.001 | 0.037 | 0.013 |
| % Time ΔCPPopt-PRx > 5 mmHg | 0 | 0.017 | 0.027 |
| % Time ΔCPPopt-PRx > 10 mmHg | 0.007 | 0.012 | 0.043 |
| % Time ΔCPPopt-PRx < -5 mmHg | 0.011 | 0.014 | 0.003 |
| % Time ΔCPPopt-PRx < -10 mmHg | 0.016 | 0.006 | 0 |
| % Time CPP > ULR-PRx | 0.059 | 0.132 | 0.014 |
| % Time CPP < LLR-PRx | 0.002 | 0.007 | 0.009 |
| CPPopt-PAx | 0 | 0.017 | 0.02 |
| % Time ΔCPPopt-PAx > 5 mmHg | 0.008 | 0.062 | 0.042 |
| % Time ΔCPPopt-PAx > 10 mmHg | 0.009 | 0.049 | 0.062 |
| % Time ΔCPPopt-PAx < -5 mmHg | 0.034 | 0.076 | 0 |
| % Time ΔCPPopt-PAx < -10 mmHg | 0.027 | 0.047 | 0.002 |
| % Time CPP > ULR-PAx | 0.115 | 0.178 | 0.005 |
| % Time CPP < LLR-PAx | 0.03 | 0.01 | 0.001 |
| CPPopt-RAC | 0.001 | 0.014 | 0.018 |
| % Time ΔCPPopt-RAC > 5 mmHg | 0.006 | 0.046 | 0.009 |
| % Time ΔCPPopt-RAC > 10 mmHg | 0.009 | 0.017 | 0.055 |
| % Time ΔCPPopt-RAC < -5 mmHg | 0.043 | 0.02 | 0.001 |
| % Time ΔCPPopt-RAC < -10 mmHg | 0.015 | 0.002 | 0 |
| % Time CPP > ULR-RAC | 0.06 | 0.14 | 0.029 |
| % Time CPP < LLR-RAC | 0.002 | 0.057 | 0.027 |
| **Core + CT + ICP > 20 mmHg** | | | |
| **Variable** | **1 Month 🡪 3 Month** | **3 Month 🡪 6 Month** | **1 Month 🡪 6 Month** |
| CPPopt-PRx | 0.001 | 0.025 | 0.014 |
| % Time ΔCPPopt-PRx > 5 mmHg | 0 | 0.012 | 0.027 |
| % Time ΔCPPopt-PRx > 10 mmHg | 0.007 | 0.008 | 0.046 |
| % Time ΔCPPopt-PRx < -5 mmHg | 0.012 | 0.02 | 0.006 |
| % Time ΔCPPopt-PRx < -10 mmHg | 0.019 | 0.017 | 0.001 |
| % Time CPP > ULR-PRx | 0.058 | 0.135 | 0.018 |
| % Time CPP < LLR-PRx | 0.002 | 0.009 | 0.009 |
| CPPopt-PAx | 0 | 0.016 | 0.027 |
| % Time ΔCPPopt-PAx > 5 mmHg | 0.01 | 0.037 | 0.034 |
| % Time ΔCPPopt-PAx > 10 mmHg | 0.011 | 0.027 | 0.055 |
| % Time ΔCPPopt-PAx < -5 mmHg | 0.033 | 0.073 | 0 |
| % Time ΔCPPopt-PAx < -10 mmHg | 0.028 | 0.054 | 0.001 |
| % Time CPP > ULR-PAx | 0.113 | 0.138 | 0.003 |
| % Time CPP < LLR-PAx | 0.028 | 0.003 | 0.004 |
| CPPopt-RAC | 0.001 | 0.012 | 0.024 |
| % Time ΔCPPopt-RAC > 5 mmHg | 0.005 | 0.033 | 0.006 |
| % Time ΔCPPopt-RAC > 10 mmHg | 0.01 | 0.008 | 0.05 |
| % Time ΔCPPopt-RAC < -5 mmHg | 0.045 | 0.024 | 0.002 |
| % Time ΔCPPopt-RAC < -10 mmHg | 0.018 | 0.009 | 0 |
| % Time CPP > ULR-RAC | 0.058 | 0.111 | 0.028 |
| % Time CPP < LLR-RAC | 0.001 | 0.044 | 0.021 |
| **Core + CT + ICP > 22 mmHg** | | | |
| **Variable** | **1 Month 🡪 3 Month** | **3 Month 🡪 6 Month** | **1 Month 🡪 6 Month** |
| CPPopt-PRx | 0 | 0.02 | 0.012 |
| % Time ΔCPPopt-PRx > 5 mmHg | 0 | 0.011 | 0.027 |
| % Time ΔCPPopt-PRx > 10 mmHg | 0.009 | 0.007 | 0.045 |
| % Time ΔCPPopt-PRx < -5 mmHg | 0.012 | 0.023 | 0.006 |
| % Time ΔCPPopt-PRx < -10 mmHg | 0.02 | 0.019 | 0.001 |
| % Time CPP > ULR-PRx | 0.05 | 0.122 | 0.016 |
| % Time CPP < LLR-PRx | 0.002 | 0.007 | 0.009 |
| CPPopt-PAx | 0 | 0.011 | 0.024 |
| % Time ΔCPPopt-PAx > 5 mmHg | 0.011 | 0.041 | 0.036 |
| % Time ΔCPPopt-PAx > 10 mmHg | 0.016 | 0.025 | 0.055 |
| % Time ΔCPPopt-PAx < -5 mmHg | 0.033 | 0.078 | 0 |
| % Time ΔCPPopt-PAx < -10 mmHg | 0.029 | 0.057 | 0.001 |
| % Time CPP > ULR-PAx | 0.1 | 0.131 | 0.003 |
| % Time CPP < LLR-PAx | 0.025 | 0.003 | 0.003 |
| CPPopt-RAC | 0.002 | 0.008 | 0.022 |
| % Time ΔCPPopt-RAC > 5 mmHg | 0.007 | 0.037 | 0.007 |
| % Time ΔCPPopt-RAC > 10 mmHg | 0.011 | 0.009 | 0.052 |
| % Time ΔCPPopt-RAC < -5 mmHg | 0.048 | 0.025 | 0.002 |
| % Time ΔCPPopt-RAC < -10 mmHg | 0.021 | 0.01 | 0 |
| % Time CPP > ULR-RAC | 0.05 | 0.105 | 0.028 |
| % Time CPP < LLR-RAC | 0.001 | 0.043 | 0.022 |

*Core model consisted of age, admission Glasgow Coma Scale - motor score, and admission pupillary response. CT variables consisted of admission Marshall CT grade, presence of traumatic subarachnoid hemorrhage, and presence of epidural hematoma.*

*AMP = pulse amplitude of ICP, CPP = cerebral perfusion pressure, CPPopt = cerebral perfusion pressure optimum, ΔCPPopt = CPP - CPPopt, CT = computed tomography, GOSE = Glasgow Outcome Scale-Extended, ICP = intracranial pressure, IMPACT = International Mission for Prognosis and Analysis of Clinical Trials, LLR = lower limit of reactivity, PAx = pulse amplitude index, PRx = pressure reactivity index, RAC = correlation (R) between slow-waves of AMP (A) and CPP (C), ULR = upper limit of reactivity.*

Appendix X. Added Variance in Transition in Outcome of Cerebrovascular Reactivity Measures Over IMPACT Core ± CT ± ICP > 20 or 22 mmHg with Patients Trichotomized by Age

| **Δ Nagelkerke’s R2** | | | | | | | | | |
| --- | --- | --- | --- | --- | --- | --- | --- | --- | --- |
| **Core** | | | | | | | | | |
| **Variable** | **Age < 30** | | | **Age 30 – 60** | | | **Age > 60** | | |
|  | 1M 🡪 3M | 3M 🡪 6M | 1M 🡪 6M | 1M 🡪 3M | 3M 🡪 6M | 1M 🡪 6M | 1M 🡪 3M | 3M 🡪 6M | 1M 🡪 6M |
| CPPopt-PRx | 0.001 | 0.016 | 0.001 | 0.004 | 0.036 | 0.055 | 0.388 | 0.06 | 0.38 |
| % Time ΔCPPopt-PRx > 5 mmHg | 0.096 | 0.236 | 0.332 | 0.003 | 0.008 | 0.014 | 0.004 | 0.655 | 0.003 |
| % Time ΔCPPopt-PRx > 10 mmHg | 0.086 | 0.208 | 0.361 | 0.03 | 0.008 | 0.028 | 0 | 0.655 | 0.039 |
| % Time ΔCPPopt-PRx < -5 mmHg | 0.029 | 0.278 | 0.15 | 0.004 | 0.006 | 0.001 | 0.388 | 0.156 | 0.38 |
| % Time ΔCPPopt-PRx < -10 mmHg | 0.048 | 0.175 | 0.123 | 0.008 | 0.004 | 0.001 | 0.388 | 0.306 | 0.38 |
| % Time CPP > ULR-PRx | 0.09 | 0.028 | 0.001 | 0.001 | 0.135 | 0.044 | 0.009 | 0.3 | 0.079 |
| % Time CPP < LLR-PRx | 0.078 | 0.175 | 0.131 | 0.002 | 0.091 | 0.001 | 0.028 | 0.001 | 0.004 |
| CPPopt-PAx | 0.012 | 0.06 | 0.023 | 0.006 | 0.004 | 0.061 | 0.012 | 0.015 | 0.004 |
| % Time ΔCPPopt-PAx > 5 mmHg | 0.194 | 0.149 | 0.429 | 0.006 | 0.045 | 0.009 | 0.044 | 0.655 | 0.011 |
| % Time ΔCPPopt-PAx > 10 mmHg | 0.087 | 0.177 | 0.396 | 0.023 | 0.027 | 0.027 | 0.007 | 0.655 | 0.003 |
| % Time ΔCPPopt-PAx < -5 mmHg | 0.1 | 0.095 | 0.26 | 0.038 | 0.085 | 0.008 | 0 | 0.012 | 0.001 |
| % Time ΔCPPopt-PAx < -10 mmHg | 0.065 | 0.09 | 0.22 | 0.019 | 0.035 | 0.009 | 0 | 0.046 | 0 |
| % Time CPP > ULR-PAx | 0.327 | 0.009 | 0.149 | 0.049 | 0.18 | 0.006 | 0.024 | 0.655 | 0.07 |
| % Time CPP < LLR-PAx | 0.318 | 0.235 | 0.515 | 0.051 | 0.021 | 0.003 | 0 | 0.069 | 0.056 |
| CPPopt-RAC | 0.02 | 0.037 | 0.018 | 0.01 | 0.008 | 0.058 | 0.001 | 0.02 | 0 |
| % Time ΔCPPopt-RAC > 5 mmHg | 0.058 | 0.209 | 0.305 | 0.001 | 0.012 | 0.013 | 0.098 | 0.655 | 0.019 |
| % Time ΔCPPopt-RAC > 10 mmHg | 0.057 | 0.196 | 0.318 | 0.045 | 0.001 | 0.048 | 0.054 | 0.655 | 0.001 |
| % Time ΔCPPopt-RAC < -5 mmHg | 0.076 | 0.14 | 0.305 | 0.023 | 0.014 | 0 | 0.028 | 0.003 | 0.012 |
| % Time ΔCPPopt-RAC < -10 mmHg | 0.096 | 0.06 | 0.247 | 0.008 | 0 | 0.002 | 0.035 | 0.002 | 0.021 |
| % Time CPP > ULR-RAC | 0.419 | 0.008 | 0.164 | 0.004 | 0.121 | 0.043 | 0.033 | 0.41 | 0.067 |
| % Time CPP < LLR-RAC | 0.22 | 0.163 | 0.403 | 0.01 | 0.137 | 0.015 | 0.004 | 0.003 | 0.051 |
| **Core + CT** | | | | | | | | | |
| **Variable** | **Age < 30** | | | **Age 30 – 60** | | | **Age > 60** | | |
|  | 1M 🡪 3M | 3M 🡪 6M | 1M 🡪 6M | 1M 🡪 3M | 3M 🡪 6M | 1M 🡪 6M | 1M 🡪 3M | 3M 🡪 6M | 1M 🡪 6M |
| CPPopt-PRx | 0.058 | 0 | 0.144 | 0.002 | 0.043 | 0.047 | 0.321 | 0.044 | 0.36 |
| % Time ΔCPPopt-PRx > 5 mmHg | 0.014 | 0.153 | 0.085 | 0.004 | 0.009 | 0.015 | 0.001 | 0.52 | 0 |
| % Time ΔCPPopt-PRx > 10 mmHg | 0.001 | 0.118 | 0.089 | 0.036 | 0.011 | 0.034 | 0.004 | 0.52 | 0.023 |
| % Time ΔCPPopt-PRx < -5 mmHg | 0.001 | 0.223 | 0.021 | 0.005 | 0.006 | 0.001 | 0.321 | 0.52 | 0.36 |
| % Time ΔCPPopt-PRx < -10 mmHg | 0.006 | 0.137 | 0.009 | 0.01 | 0.004 | 0.002 | 0.321 | 0.23 | 0.36 |
| % Time CPP > ULR-PRx | 0.375 | 0 | 0.117 | 0.002 | 0.142 | 0.037 | 0.001 | 0.166 | 0.065 |
| % Time CPP < LLR-PRx | 0.03 | 0.143 | 0.018 | 0.002 | 0.087 | 0 | 0.001 | 0.101 | 0.001 |
| CPPopt-PAx | 0.016 | 0.009 | 0.077 | 0.004 | 0.007 | 0.053 | 0.002 | 0.028 | 0.008 |
| % Time ΔCPPopt-PAx > 5 mmHg | 0.094 | 0.092 | 0.245 | 0.009 | 0.041 | 0.012 | 0.039 | 0.52 | 0.018 |
| % Time ΔCPPopt-PAx > 10 mmHg | 0.018 | 0.106 | 0.252 | 0.024 | 0.025 | 0.028 | 0.005 | 0.52 | 0 |
| % Time ΔCPPopt-PAx < -5 mmHg | 0.068 | 0.082 | 0.204 | 0.031 | 0.079 | 0.005 | 0.009 | 0.036 | 0 |
| % Time ΔCPPopt-PAx < -10 mmHg | 0.038 | 0.078 | 0.143 | 0.016 | 0.032 | 0.007 | 0.008 | 0.093 | 0.001 |
| % Time CPP > ULR-PAx | 0.318 | 0.009 | 0.13 | 0.044 | 0.179 | 0.008 | 0.006 | 0.52 | 0.059 |
| % Time CPP < LLR-PAx | 0.211 | 0.173 | 0.337 | 0.041 | 0.016 | 0.001 | 0.038 | 0.007 | 0.036 |
| CPPopt-RAC | 0.006 | 0.002 | 0.071 | 0.007 | 0.01 | 0.05 | 0.004 | 0.015 | 0 |
| % Time ΔCPPopt-RAC > 5 mmHg | 0.014 | 0.158 | 0.13 | 0.001 | 0.011 | 0.012 | 0.071 | 0.52 | 0.043 |
| % Time ΔCPPopt-RAC > 10 mmHg | 0.006 | 0.128 | 0.155 | 0.046 | 0 | 0.049 | 0.031 | 0.52 | 0.012 |
| % Time ΔCPPopt-RAC < -5 mmHg | 0.034 | 0.108 | 0.186 | 0.022 | 0.014 | 0 | 0.055 | 0.001 | 0.01 |
| % Time ΔCPPopt-RAC < -10 mmHg | 0.031 | 0.025 | 0.081 | 0.009 | 0.001 | 0.003 | 0.074 | 0.002 | 0.019 |
| % Time CPP > ULR-RAC | 0.392 | 0.006 | 0.129 | 0.002 | 0.118 | 0.049 | 0.014 | 0.52 | 0.052 |
| % Time CPP < LLR-RAC | 0.124 | 0.108 | 0.213 | 0.005 | 0.124 | 0.025 | 0.051 | 0.016 | 0.031 |
| **Core + CT + ICP > 20 mmHg** | | | | | | | | | |
| **Variable** | **Age < 30** | | | **Age 30 – 60** | | | **Age > 60** | | |
|  | 1M 🡪 3M | 3M 🡪 6M | 1M 🡪 6M | 1M 🡪 3M | 3M 🡪 6M | 1M 🡪 6M | 1M 🡪 3M | 3M 🡪 6M | 1M 🡪 6M |
| CPPopt-PRx | 0.014 | 0 | 0.072 | 0.001 | 0.035 | 0.039 | 0 | 0.043 | 0.327 |
| % Time ΔCPPopt-PRx > 5 mmHg | 0.001 | 0.136 | 0.022 | 0.002 | 0.015 | 0.012 | 0 | 0.518 | 0 |
| % Time ΔCPPopt-PRx > 10 mmHg | 0.004 | 0.102 | 0.025 | 0.033 | 0.016 | 0.03 | 0 | 0.518 | 0.017 |
| % Time ΔCPPopt-PRx < -5 mmHg | 0.076 | 0.212 | 0.008 | 0.008 | 0.011 | 0.002 | 0 | 0 | 0.327 |
| % Time ΔCPPopt-PRx < -10 mmHg | 0.059 | 0.125 | 0.016 | 0.023 | 0.014 | 0.006 | 0 | 0.228 | 0.327 |
| % Time CPP > ULR-PRx | 0.323 | 0.002 | 0.151 | 0.004 | 0.129 | 0.031 | 0 | 0.202 | 0.128 |
| % Time CPP < LLR-PRx | 0.001 | 0.126 | 0.001 | 0.001 | 0.103 | 0 | 0 | 0.518 | 0.003 |
| CPPopt-PAx | 0.01 | 0.011 | 0.052 | 0.001 | 0.002 | 0.042 | 0 | 0.518 | 0.02 |
| % Time ΔCPPopt-PAx > 5 mmHg | 0.058 | 0.08 | 0.142 | 0.009 | 0.038 | 0.011 | 0 | 0.518 | 0.03 |
| % Time ΔCPPopt-PAx > 10 mmHg | 0.005 | 0.092 | 0.159 | 0.025 | 0.023 | 0.027 | 0 | 0.518 | 0 |
| % Time ΔCPPopt-PAx < -5 mmHg | 0.014 | 0.065 | 0.097 | 0.028 | 0.085 | 0.003 | 0 | 0.518 | 0.015 |
| % Time ΔCPPopt-PAx < -10 mmHg | 0 | 0.061 | 0.047 | 0.016 | 0.033 | 0.006 | 0 | 0.518 | 0.026 |
| % Time CPP > ULR-PAx | 0.163 | 0 | 0.073 | 0.033 | 0.203 | 0.012 | 0 | 0.518 | 0.103 |
| % Time CPP < LLR-PAx | 0.063 | 0.203 | 0.263 | 0.029 | 0.029 | 0 | 0 | 0.009 | 0.054 |
| CPPopt-RAC | 0.003 | 0.003 | 0.048 | 0.002 | 0.004 | 0.039 | 0 | 0.021 | 0.001 |
| % Time ΔCPPopt-RAC > 5 mmHg | 0.002 | 0.141 | 0.062 | 0.001 | 0.01 | 0.011 | 0 | 0.518 | 0.07 |
| % Time ΔCPPopt-RAC > 10 mmHg | 0.003 | 0.115 | 0.104 | 0.044 | 0 | 0.047 | 0 | 0.518 | 0.021 |
| % Time ΔCPPopt-RAC < -5 mmHg | 0 | 0.091 | 0.083 | 0.018 | 0.02 | 0 | 0 | 0.003 | 0 |
| % Time ΔCPPopt-RAC < -10 mmHg | 0.007 | 0.009 | 0.008 | 0.008 | 0.002 | 0.002 | 0 | 0.003 | 0.005 |
| % Time CPP > ULR-RAC | 0.224 | 0 | 0.056 | 0 | 0.157 | 0.078 | 0 | 0.518 | 0.109 |
| % Time CPP < LLR-RAC | 0.005 | 0.121 | 0.088 | 0 | 0.207 | 0.053 | 0 | 0.048 | 0.022 |
| **Core + CT + ICP > 22 mmHg** | | | | | | | | | |
| **Variable** | **Age < 30** | | | **Age 30 – 60** | | | **Age > 60** | | |
|  | 1M 🡪 3M | 3M 🡪 6M | 1M 🡪 6M | 1M 🡪 3M | 3M 🡪 6M | 1M 🡪 6M | 1M 🡪 3M | 3M 🡪 6M | 1M 🡪 6M |
| CPPopt-PRx | 0.013 | 0 | 0.072 | 0.001 | 0.034 | 0.037 | 0 | 0.045 | 0.333 |
| % Time ΔCPPopt-PRx > 5 mmHg | 0.001 | 0.137 | 0.018 | 0.002 | 0.016 | 0.011 | 0 | 0.519 | 0 |
| % Time ΔCPPopt-PRx > 10 mmHg | 0.003 | 0.104 | 0.022 | 0.032 | 0.016 | 0.029 | 0 | 0.519 | 0.024 |
| % Time ΔCPPopt-PRx < -5 mmHg | 0.108 | 0.222 | 0.013 | 0.008 | 0.012 | 0.002 | 0 | 0.519 | 0.333 |
| % Time ΔCPPopt-PRx < -10 mmHg | 0.078 | 0.135 | 0.023 | 0.024 | 0.016 | 0.007 | 0 | 0.231 | 0.333 |
| % Time CPP > ULR-PRx | 0.259 | 0.003 | 0.142 | 0.004 | 0.126 | 0.03 | 0 | 0.199 | 0.333 |
| % Time CPP < LLR-PRx | 0.002 | 0.129 | 0.001 | 0.001 | 0.108 | 0 | 0 | 0.101 | 0 |
| CPPopt-PAx | 0.016 | 0.01 | 0.052 | 0 | 0.002 | 0.038 | 0 | 0.028 | 0.018 |
| % Time ΔCPPopt-PAx > 5 mmHg | 0.056 | 0.08 | 0.127 | 0.009 | 0.037 | 0.011 | 0 | 0.519 | 0.019 |
| % Time ΔCPPopt-PAx > 10 mmHg | 0.01 | 0.094 | 0.151 | 0.025 | 0.023 | 0.027 | 0 | 0.519 | 0.001 |
| % Time ΔCPPopt-PAx < -5 mmHg | 0.017 | 0.066 | 0.085 | 0.026 | 0.089 | 0.003 | 0 | 0.04 | 0.014 |
| % Time ΔCPPopt-PAx < -10 mmHg | 0 | 0.062 | 0.04 | 0.014 | 0.035 | 0.006 | 0 | 0.519 | 0.028 |
| % Time CPP > ULR-PAx | 0.121 | 0 | 0.074 | 0.032 | 0.204 | 0.013 | 0 | 0.519 | 0.333 |
| % Time CPP < LLR-PAx | 0.044 | 0.199 | 0.208 | 0.028 | 0.03 | 0 | 0 | 0.01 | 0.065 |
| CPPopt-RAC | 0.008 | 0.002 | 0.054 | 0.002 | 0.003 | 0.035 | 0 | 0.015 | 0.001 |
| % Time ΔCPPopt-RAC > 5 mmHg | 0 | 0.142 | 0.048 | 0.001 | 0.009 | 0.011 | 0 | 0.519 | 0.049 |
| % Time ΔCPPopt-RAC > 10 mmHg | 0.004 | 0.115 | 0.085 | 0.044 | 0 | 0.046 | 0 | 0.519 | 0.012 |
| % Time ΔCPPopt-RAC < -5 mmHg | 0 | 0.092 | 0.076 | 0.016 | 0.022 | 0 | 0 | 0.001 | 0.001 |
| % Time ΔCPPopt-RAC < -10 mmHg | 0.012 | 0.01 | 0.005 | 0.007 | 0.002 | 0.001 | 0 | 0.002 | 0.007 |
| % Time CPP > ULR-RAC | 0.164 | 0 | 0.051 | 0 | 0.166 | 0.087 | 0 | 0.519 | 0.333 |
| % Time CPP < LLR-RAC | 0 | 0.131 | 0.078 | 0 | 0.213 | 0.056 | 0 | 0.017 | 0.029 |

*Core model consisted of age, admission Glasgow Coma Scale - motor score, and admission pupillary response. CT variables consisted of admission Marshall CT grade, presence of traumatic subarachnoid hemorrhage, and presence of epidural hematoma.*

*AMP = pulse amplitude of ICP, CPP = cerebral perfusion pressure, CPPopt = cerebral perfusion pressure optimum, ΔCPPopt = CPP - CPPopt, CT = computed tomography, GOSE = Glasgow Outcome Scale-Extended, ICP = intracranial pressure, IMPACT = International Mission for Prognosis and Analysis of Clinical Trials, LLR = lower limit of reactivity, PAx = pulse amplitude index, PRx = pressure reactivity index, RAC = correlation (R) between slow-waves of AMP (A) and CPP (C), ULR = upper limit of reactivity.*

Appendix Y. Extended Discussion

Through our investigation on the association between CPPopt metrics and improvement in outcome, we uncovered several interesting findings. In order to keep the discussion focused on the main findings of the study, and to keep the paper to an appropriate length, we left out a couple of interesting findings. Here, we discuss these additional findings. Firstly, the Mann-Whitney U and Chi-square testing of demographic and cerebrovascular reactivity metrics for *Improved* versus *Not Improved* produced results that are consistent with the existing literature. The association between increased age and worse outcome is well documented [1–3], while the association between length of hospital stay and improvement in outcome can be explained by the fact that those with poorer outcomes are more likely to pass away or be palliated and have invasive ICP monitoring discontinued. This explanation is supported by the fact that the analysis with those who died (GOSE 1) removed failed to find the association between length of hospital stay and outcome improvement statistically significant. Interestingly, ICP and CPP were not shown to be associated with outcome transition. This is likely attributable to the highly controlled nature of these parameters during therapeutic management. With regard to the cerebrovascular reactivity indices, our results support their well-established association with outcome [4–10].

In Supplemental Appendix Z, we present linear regressions and LOESS curves comparing the three cerebrovascular reactivity indices and their relationships with ICP. It is evident that a general positive correlation exists between each index and ICP. This can be understood by considering the impact that elevated ICP has on the cerebral vasculature. First, increased ICP can result a significant drop in CPP, due to their close relationship; CPP = MAP – ICP. The resulting hypoperfusion can be significant enough that it can exhaust cerebral autoregulatory reserves. Additionally, elevated pressure within the cranial cavity can compress blood vessels, further hindering proper functionality of autoregulatory mechanisms. The differences that are seen between the cerebrovascular reactivity indices in Supplemental Appendix W can be explained by differences in their derivations. While PRx uses ICP itself, and its relationship with MAP, PAx and RAC are more refined, using the fundamental amplitude of ICP (AMP) instead. When it comes to outcome prognostic ability, there are differences between the indices as well. Looking at the Mann-Whitney U and Chi-square testing results, only PAx and RAC were able to consistently produce statistically significant p-values, while PRx failed to produce any. This mirrors the findings of an earlier study from our lab [7], and suggests that PAx and RAC based metrics may be superior to PRx for predicting outcome transition. This is further supported by research suggesting that PAx is superior to PRx for long-term outcome prediction among patients with lower ICPs [1, 10, 11], and that RAC exhibits a more robust association with 6-month outcomes compared to both PRx and PAx [12].

References:

1. Batson C, Froese L, Gomez A, et al (2021) Impact of Age and Biological Sex on Cerebrovascular Reactivity in Adult Moderate/Severe Traumatic Brain Injury: An Exploratory Analysis. Neurotrauma Reports 2:488–501. https://doi.org/10.1089/neur.2021.0039

2. Batson C, Gomez A, Sainbhi AS, et al (2021) Association of Age and Sex With Multi-Modal Cerebral Physiology in Adult Moderate/Severe Traumatic Brain Injury: A Narrative Overview and Future Avenues for Personalized Approaches. Front Pharmacol 12:676154. https://doi.org/10.3389/fphar.2021.676154

3. Batson C, Stein KY, Gomez A, et al (2022) Intracranial Pressure–Derived Cerebrovascular Reactivity Indices, Chronological Age, and Biological Sex in Traumatic Brain Injury: A Scoping Review. Neurotrauma Rep 3:44–56. https://doi.org/10.1089/neur.2021.0054

4. Adams H, Donnelly J, Czosnyka M, et al (2017) Temporal profile of intracranial pressure and cerebrovascular reactivity in severe traumatic brain injury and association with fatal outcome: An observational study. PLoS Med 14:e1002353. https://doi.org/10.1371/journal.pmed.1002353

5. Bennis FC, Teeuwen B, Zeiler FA, et al (2020) Improving Prediction of Favourable Outcome After 6 Months in Patients with Severe Traumatic Brain Injury Using Physiological Cerebral Parameters in a Multivariable Logistic Regression Model. Neurocrit Care 33:542–551. https://doi.org/10.1007/s12028-020-00930-6

6. Sorrentino E, Diedler J, Kasprowicz M, et al (2012) Critical Thresholds for Cerebrovascular Reactivity After Traumatic Brain Injury. Neurocrit Care 16:258–266. https://doi.org/10.1007/s12028-011-9630-8

7. Stein KY, Froese L, Gomez A, et al (2022) Association between cerebrovascular reactivity in adult traumatic brain injury and improvement in patient outcome over time: an exploratory analysis. Acta Neurochir (Wien). https://doi.org/10.1007/s00701-022-05366-9

8. Zeiler FA, Donnelly J, Smielewski P, et al (2018) Critical Thresholds of Intracranial Pressure-Derived Continuous Cerebrovascular Reactivity Indices for Outcome Prediction in Noncraniectomized Patients with Traumatic Brain Injury. J Neurotrauma 35:1107–1115. https://doi.org/10.1089/neu.2017.5472

9. Zeiler FA, Ercole A, Beqiri E, et al (2020) Association between Cerebrovascular Reactivity Monitoring and Mortality Is Preserved When Adjusting for Baseline Admission Characteristics in Adult Traumatic Brain Injury: A CENTER-TBI Study. J Neurotrauma 37:1233–1241. https://doi.org/10.1089/neu.2019.6808

10. Zeiler FA, Ercole A, Cabeleira M, et al (2019) Univariate comparison of performance of different cerebrovascular reactivity indices for outcome association in adult TBI: a CENTER-TBI study. Acta Neurochir (Wien) 161:1217–1227. https://doi.org/10.1007/s00701-019-03844-1

11. Aries MJH, Czosnyka M, Budohoski KP, et al (2012) Continuous Monitoring of Cerebrovascular Reactivity Using Pulse Waveform of Intracranial Pressure. Neurocrit Care 17:67–76. https://doi.org/10.1007/s12028-012-9687-z

12. Zeiler FA, Donnelly J, Menon DK, et al (2018) A Description of a New Continuous Physiological Index in Traumatic Brain Injury Using the Correlation between Pulse Amplitude of Intracranial Pressure and Cerebral Perfusion Pressure. J Neurotrauma 35:963–974. https://doi.org/10.1089/neu.2017.5241

Appendix Z. Graphical Illustration of the Relationships Between ICP and Cerebrovascular Reactivity


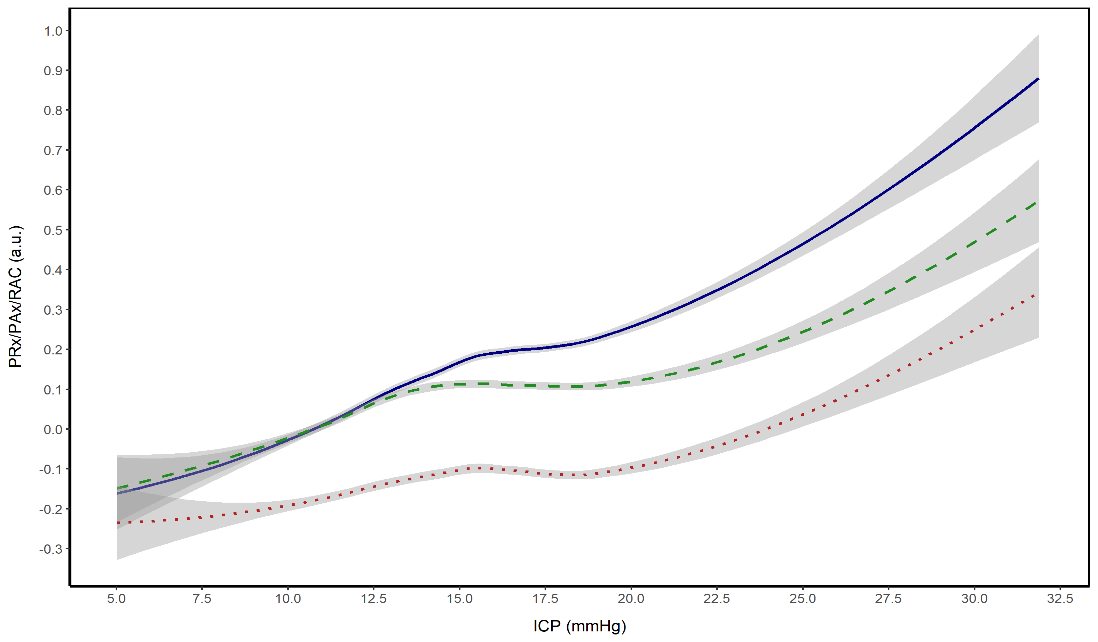

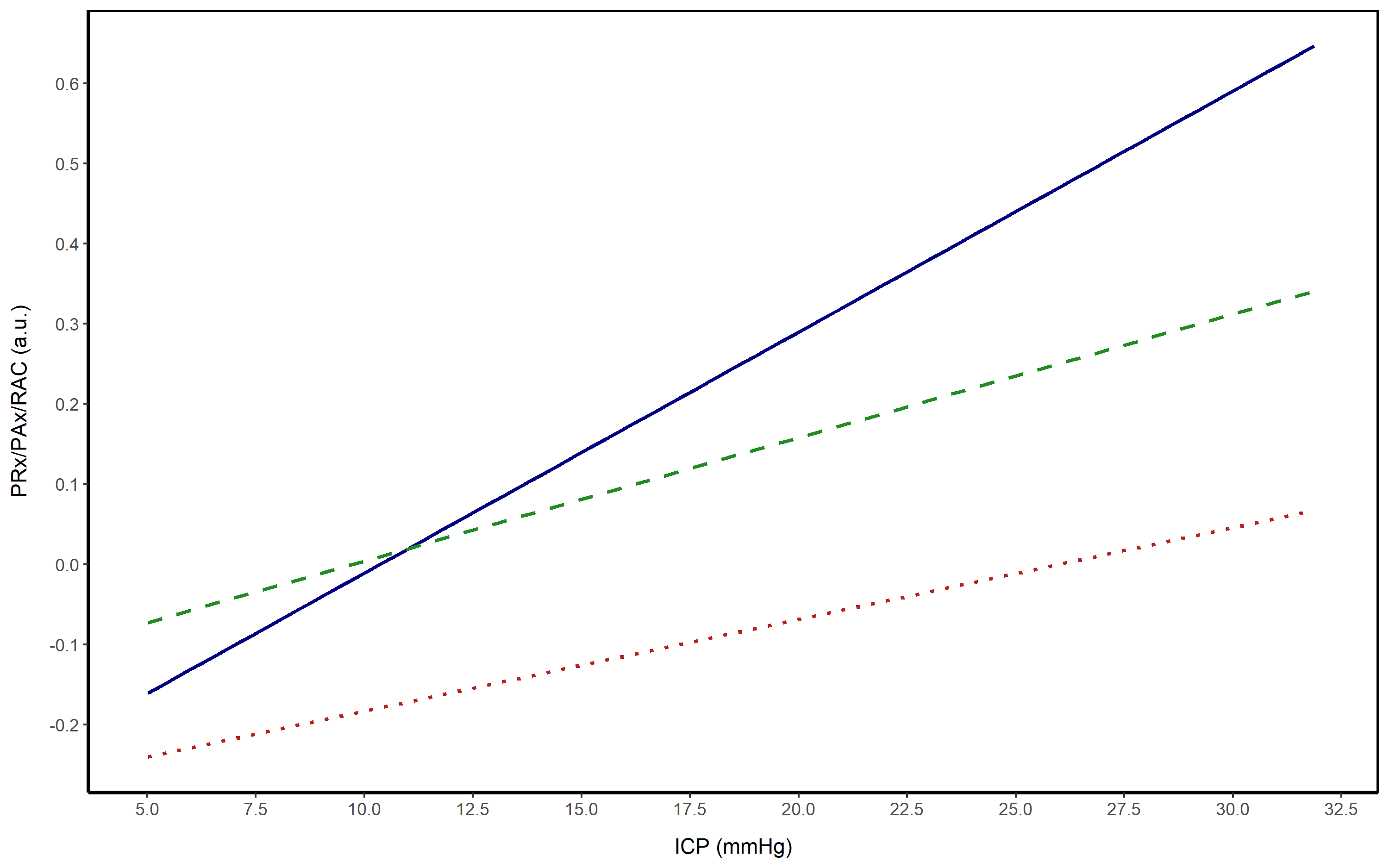


*The top panel presents linear regression relationships between ICP and PRx (solid line), ICP and PAx (dashed line), and ICP and RAC (dotted line). All three indices demonstrate a positive correlation with ICP. The bottom panel presents LOESS curves, with 95% confidence intervals, of the relationships between ICP and PRx (solid line), ICP and PAx (dashed line), and ICP and RAC (dotted line). Both graphs were created using the minute-by-minute physiology data from a single patient.*

*a.u. = arbitrary units, ICP = intracranial pressure, LOESS = locally estimated scatterplot smoothing, PAx = pulse amplitude index, PRx = pressure reactivity index, RAC = correlation (R) between slow-waves of AMP (A) and CPP (C).*
